# Investigating the causal links between inflammatory cytokines and scoliosis through bidirectional Mendelian randomization analysis

**DOI:** 10.1002/jsp2.70019

**Published:** 2024-12-11

**Authors:** Muradil Mardan, Mardan Mamat, Parhat Yasin, Xiaoyu Cai, Huoliang Zheng, Qingyin Xu, Shaokuan Song, Bo Li, Hao Cai, Pengbo Chen, Zeyu Lu, Shahna Omar, Shengdan Jiang, Leisheng Jiang, Xin‐feng Zheng

**Affiliations:** ^1^ Department of Spine Center Xinhua Hospital Affiliated to Shanghai Jiaotong University School of Medicine Shanghai China; ^2^ Department of Spine Surgery The First Affiliated Hospital of Xinjiang Medical University Urumqi China; ^3^ Department of Research Commercialization Wuxi China

**Keywords:** causal, genetic, GWAS, inflammatory factors, Mendelian randomization, scoliosis

## Abstract

**Background:**

Scoliosis, characterized by a lateral curvature of the spine, affects millions globally. The role of inflammatory cytokines in the pathogenesis of scoliosis is increasingly acknowledged, yet their causal relationships remain poorly defined.

**Aims:**

This study aims to explore the genetic‐level causal relationships between inflammatory cytokines and scoliosis utilizing bidirectional Mendelian randomization (MR) analysis.

**Materials and Methods:**

This study leverages genetic data from public Genome‐Wide Association Studies (GWAS). Bidirectional MR was employed to investigate the causal relationships between 44 inflammatory cytokines and scoliosis. The inflammatory cytokine data include 8293 Finnish individuals, while the scoliosis data consist of 165 850 participants of European descent, including 1168 scoliosis cases and 164 682 controls. Causal links were assessed using the inverse variance‐weighted method, supplemented by MR‐Egger, weighted median, and weighted mode analyses. Heterogeneity and pleiotropy were assessed using standard tests, with sensitivity analysis conducted through leave‐one‐out analysis.

**Results:**

Our analysis demonstrated a significant causal association between the cytokine Resistin (RETN) and the development of scoliosis (*p* = 0.024, OR 95% CI = 1.344 [1.039–1.739]). No other cytokines among the 44 studied showed significant associations.

**Discussion:**

The findings highlight the critical role of RETN in scoliosis progression and underscore the complex interplay of genetic and inflammatory pathways. Further research is needed to explore additional biomarkers and their mechanisms in scoliosis.

**Conclusion:**

This study provides evidence of a significant causal relationship between RETN and scoliosis, emphasizing its potential as a therapeutic target. These findings contribute to understanding scoliosis pathogenesis and pave the way for future research on inflammation‐related pathways and therapies.

## Graphical abstract

This study explores the genetic‐level causal relationships between inflammatory cytokines and scoliosis using bidirectional Mendelian randomization analysis. The results demonstrate a significant association between the cytokine Resistin (RETN) and scoliosis progression, suggesting RETN as a potential therapeutic target.

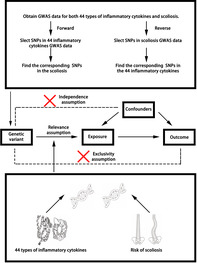

## 1 INTRODUCTION

Scoliosis is a common spinal disorder characterized by a lateral curvature of the spine in the coronal plane, often accompanied by vertebral rotation.^1^, ^2^ This condition impacts millions across all age groups globally, not only possibly resulting in morphological changes in the shoulders, waist, and chest but also associated with chronic pain and respiratory dysfunction.^3^, ^4^, ^5^ Although the exact pathogenesis of scoliosis remains not fully elucidated, recent studies have progressively uncovered the pivotal role that cytokines may play in its development.^6^

Cytokines are a category of small proteins secreted by immune system cells that can affect intercellular communication, regulate inflammatory responses, and influence cell growth. Within the pathophysiology of scoliosis, inflammation is considered a critical factor in disease progression. Studies have indicated that alterations in certain cytokine levels are closely linked to the incidence, progression, and severity of scoliosis. For instance, research has discovered variations in the expression levels of pro‐inflammatory cytokines such as tumor necrosis factor‐alpha (TNF‐α), interleukin‐6 (IL‐6), and interleukin‐17 (IL‐17) in individuals with scoliosis, which may relate to abnormal spinal curvature and bone remodeling.^7^, ^8^, ^9^, ^10^ Moreover, changes in the levels of anti‐inflammatory cytokines in patients with scoliosis suggest a complex network of inflammation regulation during disease progression. This network involves not only an imbalance between pro‐inflammatory and anti‐inflammatory factors but may also be associated with patients' genetic backgrounds, immune status, and other environmental factors.^11^, ^12^, ^13^ Thus, a deeper understanding of the role cytokines play in scoliosis is essential for unveiling the disease's pathogenesis and identifying potential therapeutic targets, with significant theoretical and practical implications.

Although existing research has offered valuable insights, research on the causal relationship between cytokines and scoliosis remains limited. Traditional epidemiological approaches have limitations in this area of research, as they often cannot eliminate the interference of confounding factors, nor can they determine the direction of causality.

The Mendelian randomization (MR) research design is grounded in the basic tenets of Mendelian genetics, wherein alleles from parents are randomly allocated to their offspring. This foundational principle supports the notion that an individual's genotype is a primary determinant of their phenotype, thereby suggesting that genotypes influence disease mainly through their effect on phenotypes. As a result, genotypes can act as instrumental variables (IVs) for deducing relationships between phenotypes and diseases. The MR approach uses genetic variants as IVs to build a model for inferring causal effects. In the field of epidemiological research, the challenge of confounding factors has often obscured the establishment of causal links between exposures and outcomes. Theoretically, the MR technique provides an effective strategy for minimizing the influence of confounders and eliminating the issues related to reverse causation. Presently, MR methodology is extensively applied in assessing causal connections between traits and diseases, as well as among various diseases. This study seeks to delve into the genetic‐level causal relationships between inflammatory cytokines and scoliosis using MR analysis, aiming to augment the body of research on scoliosis.

## 2 MATERIALS AND METHODS

### 2.1 Study design

**FIGURE 1:**
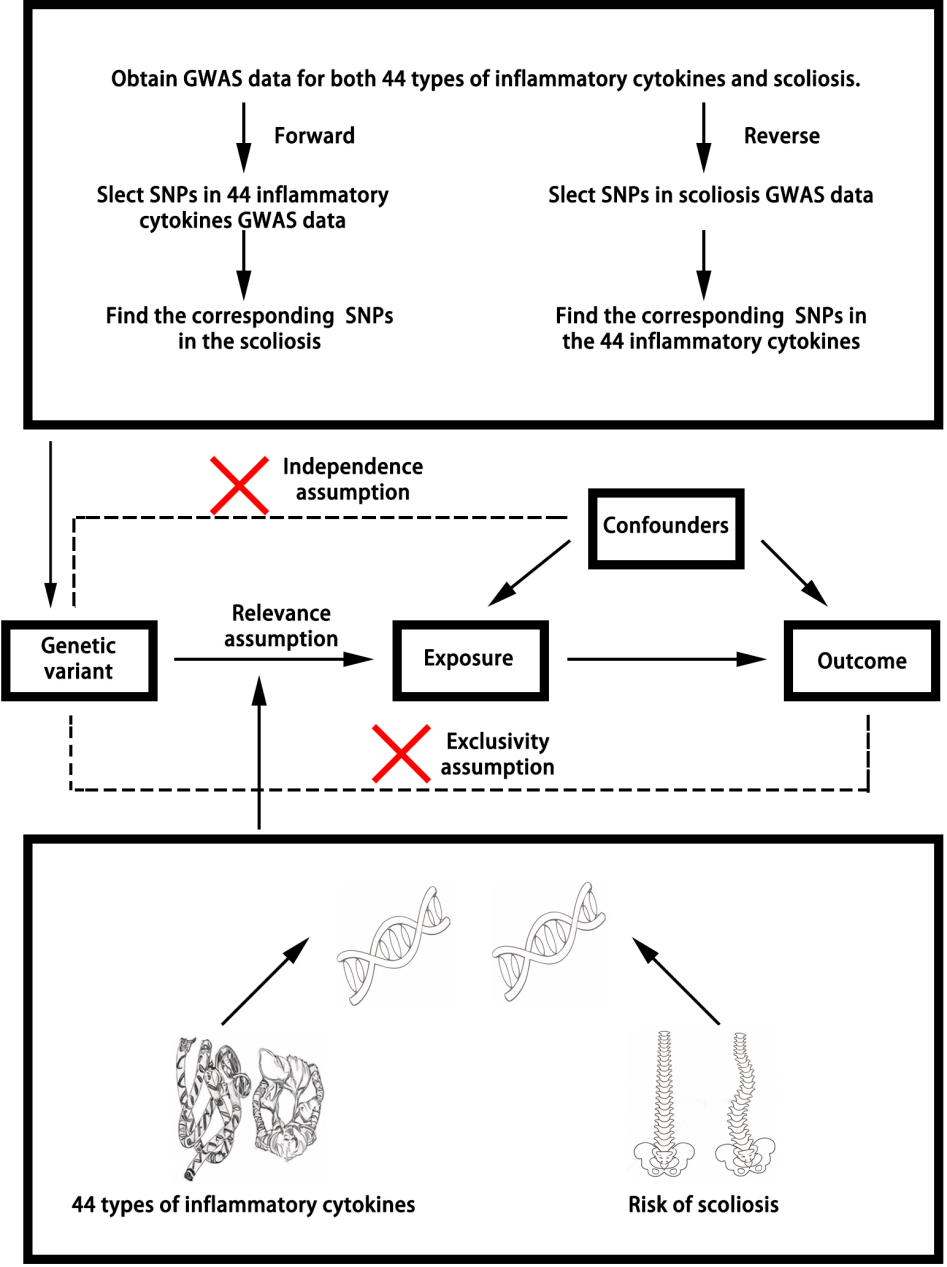
The basic principle of bidirectional Mendelian randomization (MR) analysis.

Utilizing summary data from a Genome‐Wide Association Study (GWAS) focused on inflammatory cytokines and scoliosis, this research undertook the task of identifying appropriate IVs for conducting MR analysis. The goal was to examine the causal link between inflammatory cytokines and scoliosis. This study was meticulously designed to align with the three fundamental assumptions integral to MR analysis: (1) The selected IVs exhibited a direct association with the exposure of interest; (2) the IVs were not correlated with any potential confounders; and (3) the influence of the IVs on the outcomes was exclusively mediated through their effect on the exposure (Figure 1). The datasets employed in this research were obtained from publicly available sources, and the originating studies had received ethical approval, including informed written consent.

### 2.2 GWAS summary data for inflammatory cytokines

The MR analysis leveraged GWAS data for 44 inflammatory cytokines, derived from a cohort of 8293 Finnish individuals. These data encompassed 44 specific inflammatory cytokines.^14^ Additionally, GWAS data for these cytokines are available in the IEU Open GWAS project database (https://gwas.mrcieu.ac.uk/), with specific GWAS IDs provided in Supplementary Material 1.

### 2.3 GWAS summary data for scoliosis

GWAS summary data for scoliosis were sourced from the IEU Open GWAS project (https://gwas.mrcieu.ac.uk/). The study concentrated on individuals of European descent, comprising 1168 cases and 164 682 controls of European ancestry, and encompassed a total of 16 380 270 Single Nucleotide Polymorphisms (SNPs). The corresponding GWAS IDs are detailed in Supplementary Material 2.

### 2.4 IV selection

Initially, we set a genome‐wide significance threshold of *p* < 5 × 10^−8^ to identify SNPs highly correlated with inflammatory cytokines and Scoliosis. Due to the limited number of SNPs identified for certain inflammatory cytokines and Scoliosis when considered as the exposure, we adjusted to a slightly higher cutoff (*p* < 5 × 10^−6^). To mitigate linkage disequilibrium and avoid bias, we conducted a linkage disequilibrium analysis with a threshold of (*r* ^2^ = 0.001, kb = 10 000), ensuring the independence of instrumental variables. Finally, to ensure a robust association with the exposure, we selected SNPs with an *F*‐statistic >10 as IVs. The *F*‐statistics were calculated using the formula *F* = *R* ^2^(*N* − *K* − 1)/*K*(1 – *R* ^2^), where *R* ^2^ was calculated using the formula *R* ^2^ = (2 × EAF × (1 − EAF) × Beta^2^)/[(2 × EAF × (1 − EAF) × Beta^2^) + (2 × EAF × (1 − EAF) × *N* × SE^2^)].^15^, ^16^

### 2.5 Statistical analysis

Utilizing the IVs previously selected, we performed bidirectional MR analyses to investigate the relationship between inflammatory cytokines and Scoliosis. These analyses were conducted using the TwoSampleMR and MRPRESSO packages within R software (version 4.1.2). Our MR analysis incorporated five distinct methodologies: the random‐effect Inverse Variance‐Weighted (IVW) method as the primary approach, supplemented by MR‐Egger, weighted median, simple mode, and weighted mode methods. The results from the random‐effects IVW were primarily relied upon in our study. Although each genetic variant met the criteria for IVs, the IVW method applied a meta‐analytical framework to integrate Wald ratio estimates of the causal link from multiple SNPs, thus offering a robust evaluation of the causal relationship between the exposure and outcome.^17^ The MR‐Egger was not dependent on nonzero mean pleiotropy, but it reduced statistical power.^18^ Weighted medians can provide strong estimates of effective IVs with at least a 50% weight.^19^ The simple mode is a model‐based assessment approach that offers pleiotropy robustness.^20^ For mode assessment, the weighted mode is sensitive to the hard throughput collection.^21^

We employed Cochran's Q statistic (MR‐IVW) and Rucker's Q statistic (MR‐Egger) to assess the heterogeneity in our MR analysis, where *p* > 0.05 indicated the absence of heterogeneity. The intercept test of MR‐Egger was utilized for detecting horizontal pleiotropy, with *p* > 0.05 indicating no horizontal pleiotropy.^22^ Additionally, MR pleiotropy residual sum and outlier (MR‐PRESSO) not only detects horizontal pleiotropy but also identifies outliers. The “leave‐one‐out” analysis was employed to examine whether an SNP influenced the causal relationship between inflammatory cytokines and Scoliosis.^17^ The global test in MR‐PRESSO analysis served as the horizontal pleiotropy test, with *p* > 0.05 indicating the absence of horizontal pleiotropy. Furthermore, the distortion test in MR‐PRESSO analysis was utilized to identify potential outliers in our MR analysis.^23^

## 3 RESULTS

### 3.1 Influence of 44 inflammatory cytokines on scoliosis

**FIGURE 2:**
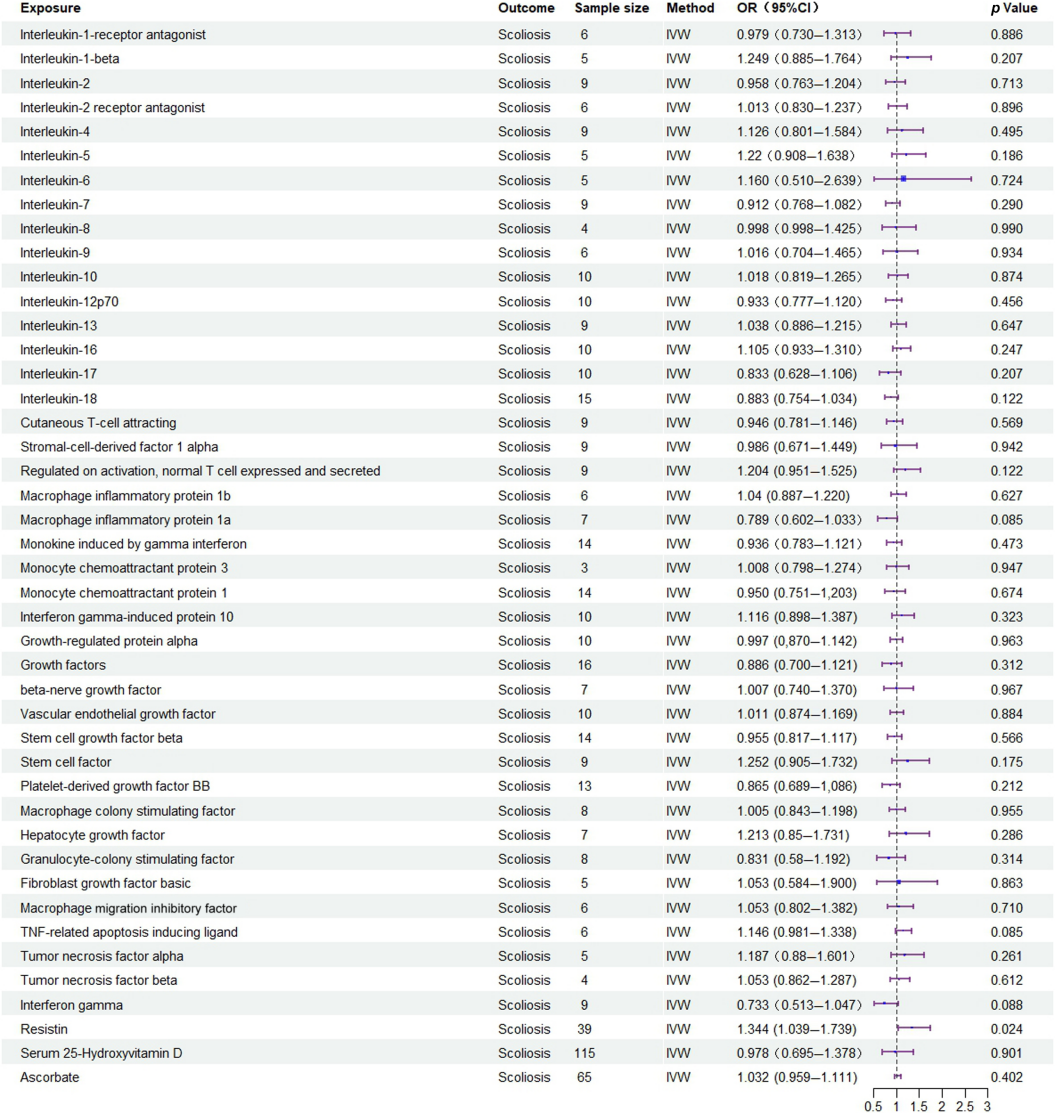
Mendelian randomization (MR) analysis between inflammatory cytokines and scoliosis (Exposure: Inflammatory cytokines, Outcome: Scoliosis).

**FIGURE 3:**
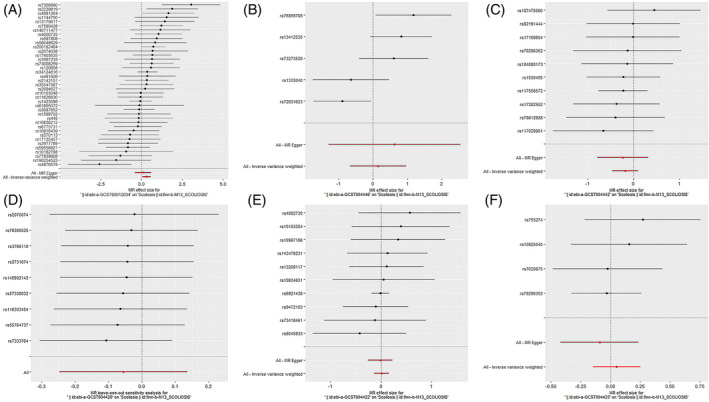
Forest plot of MR analysis: impact of inflammatory (A: RETN, B: IL‐6, C: IL‐17, D: CTACK, E: VEGF, F: TNFβ) cytokines on scoliosis. CTACK, cutaneous T‐cell attracting; IL‐17, interleukin‐17; IL‐6, interleukin‐6; MR, Mendelian randomization; RETN, Resistin; TNFβ, Tumor necrosis factor beta; VEGF, vascular endothelial growth factor.

**FIGURE 4:**
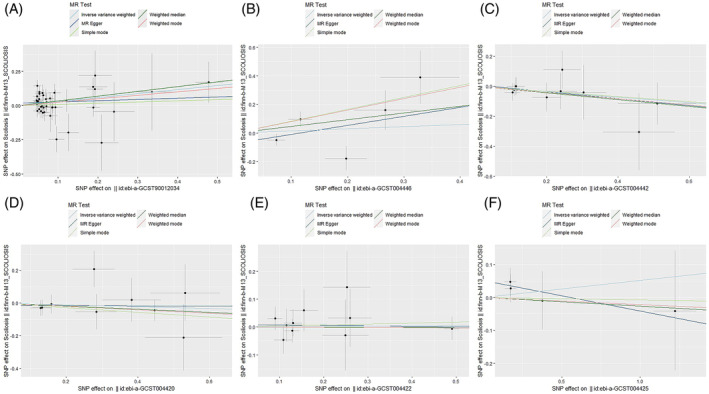
Scatter plot of MR analysis: impact of inflammatory (A: RETN, B: IL‐6, C: IL‐17, D: CTACK, E: VEGF, F: TNFβ) cytokines on scoliosis. CTACK, cutaneous T‐cell attracting; IL‐17, interleukin‐17; IL‐6, interleukin‐6; MR, Mendelian randomization; RETN, Resistin; TNFβ, Tumor necrosis factor beta; VEGF, vascular endothelial growth factor.

**FIGURE 5:**
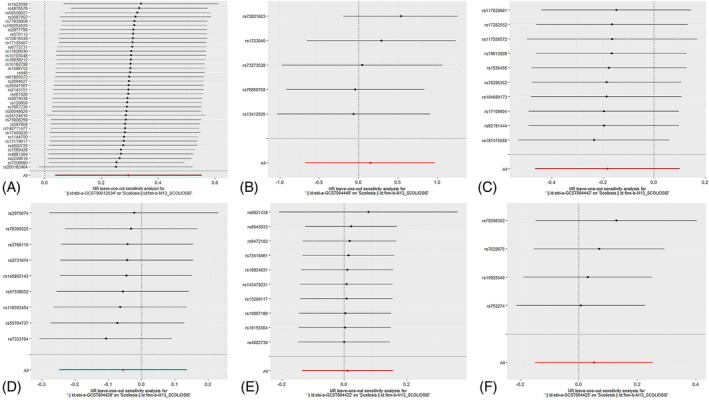
“Leave‐one‐out” analysis: impact of inflammatory (A: RETN, B: IL‐6, C: IL‐17, D: CTACK, E: VEGF, F: TNFβ) cytokines on scoliosis. CTACK, cutaneous T‐cell attracting; IL‐17, interleukin‐17; IL‐6, interleukin‐6; MR, Mendelian randomization; RETN, Resistin; TNFβ, Tumor necrosis factor beta; VEGF, vascular endothelial growth factor.

**FIGURE 6:**
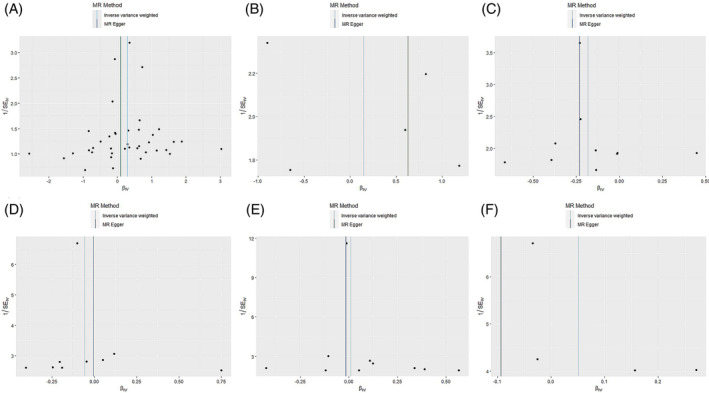
Funnel plot of MR analysis: impact of inflammatory (A: RETN, B: IL‐6, C: IL‐17, D: CTACK, E: VEGF, F: TNFβ) cytokines on scoliosis. CTACK, cutaneous T‐cell attracting; IL‐17, interleukin‐17; IL‐6, interleukin‐6; MR, Mendelian randomization; RETN, Resistin; TNFβ, Tumor necrosis factor beta; VEGF, vascular endothelial growth factor.

Among the inflammatory markers examined in this study, when the genome‐wide significance threshold was set at 5 × 10^−8^, four factors (namely MIP1β, TRAIL, VD, VC) exhibited a sufficient number of effective SNPs. For other inflammatory factors, we adopted a higher threshold of *p* < 5 × 10^−6^ to ensure an adequate number of SNPs for subsequent MR analysis. Additionally, all *F*‐statistics exceeded 10, suggesting a minimal likelihood of weak instrumental bias (all SNPs involved in MR analysis can be found in Supplementary Material 3). To evaluate the impact of 44 inflammatory cytokines on scoliosis, we primarily employed the IVW method, supplemented by MR‐Egger, weighted median, simple mode, and weighted mode. The IVW analysis revealed a significant positive correlation between Resistin (RETN) and scoliosis, with an odds ratio (OR) of 1.344 (95% CI: 1.039–1.739, *p* = 0.024). The remaining 43 inflammatory cytokines did not exhibit a positive or negative correlation with scoliosis (*p* > 0.05). The results of the IVW analysis for the 44 inflammatory cytokines can be found in Figure 2 (all Mendelian randomization analysis results, including MR‐Egger, weighted median, IVW, simple mode, and weighted mode, are available in Supplementary Material 3). Cochran's *Q* test did not detect any evidence of heterogeneity, and no significant intercept was observed, indicating the absence of pleiotropy. Additionally, the results of MR‐PRESSO indicated no horizontal pleiotropy in this MR analysis, and the MR‐PRESSO distortion test revealed no outliers. Table 1 provides a summary of the tests for multidirectionality and heterogeneity. (Due to the abundance of images, only partial results of the MR analysis forest plot, scatter plot, funnel plot, and “leave‐one‐out” analysis are shown in Figures 3, 4, 5, 6; the rest of the images can be found in Supplementary Material 3).

**TABLE 1:** Sensitivity analysis of the MR analysis results of exposures (inflammatory cytokines) and outcomes (scoliosis).

Exposure	Outcome	Heterogeneity test		Pleiotropy test	MR‐PRESSO	
		Cochran's *Q* test (*p* value)	Rucker's *Q* test (*p* value)	Egger intercept (*p* value)	Distortion test	Global test
		IVW	MR‐Egger	MR‐Egger	Outliers	*p* value
IL‐1RA	Scoliosis	0.508	0.426	0.543	NA	0.876
IL‐1β	Scoliosis	0.438	0.289	0.917	NA	0.432
IL‐2	Scoliosis	0.325	0.241	0.853	NA	0.479
IL‐2RA	Scoliosis	0.655	0.582	0.546	NA	0.687
IL‐4	Scoliosis	0.581	0.807	0.136	NA	0.652
IL‐5	Scoliosis	0.38	0.253	0.79	NA	0.334
IL‐6	Scoliosis	0.005	0.006	0.619	NA	0.001
IL‐7	Scoliosis	0.957	0.928	0.756	NA	0.981
IL‐8	Scoliosis	0.801	0.661	0.718	NA	0.809
IL‐9	Scoliosis	0.176	0.248	0.266	NA	0.313
IL‐10	Scoliosis	0.812	0.821	0.378	NA	0.718
IL‐12p70	Scoliosis	0.463	0.399	0.576	NA	0.607
IL‐13	Scoliosis	0.397	0.927	0.046	NA	0.61
IL‐16	Scoliosis	0.918	0.939	0.353	NA	0.943
IL‐17	Scoliosis	0.972	0.95	0.845	NA	0.894
IL‐18	Scoliosis	0.281	0.223	0.842	NA	0.411
CTACK	Scoliosis	0.655	0.56	0.765	NA	0.701
SDF‐1α	Scoliosis	0.828	0.744	0.956	NA	0.859
RANTES	Scoliosis	0.391	0.434	0.26	NA	0.499
MIP1β	Scoliosis	0.142	0.107	0.583	NA	0.254
MIP1α	Scoliosis	0.73	0.786	0.328	NA	0.737
MIG	Scoliosis	0.56	0.486	0.756	NA	0.661
MCP3	Scoliosis	0.986	0.867	0.983	NA	0.998
MCP1	Scoliosis	0.279	0.217	0.964	NA	0.121
IP‐10	Scoliosis	0.798	0.72	0.824	NA	0.722
GRPa	Scoliosis	0.85	0.786	0.772	NA	0.888
Eotaxin	Scoliosis	0.154	0.302	0.074	NA	0.236
βNGF	Scoliosis	0.206	0.136	0.848	NA	0.124
VEGF	Scoliosis	0.942	0.905	0.792	NA	0.861
SCGFβ	Scoliosis	0.402	0.945	0.014	NA	0.476
SCF	Scoliosis	0.796	0.725	0.697	NA	0.899
PDGFbb	Scoliosis	0.226	0.171	0.868	NA	0.28
MCSF	Scoliosis	0.775	0.675	0.869	NA	0.82
HGF	Scoliosis	0.636	0.508	0.928	NA	0.784
GCSF	Scoliosis	0.322	0.236	0.814	NA	0.435
bFGF	Scoliosis	0.159	0.094	0.779	NA	0.425
MIF	Scoliosis	0.773	0.642	0.93	NA	0.95
TRAIL	Scoliosis	0.501	0.369	0.82	NA	0.456
TNFα	Scoliosis	0.633	0.861	0.271	NA	0.657
TNFβ	Scoliosis	0.707	0.903	0.389	NA	0.777
IFN‐γ	Scoliosis	0.331	0.281	0.539	NA	0.188
RETN	Scoliosis	0.071	0.071	0.358	NA	0.053
VD	Scoliosis	0.594	0.568	0.952	NA	0.952
VC	Scoliosis	0.809	0.813	0.308	NA	0.801

Abbreviations: CTACK, cutaneous T‐cell attracting; IVW, Inverse Variance‐Weighted; MR, Mendelian randomization; MR‐PRESSO, MR pleiotropy residual sum and outlier; RETN, Resistin; TNF‐α, tumor necrosis factor‐alpha; TNFβ, Tumor necrosis factor beta; VEGF, vascular endothelial growth factor.

### 3.2 Influence of scoliosis on 44 inflammatory cytokines

**FIGURE 7:**
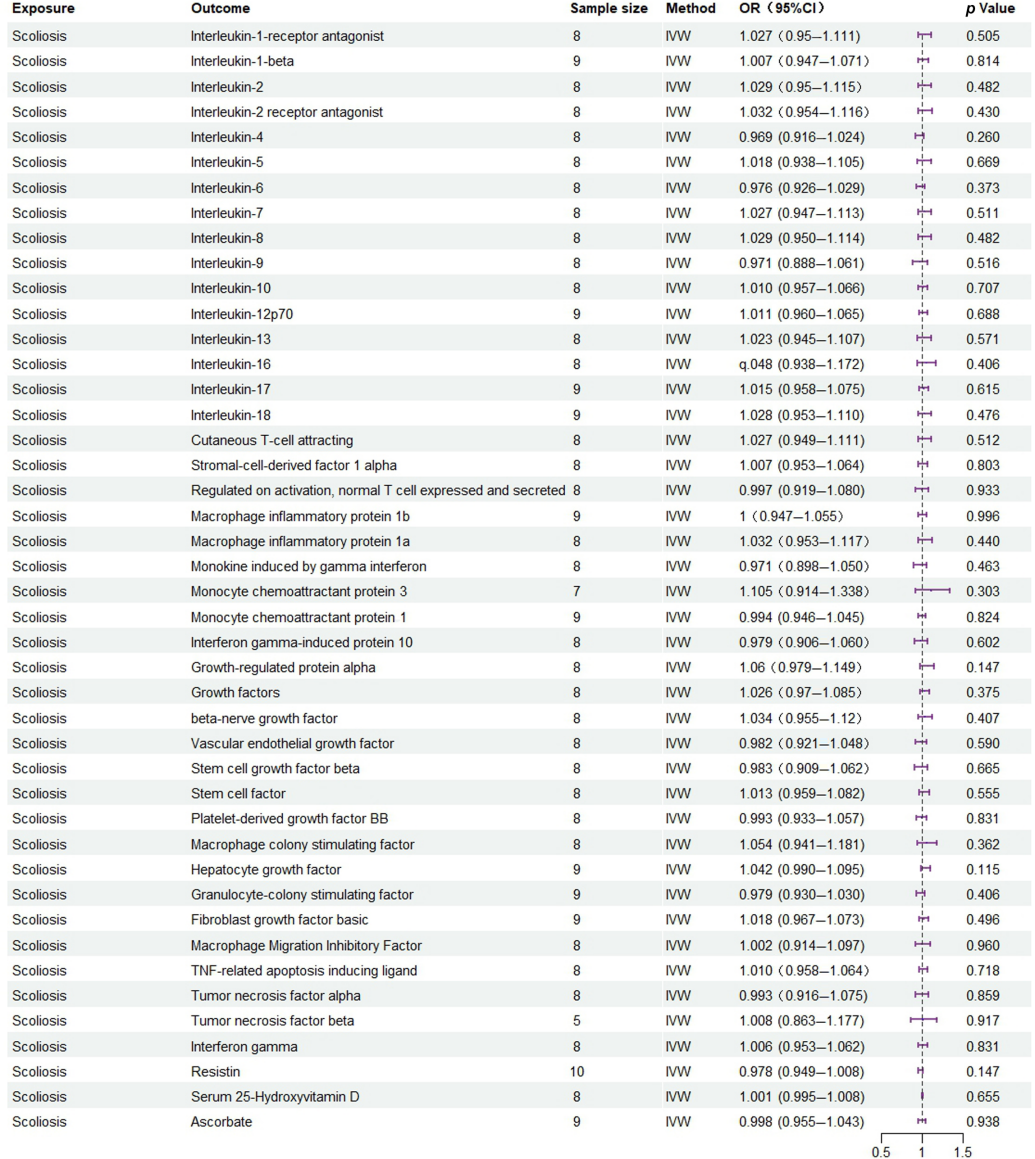
Mendelian randomization (MR) analysis between inflammatory cytokines and scoliosis (Exposure: Scoliosis, Outcome: Inflammatory cytokines).

**FIGURE 8:**
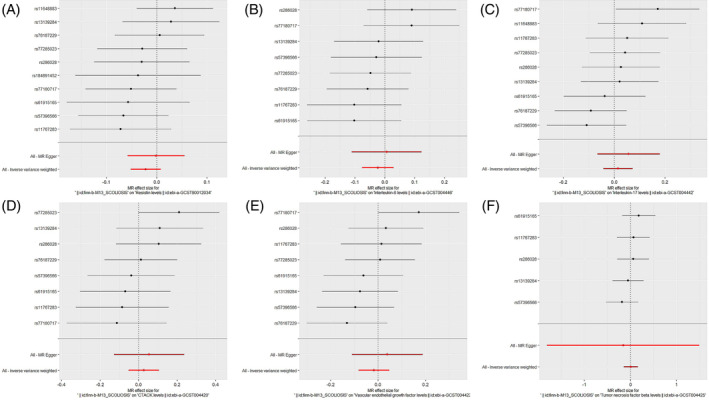
Forest plot of MR analysis: impact of scoliosis on inflammatory (A: RETN, B: IL‐6, C: IL‐17, D: CTACK, E: VEGF, F: TNFβ) cytokines. CTACK, cutaneous T‐cell attracting; IL‐17, interleukin‐17; IL‐6, interleukin‐6; MR, Mendelian randomization; RETN, Resistin; TNFβ, Tumor necrosis factor beta; VEGF, vascular endothelial growth factor.

**FIGURE 9:**
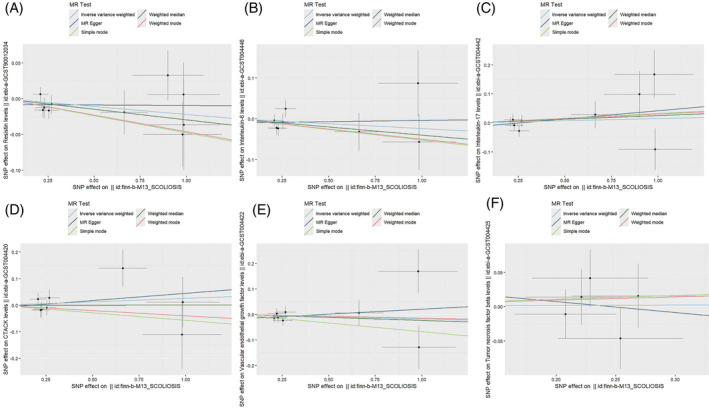
Scatter plot of MR analysis: impact of scoliosis on inflammatory (A: RETN, B: IL‐6, C: IL‐17, D: CTACK, E: VEGF, F: TNFβ) cytokines. CTACK, cutaneous T‐cell attracting; IL‐17, interleukin‐17; IL‐6, interleukin‐6; MR, Mendelian randomization; RETN, Resistin; TNFβ, Tumor necrosis factor beta; VEGF, vascular endothelial growth factor.

**FIGURE 10:**
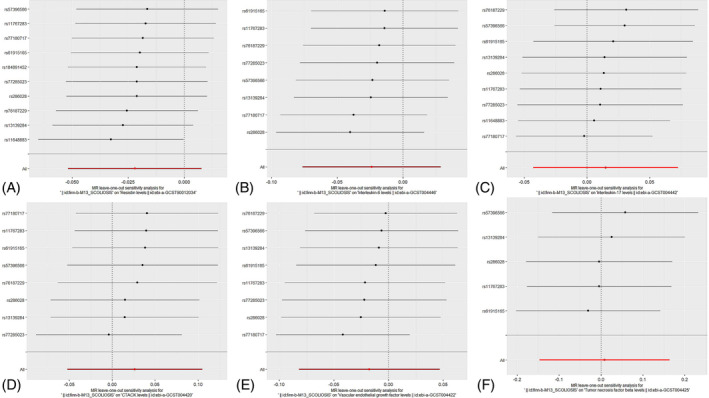
“Leave‐one‐out” analysis: impact of scoliosis on inflammatory (A: RETN, B: IL‐6, C: IL‐17, D: CTACK, E: VEGF, F: TNFβ) cytokines. CTACK, cutaneous T‐cell attracting; IL‐17, interleukin‐17; IL‐6, interleukin‐6; MR, Mendelian randomization; RETN, Resistin; TNFβ, Tumor necrosis factor beta; VEGF, vascular endothelial growth factor.

**FIGURE 11:**
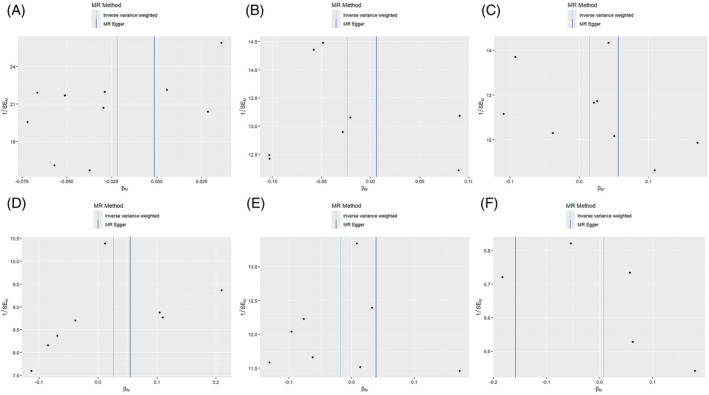
Funnel plot of MR analysis: impact of scoliosis on inflammatory (A: RETN, B: IL‐6, C: IL‐17, D: CTACK, E: VEGF, F: TNFβ) cytokines. CTACK, cutaneous T‐cell attracting; IL‐17, interleukin‐17; IL‐6, interleukin‐6; TNFβ, Tumor necrosis factor beta; MR, Mendelian randomization; RETN, Resistin; VEGF, vascular endothelial growth factor; VEGF, vascular endothelial growth factor.

Initially, the genome‐wide significance threshold was set at 5 × 10^−8^, but this cutoff yielded an insufficient number of SNPs for the MR analysis. Consequently, we adjusted the threshold to 5 × 10^−6^ to ensure a sufficient SNP count for robust analysis. Additionally, all *F*‐statistics exceeded the value of 10, suggesting a minimal likelihood of weak instrument bias (all SNPs involved in the MR analysis can be found in Supplementary Material 4). To assess the impact of scoliosis on 44 inflammatory cytokines, we primarily utilized the IVW method, supplemented by MR‐Egger, the weighted median, simple mode, and weighted mode. The IVW analysis showed that when scoliosis was considered as the exposure and the 44 inflammatory cytokines as the outcome, there was no evidence of either a positive or negative correlation (*p* > 0.05). The detailed results of the IVW analysis for the 44 cytokines are available in Figure 7. All results of the Mendelian randomization analysis, including MR‐Egger, weighted median, IVW, simple mode, and weighted mode, can be found in Supplementary Material 4. The Cochran's *Q* test did not detect any evidence of heterogeneity, and no significant intercept was observed, indicating the absence of pleiotropy. Moreover, the MR‐PRESSO results showed no horizontal pleiotropy in this analysis, and the MR‐PRESSO distortion test revealed no outliers. Table 2 provides a summary of the tests for multidirectionality and heterogeneity. (Due to the excessive number of images, only partial results of the MR analysis forest plot, scatter plot, funnel plot, and “leave‐one‐out” analysis are displayed in Figures 8, 9, 10, 11; the additional images are accessible in Supplementary Material 4).

**TABLE 2:** Sensitivity analysis of the MR analysis results of exposures (scoliosis) and outcomes (inflammatory cytokines).

Exposure	Outcome	Heterogeneity test		Pleiotropy test	MR‐PRESSO	
		Cochran's *Q* test (*p* value)	Rucker's *Q* test (*p* value)	Egger intercept (*p* value)	Distortion test	Global test
		IVW	MR‐Egger	MR‐Egger	Outliers	*p* value
Scoliosis	IL‐1RA	0.75	0.796	0.324	NA	0.769
Scoliosis	IL‐1β	0.723	0.639	0.712	NA	0.725
Scoliosis	IL‐2	0.682	0.593	0.674	NA	0.693
Scoliosis	IL‐2RA	0.736	0.725	0.428	NA	0.723
Scoliosis	IL‐4	0.357	0.264	0.821	NA	0.393
Scoliosis	IL‐5	0.962	0.943	0.645	NA	0.967
Scoliosis	IL‐6	0.486	0.407	0.594	NA	0.521
Scoliosis	IL‐7	0.884	0.925	0.34	NA	0.878
Scoliosis	IL‐8	0.814	0.728	0.785	NA	0.8
Scoliosis	IL‐9	0.252	0.598	0.08	NA	0.263
Scoliosis	IL‐10	0.573	0.526	0.477	NA	0.607
Scoliosis	IL‐12p70	0.375	0.418	0.261	NA	0.395
Scoliosis	IL‐13	0.828	0.916	0.262	NA	0.849
Scoliosis	IL‐16	0.059	0.083	0.299	NA	0.075
Scoliosis	IL‐17	0.263	0.235	0.473	NA	0.281
Scoliosis	IL‐18	0.695	0.79	0.239	NA	0.702
Scoliosis	CTACK	0.441	0.344	0.745	NA	0.45
Scoliosis	SDF‐1α	0.425	0.54	0.206	NA	0.445
Scoliosis	RANTES	0.637	0.761	0.226	NA	0.614
Scoliosis	MIP1β	0.308	0.225	0.917	NA	0.303
Scoliosis	MIP1α	0.689	0.794	0.247	NA	0.732
Scoliosis	MIG	0.593	0.509	0.247	NA	0.552
Scoliosis	MCP3	0.797	0.159	0.467	NA	0.173
Scoliosis	MCP1	0.852	0.82	0.543	NA	0.842
Scoliosis	IP‐10	0.426	0.392	0.433	NA	0.457
Scoliosis	GRPa	0.568	0.549	0.406	NA	0.575
Scoliosis	Eotaxin	0.337	0.523	0.146	NA	0.373
Scoliosis	βNGF	0.954	0.912	0.898	NA	0.956
Scoliosis	VEGF	0.269	0.247	0.442	NA	0.285
Scoliosis	SCGFβ	0.44	0.517	0.242	NA	0.432
Scoliosis	SCF	0.233	0.159	0.928	NA	0.249
Scoliosis	PDGFbb	0.189	0.127	0.888	NA	0.18
Scoliosis	MCSF	0.194	0.154	0.582	NA	0.216
Scoliosis	HGF	0.416	0.358	0.537	NA	0.444
Scoliosis	GCSF	0.602	0.524	0.62	NA	0.598
Scoliosis	bFGF	0.816	0.731	0.9	NA	0.843
Scoliosis	MIF	0.234	0.161	0.892	NA	0.25
Scoliosis	TRAIL	0.553	0.672	0.223	NA	0.554
Scoliosis	TNFα	0.633	0.584	0.495	NA	0.649
Scoliosis	TNFβ	0.669	0.508	0.855	NA	0.686
Scoliosis	IFN‐γ	0.453	0.355	0.741	NA	0.47
Scoliosis	RETN	0.699	0.681	0.426	NA	0.703
Scoliosis	VD	0.485	0.619	0.202	NA	0.535
Scoliosis	VC	0.943	0.915	0.665	NA	0.938

Abbreviations: IVW, Inverse Variance‐Weighted; MR, Mendelian randomization; MR‐PRESSO, MR pleiotropy residual sum and outlier; RETN, Resistin; TNF‐α, tumor necrosis factor‐alpha.

## 4 DISCUSSION

Our study employed a bidirectional Mendelian randomization analysis to explore the causal relationship between inflammatory cytokines and the development of scoliosis. The results indicated a significant positive correlation between RETN and the progression of scoliosis. This finding aligns with recent research on the role of cytokines in scoliosis, suggesting that inflammatory pathways may play a crucial role in its pathomechanism. Research by M Constantine Samaan and colleagues has previously explored the role of the immune system in adolescent idiopathic scoliosis (AIS), proposing that the association between immunometabolic processes and scoliosis might be mediated through inflammatory pathways.^24^ In this study, the discovery of RETN presents new potential therapeutic targets for inflammation modulation. To date, direct research linking RETN and scoliosis has been relatively scarce. RETN is commonly associated with inflammatory states, insulin resistance, and other metabolic disorders. Research by Ivan Nagaev and colleagues has emphasized that in humans, RETN is primarily regulated by CEBPE—a transcription factor in myeloid cells. This regulation underscores RETN's significant role within the immune system.^25^ Research by Yanran Li and colleagues has shown that human RETN acts as a pro‐inflammatory molecule, impacting a variety of chronic inflammatory diseases, metabolic disorders, and cancers, which highlights its potential role in inflammatory pathways.^26^ Additionally, insights from Md S Jamaluddin and others suggest that RETN serves both as an immune modulator and an antimicrobial agent, enhancing our understanding of how it might influence diseases characterized by immune dysregulation.^27^ These findings underscore the potential indirect impact of RETN on spinal health. Nevertheless, the specific role of RETN in the pathology of scoliosis remains unclear, necessitating further research to explore this. Recent studies have explored RETN as a therapeutic target in a variety of conditions, particularly those involving chronic inflammation and metabolic dysregulation. For example, interventions targeting RETN have been shown to reduce inflammation and improve outcomes in diseases such as insulin resistance, obesity, and cardiovascular disorders. Yang et al. demonstrated that the endocannabinoid system regulates resistin production in humans, linking it to insulin resistance and inflammation in conditions such as obesity.^28^ This highlights the potential of resistin as a broader therapeutic target beyond its metabolic roles. Additionally, research by Akbari et al. found that the injection of resistin into specific brain regions induced cardiovascular responses, further emphasizing its systemic effects and potential implications for treating cardiovascular diseases.^29^ Behrouzifar et al. showed that resistin administration in a cerebral ischemia mouse model reduced infarct size and improved neurological function through its anti‐apoptotic and anti‐inflammatory properties.^30^ Srikanth et al. also suggested resistin as a plausible therapeutic target in psoriasis, a chronic inflammatory skin disorder, underscoring resistin's role in modulating inflammation across diverse conditions.^31^ Recent findings indicate that adipokines like resistin may contribute to the development and progression of AIS. Normand et al. identified elevated levels of resistin in AIS patients, particularly in those with more severe spinal curvature, suggesting a potential role for resistin in modulating the inflammatory and metabolic pathways that underlie scoliosis progression.^32^ While no specific RETN‐targeted therapies have been developed for scoliosis, its involvement in modulating immune responses and systemic inflammation makes it a promising candidate for future therapeutic strategies. Future studies that reveal the potential connection between RETN and the progression of scoliosis could provide new insights into the complex pathophysiology of the condition and may guide the development of new therapeutic strategies. Particularly considering RETN's role in modulating inflammation and immune responses, understanding its expression and activity in patients with scoliosis could be crucial for disease management.

However, no significant associations were observed between the other 43 inflammatory cytokines and scoliosis, which may suggest that the relationship between inflammatory cytokines and scoliosis is more complex than the effect of any single cytokine. This complexity may involve interactions between multiple cytokines and other unknown biological mechanisms. In further discussions of the role of inflammatory cytokines in scoliosis, it is noteworthy that the impact of exercise‐induced cytokine changes on musculoskeletal health is increasingly being studied. Particularly in diseases like osteoarthritis, exercise‐induced cytokine changes may help modulate pathological processes, influencing the inflammatory environment and disease progression.^33^ These findings not only provide a new perspective on understanding the role of inflammatory cytokines in scoliosis but may also offer clues for the treatment of other related diseases. Additionally, genetic factors play a crucial role in the development of scoliosis. Recent systematic reviews have revealed multiple SNPs associated with the risk of AIS, particularly SNPs near the *LBX1* and *GPR126* genes showing significant associations.^34^ These studies highlight the importance of increasing cohort diversity and selecting appropriate control groups in different populations to better understand the impact of genetic backgrounds on the development of scoliosis. As mentioned above, the role of inflammatory cytokines is not limited to the inflammation process alone; they play a complex role in disease models influenced by both genetic and environmental factors. By thoroughly investigating the relationship between these cytokines and scoliosis, we can provide a scientific basis for developing more targeted therapeutic strategies.

A major limitation of this study is that the sample predominantly consists of individuals of European descent, which may limit the generalizability of the results to other populations. Future research should validate these findings across more diverse ethnic and geographic populations to enhance the study's external validity. In particular, the genetic architecture of inflammatory cytokines and scoliosis may differ between populations with distinct genetic backgrounds, such as Asian, African, and Latin American populations, due to variations in allele frequencies and gene‐environment interactions. For instance, certain environmental factors like nutrition, physical activity, and exposure to infections, which may differ significantly across regions, could influence the expression of cytokines and the progression of scoliosis.^35^, ^36^ Thus, replicating this study in populations with different genetic profiles and environmental contexts would be crucial to assess whether the causal relationships identified in this study hold across diverse settings. Moreover, exploring how genetic diversity impacts the effect of cytokines on scoliosis could provide insights into population‐specific treatment strategies. Furthermore, the dataset we utilized from the OPEN GWAS database for scoliosis does not provide a detailed classification of scoliosis types (e.g., congenital scoliosis, AIS, or degenerative scoliosis). As a result, it was not possible to distinguish the impact of inflammatory cytokines on different types of scoliosis in terms of onset and severity. This is acknowledged as another limitation of the current study, and future research incorporating more detailed scoliosis classifications would be beneficial for understanding the differential impact of cytokines on various scoliosis subtypes. Additionally, although high thresholds were used to select instrumental variables, caution is still needed to mitigate potential biases due to weak instrumental variables and genetic diversity.^37^

Future research should focus on further exploring the specific pathways through which resistin exerts its effects, particularly those involved in inflammation and immune regulation. One key pathway is the Toll‐like receptor (TLR) signaling pathway, where resistin has been shown to activate TLR4, leading to the downstream activation of the NF‐κB pathway. This cascade promotes the expression of pro‐inflammatory cytokines and contributes to chronic inflammation. Investigating the role of the TLR4/NF‐κB pathway in scoliosis could provide new insights into how resistin influences inflammation‐driven disease progression.^38^, ^39^, ^40^ Another important pathway involves resistin's interaction with the AMPK (AMP‐activated protein kinase) signaling pathway, which is crucial for regulating cellular energy balance and metabolic homeostasis. Disruption of this pathway by resistin has been linked to metabolic disorders, and exploring how resistin‐mediated AMPK dysregulation might affect scoliosis progression could open new avenues for research, particularly in understanding the link between metabolic dysfunction and spinal deformities.^41^, ^42^ Moreover, resistin's influence on adipogenesis and extracellular matrix (ECM) remodeling through pathways such as the TGF‐β/SMAD signaling cascade may also play a significant role in scoliosis development. Future studies could explore how resistin modulates ECM production and degradation, contributing to the structural changes observed in scoliosis.^43^ Animal models should be used to further investigate these pathways, focusing on how modulating resistin‐related signaling could slow or prevent scoliosis progression. Clinical trials targeting these pathways, particularly through inhibitors of TLR4 or NF‐κB, could also be designed to assess the efficacy of treatments aimed at reducing resistin‐driven inflammation and its impact on spinal deformity progression.

In summary, the bidirectional MR analysis approach employed in this study provides a new perspective for analyzing the directionality of the relationship between inflammatory cytokines and scoliosis. Traditional observational studies often struggle to discern the direction of causality, whereas our methodology uses genetic variation as a natural experiment, helping to overcome this limitation to some extent. Moreover, this study not only enhances our understanding of the inflammatory mechanisms potentially involved in the pathophysiology of scoliosis but also provides a scientific basis for the development of targeted therapeutic strategies. Future research should further explore other potential inflammatory biomarkers and validate their effects in animal models and clinical trials to develop new treatment methods.

## 5 CONCLUSIONS

Our study employed a bidirectional MR analysis approach to explore the causal links between inflammatory cytokines and scoliosis. We found a significant association between RETN and the progression of scoliosis, highlighting its potential role in the disease's pathophysiology. While RETN emerged as a key factor, other studied cytokines did not show significant associations, suggesting a complex interplay of multiple factors in scoliosis development. These findings provide a foundation for further research to uncover the detailed mechanisms of scoliosis and develop targeted therapeutic strategies. Importantly, the identification of RETN as a key player in scoliosis progression opens new avenues for potential clinical interventions. Future therapies could focus on modulating RETN‐related pathways to mitigate inflammation and slow or prevent disease progression, offering new hope for improved scoliosis treatment strategies.

## AUTHOR CONTRIBUTIONS

Each author has participated in the drafting and critical revision of the manuscript and has approved the final version.

## FUNDING INFORMATION

This study received no financial support. The authors declare that there are no potential conflicts of interest or biases associated with the conduct of this study.

## CONFLICT OF INTEREST STATEMENT

The authors declare no conflicts of interest.

## Supporting information

Supplementary Material 1.

Supplementary Material 2.

Supplementary Material 3.

Supplementary Material 4.
