# Supplementary figures and images for: Investigating the causal links between inflammatory cytokines and scoliosis through bidirectional Mendelian randomization analysis (part 2 of 3)
Source: JOR Spine. 2024 Dec 11;7(4):e70019. doi: 10.1002/jsp2.70019 (PMC11632254; doi:10.1002/jsp2.70019)

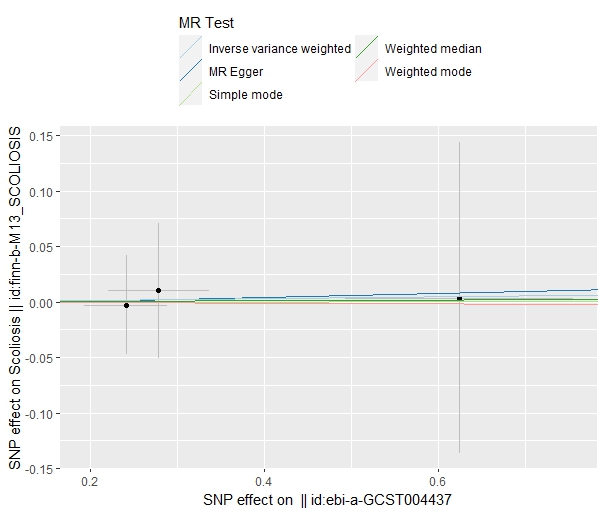

Supplement: Supplementary file 3 — Supplementary Material 3. [file JSP2-7-e70019-s004.zip › Supplementary Material 3/Exposureú║inflammatory cytokinesú1⁄4Outcomeú║Scoliosis - ╕▒▒╛/MCP3/Supplementary Material 3 MCP3 1.jpeg]

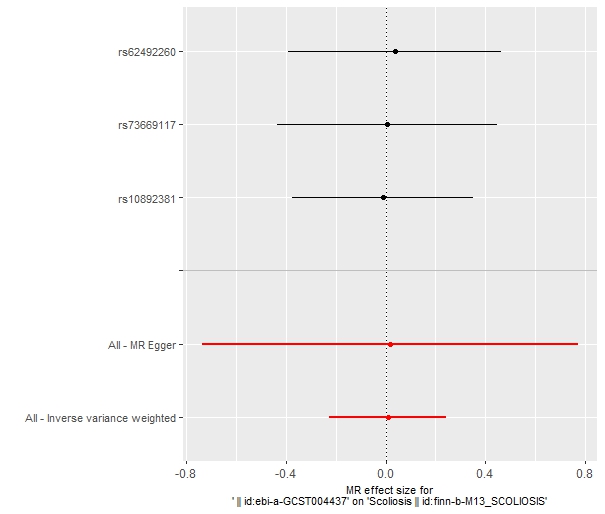

Supplement: Supplementary file 3 — Supplementary Material 3. [file JSP2-7-e70019-s004.zip › Supplementary Material 3/Exposureú║inflammatory cytokinesú1⁄4Outcomeú║Scoliosis - ╕▒▒╛/MCP3/Supplementary Material 3 MCP3 2.jpeg]

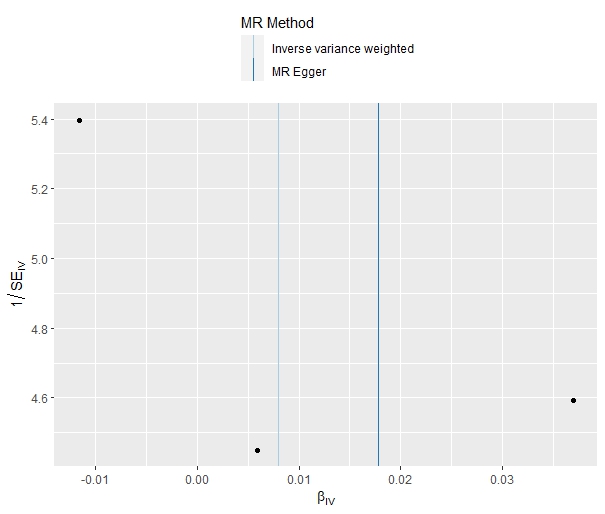

Supplement: Supplementary file 3 — Supplementary Material 3. [file JSP2-7-e70019-s004.zip › Supplementary Material 3/Exposureú║inflammatory cytokinesú1⁄4Outcomeú║Scoliosis - ╕▒▒╛/MCP3/Supplementary Material 3 MCP3 3.jpeg]

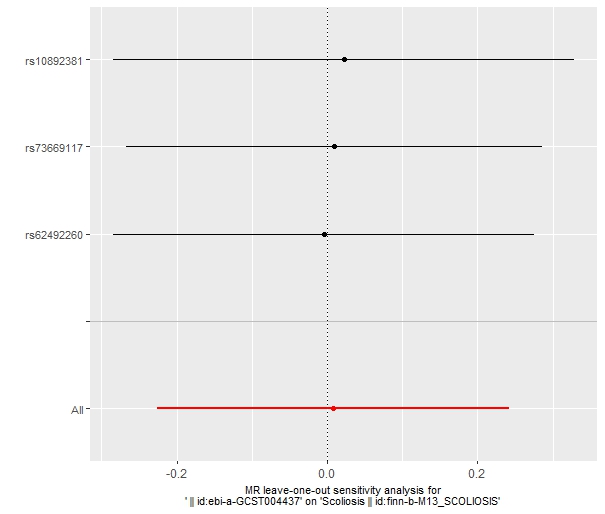

Supplement: Supplementary file 3 — Supplementary Material 3. [file JSP2-7-e70019-s004.zip › Supplementary Material 3/Exposureú║inflammatory cytokinesú1⁄4Outcomeú║Scoliosis - ╕▒▒╛/MCP3/Supplementary Material 3 MCP3 4.jpeg]

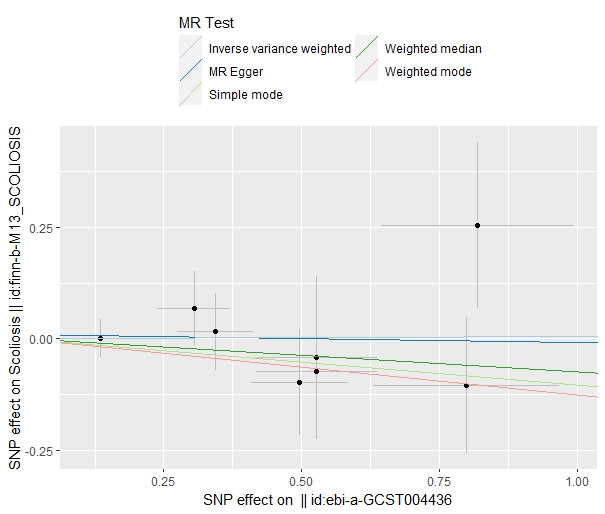

Supplement: Supplementary file 3 — Supplementary Material 3. [file JSP2-7-e70019-s004.zip › Supplementary Material 3/Exposureú║inflammatory cytokinesú1⁄4Outcomeú║Scoliosis - ╕▒▒╛/MCSF/Supplementary Material 3 MCSF 1.jpeg]

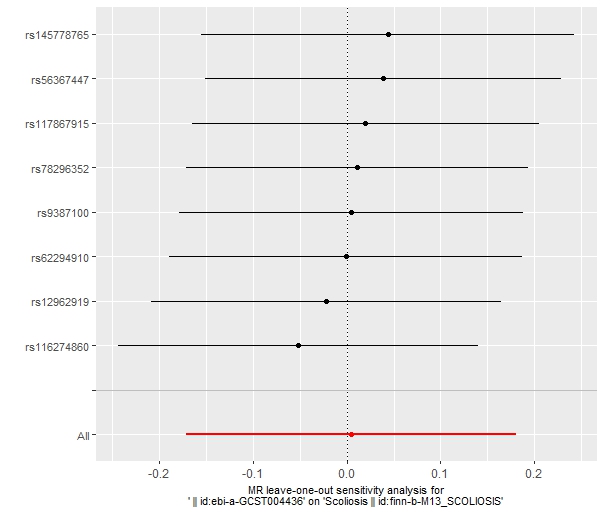

Supplement: Supplementary file 3 — Supplementary Material 3. [file JSP2-7-e70019-s004.zip › Supplementary Material 3/Exposureú║inflammatory cytokinesú1⁄4Outcomeú║Scoliosis - ╕▒▒╛/MCSF/Supplementary Material 3 MCSF 2.jpeg]

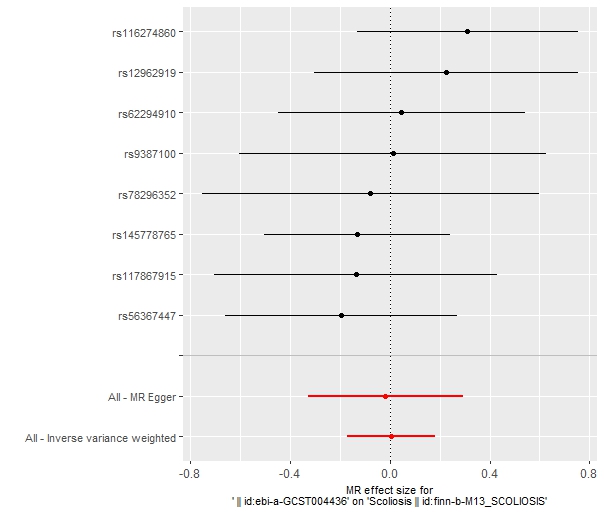

Supplement: Supplementary file 3 — Supplementary Material 3. [file JSP2-7-e70019-s004.zip › Supplementary Material 3/Exposureú║inflammatory cytokinesú1⁄4Outcomeú║Scoliosis - ╕▒▒╛/MCSF/Supplementary Material 3 MCSF 3.jpeg]

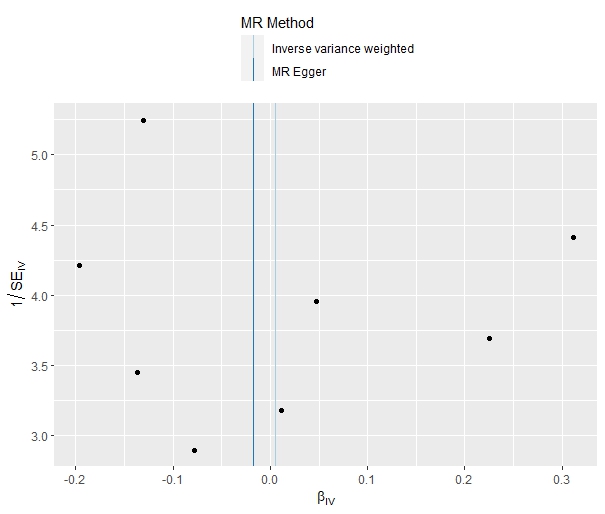

Supplement: Supplementary file 3 — Supplementary Material 3. [file JSP2-7-e70019-s004.zip › Supplementary Material 3/Exposureú║inflammatory cytokinesú1⁄4Outcomeú║Scoliosis - ╕▒▒╛/MCSF/Supplementary Material 3 MCSF 4.jpeg]

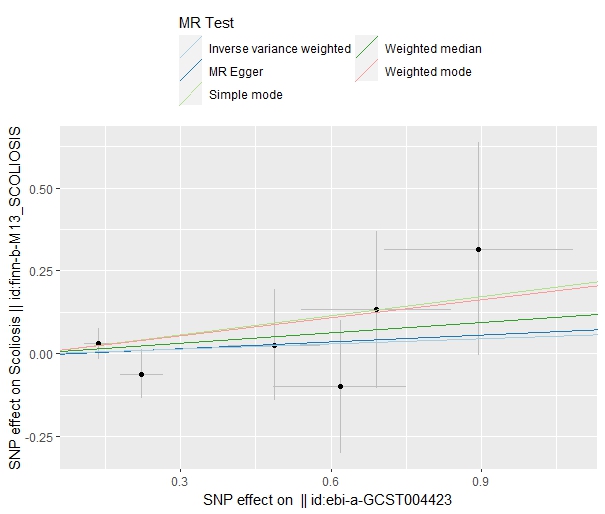

Supplement: Supplementary file 3 — Supplementary Material 3. [file JSP2-7-e70019-s004.zip › Supplementary Material 3/Exposureú║inflammatory cytokinesú1⁄4Outcomeú║Scoliosis - ╕▒▒╛/MIF/Supplementary Material 3 MIF 1.jpeg]

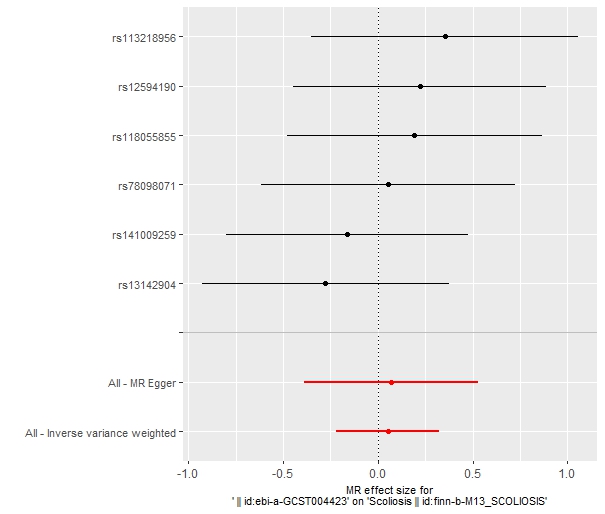

Supplement: Supplementary file 3 — Supplementary Material 3. [file JSP2-7-e70019-s004.zip › Supplementary Material 3/Exposureú║inflammatory cytokinesú1⁄4Outcomeú║Scoliosis - ╕▒▒╛/MIF/Supplementary Material 3 MIF 2.jpeg]

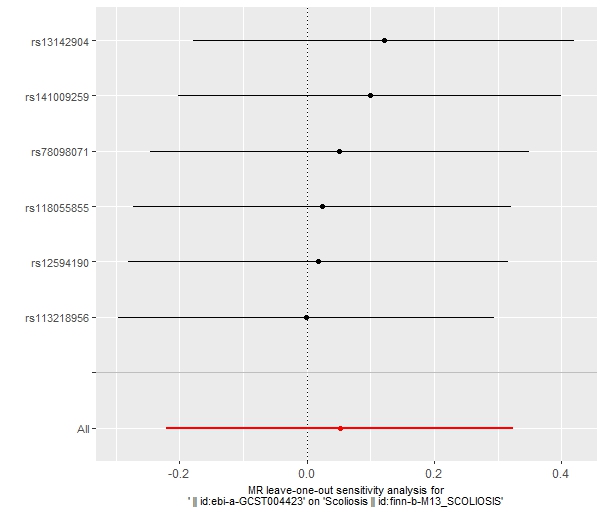

Supplement: Supplementary file 3 — Supplementary Material 3. [file JSP2-7-e70019-s004.zip › Supplementary Material 3/Exposureú║inflammatory cytokinesú1⁄4Outcomeú║Scoliosis - ╕▒▒╛/MIF/Supplementary Material 3 MIF 3.jpeg]

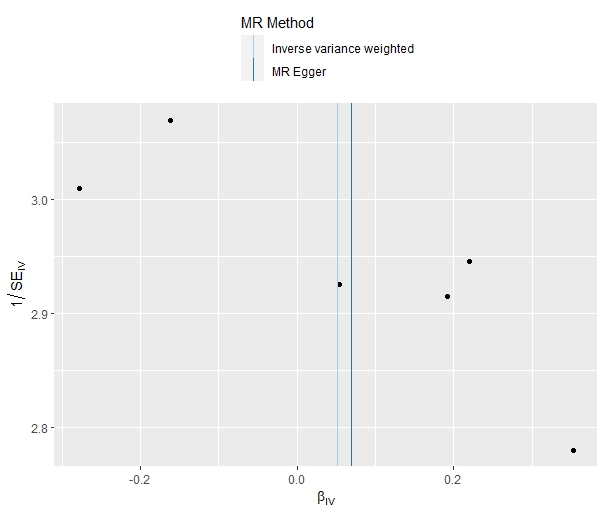

Supplement: Supplementary file 3 — Supplementary Material 3. [file JSP2-7-e70019-s004.zip › Supplementary Material 3/Exposureú║inflammatory cytokinesú1⁄4Outcomeú║Scoliosis - ╕▒▒╛/MIF/Supplementary Material 3 MIF 4.jpeg]

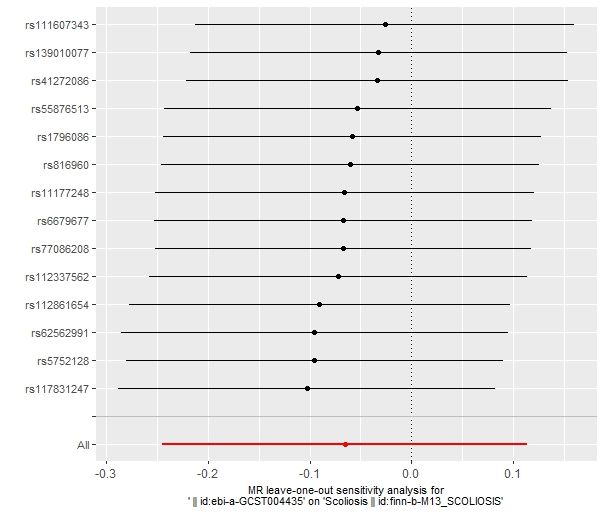

Supplement: Supplementary file 3 — Supplementary Material 3. [file JSP2-7-e70019-s004.zip › Supplementary Material 3/Exposureú║inflammatory cytokinesú1⁄4Outcomeú║Scoliosis - ╕▒▒╛/MIG/Supplementary Material 3 MIG 1.jpeg]

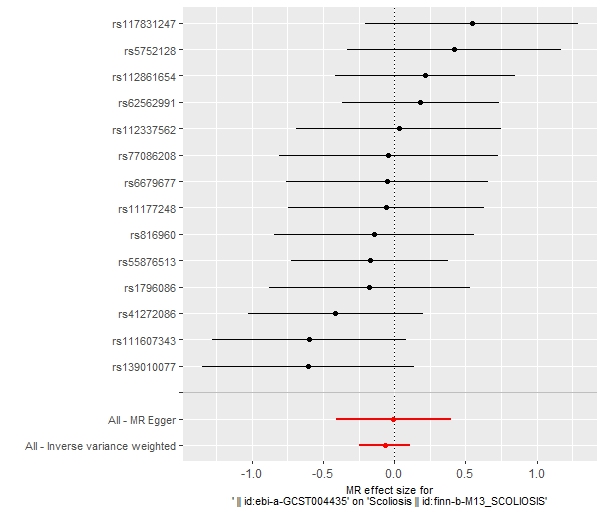

Supplement: Supplementary file 3 — Supplementary Material 3. [file JSP2-7-e70019-s004.zip › Supplementary Material 3/Exposureú║inflammatory cytokinesú1⁄4Outcomeú║Scoliosis - ╕▒▒╛/MIG/Supplementary Material 3 MIG 2.jpeg]

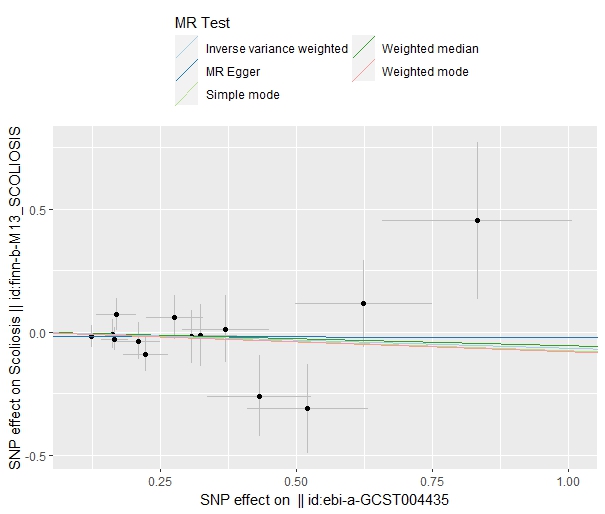

Supplement: Supplementary file 3 — Supplementary Material 3. [file JSP2-7-e70019-s004.zip › Supplementary Material 3/Exposureú║inflammatory cytokinesú1⁄4Outcomeú║Scoliosis - ╕▒▒╛/MIG/Supplementary Material 3 MIG 3.jpeg]

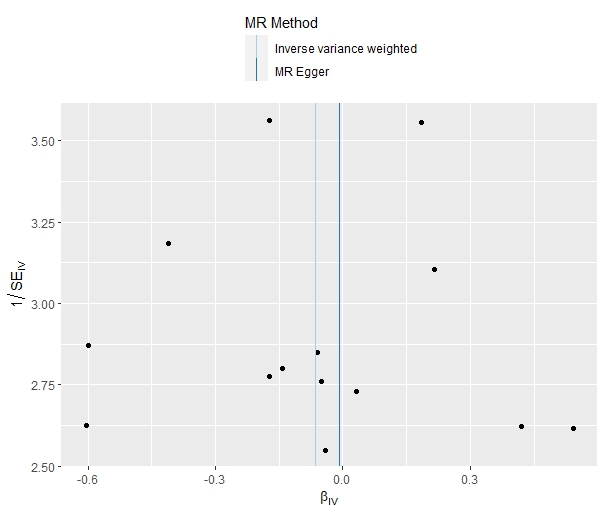

Supplement: Supplementary file 3 — Supplementary Material 3. [file JSP2-7-e70019-s004.zip › Supplementary Material 3/Exposureú║inflammatory cytokinesú1⁄4Outcomeú║Scoliosis - ╕▒▒╛/MIG/Supplementary Material 3 MIG 4.jpeg]

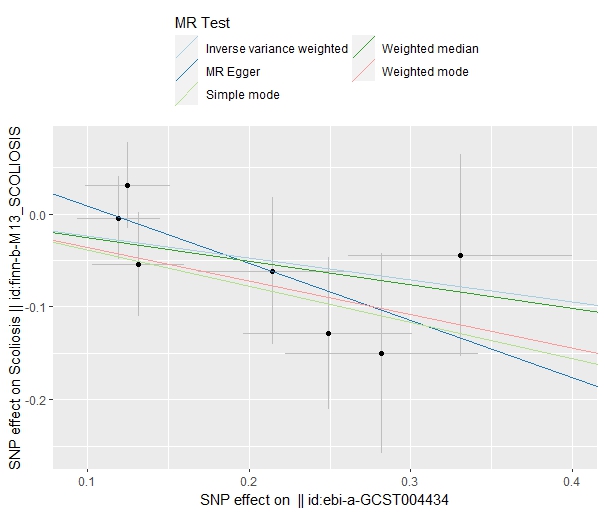

Supplement: Supplementary file 3 — Supplementary Material 3. [file JSP2-7-e70019-s004.zip › Supplementary Material 3/Exposureú║inflammatory cytokinesú1⁄4Outcomeú║Scoliosis - ╕▒▒╛/MIP1 A/Supplementary Material 3 MIP1 A 1.jpeg]

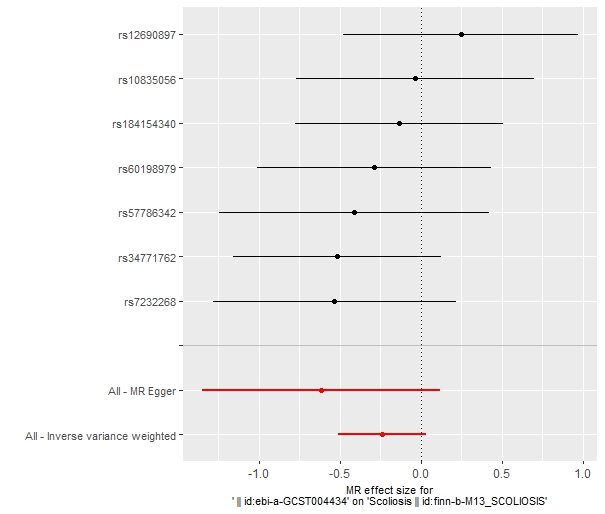

Supplement: Supplementary file 3 — Supplementary Material 3. [file JSP2-7-e70019-s004.zip › Supplementary Material 3/Exposureú║inflammatory cytokinesú1⁄4Outcomeú║Scoliosis - ╕▒▒╛/MIP1 A/Supplementary Material 3 MIP1 A 2.jpeg]

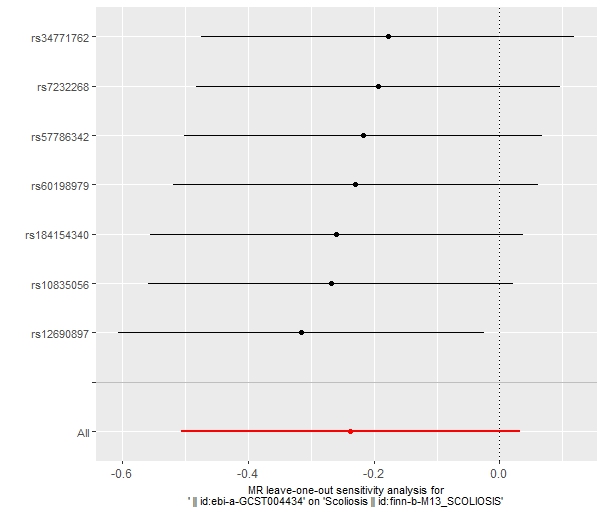

Supplement: Supplementary file 3 — Supplementary Material 3. [file JSP2-7-e70019-s004.zip › Supplementary Material 3/Exposureú║inflammatory cytokinesú1⁄4Outcomeú║Scoliosis - ╕▒▒╛/MIP1 A/Supplementary Material 3 MIP1 A 3.jpeg]

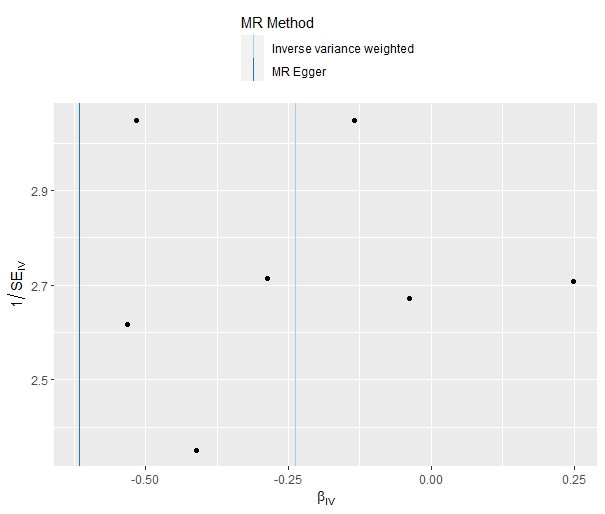

Supplement: Supplementary file 3 — Supplementary Material 3. [file JSP2-7-e70019-s004.zip › Supplementary Material 3/Exposureú║inflammatory cytokinesú1⁄4Outcomeú║Scoliosis - ╕▒▒╛/MIP1 A/Supplementary Material 3 MIP1 A 4.jpeg]

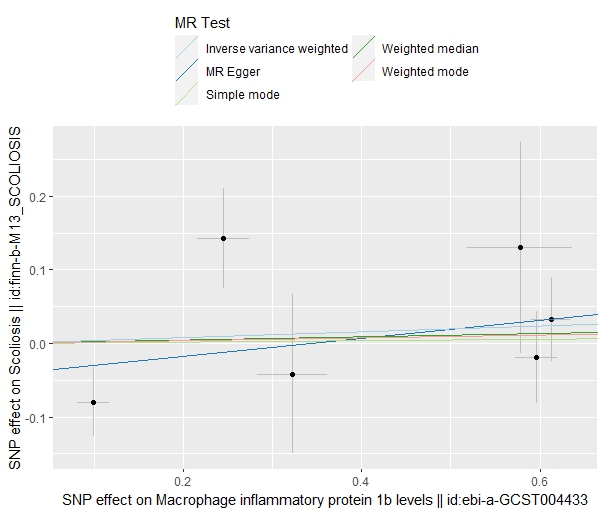

Supplement: Supplementary file 3 — Supplementary Material 3. [file JSP2-7-e70019-s004.zip › Supplementary Material 3/Exposureú║inflammatory cytokinesú1⁄4Outcomeú║Scoliosis - ╕▒▒╛/MIP1 B/Supplementary Material 3 MIP1 B 1.jpeg]

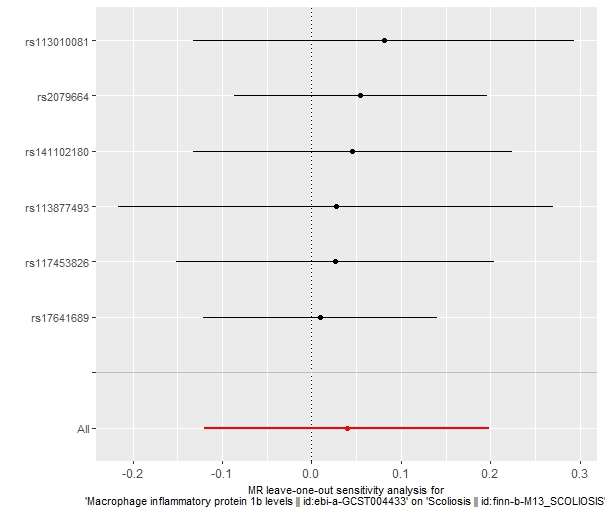

Supplement: Supplementary file 3 — Supplementary Material 3. [file JSP2-7-e70019-s004.zip › Supplementary Material 3/Exposureú║inflammatory cytokinesú1⁄4Outcomeú║Scoliosis - ╕▒▒╛/MIP1 B/Supplementary Material 3 MIP1 B 2.jpeg]

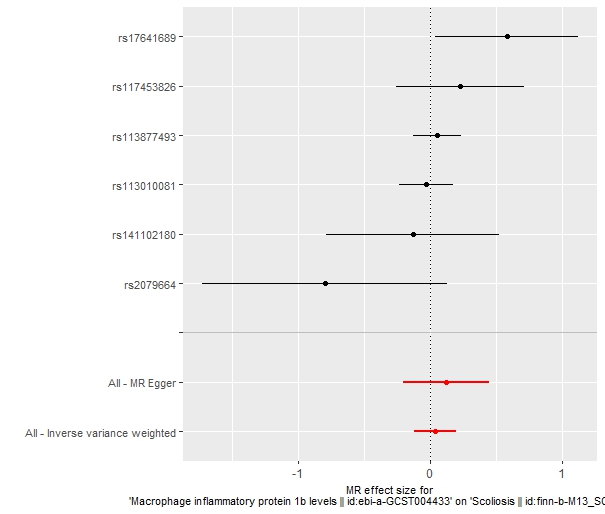

Supplement: Supplementary file 3 — Supplementary Material 3. [file JSP2-7-e70019-s004.zip › Supplementary Material 3/Exposureú║inflammatory cytokinesú1⁄4Outcomeú║Scoliosis - ╕▒▒╛/MIP1 B/Supplementary Material 3 MIP1 B 3.jpeg]

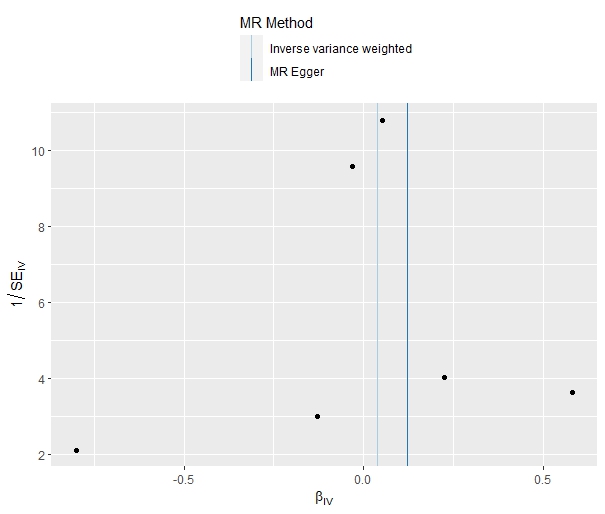

Supplement: Supplementary file 3 — Supplementary Material 3. [file JSP2-7-e70019-s004.zip › Supplementary Material 3/Exposureú║inflammatory cytokinesú1⁄4Outcomeú║Scoliosis - ╕▒▒╛/MIP1 B/Supplementary Material 3 MIP1 B 4.jpeg]

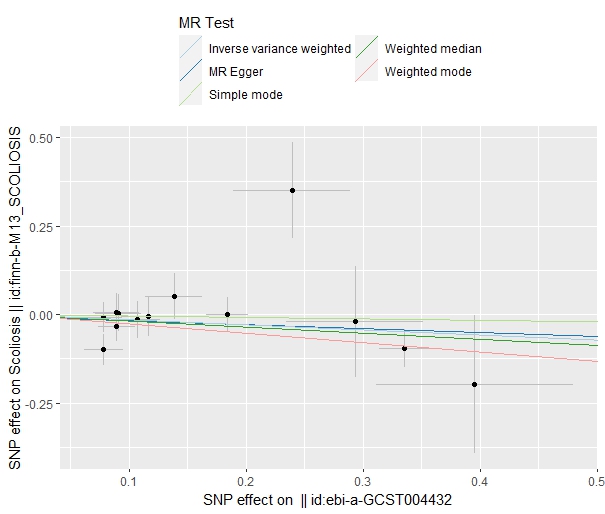

Supplement: Supplementary file 3 — Supplementary Material 3. [file JSP2-7-e70019-s004.zip › Supplementary Material 3/Exposureú║inflammatory cytokinesú1⁄4Outcomeú║Scoliosis - ╕▒▒╛/PDGFbb/Supplementary Material 3 PDGFbb 1.jpeg]

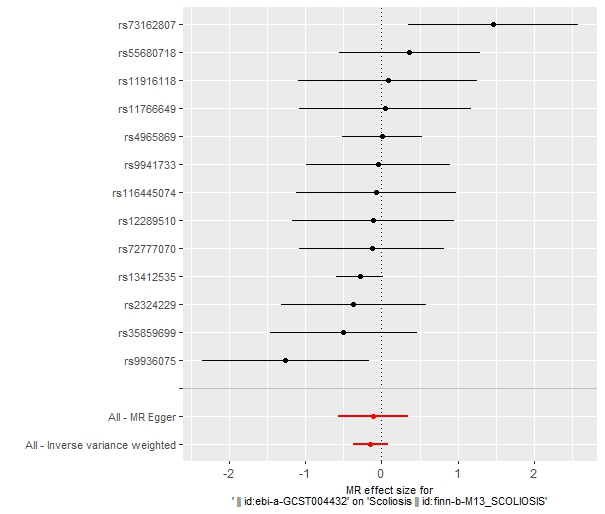

Supplement: Supplementary file 3 — Supplementary Material 3. [file JSP2-7-e70019-s004.zip › Supplementary Material 3/Exposureú║inflammatory cytokinesú1⁄4Outcomeú║Scoliosis - ╕▒▒╛/PDGFbb/Supplementary Material 3 PDGFbb 2.jpeg]

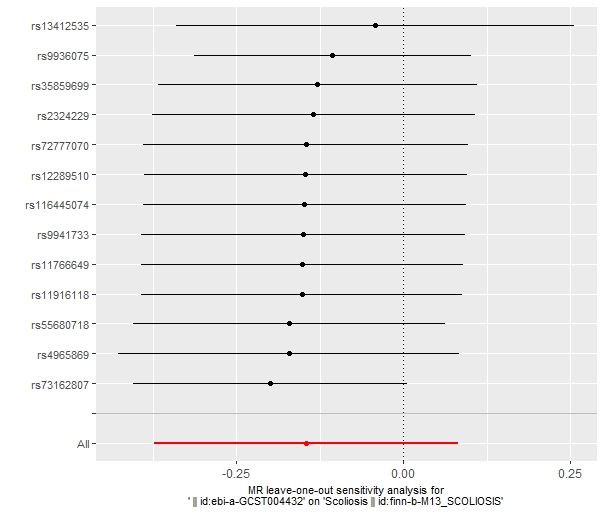

Supplement: Supplementary file 3 — Supplementary Material 3. [file JSP2-7-e70019-s004.zip › Supplementary Material 3/Exposureú║inflammatory cytokinesú1⁄4Outcomeú║Scoliosis - ╕▒▒╛/PDGFbb/Supplementary Material 3 PDGFbb 3.jpeg]

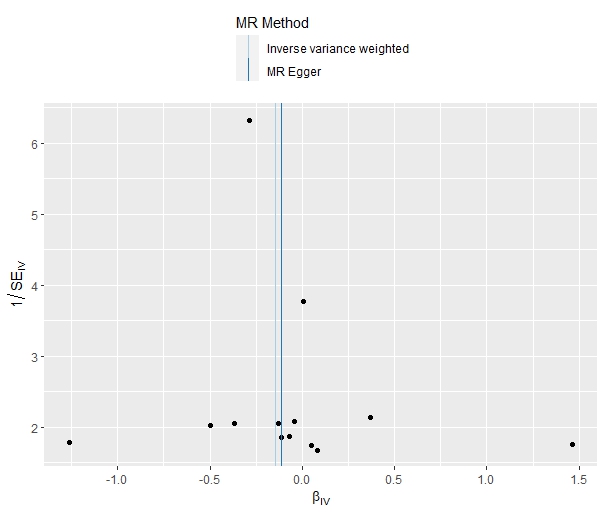

Supplement: Supplementary file 3 — Supplementary Material 3. [file JSP2-7-e70019-s004.zip › Supplementary Material 3/Exposureú║inflammatory cytokinesú1⁄4Outcomeú║Scoliosis - ╕▒▒╛/PDGFbb/Supplementary Material 3 PDGFbb 4.jpeg]

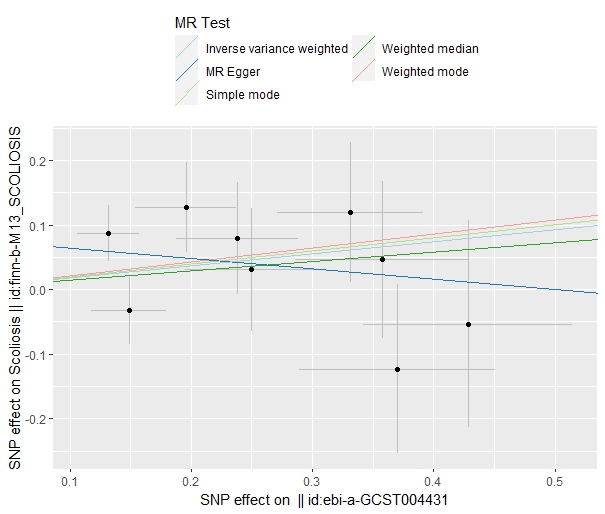

Supplement: Supplementary file 3 — Supplementary Material 3. [file JSP2-7-e70019-s004.zip › Supplementary Material 3/Exposureú║inflammatory cytokinesú1⁄4Outcomeú║Scoliosis - ╕▒▒╛/RANTES/Supplementary Material 3 RANTES 1.jpeg]

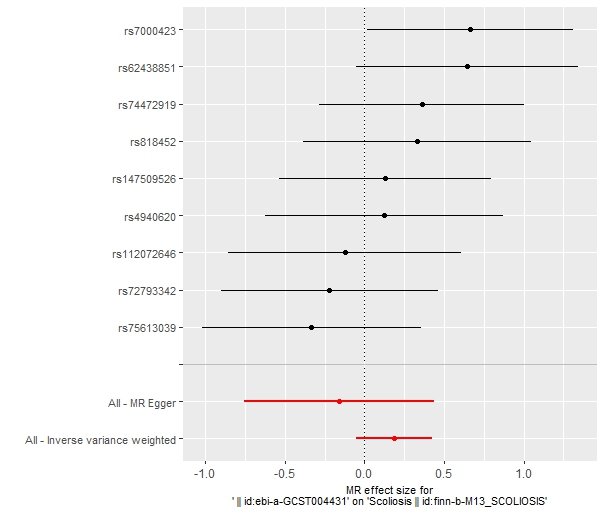

Supplement: Supplementary file 3 — Supplementary Material 3. [file JSP2-7-e70019-s004.zip › Supplementary Material 3/Exposureú║inflammatory cytokinesú1⁄4Outcomeú║Scoliosis - ╕▒▒╛/RANTES/Supplementary Material 3 RANTES 2.jpeg]

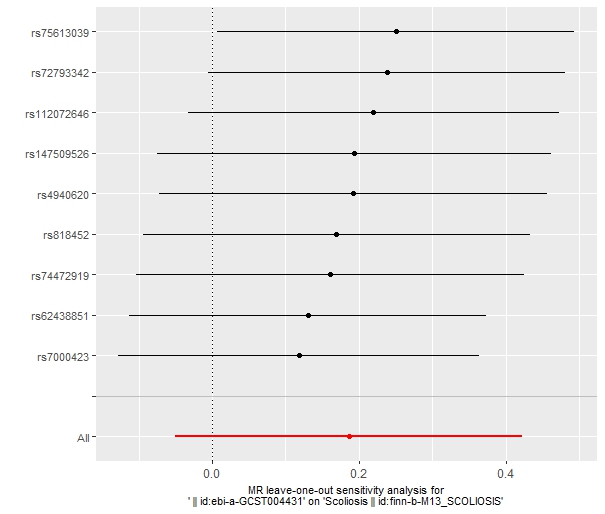

Supplement: Supplementary file 3 — Supplementary Material 3. [file JSP2-7-e70019-s004.zip › Supplementary Material 3/Exposureú║inflammatory cytokinesú1⁄4Outcomeú║Scoliosis - ╕▒▒╛/RANTES/Supplementary Material 3 RANTES 3.jpeg]

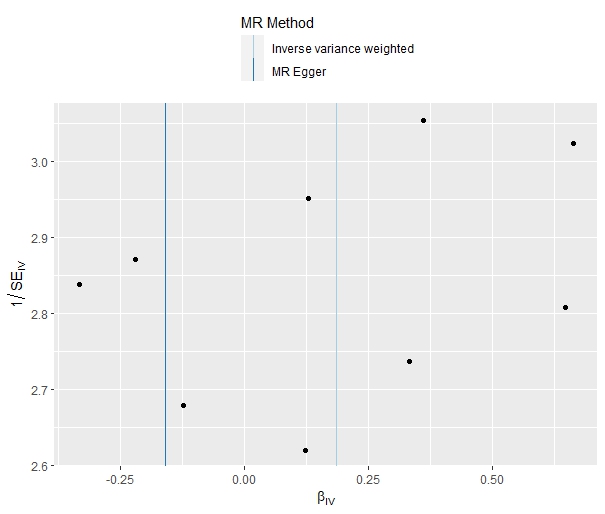

Supplement: Supplementary file 3 — Supplementary Material 3. [file JSP2-7-e70019-s004.zip › Supplementary Material 3/Exposureú║inflammatory cytokinesú1⁄4Outcomeú║Scoliosis - ╕▒▒╛/RANTES/Supplementary Material 3 RANTES 4.jpeg]

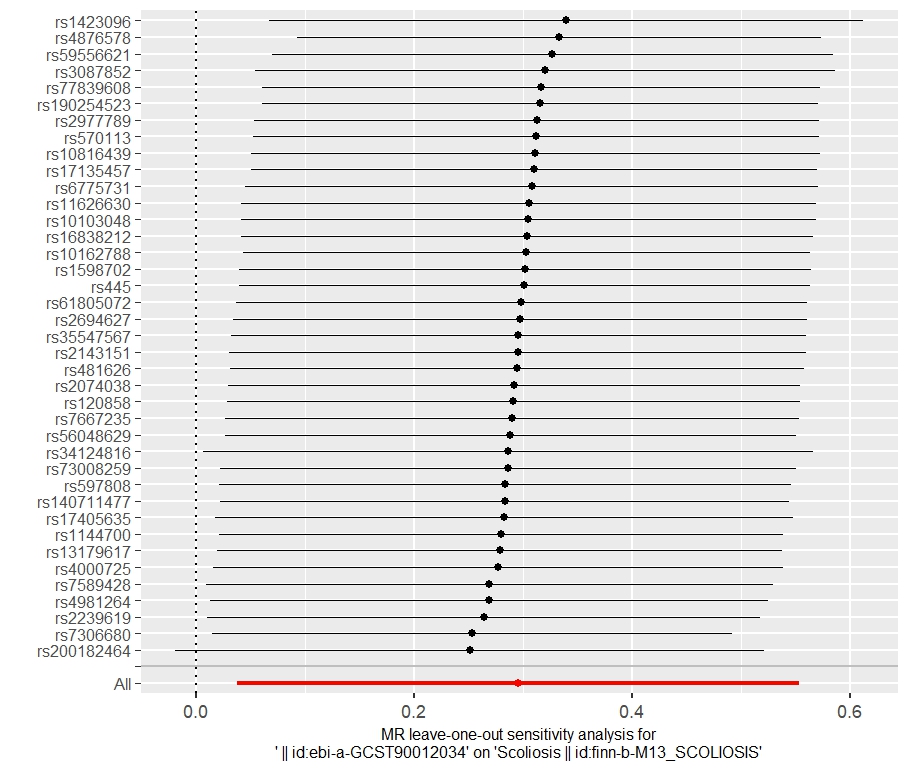

Supplement: Supplementary file 3 — Supplementary Material 3. [file JSP2-7-e70019-s004.zip › Supplementary Material 3/Exposureú║inflammatory cytokinesú1⁄4Outcomeú║Scoliosis - ╕▒▒╛/RETN/Supplementary Material 3 RETN 1.jpeg]

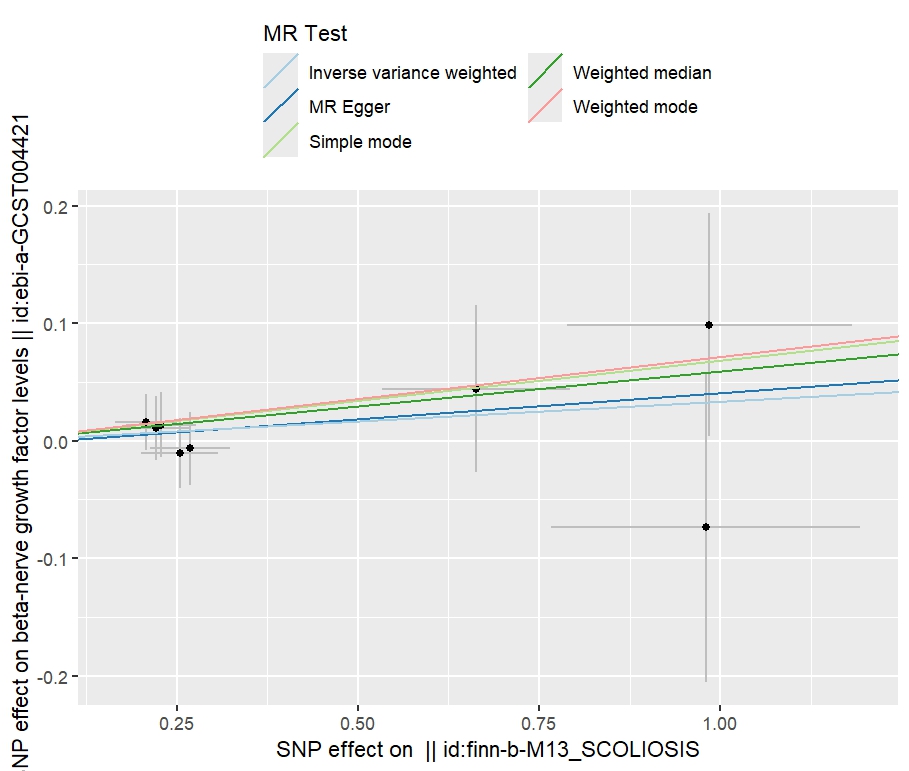

Supplement: Supplementary file 4 — Supplementary Material 4. [file JSP2-7-e70019-s002.zip › Supplementary Material 4/Exposureú║Scoliosisú1⁄4Outcomeú║inflammatory cytokines/B NGF/Supplementary Material 4 B NGF 1.jpeg]

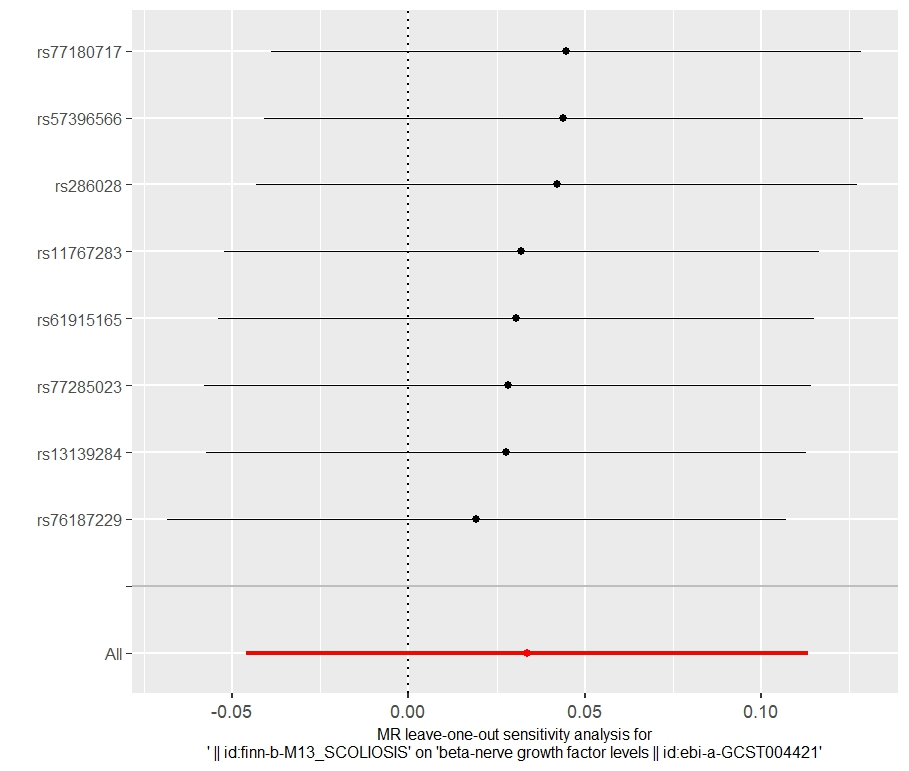

Supplement: Supplementary file 4 — Supplementary Material 4. [file JSP2-7-e70019-s002.zip › Supplementary Material 4/Exposureú║Scoliosisú1⁄4Outcomeú║inflammatory cytokines/B NGF/Supplementary Material 4 B NGF 2.jpeg]

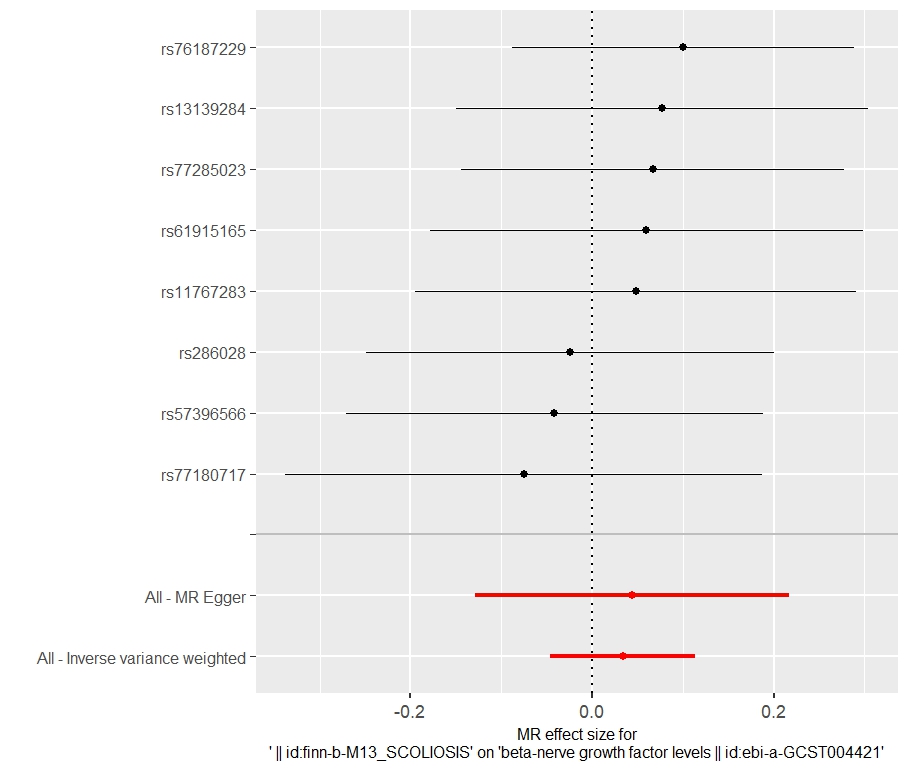

Supplement: Supplementary file 4 — Supplementary Material 4. [file JSP2-7-e70019-s002.zip › Supplementary Material 4/Exposureú║Scoliosisú1⁄4Outcomeú║inflammatory cytokines/B NGF/Supplementary Material 4 B NGF 3.jpeg]

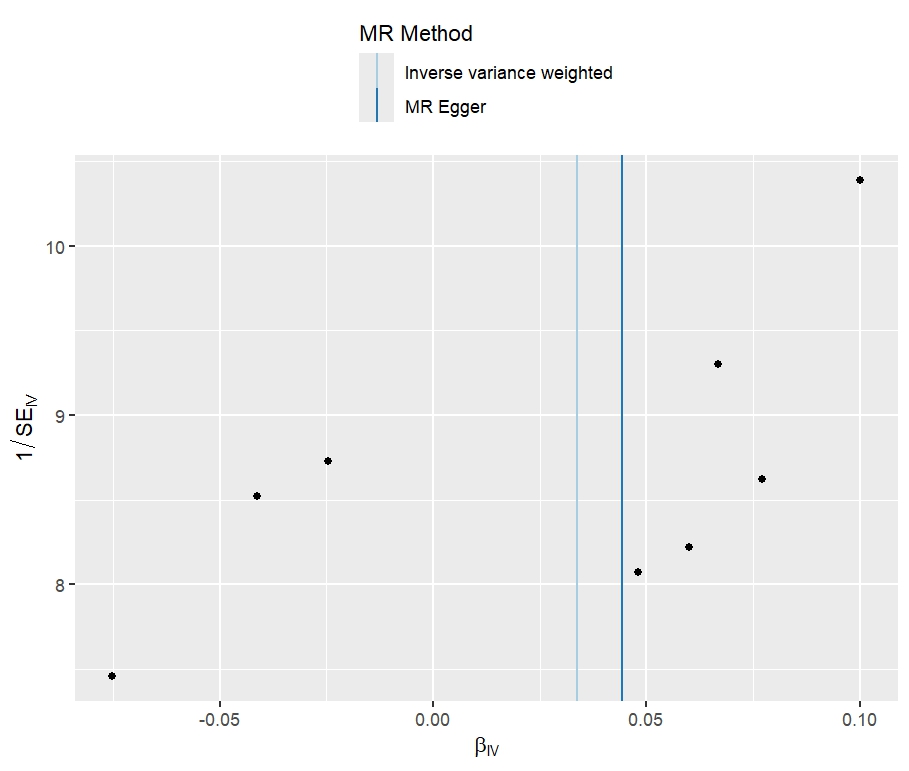

Supplement: Supplementary file 4 — Supplementary Material 4. [file JSP2-7-e70019-s002.zip › Supplementary Material 4/Exposureú║Scoliosisú1⁄4Outcomeú║inflammatory cytokines/B NGF/Supplementary Material 4 B NGF 4.jpeg]

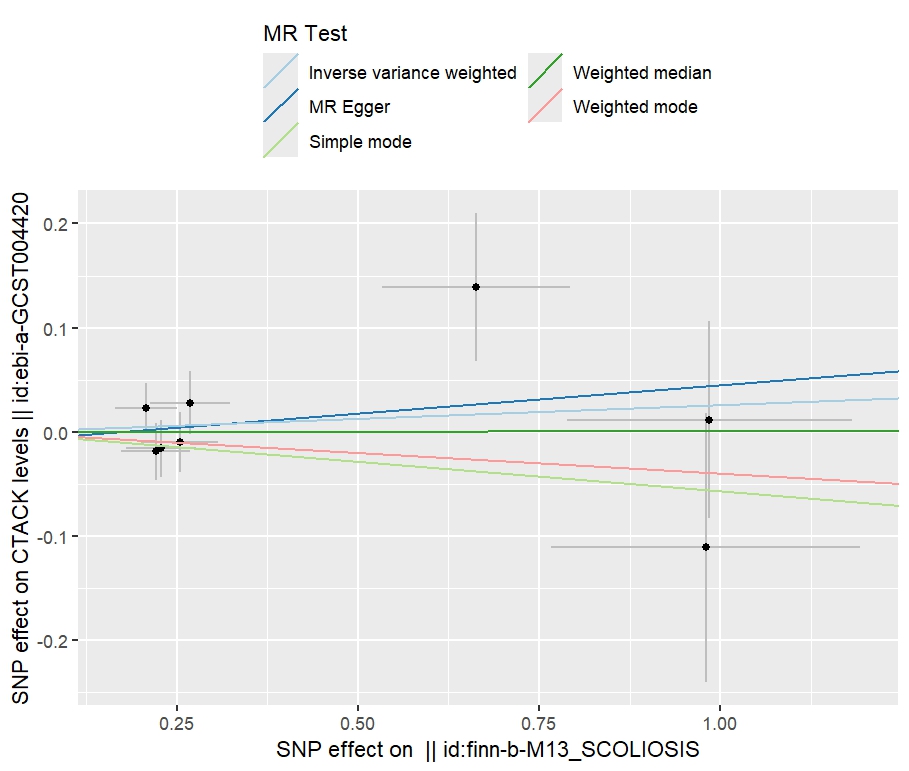

Supplement: Supplementary file 4 — Supplementary Material 4. [file JSP2-7-e70019-s002.zip › Supplementary Material 4/Exposureú║Scoliosisú1⁄4Outcomeú║inflammatory cytokines/CTACK/Supplementary Material 4 CTACK 1.jpeg]

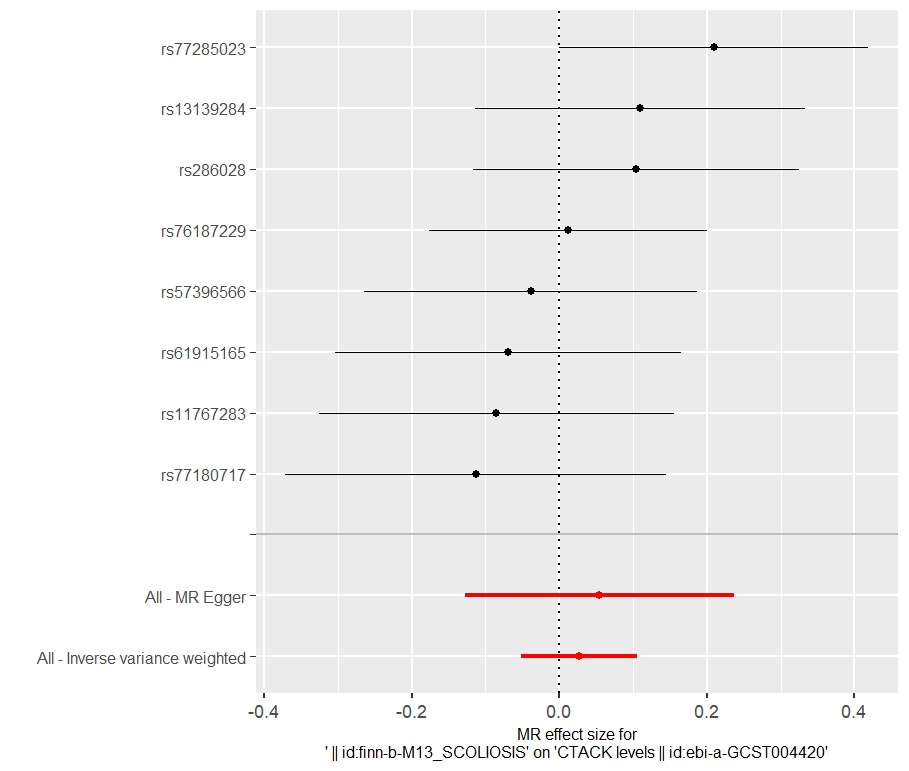

Supplement: Supplementary file 4 — Supplementary Material 4. [file JSP2-7-e70019-s002.zip › Supplementary Material 4/Exposureú║Scoliosisú1⁄4Outcomeú║inflammatory cytokines/CTACK/Supplementary Material 4 CTACK 2.jpeg]

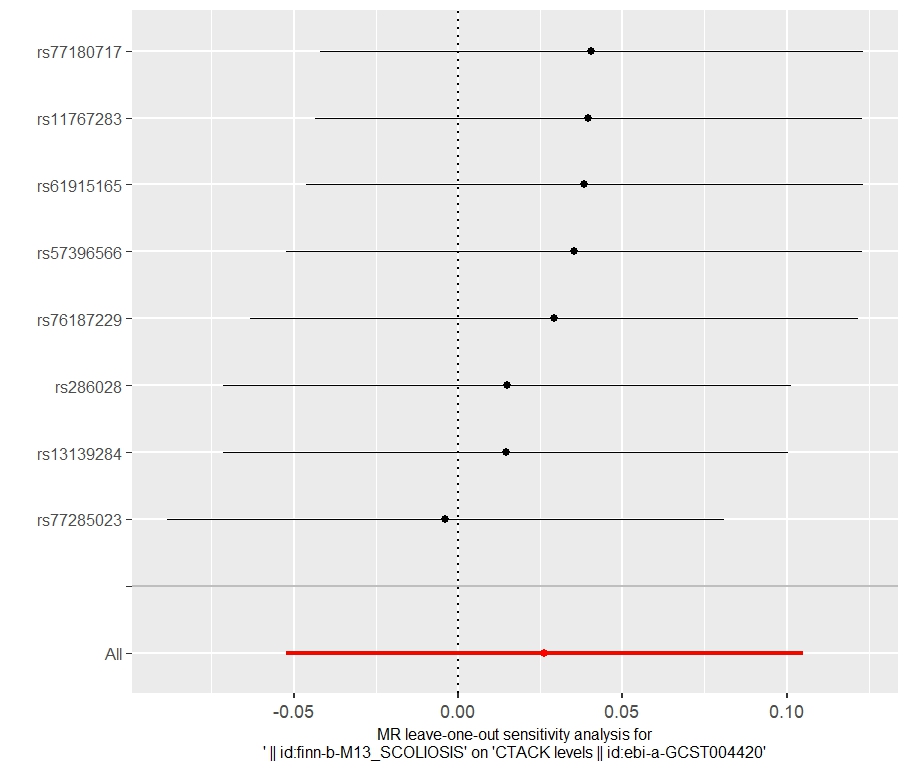

Supplement: Supplementary file 4 — Supplementary Material 4. [file JSP2-7-e70019-s002.zip › Supplementary Material 4/Exposureú║Scoliosisú1⁄4Outcomeú║inflammatory cytokines/CTACK/Supplementary Material 4 CTACK 3.jpeg]

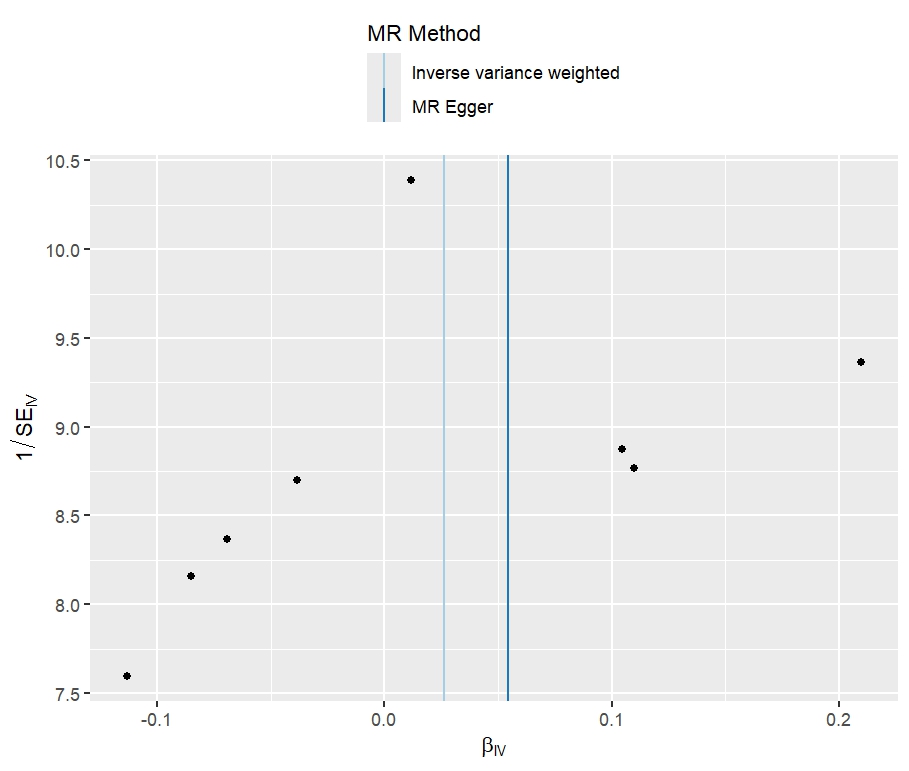

Supplement: Supplementary file 4 — Supplementary Material 4. [file JSP2-7-e70019-s002.zip › Supplementary Material 4/Exposureú║Scoliosisú1⁄4Outcomeú║inflammatory cytokines/CTACK/Supplementary Material 4 CTACK 4.jpeg]

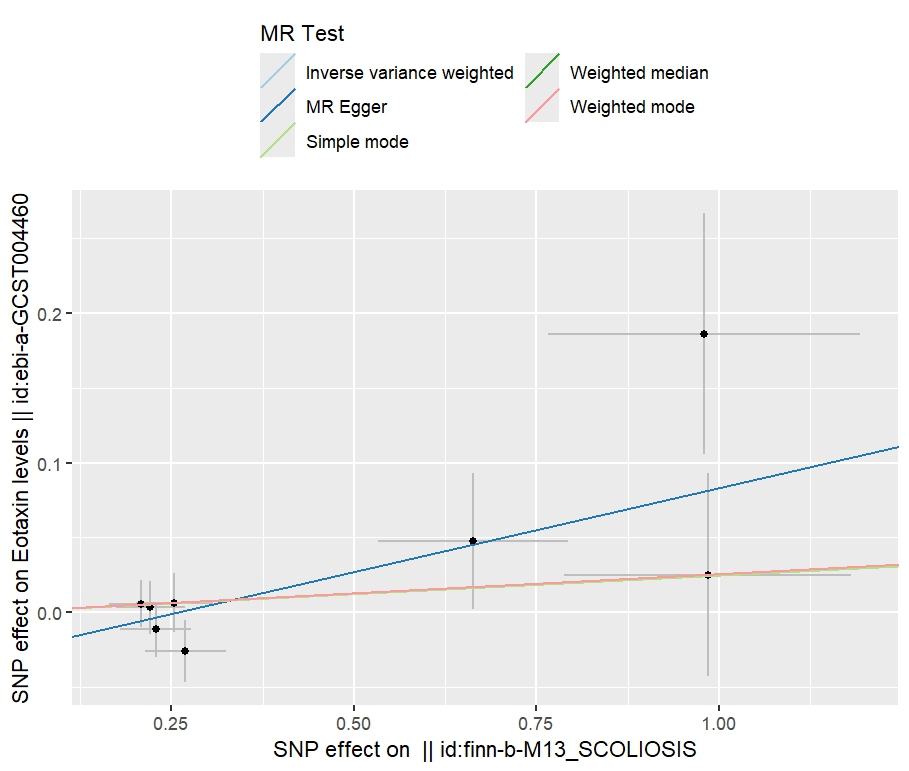

Supplement: Supplementary file 4 — Supplementary Material 4. [file JSP2-7-e70019-s002.zip › Supplementary Material 4/Exposureú║Scoliosisú1⁄4Outcomeú║inflammatory cytokines/Eotaxin/Supplementary Material 4 Eotaxin 1.jpeg]

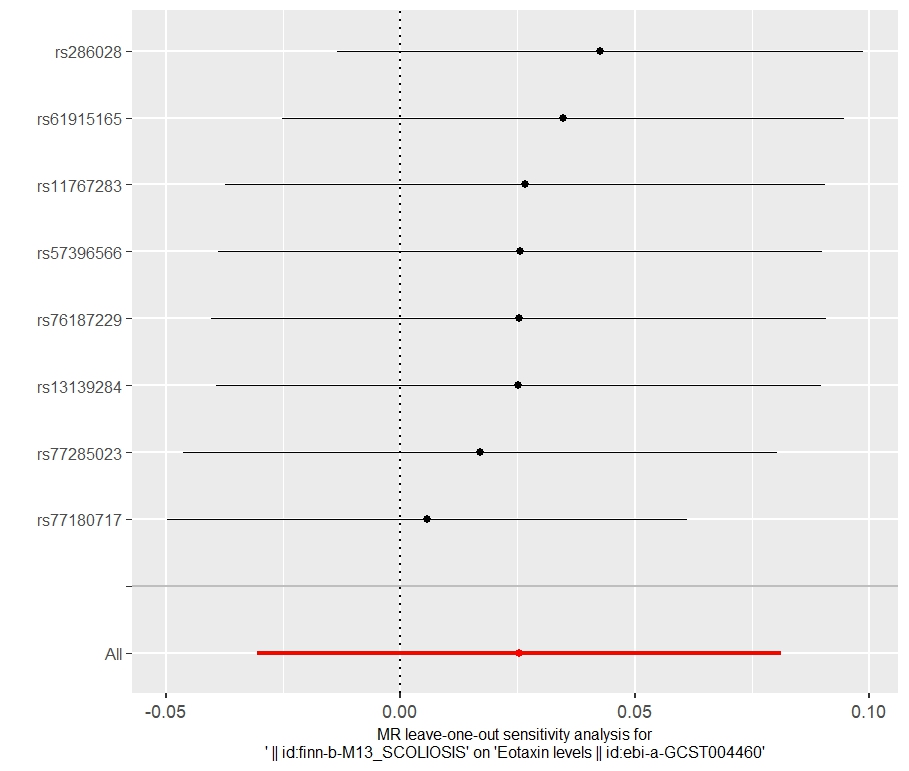

Supplement: Supplementary file 4 — Supplementary Material 4. [file JSP2-7-e70019-s002.zip › Supplementary Material 4/Exposureú║Scoliosisú1⁄4Outcomeú║inflammatory cytokines/Eotaxin/Supplementary Material 4 Eotaxin 2.jpeg]

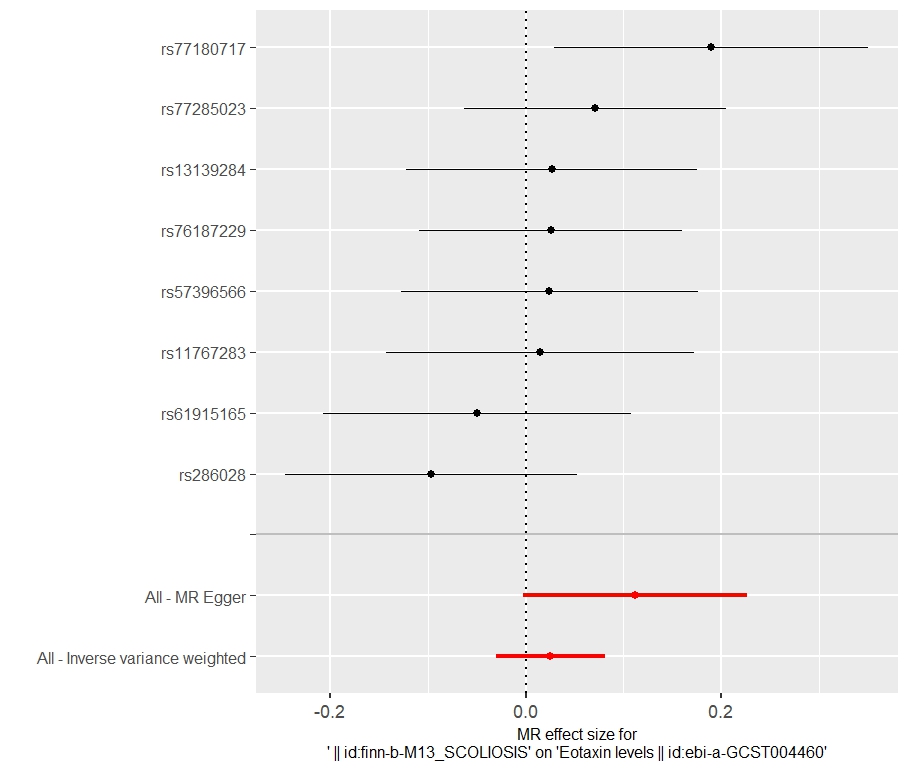

Supplement: Supplementary file 4 — Supplementary Material 4. [file JSP2-7-e70019-s002.zip › Supplementary Material 4/Exposureú║Scoliosisú1⁄4Outcomeú║inflammatory cytokines/Eotaxin/Supplementary Material 4 Eotaxin 3.jpeg]

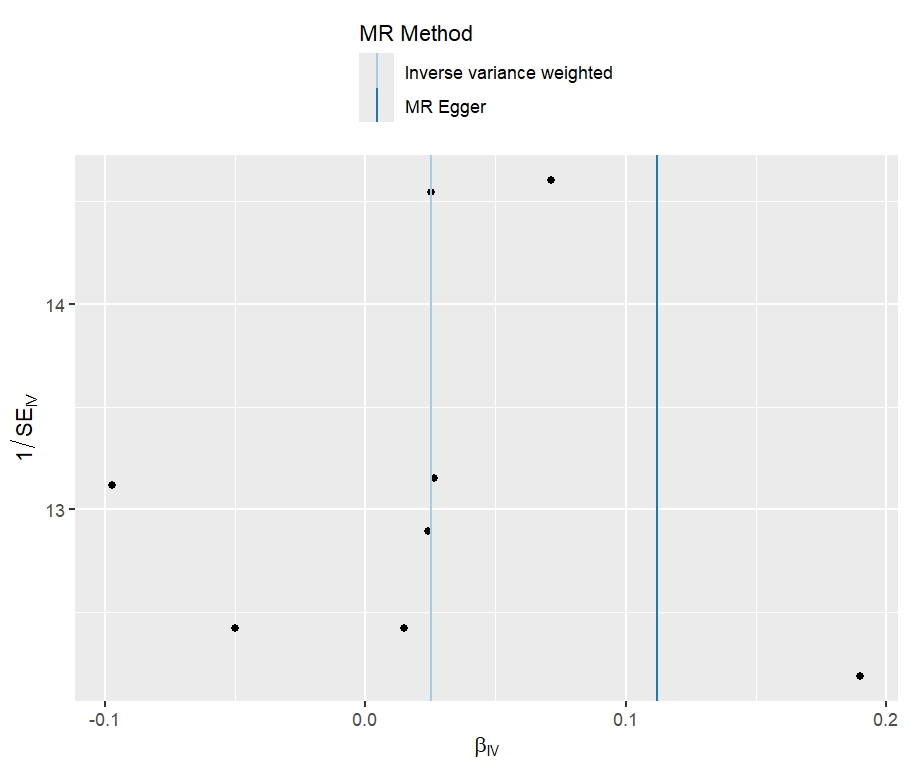

Supplement: Supplementary file 4 — Supplementary Material 4. [file JSP2-7-e70019-s002.zip › Supplementary Material 4/Exposureú║Scoliosisú1⁄4Outcomeú║inflammatory cytokines/Eotaxin/Supplementary Material 4 Eotaxin 4.jpeg]

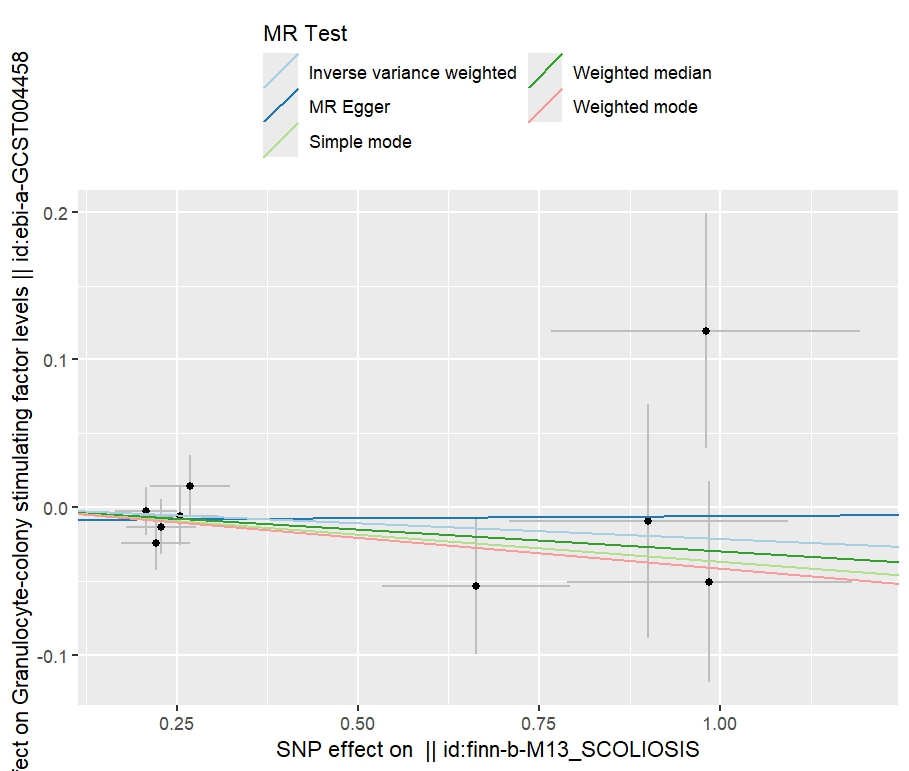

Supplement: Supplementary file 4 — Supplementary Material 4. [file JSP2-7-e70019-s002.zip › Supplementary Material 4/Exposureú║Scoliosisú1⁄4Outcomeú║inflammatory cytokines/GCSF/Supplementary Material 4 GCSF 1.jpeg]

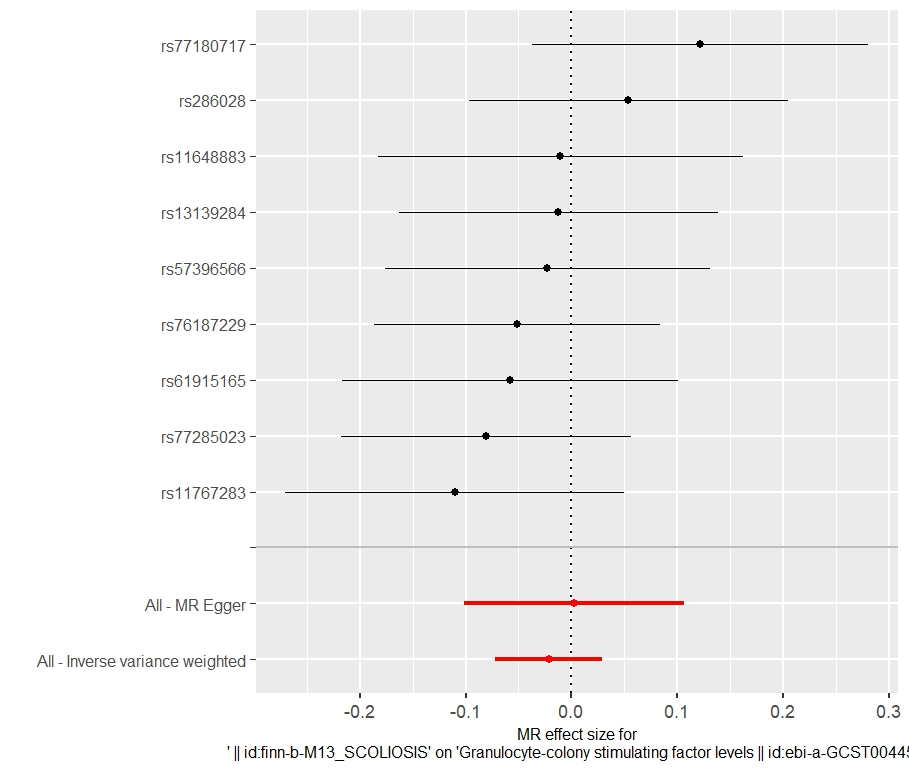

Supplement: Supplementary file 4 — Supplementary Material 4. [file JSP2-7-e70019-s002.zip › Supplementary Material 4/Exposureú║Scoliosisú1⁄4Outcomeú║inflammatory cytokines/GCSF/Supplementary Material 4 GCSF 2.jpeg]

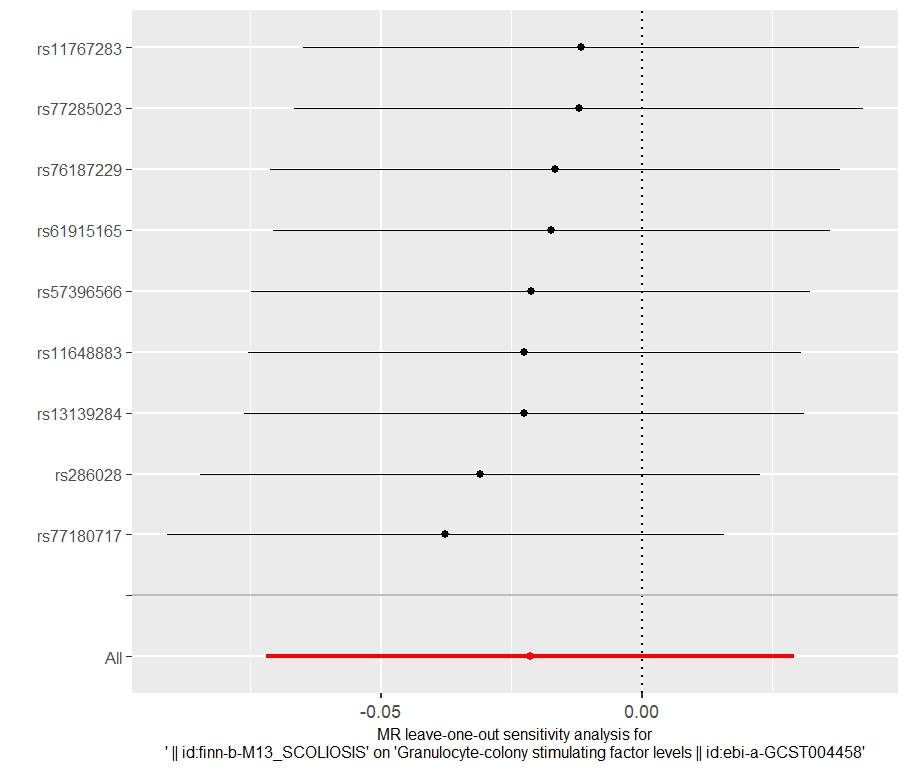

Supplement: Supplementary file 4 — Supplementary Material 4. [file JSP2-7-e70019-s002.zip › Supplementary Material 4/Exposureú║Scoliosisú1⁄4Outcomeú║inflammatory cytokines/GCSF/Supplementary Material 4 GCSF 3.jpeg]

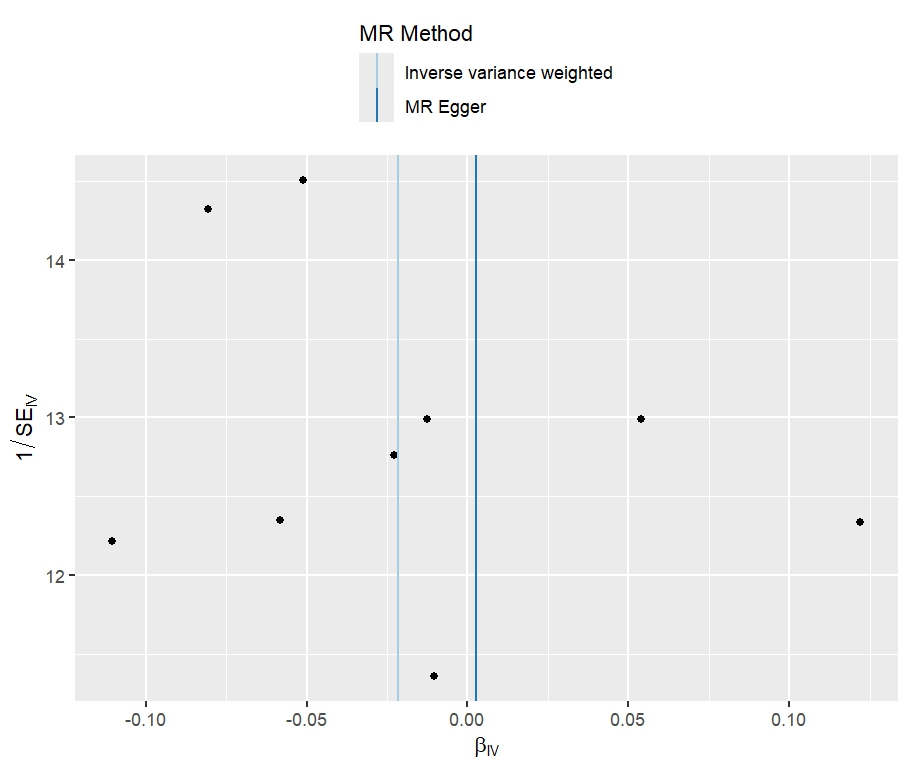

Supplement: Supplementary file 4 — Supplementary Material 4. [file JSP2-7-e70019-s002.zip › Supplementary Material 4/Exposureú║Scoliosisú1⁄4Outcomeú║inflammatory cytokines/GCSF/Supplementary Material 4 GCSF 4.jpeg]

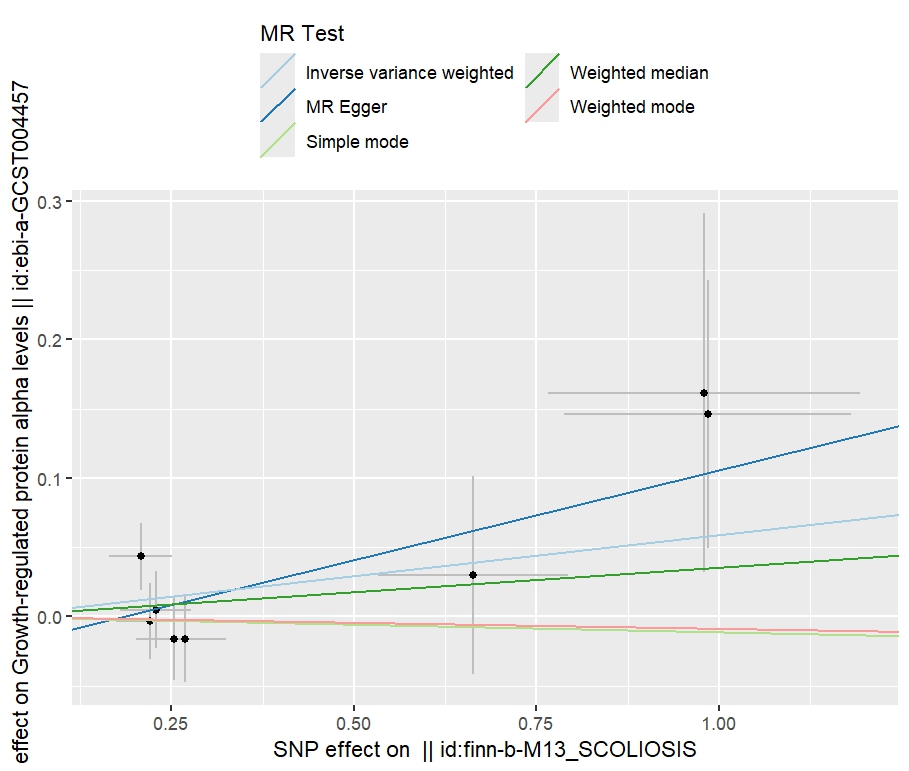

Supplement: Supplementary file 4 — Supplementary Material 4. [file JSP2-7-e70019-s002.zip › Supplementary Material 4/Exposureú║Scoliosisú1⁄4Outcomeú║inflammatory cytokines/GRPa/Supplementary Material 4 GRPa 1.jpeg]

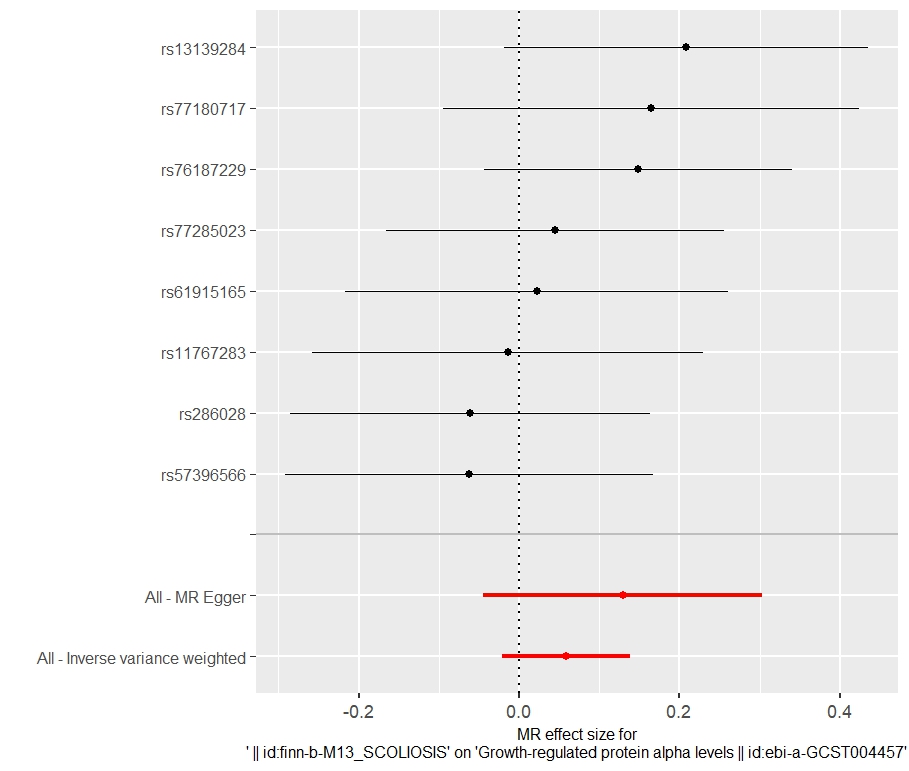

Supplement: Supplementary file 4 — Supplementary Material 4. [file JSP2-7-e70019-s002.zip › Supplementary Material 4/Exposureú║Scoliosisú1⁄4Outcomeú║inflammatory cytokines/GRPa/Supplementary Material 4 GRPa 2.jpeg]

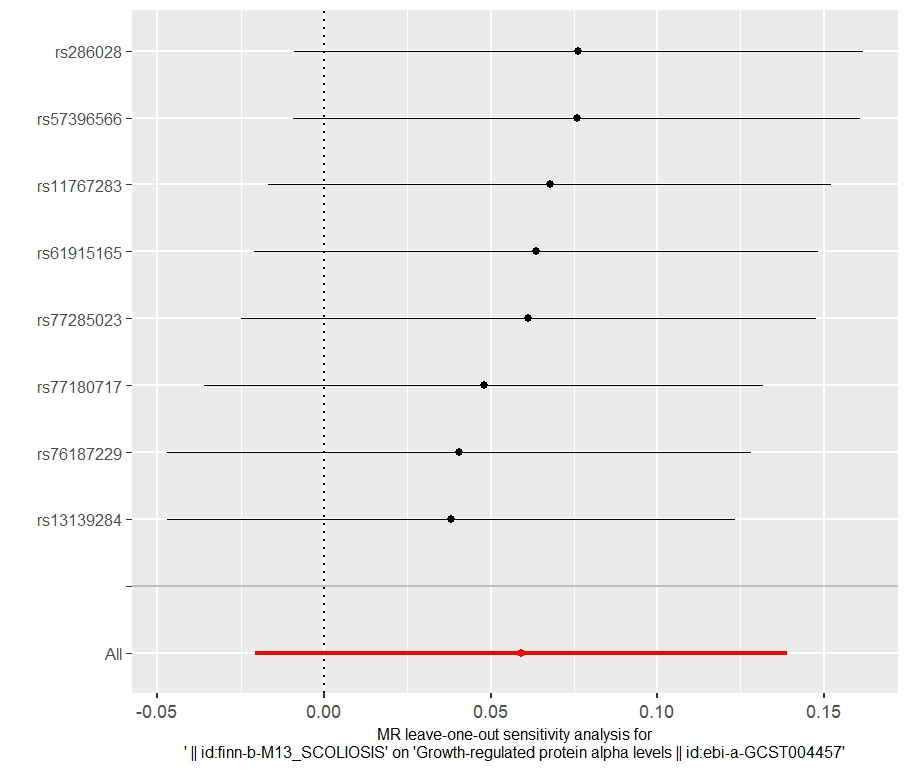

Supplement: Supplementary file 4 — Supplementary Material 4. [file JSP2-7-e70019-s002.zip › Supplementary Material 4/Exposureú║Scoliosisú1⁄4Outcomeú║inflammatory cytokines/GRPa/Supplementary Material 4 GRPa 3.jpeg]

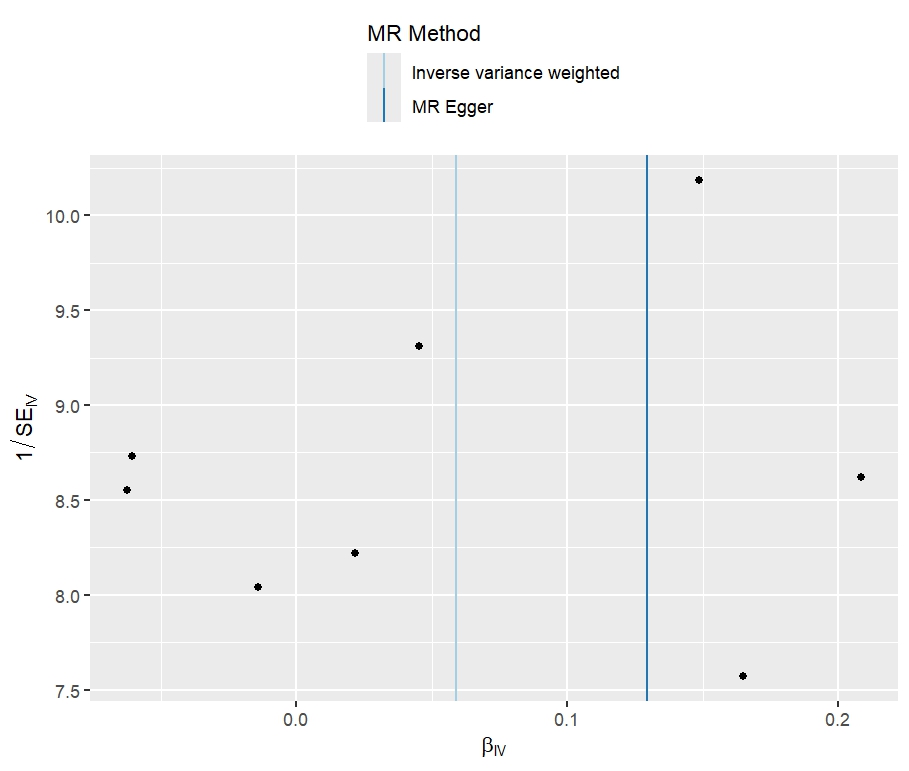

Supplement: Supplementary file 4 — Supplementary Material 4. [file JSP2-7-e70019-s002.zip › Supplementary Material 4/Exposureú║Scoliosisú1⁄4Outcomeú║inflammatory cytokines/GRPa/Supplementary Material 4 GRPa 4.jpeg]

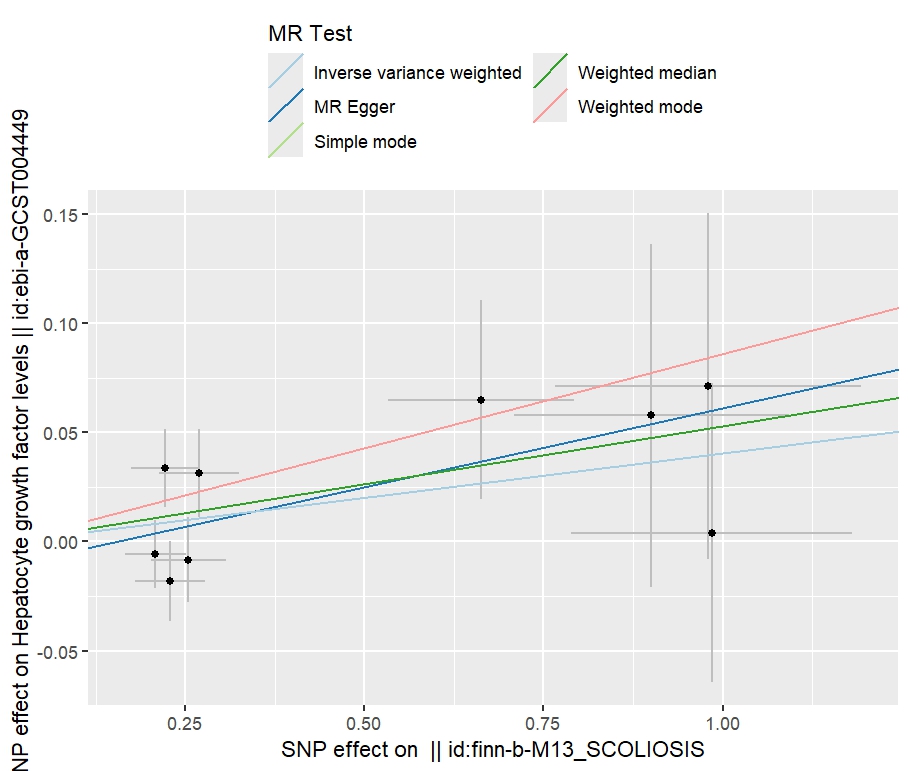

Supplement: Supplementary file 4 — Supplementary Material 4. [file JSP2-7-e70019-s002.zip › Supplementary Material 4/Exposureú║Scoliosisú1⁄4Outcomeú║inflammatory cytokines/HGF/Supplementary Material 4 HGF 1.jpeg]

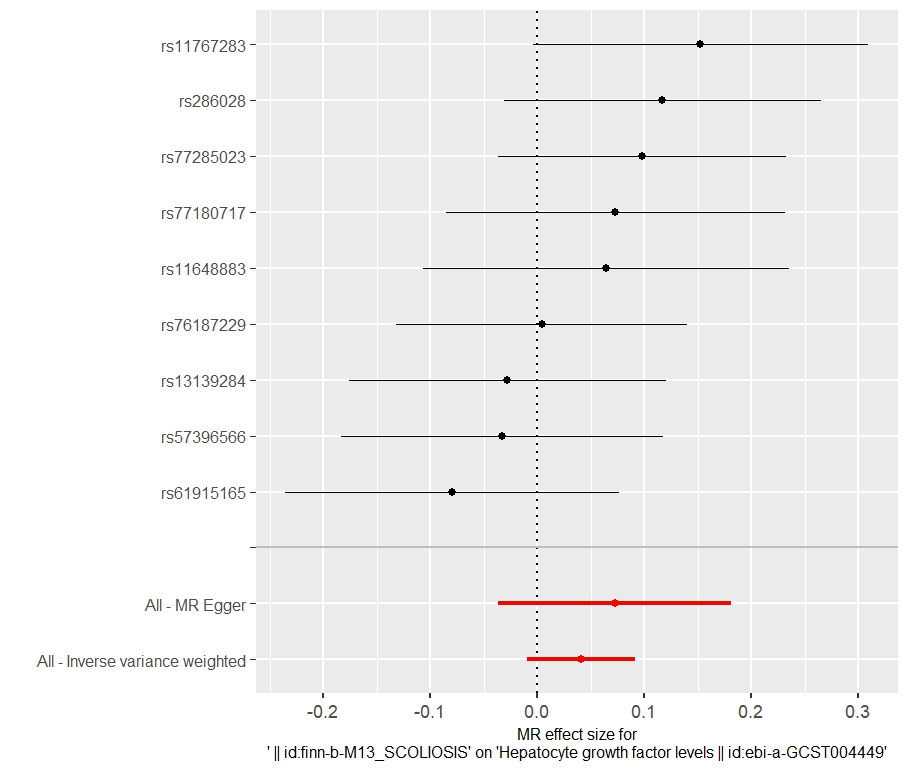

Supplement: Supplementary file 4 — Supplementary Material 4. [file JSP2-7-e70019-s002.zip › Supplementary Material 4/Exposureú║Scoliosisú1⁄4Outcomeú║inflammatory cytokines/HGF/Supplementary Material 4 HGF 2.jpeg]

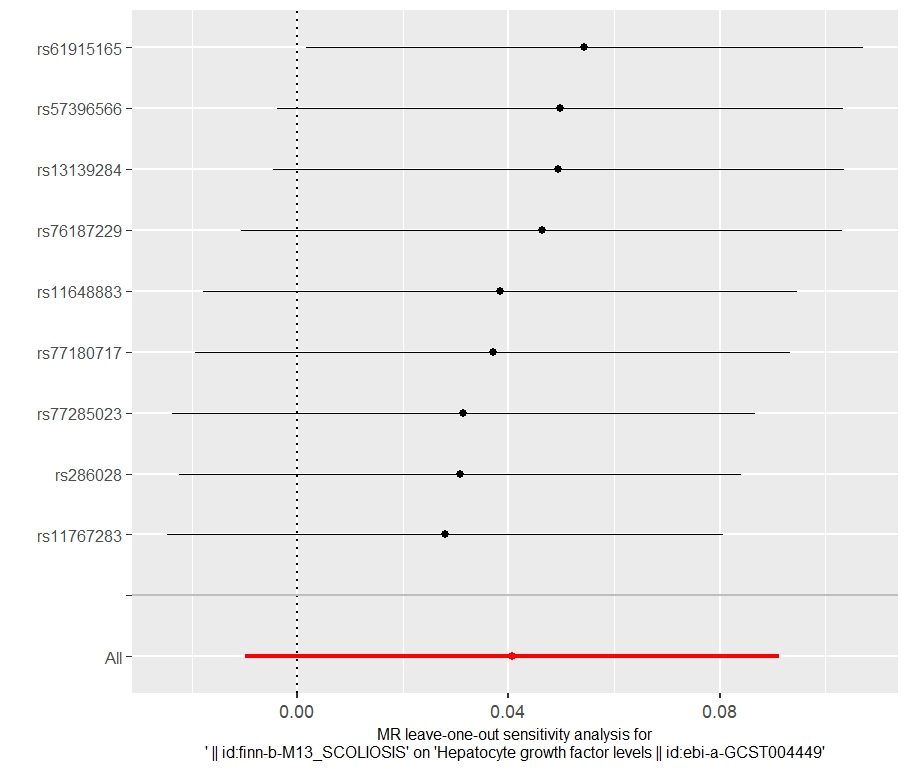

Supplement: Supplementary file 4 — Supplementary Material 4. [file JSP2-7-e70019-s002.zip › Supplementary Material 4/Exposureú║Scoliosisú1⁄4Outcomeú║inflammatory cytokines/HGF/Supplementary Material 4 HGF 3.jpeg]

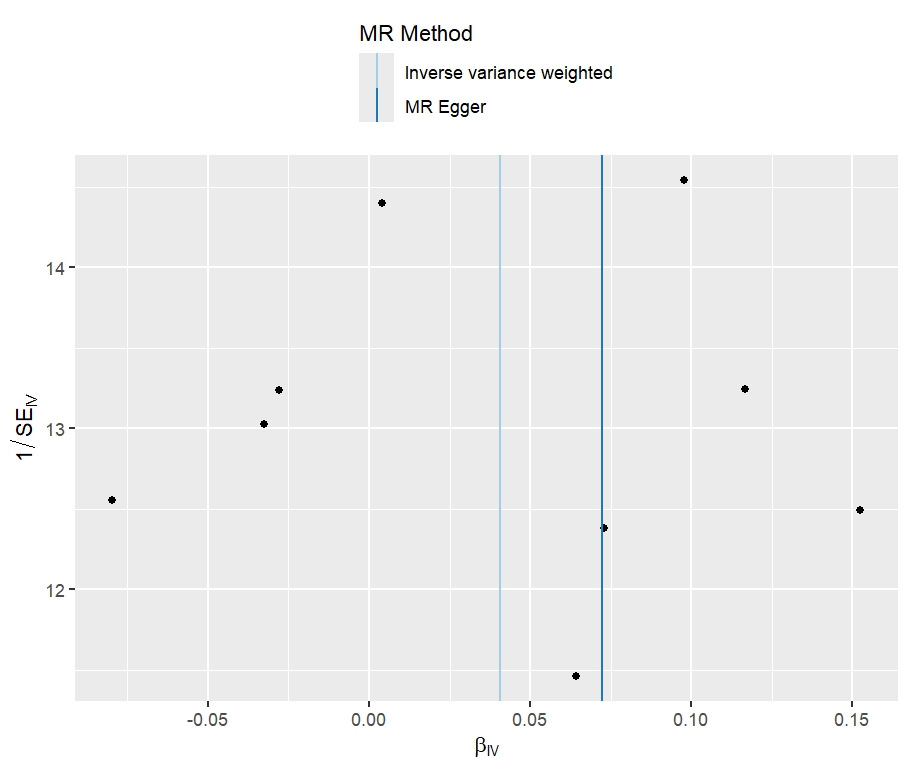

Supplement: Supplementary file 4 — Supplementary Material 4. [file JSP2-7-e70019-s002.zip › Supplementary Material 4/Exposureú║Scoliosisú1⁄4Outcomeú║inflammatory cytokines/HGF/Supplementary Material 4 HGF 4.jpeg]

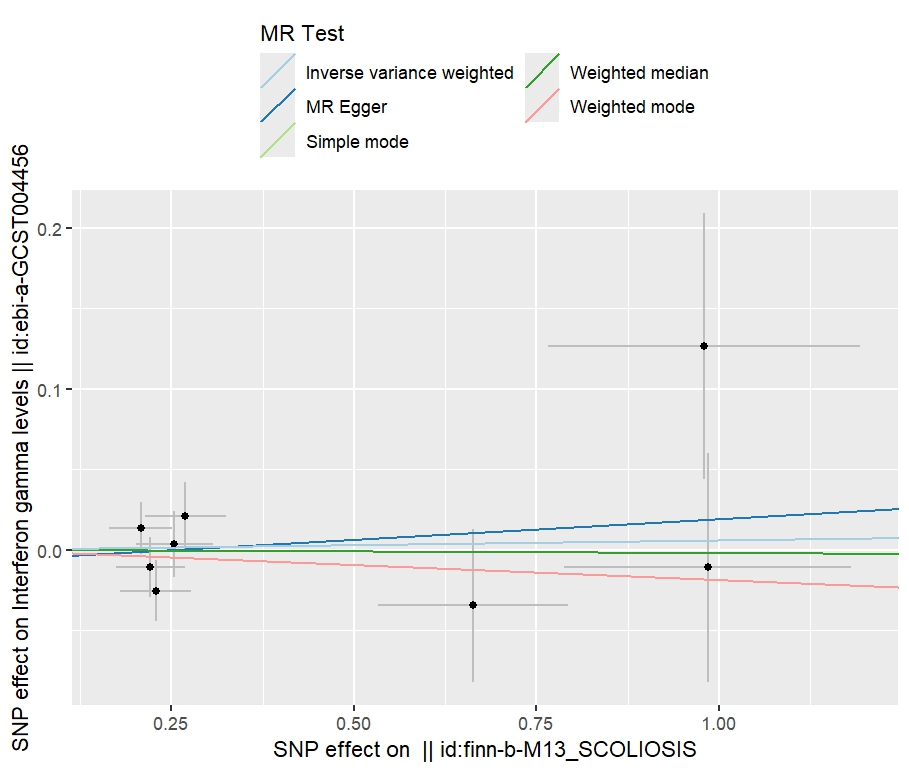

Supplement: Supplementary file 4 — Supplementary Material 4. [file JSP2-7-e70019-s002.zip › Supplementary Material 4/Exposureú║Scoliosisú1⁄4Outcomeú║inflammatory cytokines/IFN Y/Supplementary Material 4 IFN Y 1.jpeg]

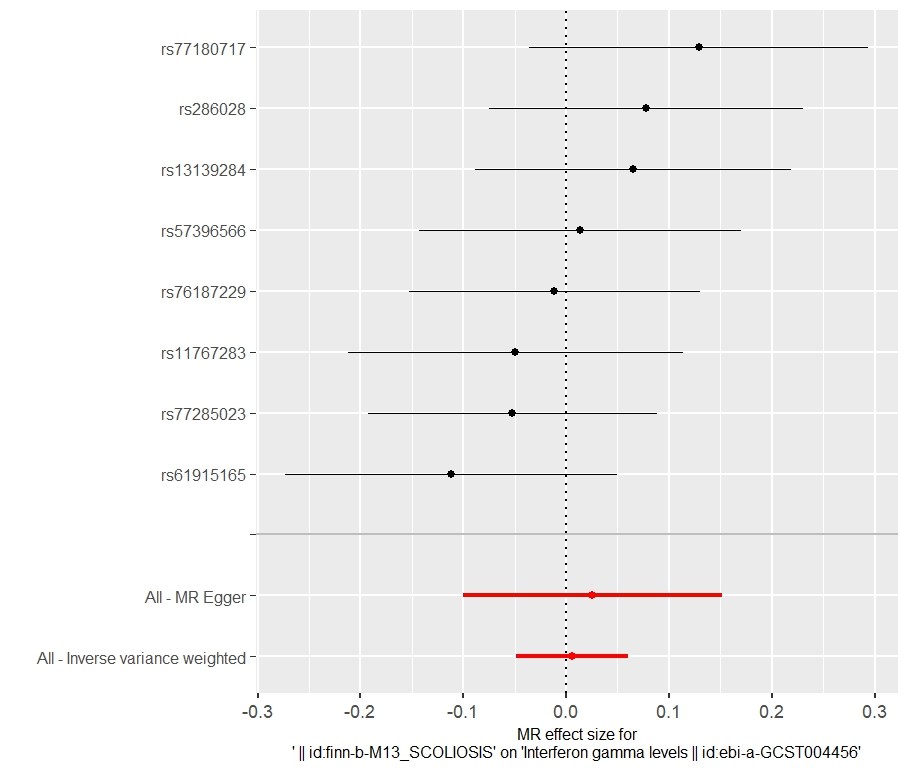

Supplement: Supplementary file 4 — Supplementary Material 4. [file JSP2-7-e70019-s002.zip › Supplementary Material 4/Exposureú║Scoliosisú1⁄4Outcomeú║inflammatory cytokines/IFN Y/Supplementary Material 4 IFN Y 2.jpeg]

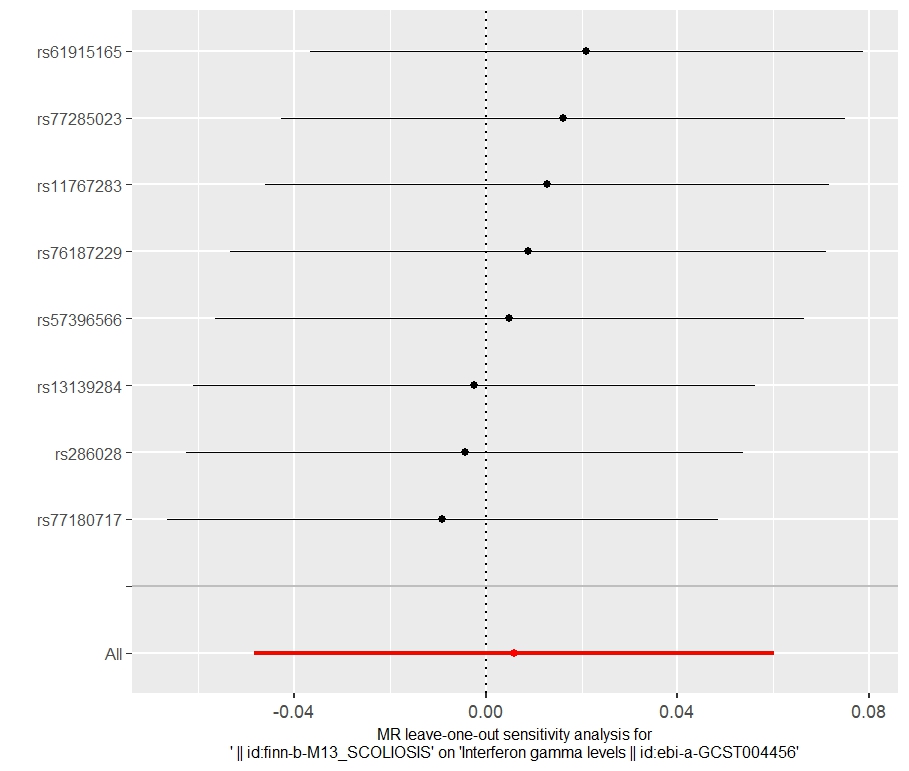

Supplement: Supplementary file 4 — Supplementary Material 4. [file JSP2-7-e70019-s002.zip › Supplementary Material 4/Exposureú║Scoliosisú1⁄4Outcomeú║inflammatory cytokines/IFN Y/Supplementary Material 4 IFN Y 3.jpeg]

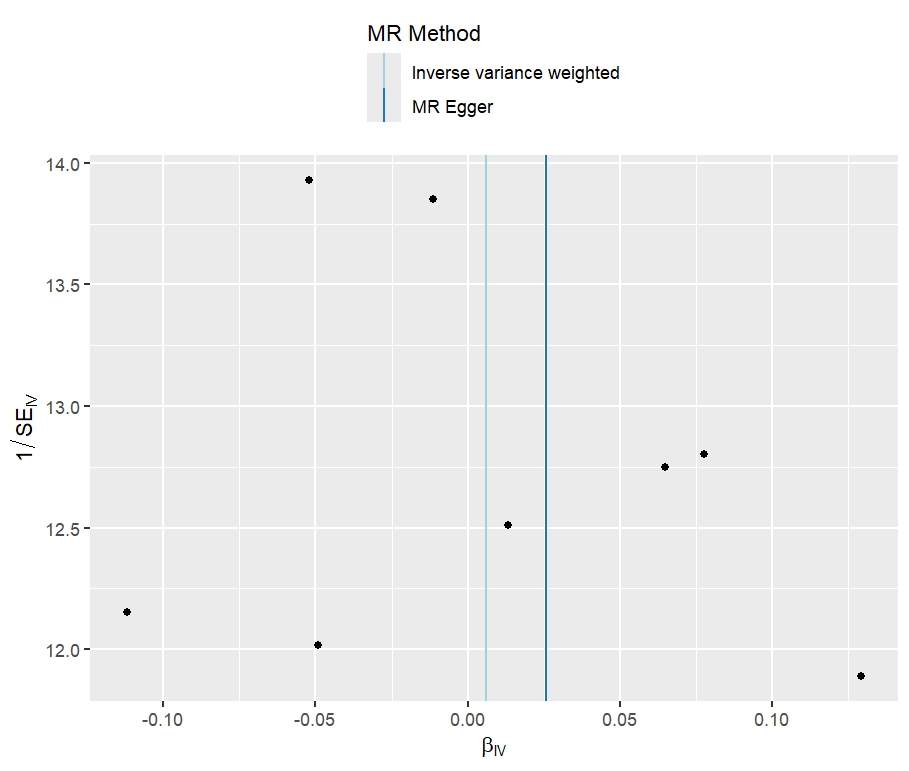

Supplement: Supplementary file 4 — Supplementary Material 4. [file JSP2-7-e70019-s002.zip › Supplementary Material 4/Exposureú║Scoliosisú1⁄4Outcomeú║inflammatory cytokines/IFN Y/Supplementary Material 4 IFN Y 4.jpeg]

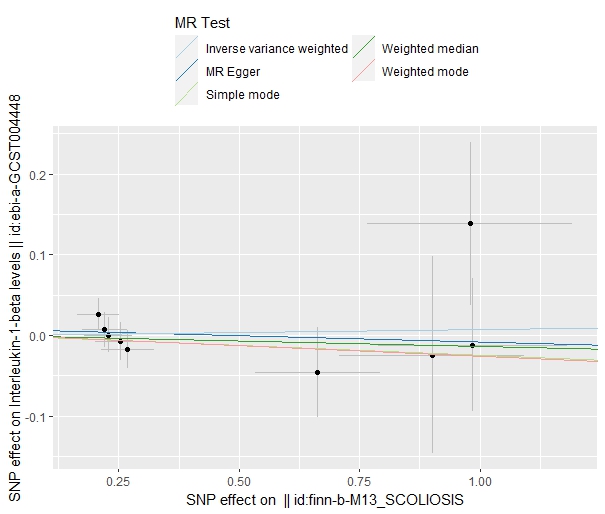

Supplement: Supplementary file 4 — Supplementary Material 4. [file JSP2-7-e70019-s002.zip › Supplementary Material 4/Exposureú║Scoliosisú1⁄4Outcomeú║inflammatory cytokines/IL1 B/Supplementary Material 4 IL1 B 1.jpeg]

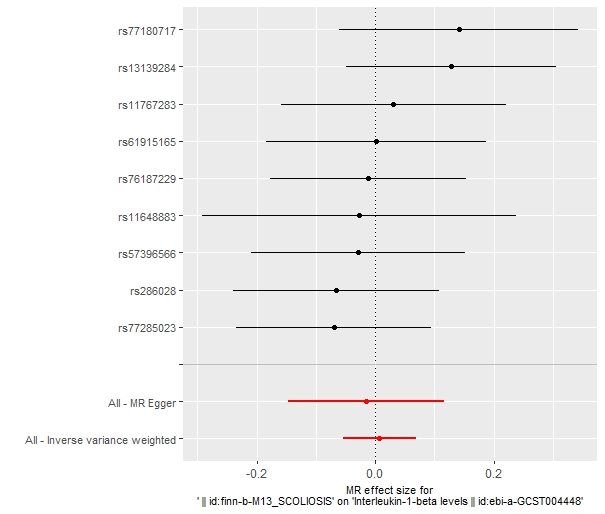

Supplement: Supplementary file 4 — Supplementary Material 4. [file JSP2-7-e70019-s002.zip › Supplementary Material 4/Exposureú║Scoliosisú1⁄4Outcomeú║inflammatory cytokines/IL1 B/Supplementary Material 4 IL1 B 2.jpeg]

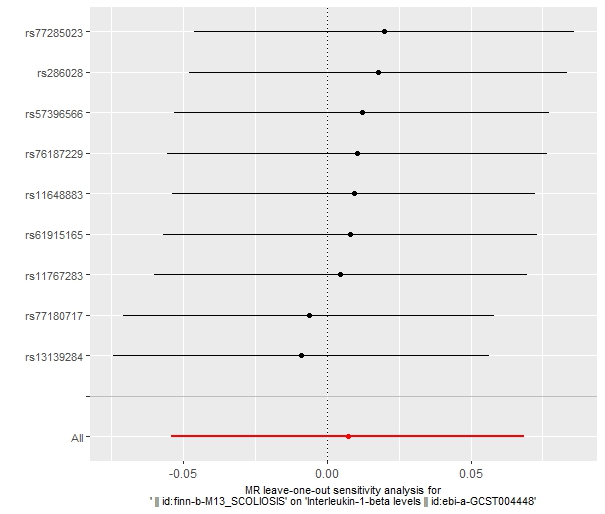

Supplement: Supplementary file 4 — Supplementary Material 4. [file JSP2-7-e70019-s002.zip › Supplementary Material 4/Exposureú║Scoliosisú1⁄4Outcomeú║inflammatory cytokines/IL1 B/Supplementary Material 4 IL1 B 3.jpeg]

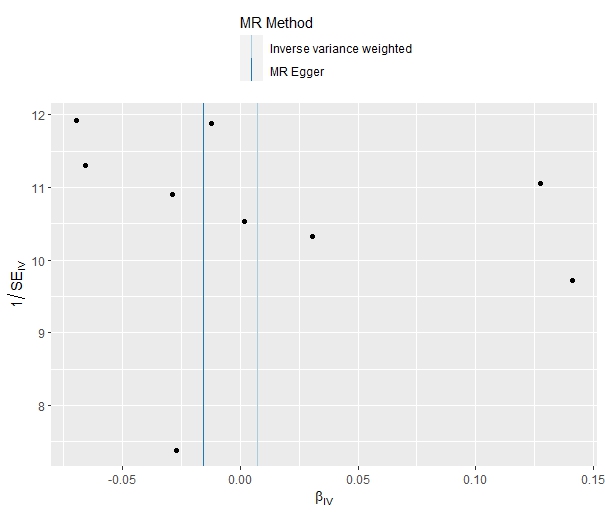

Supplement: Supplementary file 4 — Supplementary Material 4. [file JSP2-7-e70019-s002.zip › Supplementary Material 4/Exposureú║Scoliosisú1⁄4Outcomeú║inflammatory cytokines/IL1 B/Supplementary Material 4 IL1 B 4.jpeg]

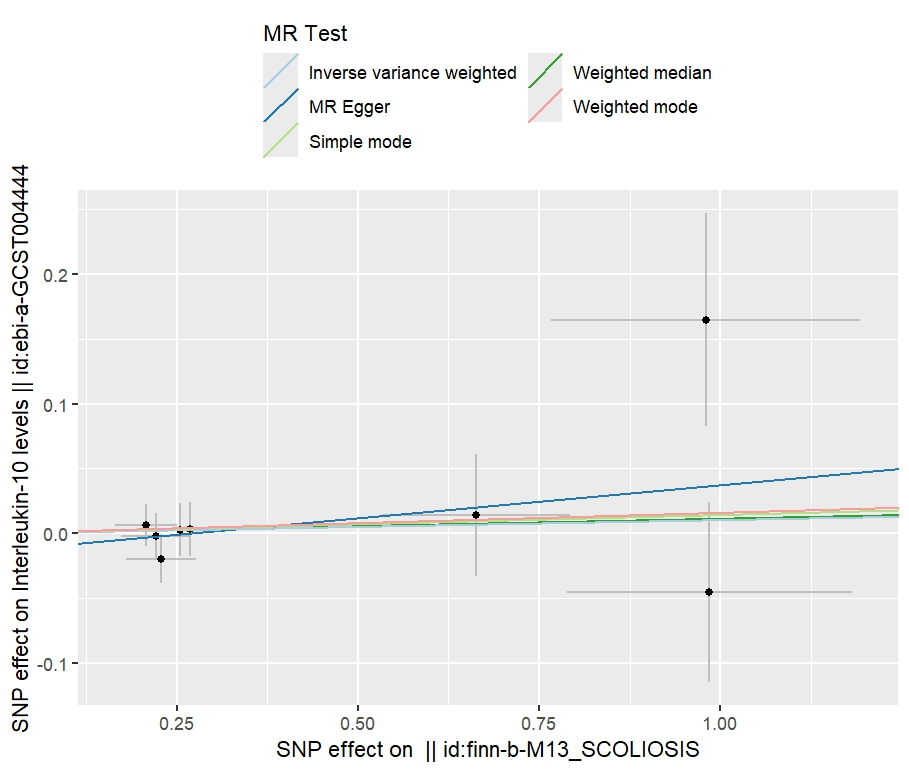

Supplement: Supplementary file 4 — Supplementary Material 4. [file JSP2-7-e70019-s002.zip › Supplementary Material 4/Exposureú║Scoliosisú1⁄4Outcomeú║inflammatory cytokines/IL10/Supplementary Material 4 IL10 1.jpeg]

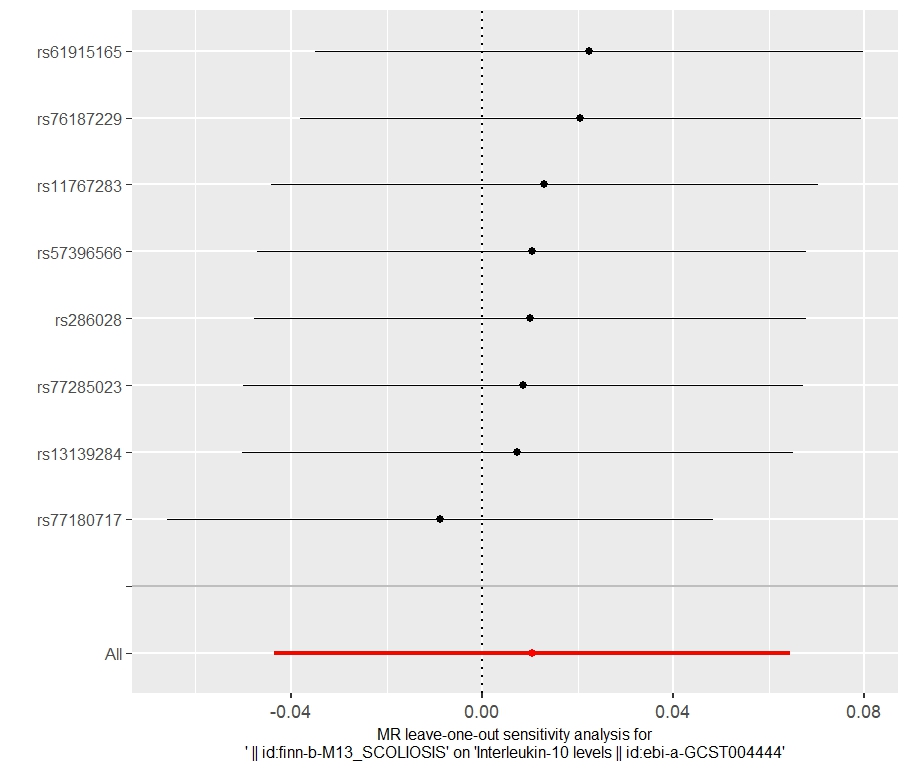

Supplement: Supplementary file 4 — Supplementary Material 4. [file JSP2-7-e70019-s002.zip › Supplementary Material 4/Exposureú║Scoliosisú1⁄4Outcomeú║inflammatory cytokines/IL10/Supplementary Material 4 IL10 2.jpeg]

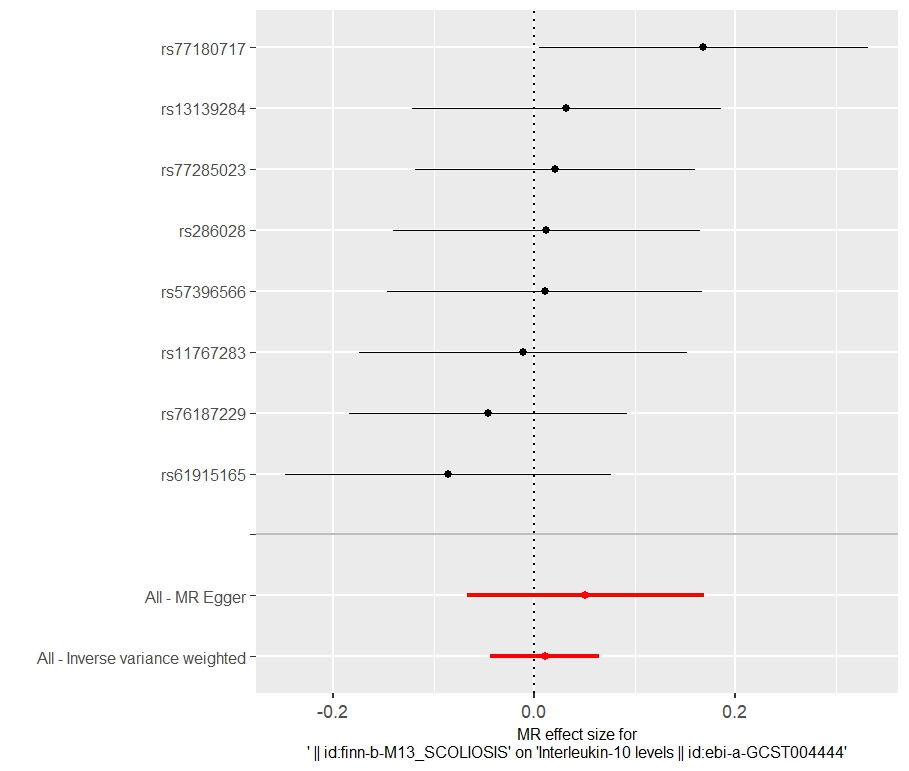

Supplement: Supplementary file 4 — Supplementary Material 4. [file JSP2-7-e70019-s002.zip › Supplementary Material 4/Exposureú║Scoliosisú1⁄4Outcomeú║inflammatory cytokines/IL10/Supplementary Material 4 IL10 3.jpeg]

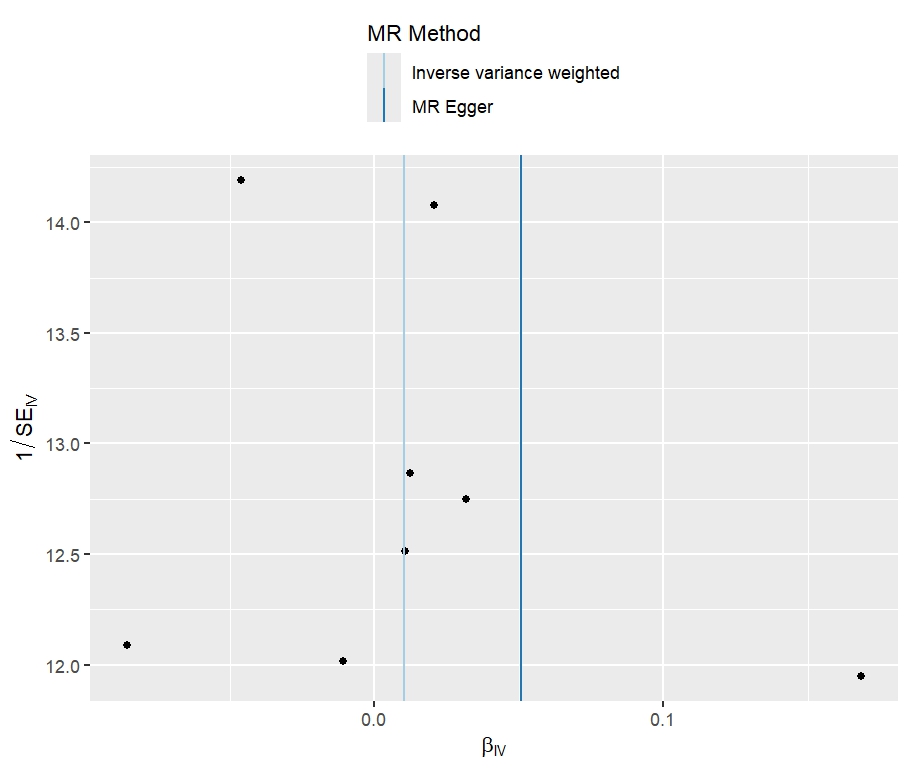

Supplement: Supplementary file 4 — Supplementary Material 4. [file JSP2-7-e70019-s002.zip › Supplementary Material 4/Exposureú║Scoliosisú1⁄4Outcomeú║inflammatory cytokines/IL10/Supplementary Material 4 IL10 4.jpeg]

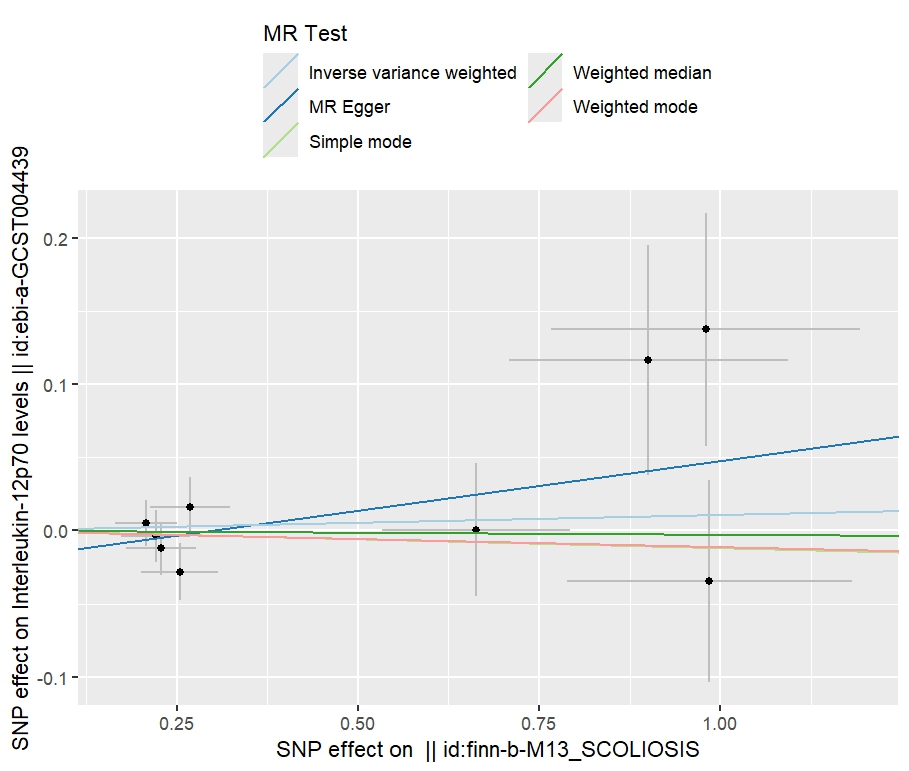

Supplement: Supplementary file 4 — Supplementary Material 4. [file JSP2-7-e70019-s002.zip › Supplementary Material 4/Exposureú║Scoliosisú1⁄4Outcomeú║inflammatory cytokines/IL12p70/Supplementary Material 4 IL12p70 1.jpeg]

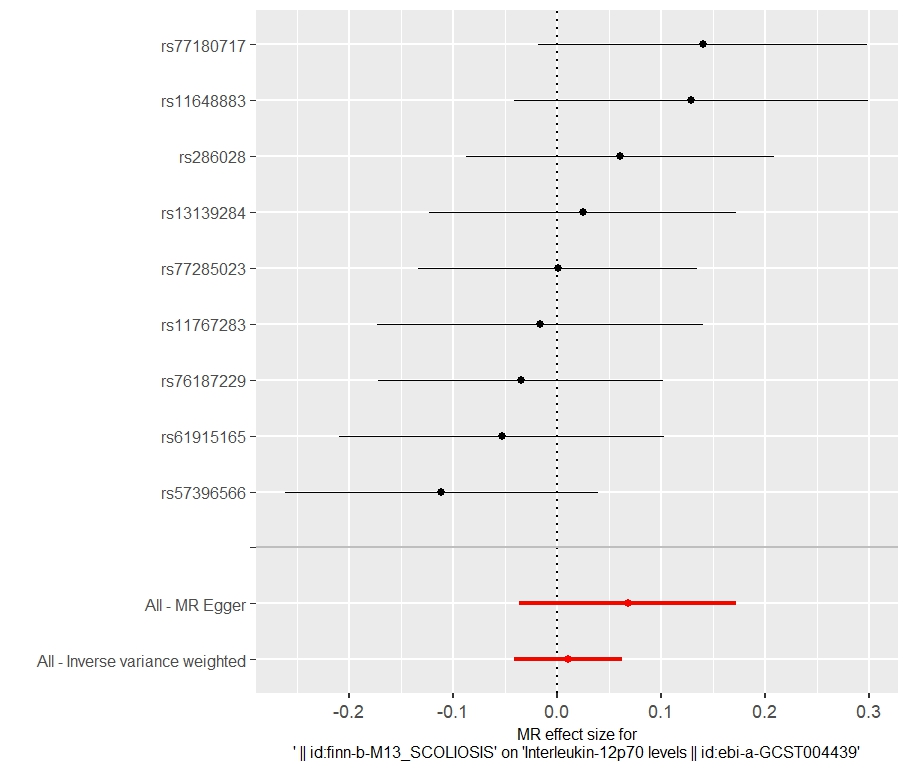

Supplement: Supplementary file 4 — Supplementary Material 4. [file JSP2-7-e70019-s002.zip › Supplementary Material 4/Exposureú║Scoliosisú1⁄4Outcomeú║inflammatory cytokines/IL12p70/Supplementary Material 4 IL12p70 2.jpeg]

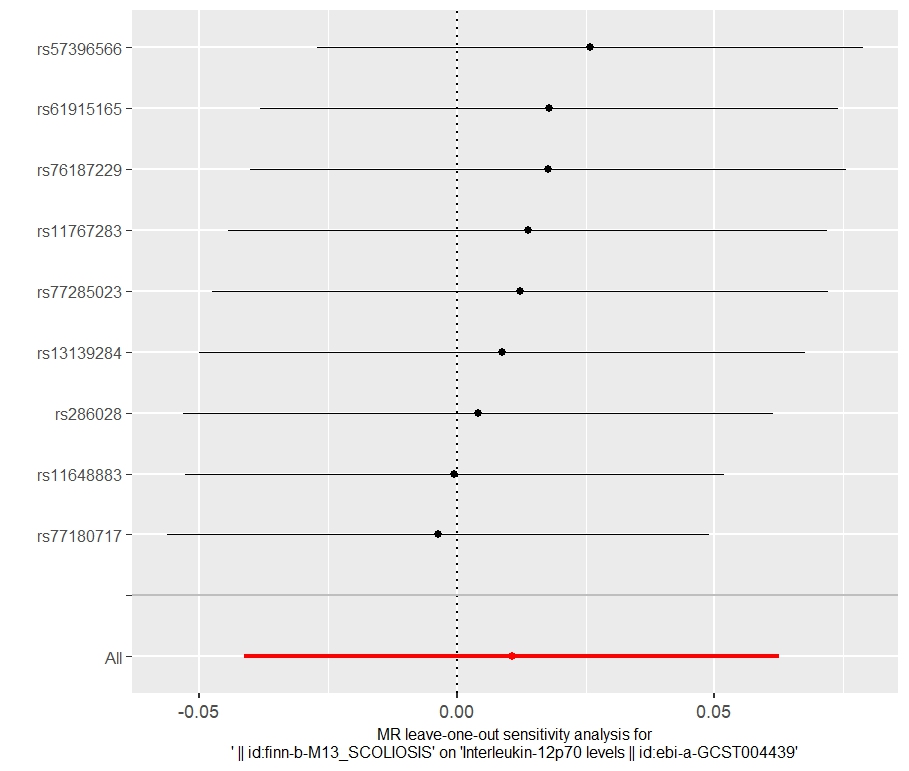

Supplement: Supplementary file 4 — Supplementary Material 4. [file JSP2-7-e70019-s002.zip › Supplementary Material 4/Exposureú║Scoliosisú1⁄4Outcomeú║inflammatory cytokines/IL12p70/Supplementary Material 4 IL12p70 3.jpeg]

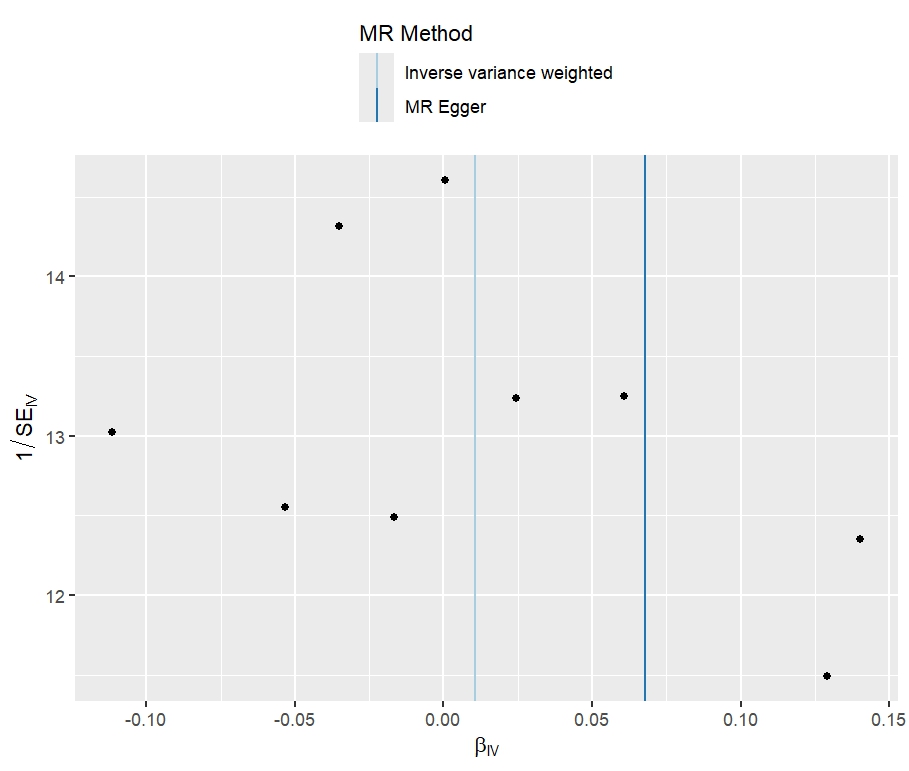

Supplement: Supplementary file 4 — Supplementary Material 4. [file JSP2-7-e70019-s002.zip › Supplementary Material 4/Exposureú║Scoliosisú1⁄4Outcomeú║inflammatory cytokines/IL12p70/Supplementary Material 4 IL12p70 4.jpeg]

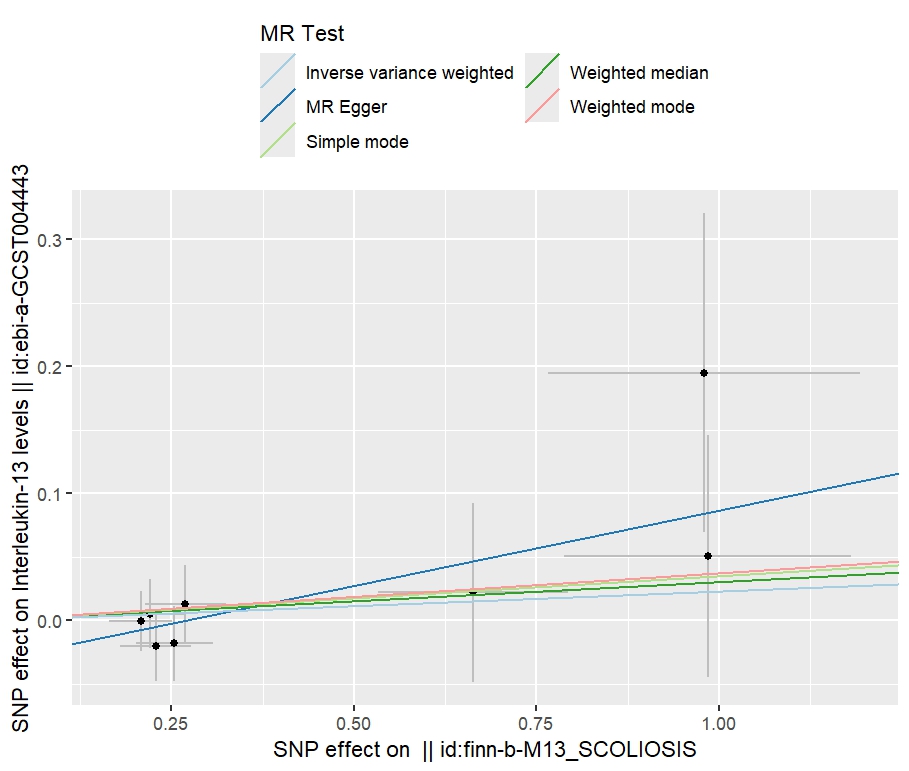

Supplement: Supplementary file 4 — Supplementary Material 4. [file JSP2-7-e70019-s002.zip › Supplementary Material 4/Exposureú║Scoliosisú1⁄4Outcomeú║inflammatory cytokines/IL13/Supplementary Material 4 IL13 1.jpeg]

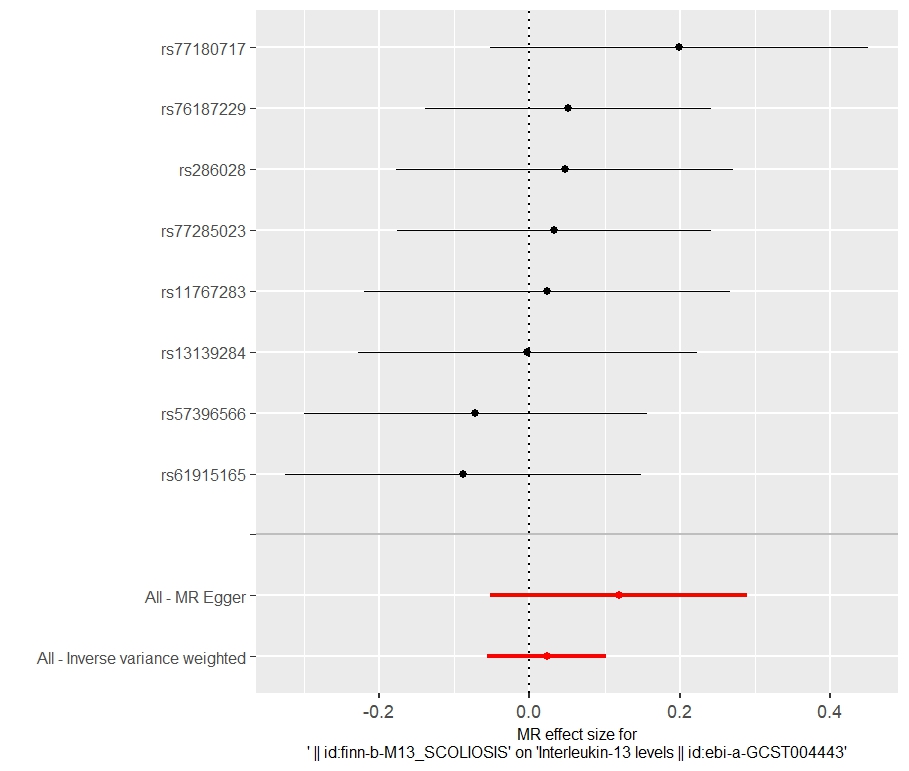

Supplement: Supplementary file 4 — Supplementary Material 4. [file JSP2-7-e70019-s002.zip › Supplementary Material 4/Exposureú║Scoliosisú1⁄4Outcomeú║inflammatory cytokines/IL13/Supplementary Material 4 IL13 2.jpeg]

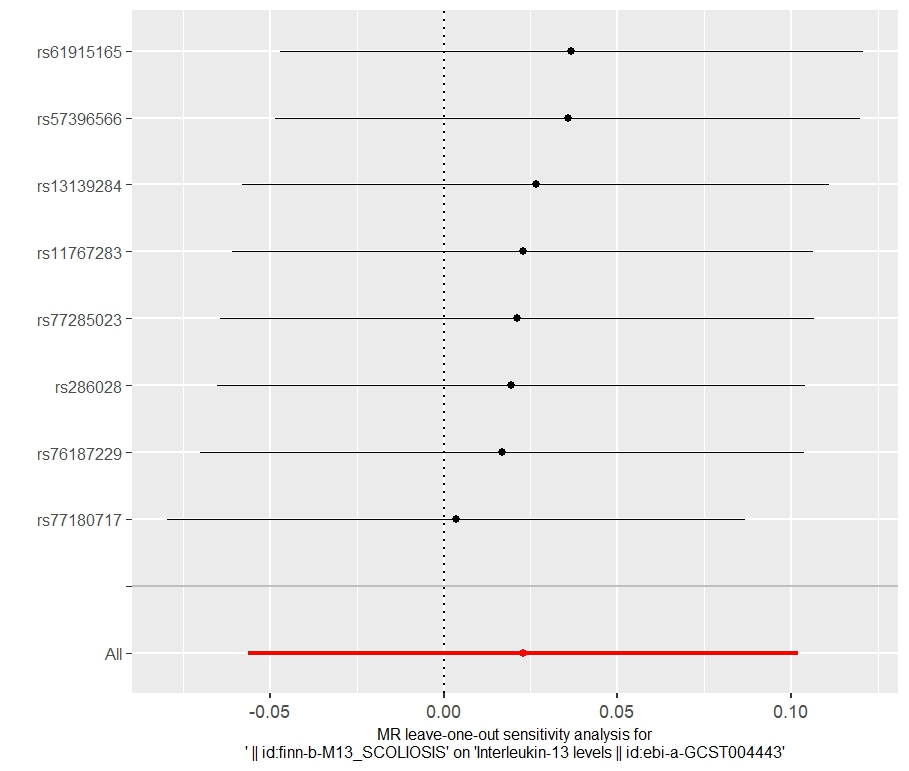

Supplement: Supplementary file 4 — Supplementary Material 4. [file JSP2-7-e70019-s002.zip › Supplementary Material 4/Exposureú║Scoliosisú1⁄4Outcomeú║inflammatory cytokines/IL13/Supplementary Material 4 IL13 3.jpeg]

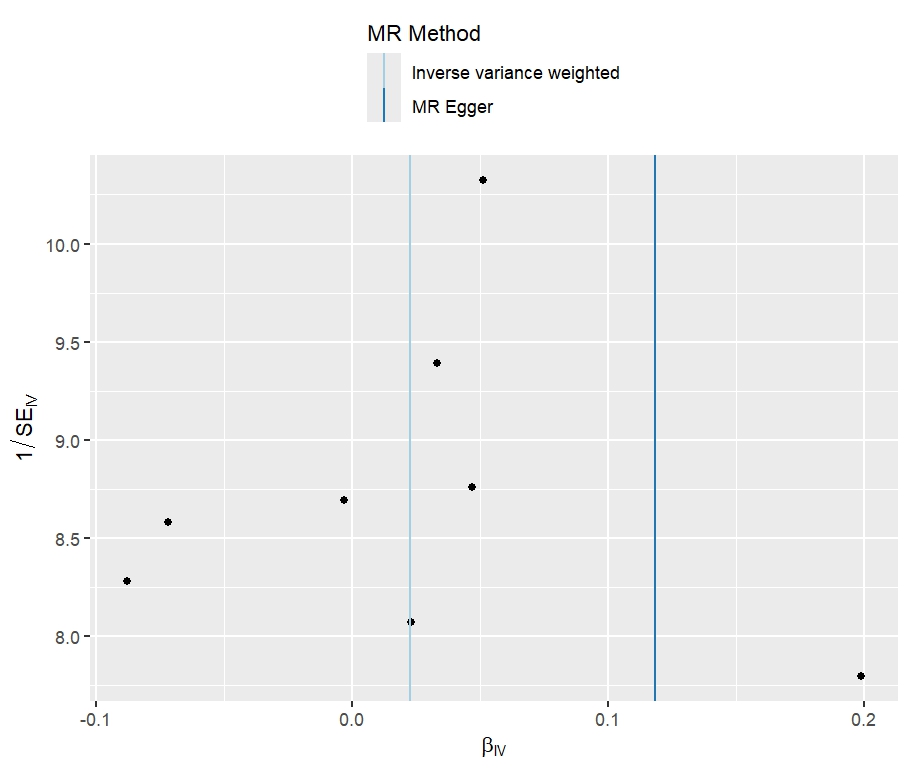

Supplement: Supplementary file 4 — Supplementary Material 4. [file JSP2-7-e70019-s002.zip › Supplementary Material 4/Exposureú║Scoliosisú1⁄4Outcomeú║inflammatory cytokines/IL13/Supplementary Material 4 IL13 4.jpeg]

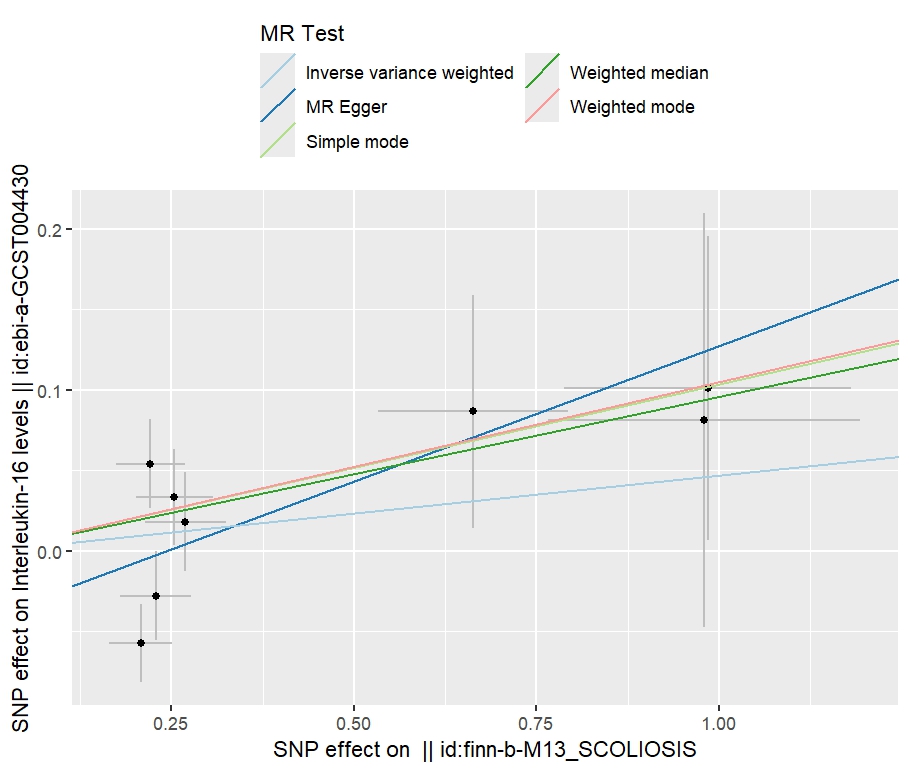

Supplement: Supplementary file 4 — Supplementary Material 4. [file JSP2-7-e70019-s002.zip › Supplementary Material 4/Exposureú║Scoliosisú1⁄4Outcomeú║inflammatory cytokines/IL16/Supplementary Material 4 IL16 1.jpeg]

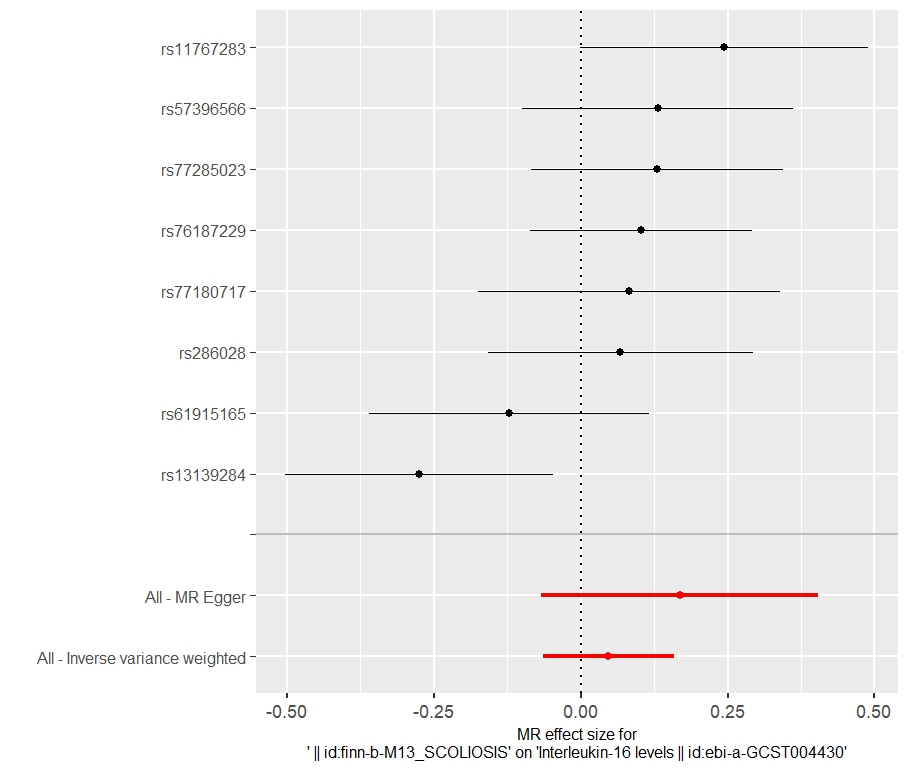

Supplement: Supplementary file 4 — Supplementary Material 4. [file JSP2-7-e70019-s002.zip › Supplementary Material 4/Exposureú║Scoliosisú1⁄4Outcomeú║inflammatory cytokines/IL16/Supplementary Material 4 IL16 2.jpeg]

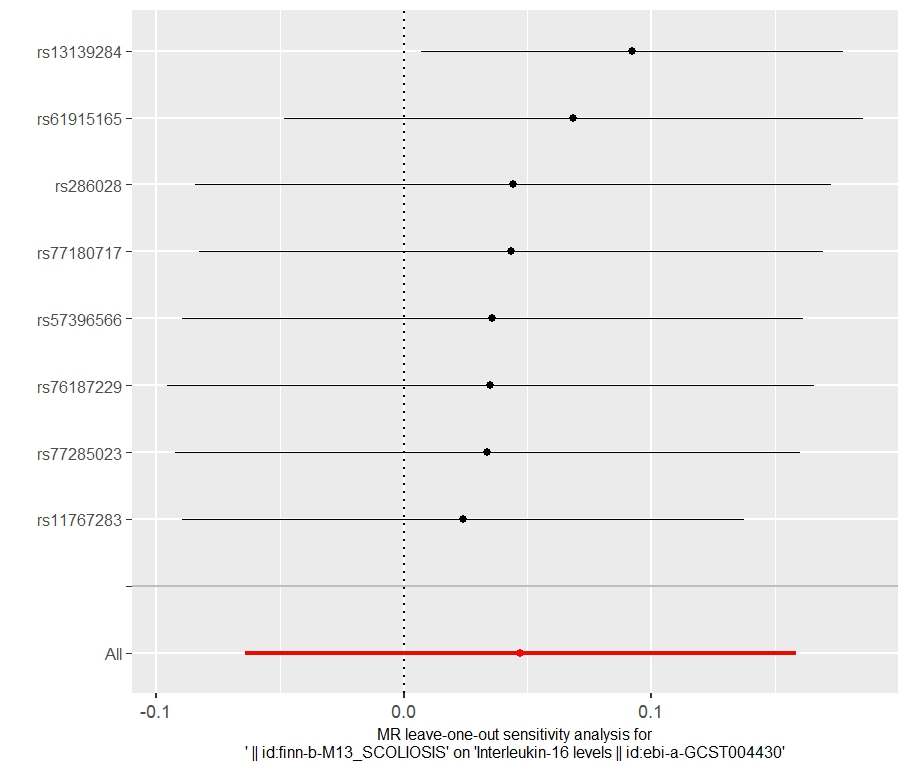

Supplement: Supplementary file 4 — Supplementary Material 4. [file JSP2-7-e70019-s002.zip › Supplementary Material 4/Exposureú║Scoliosisú1⁄4Outcomeú║inflammatory cytokines/IL16/Supplementary Material 4 IL16 3.jpeg]

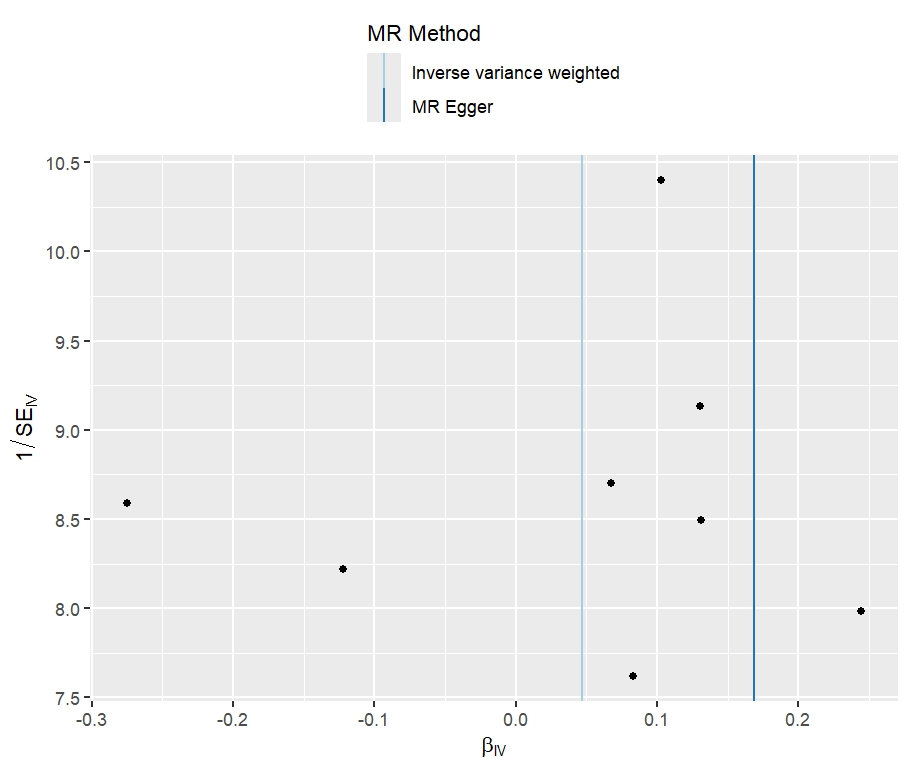

Supplement: Supplementary file 4 — Supplementary Material 4. [file JSP2-7-e70019-s002.zip › Supplementary Material 4/Exposureú║Scoliosisú1⁄4Outcomeú║inflammatory cytokines/IL16/Supplementary Material 4 IL16 4.jpeg]

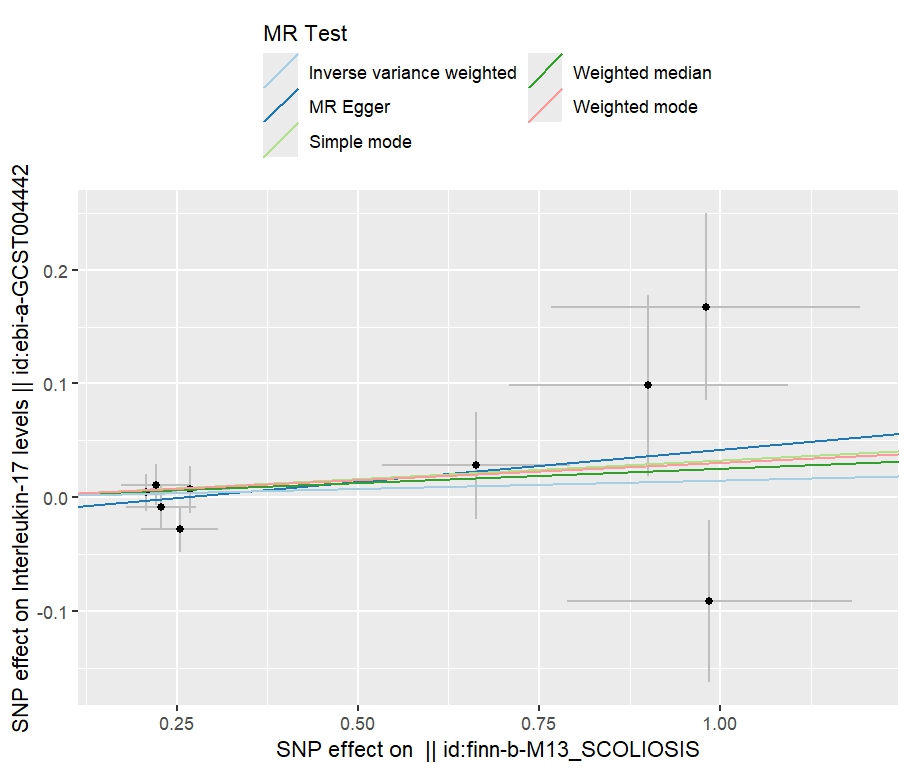

Supplement: Supplementary file 4 — Supplementary Material 4. [file JSP2-7-e70019-s002.zip › Supplementary Material 4/Exposureú║Scoliosisú1⁄4Outcomeú║inflammatory cytokines/IL17/Supplementary Material 4 IL17 1.jpeg]

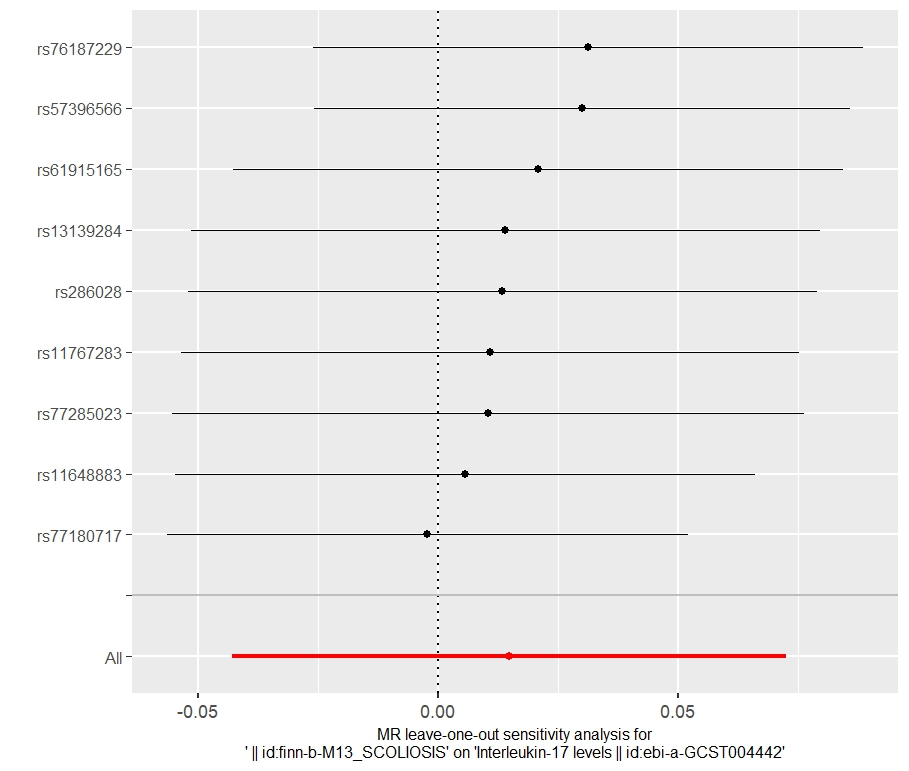

Supplement: Supplementary file 4 — Supplementary Material 4. [file JSP2-7-e70019-s002.zip › Supplementary Material 4/Exposureú║Scoliosisú1⁄4Outcomeú║inflammatory cytokines/IL17/Supplementary Material 4 IL17 2.jpeg]

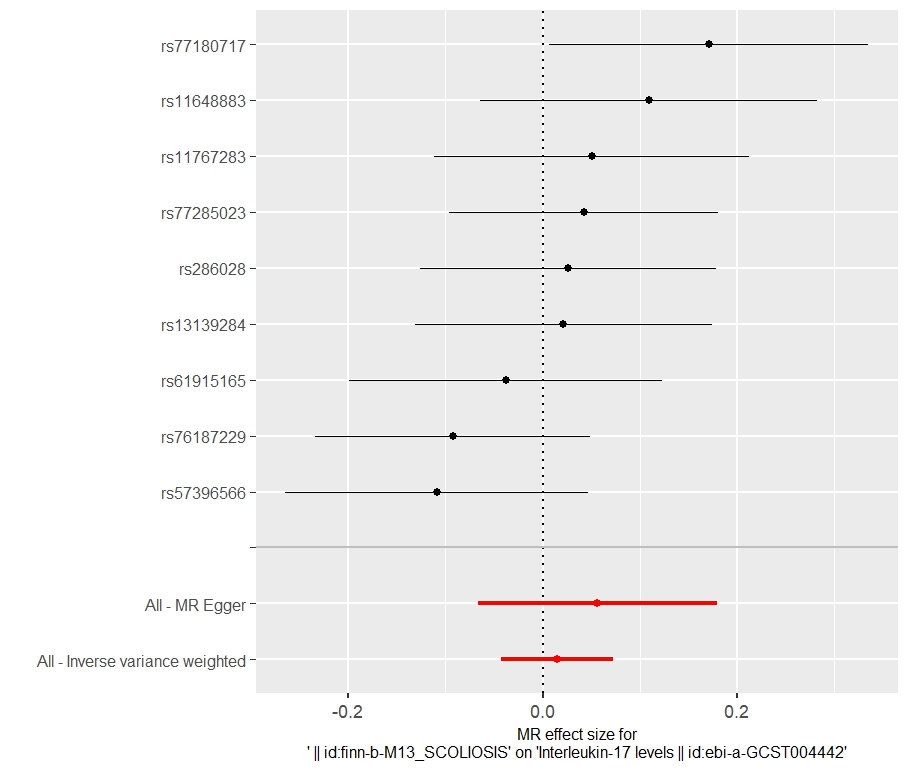

Supplement: Supplementary file 4 — Supplementary Material 4. [file JSP2-7-e70019-s002.zip › Supplementary Material 4/Exposureú║Scoliosisú1⁄4Outcomeú║inflammatory cytokines/IL17/Supplementary Material 4 IL17 3.jpeg]

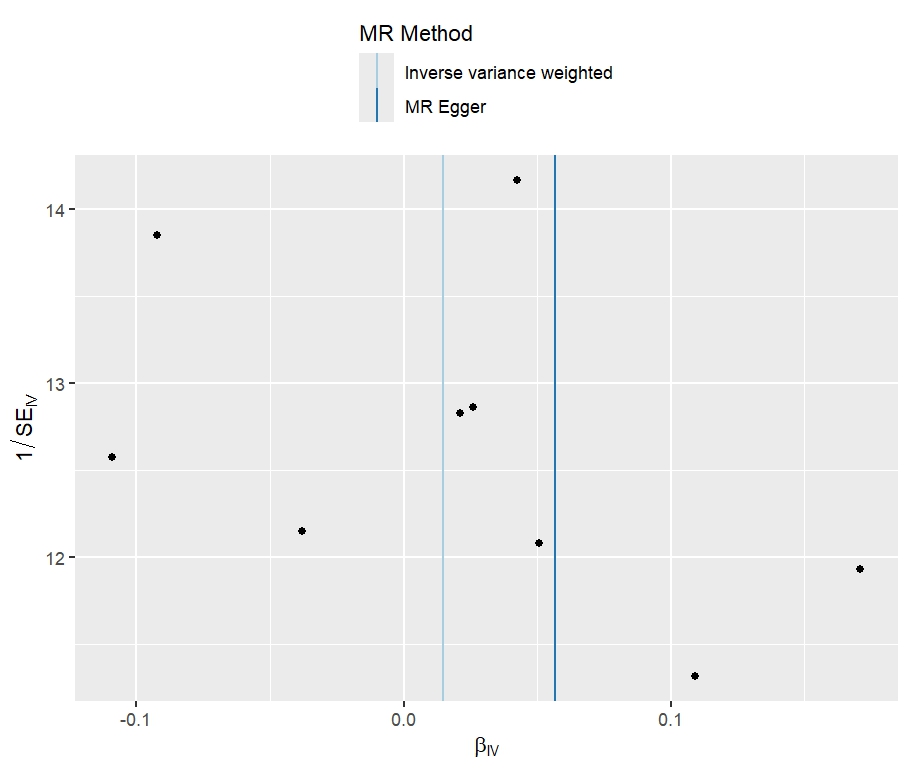

Supplement: Supplementary file 4 — Supplementary Material 4. [file JSP2-7-e70019-s002.zip › Supplementary Material 4/Exposureú║Scoliosisú1⁄4Outcomeú║inflammatory cytokines/IL17/Supplementary Material 4 IL17 4.jpeg]

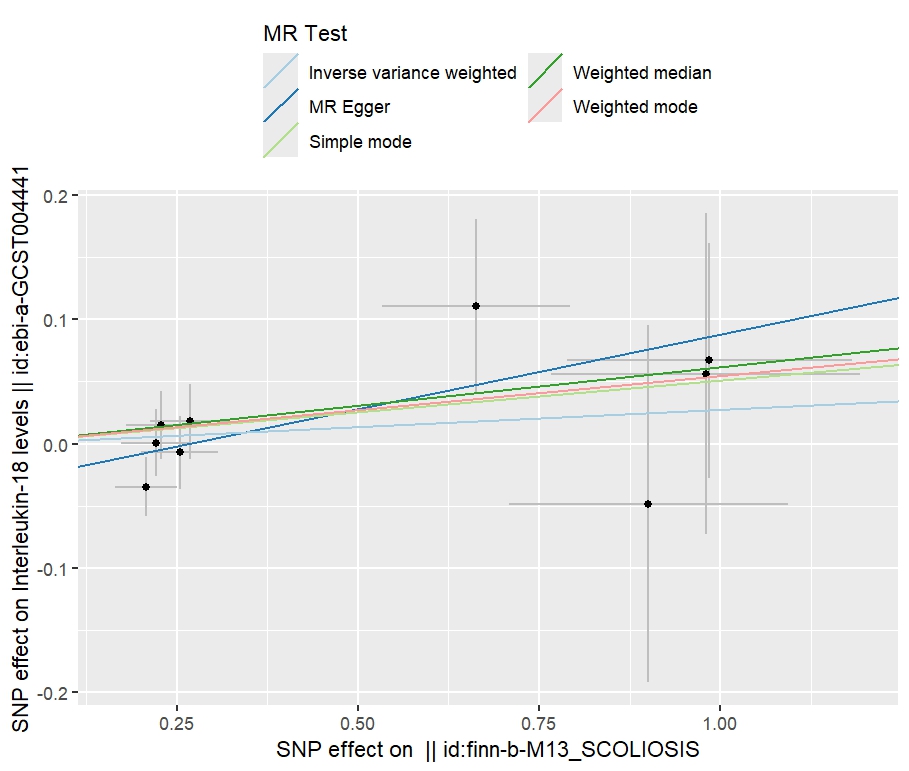

Supplement: Supplementary file 4 — Supplementary Material 4. [file JSP2-7-e70019-s002.zip › Supplementary Material 4/Exposureú║Scoliosisú1⁄4Outcomeú║inflammatory cytokines/IL18/Supplementary Material 4 IL18 1.jpeg]

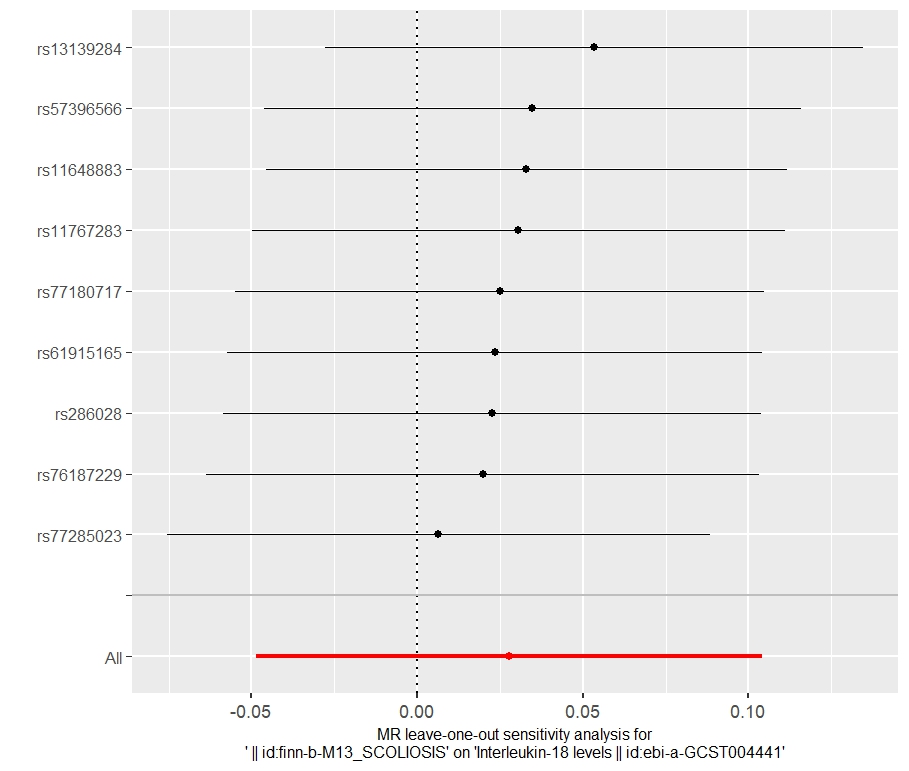

Supplement: Supplementary file 4 — Supplementary Material 4. [file JSP2-7-e70019-s002.zip › Supplementary Material 4/Exposureú║Scoliosisú1⁄4Outcomeú║inflammatory cytokines/IL18/Supplementary Material 4 IL18 2.jpeg]

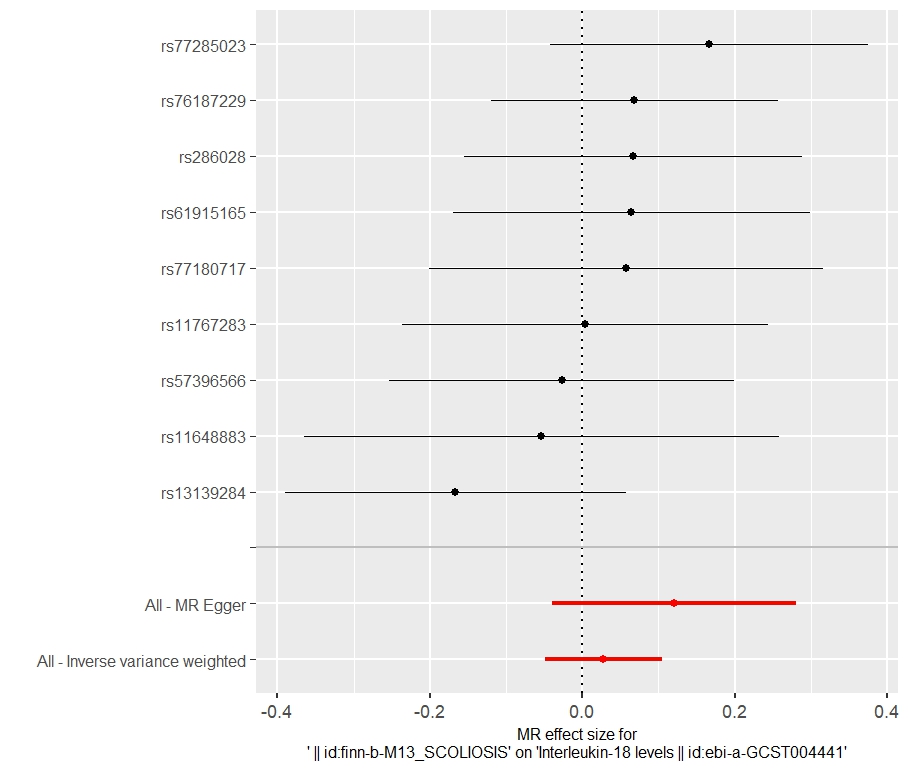

Supplement: Supplementary file 4 — Supplementary Material 4. [file JSP2-7-e70019-s002.zip › Supplementary Material 4/Exposureú║Scoliosisú1⁄4Outcomeú║inflammatory cytokines/IL18/Supplementary Material 4 IL18 3.jpeg]

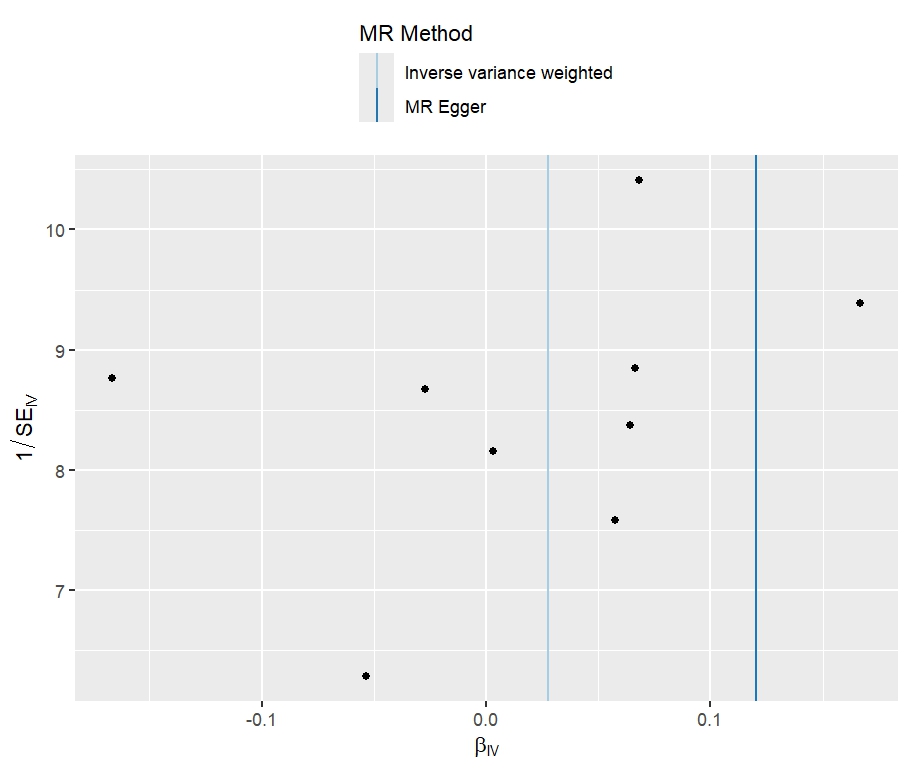

Supplement: Supplementary file 4 — Supplementary Material 4. [file JSP2-7-e70019-s002.zip › Supplementary Material 4/Exposureú║Scoliosisú1⁄4Outcomeú║inflammatory cytokines/IL18/Supplementary Material 4 IL18 4.jpeg]

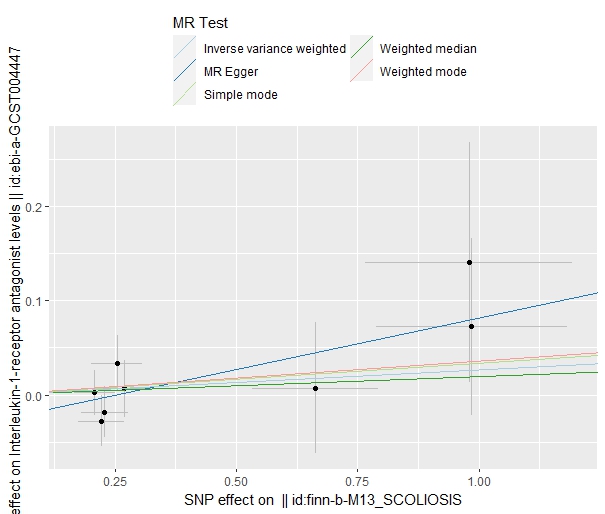

Supplement: Supplementary file 4 — Supplementary Material 4. [file JSP2-7-e70019-s002.zip › Supplementary Material 4/Exposureú║Scoliosisú1⁄4Outcomeú║inflammatory cytokines/IL1RA/Supplementary Material 4 IL1RA 1.jpeg]

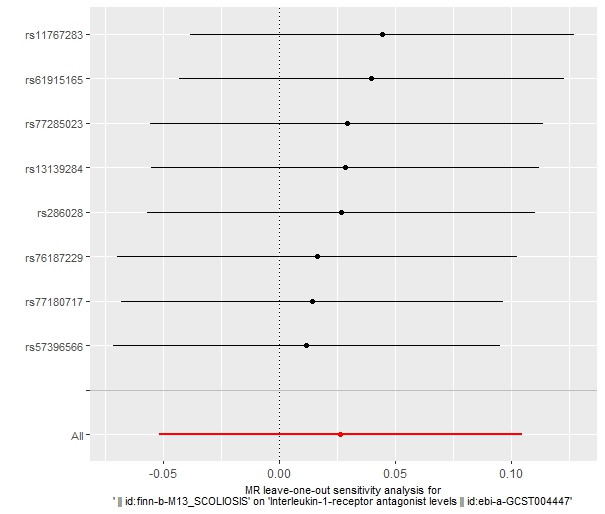

Supplement: Supplementary file 4 — Supplementary Material 4. [file JSP2-7-e70019-s002.zip › Supplementary Material 4/Exposureú║Scoliosisú1⁄4Outcomeú║inflammatory cytokines/IL1RA/Supplementary Material 4 IL1RA 2.jpeg]

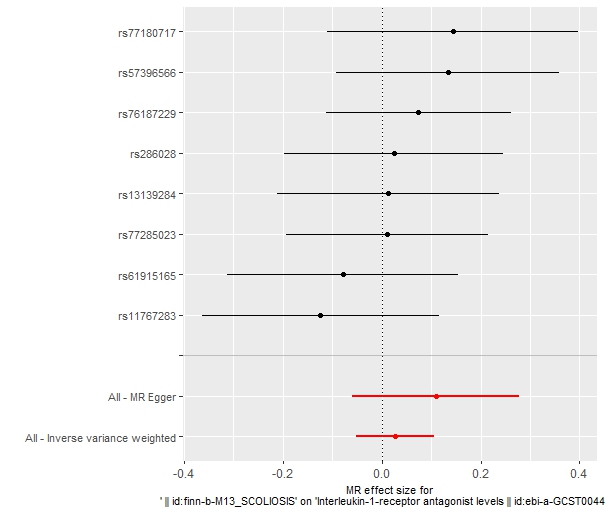

Supplement: Supplementary file 4 — Supplementary Material 4. [file JSP2-7-e70019-s002.zip › Supplementary Material 4/Exposureú║Scoliosisú1⁄4Outcomeú║inflammatory cytokines/IL1RA/Supplementary Material 4 IL1RA 3.jpeg]

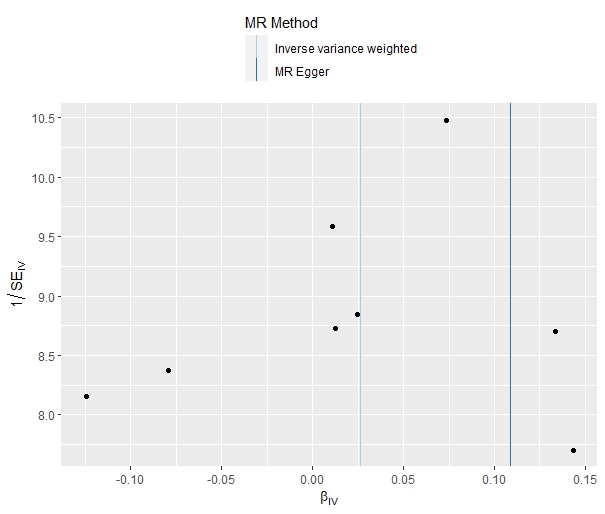

Supplement: Supplementary file 4 — Supplementary Material 4. [file JSP2-7-e70019-s002.zip › Supplementary Material 4/Exposureú║Scoliosisú1⁄4Outcomeú║inflammatory cytokines/IL1RA/Supplementary Material 4 IL1RA 4.jpeg]

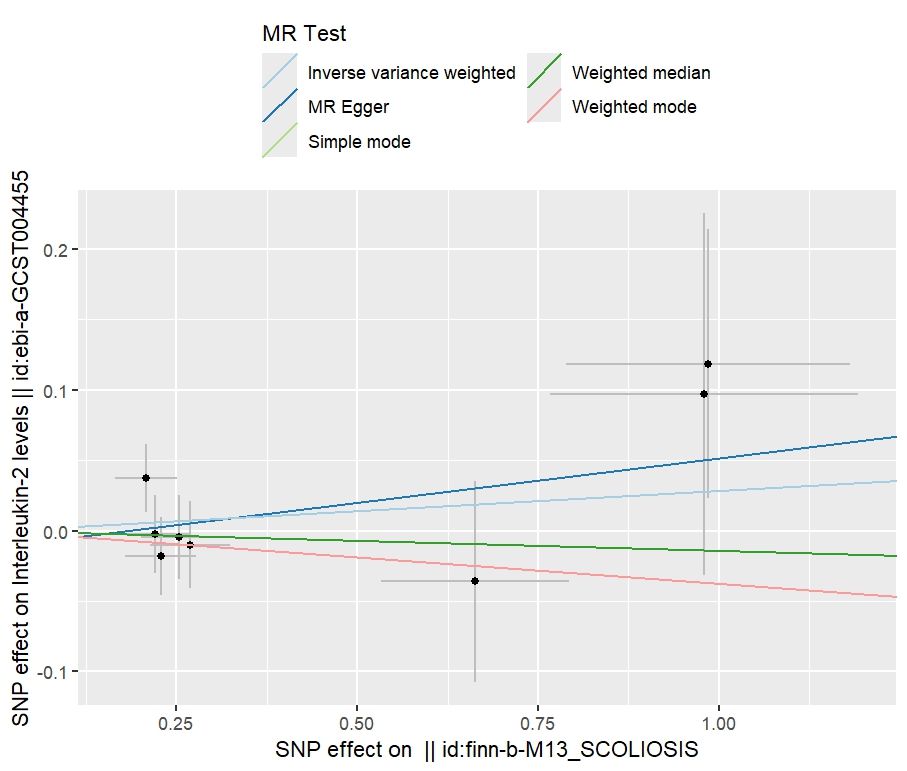

Supplement: Supplementary file 4 — Supplementary Material 4. [file JSP2-7-e70019-s002.zip › Supplementary Material 4/Exposureú║Scoliosisú1⁄4Outcomeú║inflammatory cytokines/IL2/Supplementary Material 4 IL2 1.jpeg]

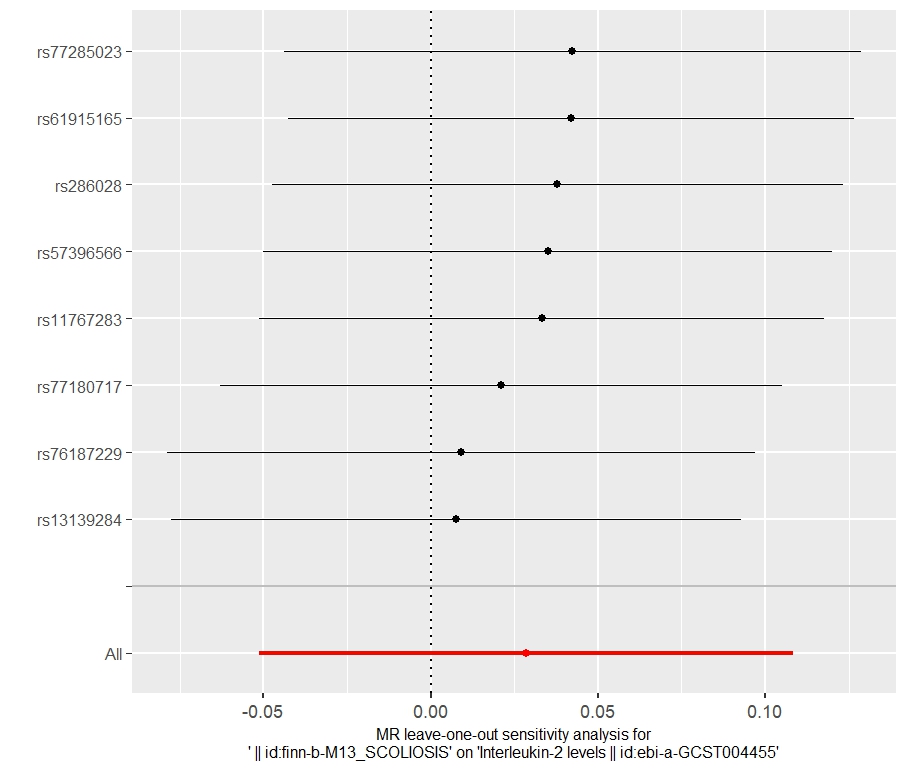

Supplement: Supplementary file 4 — Supplementary Material 4. [file JSP2-7-e70019-s002.zip › Supplementary Material 4/Exposureú║Scoliosisú1⁄4Outcomeú║inflammatory cytokines/IL2/Supplementary Material 4 IL2 2.jpeg]

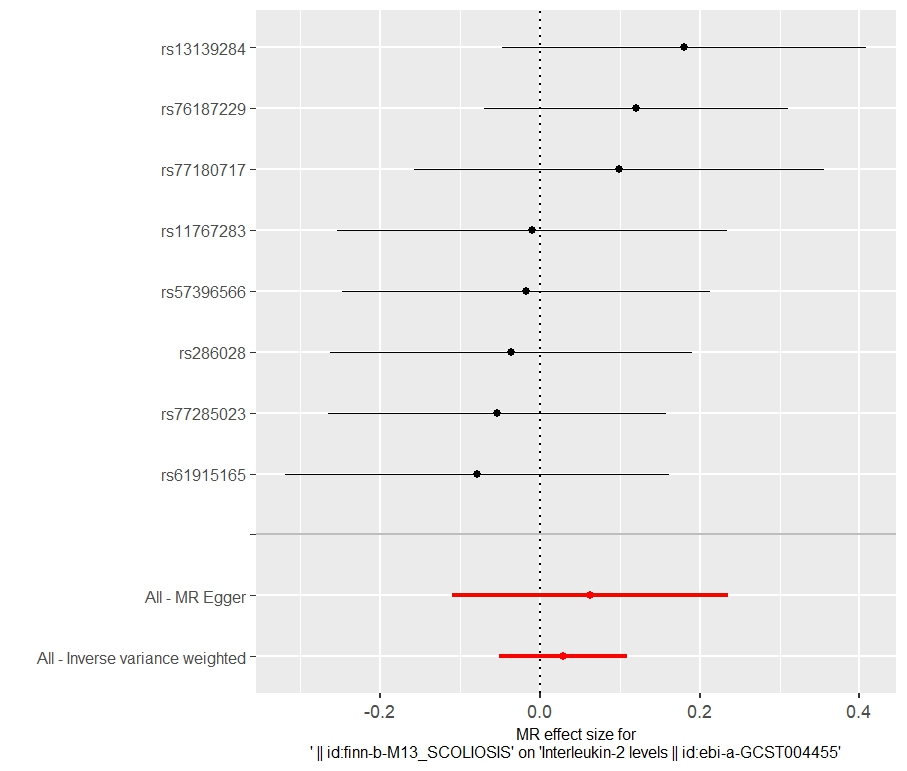

Supplement: Supplementary file 4 — Supplementary Material 4. [file JSP2-7-e70019-s002.zip › Supplementary Material 4/Exposureú║Scoliosisú1⁄4Outcomeú║inflammatory cytokines/IL2/Supplementary Material 4 IL2 3.jpeg]

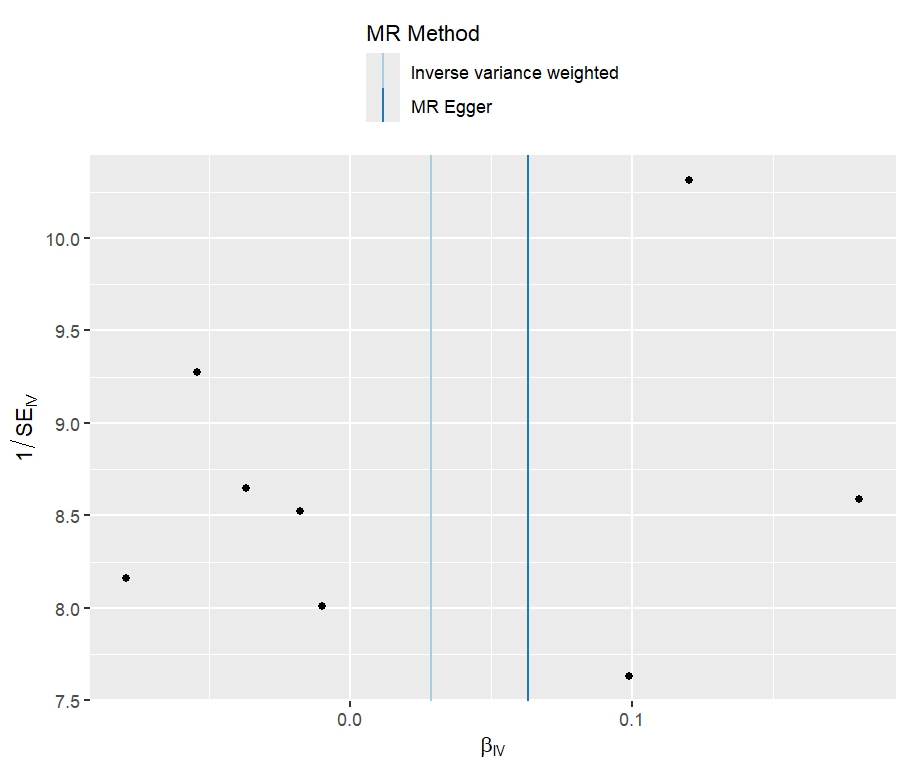

Supplement: Supplementary file 4 — Supplementary Material 4. [file JSP2-7-e70019-s002.zip › Supplementary Material 4/Exposureú║Scoliosisú1⁄4Outcomeú║inflammatory cytokines/IL2/Supplementary Material 4 IL2 4.jpeg]

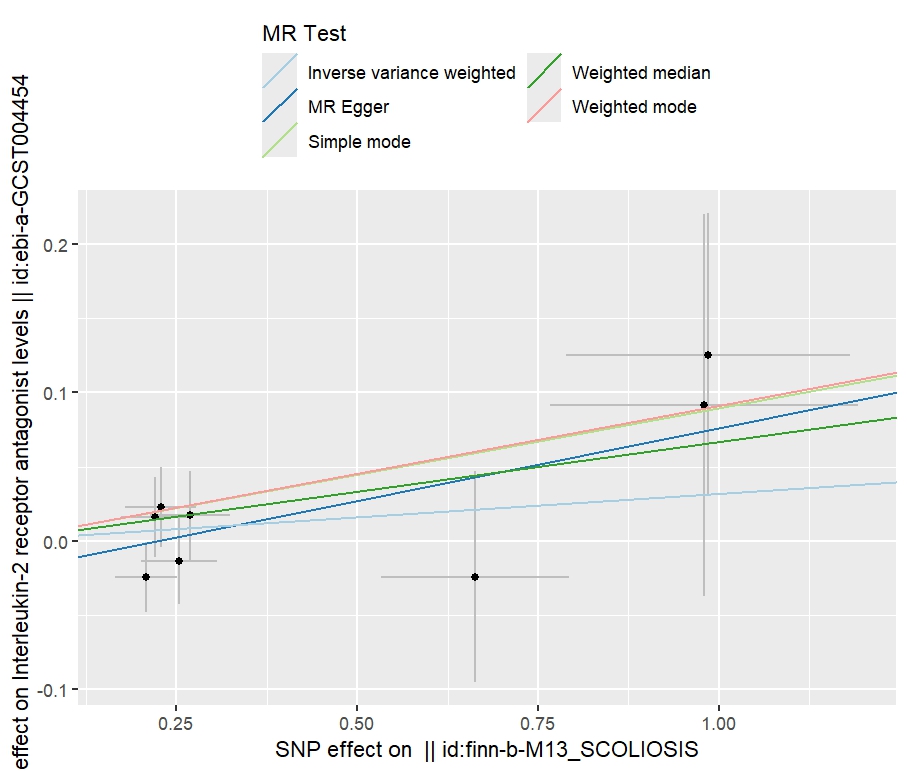

Supplement: Supplementary file 4 — Supplementary Material 4. [file JSP2-7-e70019-s002.zip › Supplementary Material 4/Exposureú║Scoliosisú1⁄4Outcomeú║inflammatory cytokines/IL2RA/Supplementary Material 4 IL2RA 1.jpeg]

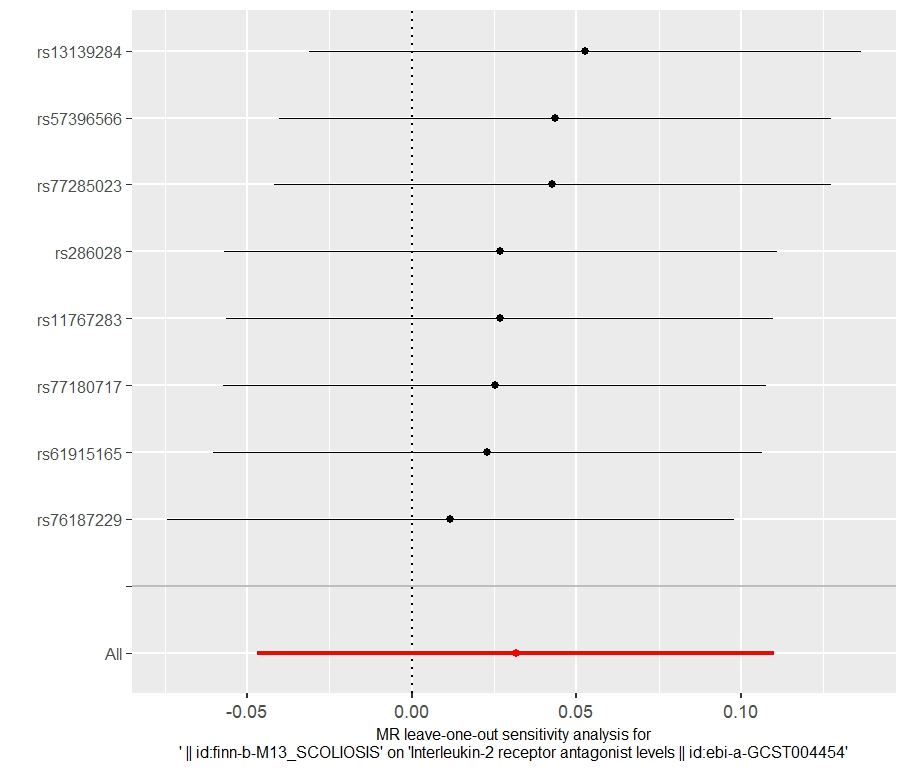

Supplement: Supplementary file 4 — Supplementary Material 4. [file JSP2-7-e70019-s002.zip › Supplementary Material 4/Exposureú║Scoliosisú1⁄4Outcomeú║inflammatory cytokines/IL2RA/Supplementary Material 4 IL2RA 2.jpeg]

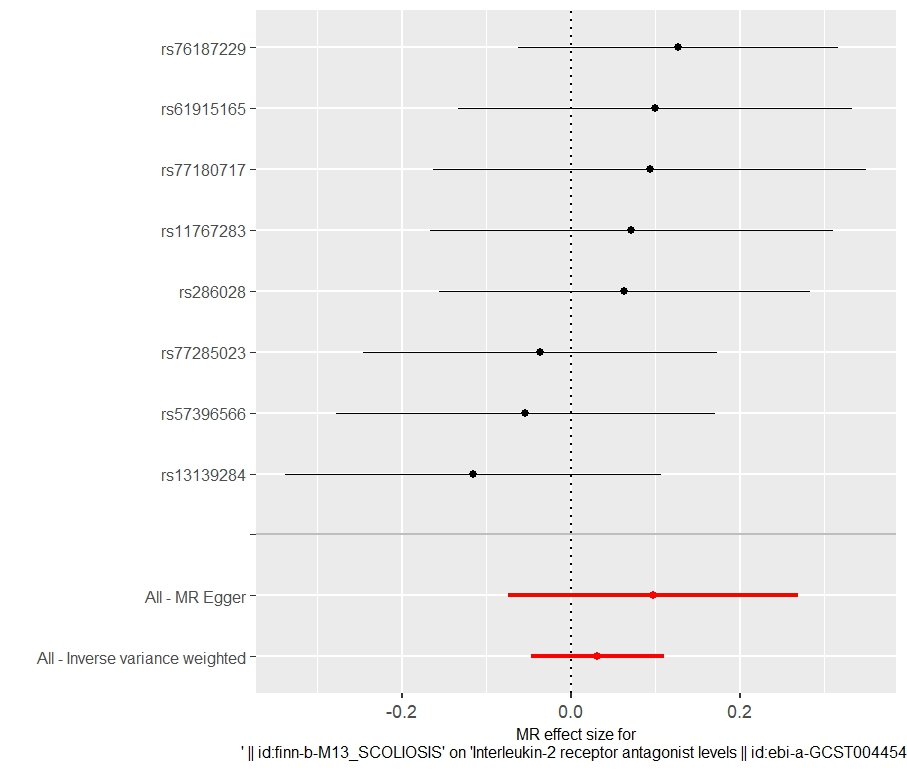

Supplement: Supplementary file 4 — Supplementary Material 4. [file JSP2-7-e70019-s002.zip › Supplementary Material 4/Exposureú║Scoliosisú1⁄4Outcomeú║inflammatory cytokines/IL2RA/Supplementary Material 4 IL2RA 3.jpeg]
