# Supplementary figures and images for: Investigating the causal links between inflammatory cytokines and scoliosis through bidirectional Mendelian randomization analysis (part 1 of 3)
Source: JOR Spine. 2024 Dec 11;7(4):e70019. doi: 10.1002/jsp2.70019 (PMC11632254; doi:10.1002/jsp2.70019)

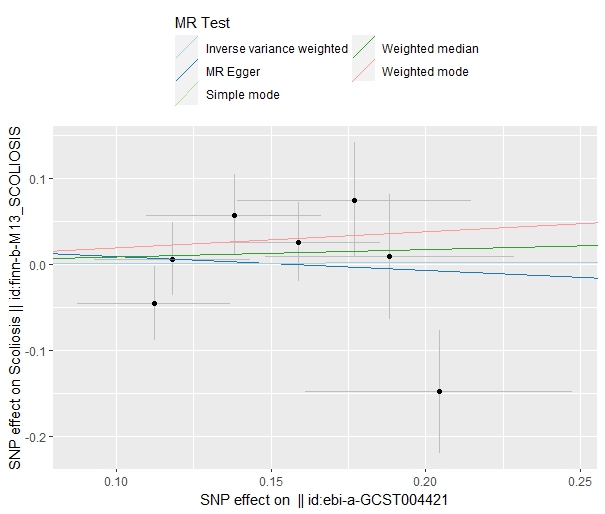

Supplement: Supplementary file 3 — Supplementary Material 3. [file JSP2-7-e70019-s004.zip › Supplementary Material 3/Exposureú║inflammatory cytokinesú1⁄4Outcomeú║Scoliosis - ╕▒▒╛/B NGF/Supplementary Material 3 B NGF 1.jpeg]

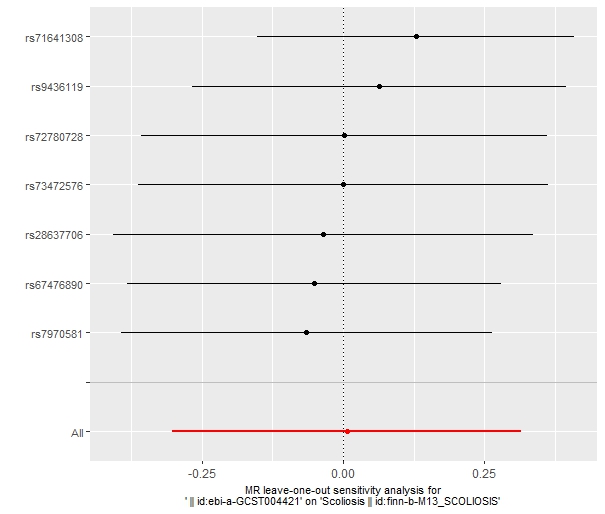

Supplement: Supplementary file 3 — Supplementary Material 3. [file JSP2-7-e70019-s004.zip › Supplementary Material 3/Exposureú║inflammatory cytokinesú1⁄4Outcomeú║Scoliosis - ╕▒▒╛/B NGF/Supplementary Material 3 B NGF 2.jpeg]

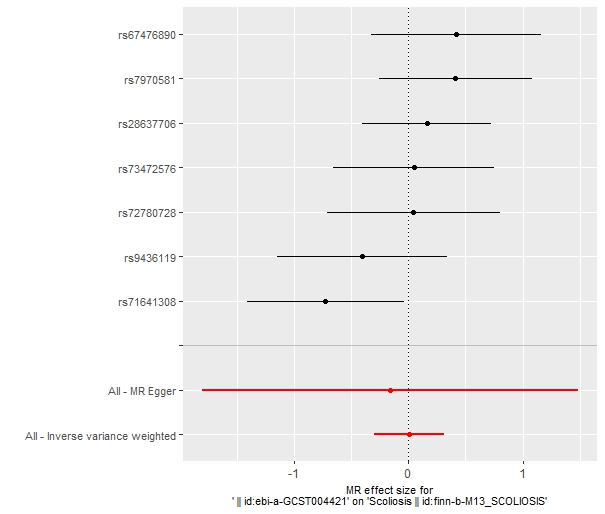

Supplement: Supplementary file 3 — Supplementary Material 3. [file JSP2-7-e70019-s004.zip › Supplementary Material 3/Exposureú║inflammatory cytokinesú1⁄4Outcomeú║Scoliosis - ╕▒▒╛/B NGF/Supplementary Material 3 B NGF 3.jpeg]

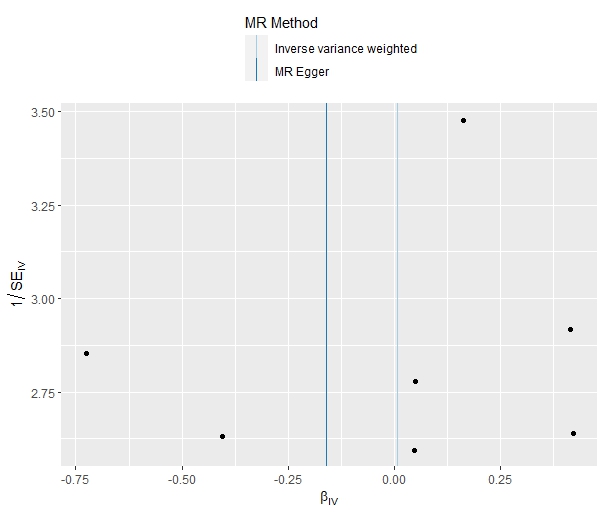

Supplement: Supplementary file 3 — Supplementary Material 3. [file JSP2-7-e70019-s004.zip › Supplementary Material 3/Exposureú║inflammatory cytokinesú1⁄4Outcomeú║Scoliosis - ╕▒▒╛/B NGF/Supplementary Material 3 B NGF 4.jpeg]

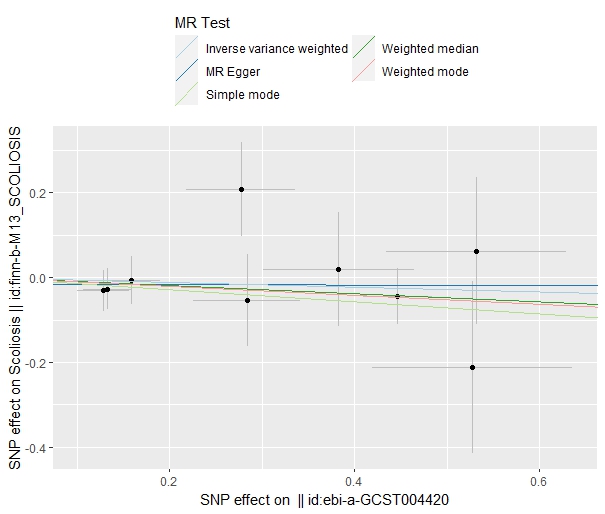

Supplement: Supplementary file 3 — Supplementary Material 3. [file JSP2-7-e70019-s004.zip › Supplementary Material 3/Exposureú║inflammatory cytokinesú1⁄4Outcomeú║Scoliosis - ╕▒▒╛/CTACK/Supplementary Material 3 CTACK 1.jpeg]

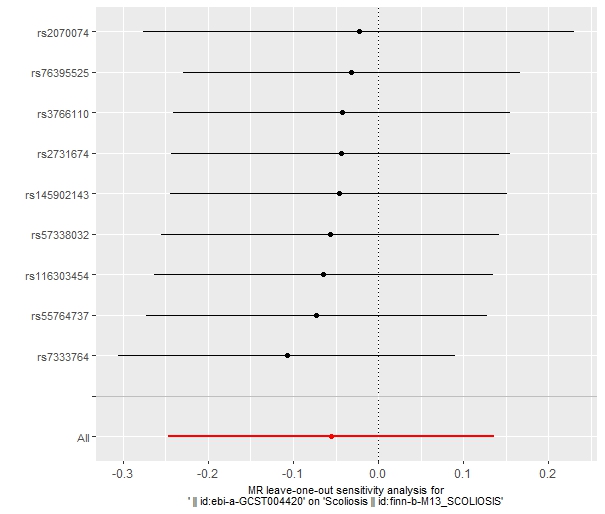

Supplement: Supplementary file 3 — Supplementary Material 3. [file JSP2-7-e70019-s004.zip › Supplementary Material 3/Exposureú║inflammatory cytokinesú1⁄4Outcomeú║Scoliosis - ╕▒▒╛/CTACK/Supplementary Material 3 CTACK 2.jpeg]

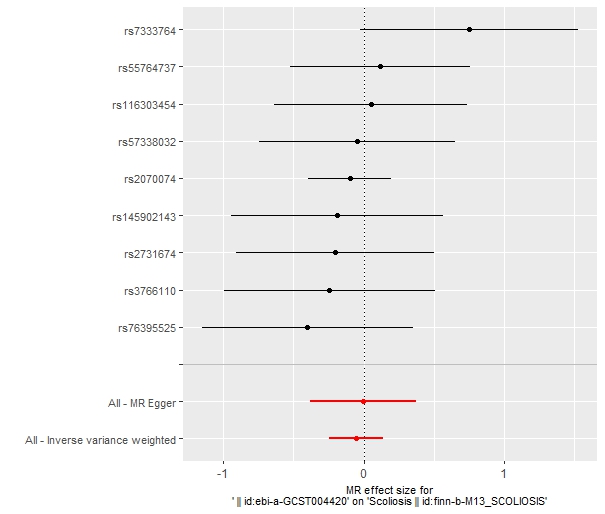

Supplement: Supplementary file 3 — Supplementary Material 3. [file JSP2-7-e70019-s004.zip › Supplementary Material 3/Exposureú║inflammatory cytokinesú1⁄4Outcomeú║Scoliosis - ╕▒▒╛/CTACK/Supplementary Material 3 CTACK 3.jpeg]

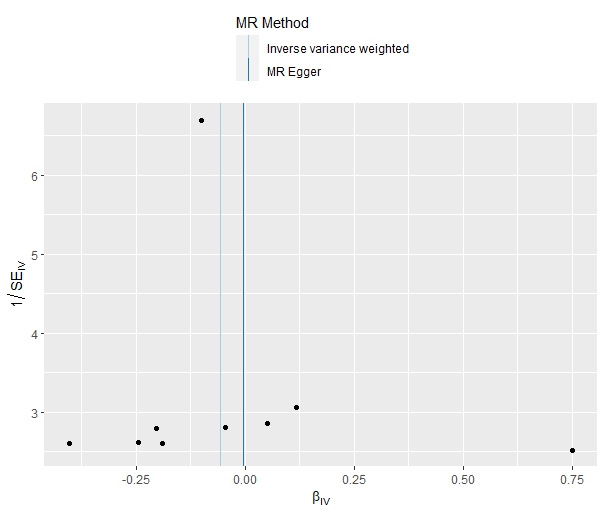

Supplement: Supplementary file 3 — Supplementary Material 3. [file JSP2-7-e70019-s004.zip › Supplementary Material 3/Exposureú║inflammatory cytokinesú1⁄4Outcomeú║Scoliosis - ╕▒▒╛/CTACK/Supplementary Material 3 CTACK 4.jpeg]

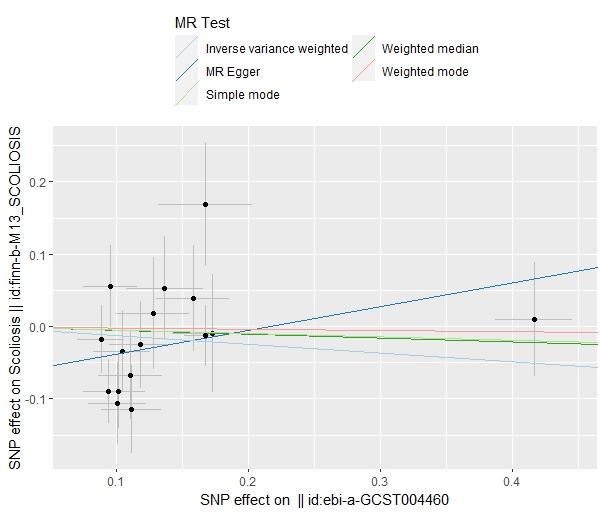

Supplement: Supplementary file 3 — Supplementary Material 3. [file JSP2-7-e70019-s004.zip › Supplementary Material 3/Exposureú║inflammatory cytokinesú1⁄4Outcomeú║Scoliosis - ╕▒▒╛/Eotaxin/Supplementary Material 3 Eotaxin 1.jpeg]

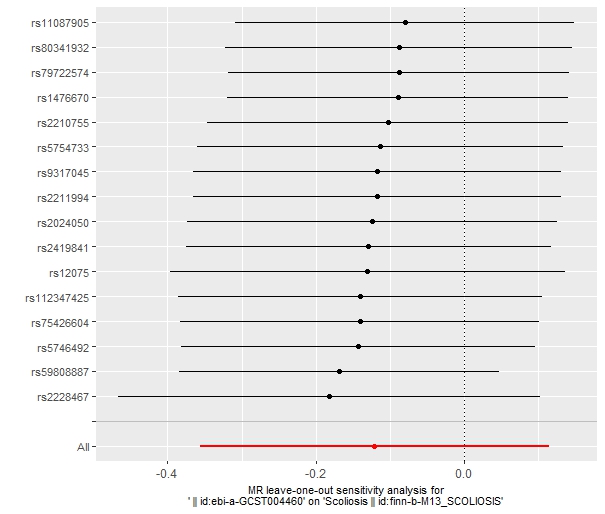

Supplement: Supplementary file 3 — Supplementary Material 3. [file JSP2-7-e70019-s004.zip › Supplementary Material 3/Exposureú║inflammatory cytokinesú1⁄4Outcomeú║Scoliosis - ╕▒▒╛/Eotaxin/Supplementary Material 3 Eotaxin 2.jpeg]

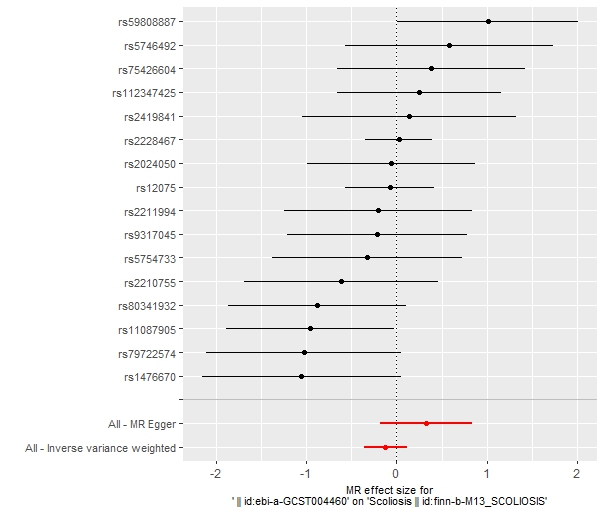

Supplement: Supplementary file 3 — Supplementary Material 3. [file JSP2-7-e70019-s004.zip › Supplementary Material 3/Exposureú║inflammatory cytokinesú1⁄4Outcomeú║Scoliosis - ╕▒▒╛/Eotaxin/Supplementary Material 3 Eotaxin 3.jpeg]

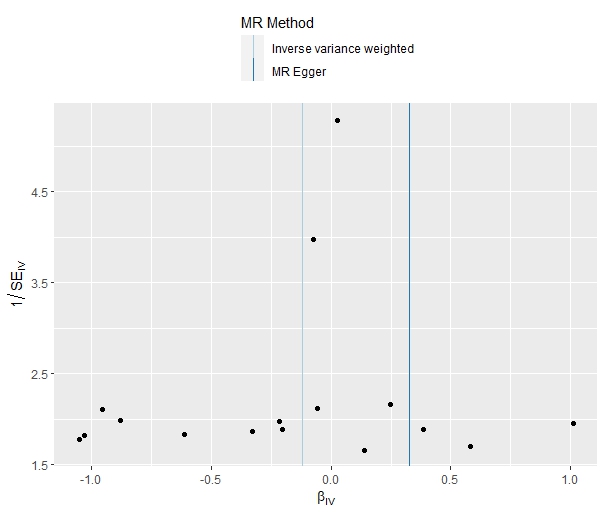

Supplement: Supplementary file 3 — Supplementary Material 3. [file JSP2-7-e70019-s004.zip › Supplementary Material 3/Exposureú║inflammatory cytokinesú1⁄4Outcomeú║Scoliosis - ╕▒▒╛/Eotaxin/Supplementary Material 3 Eotaxin 4.jpeg]

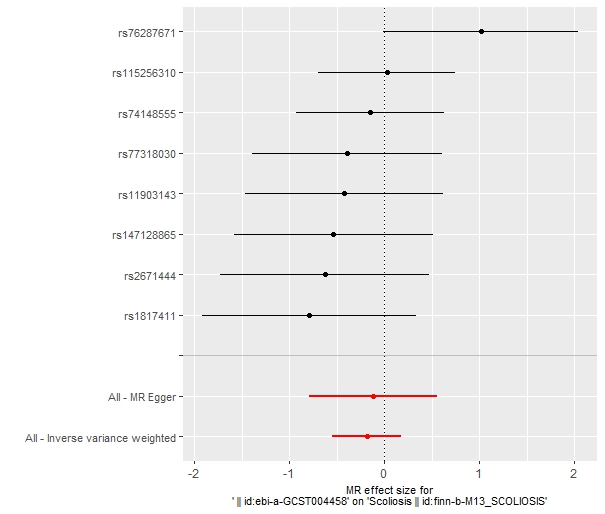

Supplement: Supplementary file 3 — Supplementary Material 3. [file JSP2-7-e70019-s004.zip › Supplementary Material 3/Exposureú║inflammatory cytokinesú1⁄4Outcomeú║Scoliosis - ╕▒▒╛/GCSF/Supplementary Material 3 GCSF 1 (2).jpeg]

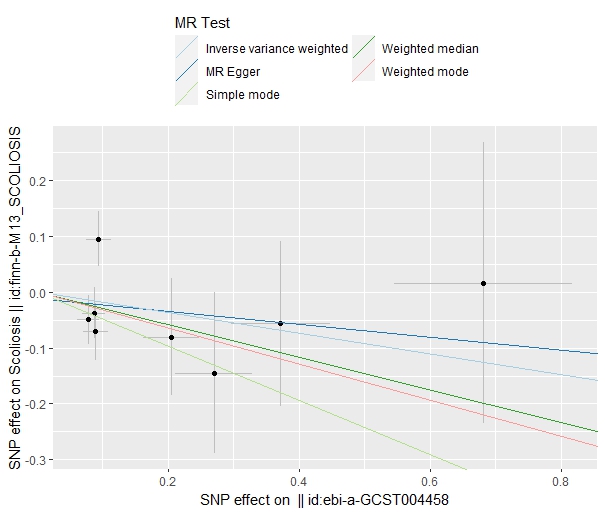

Supplement: Supplementary file 3 — Supplementary Material 3. [file JSP2-7-e70019-s004.zip › Supplementary Material 3/Exposureú║inflammatory cytokinesú1⁄4Outcomeú║Scoliosis - ╕▒▒╛/GCSF/Supplementary Material 3 GCSF 1.jpeg]

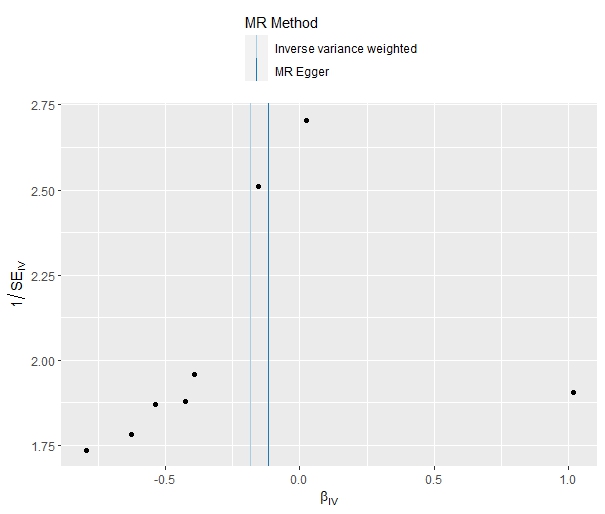

Supplement: Supplementary file 3 — Supplementary Material 3. [file JSP2-7-e70019-s004.zip › Supplementary Material 3/Exposureú║inflammatory cytokinesú1⁄4Outcomeú║Scoliosis - ╕▒▒╛/GCSF/Supplementary Material 3 GCSF 3.jpeg]

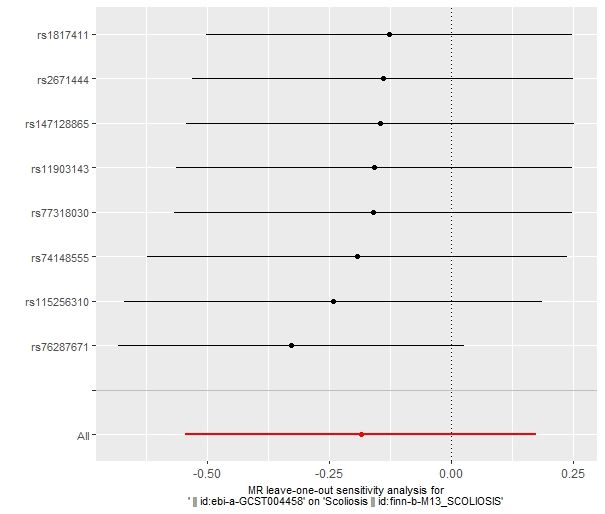

Supplement: Supplementary file 3 — Supplementary Material 3. [file JSP2-7-e70019-s004.zip › Supplementary Material 3/Exposureú║inflammatory cytokinesú1⁄4Outcomeú║Scoliosis - ╕▒▒╛/GCSF/Supplementary Material 3 GCSF 4.jpeg]

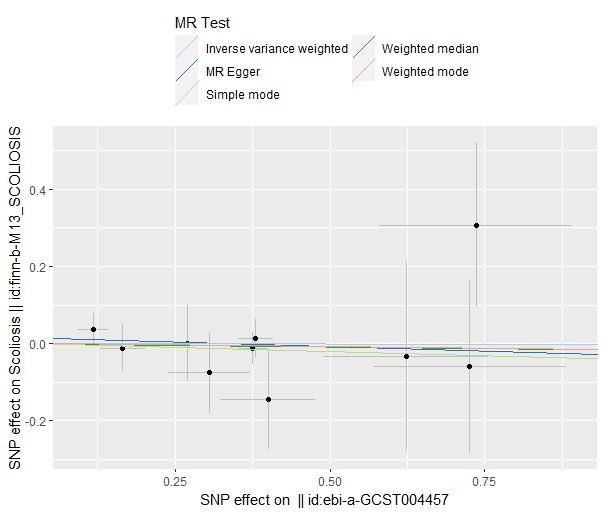

Supplement: Supplementary file 3 — Supplementary Material 3. [file JSP2-7-e70019-s004.zip › Supplementary Material 3/Exposureú║inflammatory cytokinesú1⁄4Outcomeú║Scoliosis - ╕▒▒╛/GRPa/Supplementary Material 3 GRPa 1.jpeg]

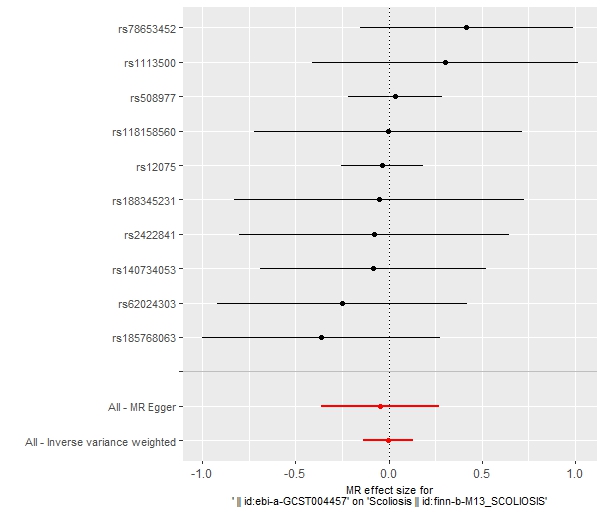

Supplement: Supplementary file 3 — Supplementary Material 3. [file JSP2-7-e70019-s004.zip › Supplementary Material 3/Exposureú║inflammatory cytokinesú1⁄4Outcomeú║Scoliosis - ╕▒▒╛/GRPa/Supplementary Material 3 GRPa 2.jpeg]

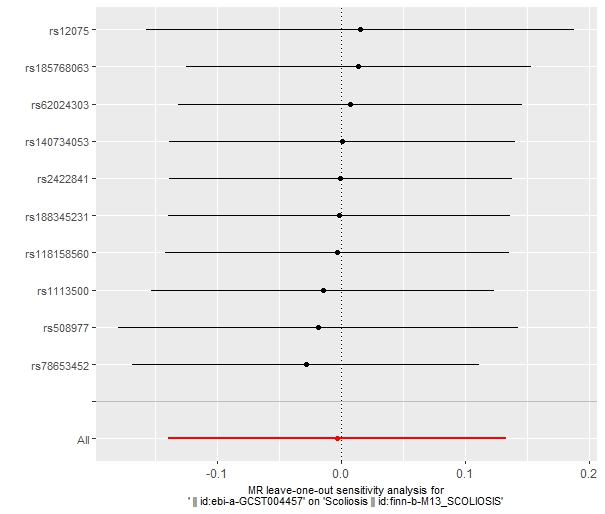

Supplement: Supplementary file 3 — Supplementary Material 3. [file JSP2-7-e70019-s004.zip › Supplementary Material 3/Exposureú║inflammatory cytokinesú1⁄4Outcomeú║Scoliosis - ╕▒▒╛/GRPa/Supplementary Material 3 GRPa 3.jpeg]

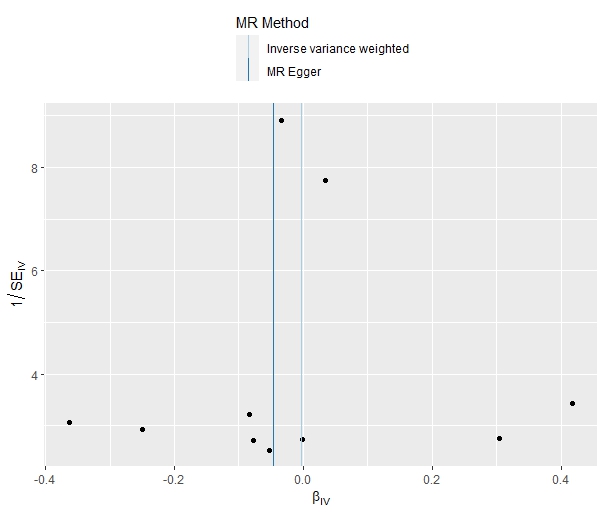

Supplement: Supplementary file 3 — Supplementary Material 3. [file JSP2-7-e70019-s004.zip › Supplementary Material 3/Exposureú║inflammatory cytokinesú1⁄4Outcomeú║Scoliosis - ╕▒▒╛/GRPa/Supplementary Material 3 GRPa 4.jpeg]

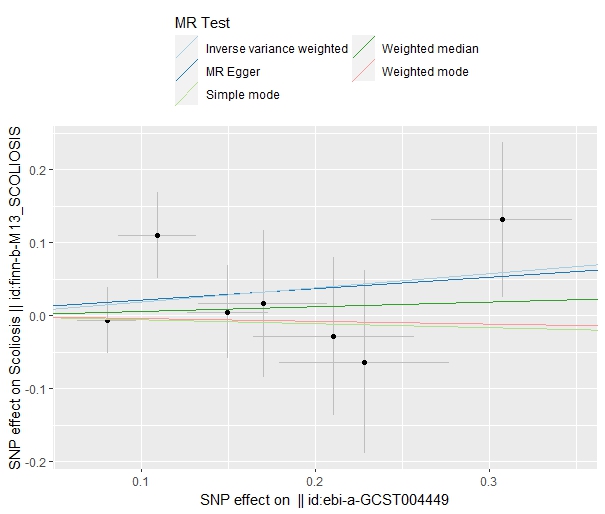

Supplement: Supplementary file 3 — Supplementary Material 3. [file JSP2-7-e70019-s004.zip › Supplementary Material 3/Exposureú║inflammatory cytokinesú1⁄4Outcomeú║Scoliosis - ╕▒▒╛/HGF/Supplementary Material 3 HGF 1.jpeg]

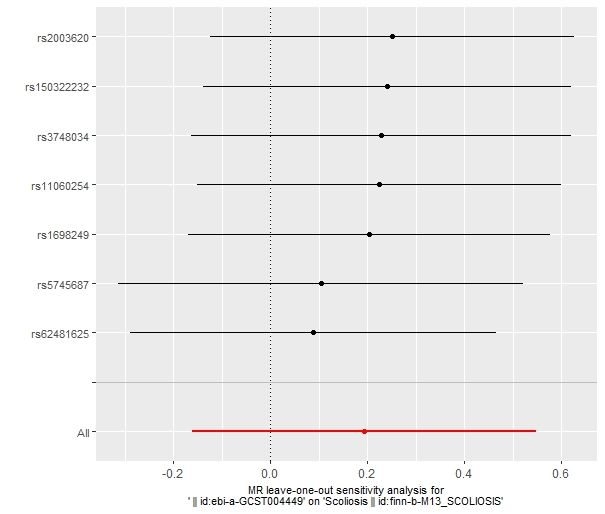

Supplement: Supplementary file 3 — Supplementary Material 3. [file JSP2-7-e70019-s004.zip › Supplementary Material 3/Exposureú║inflammatory cytokinesú1⁄4Outcomeú║Scoliosis - ╕▒▒╛/HGF/Supplementary Material 3 HGF 2.jpeg]

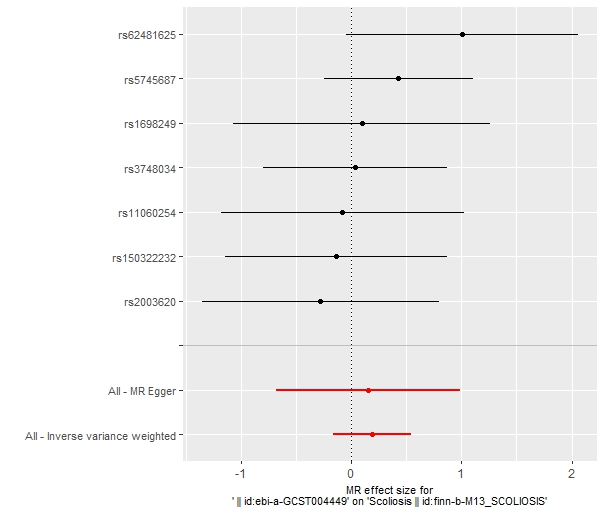

Supplement: Supplementary file 3 — Supplementary Material 3. [file JSP2-7-e70019-s004.zip › Supplementary Material 3/Exposureú║inflammatory cytokinesú1⁄4Outcomeú║Scoliosis - ╕▒▒╛/HGF/Supplementary Material 3 HGF 3.jpeg]

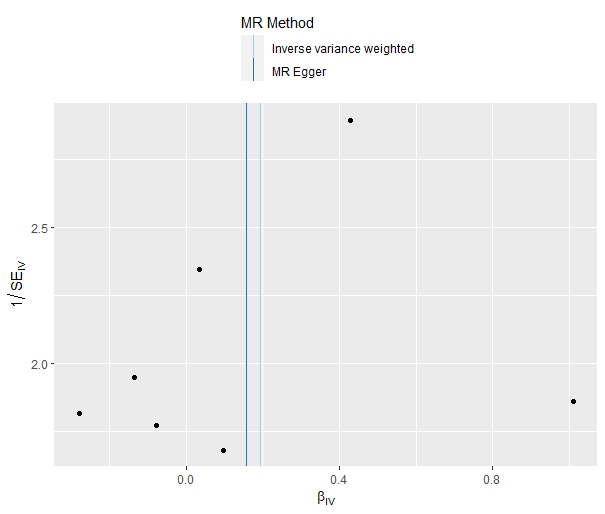

Supplement: Supplementary file 3 — Supplementary Material 3. [file JSP2-7-e70019-s004.zip › Supplementary Material 3/Exposureú║inflammatory cytokinesú1⁄4Outcomeú║Scoliosis - ╕▒▒╛/HGF/Supplementary Material 3 HGF 4.jpeg]

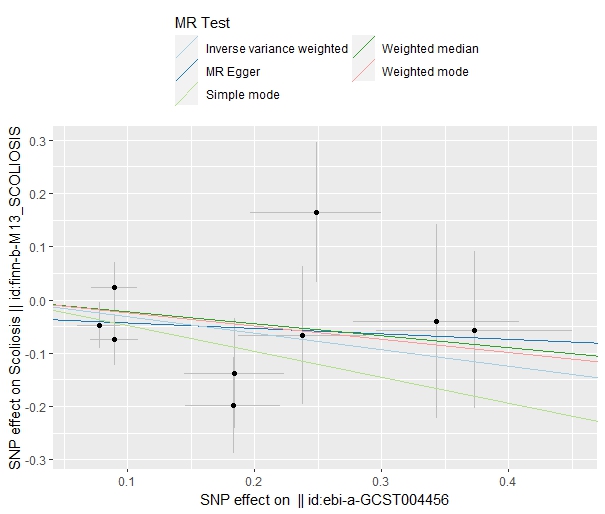

Supplement: Supplementary file 3 — Supplementary Material 3. [file JSP2-7-e70019-s004.zip › Supplementary Material 3/Exposureú║inflammatory cytokinesú1⁄4Outcomeú║Scoliosis - ╕▒▒╛/IFN Y/Supplementary Material 3 IFN Y 1.jpeg]

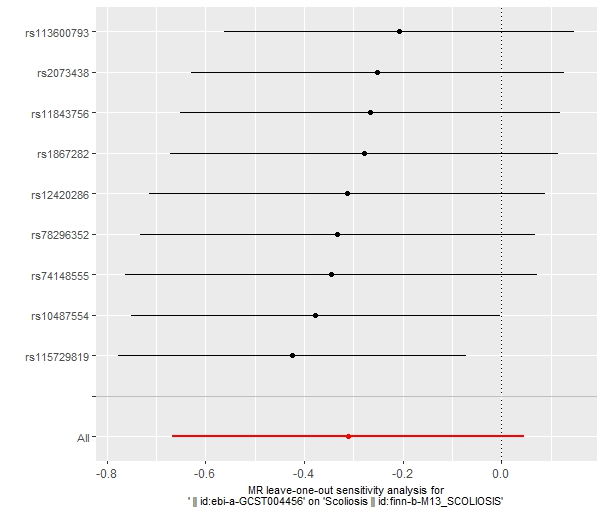

Supplement: Supplementary file 3 — Supplementary Material 3. [file JSP2-7-e70019-s004.zip › Supplementary Material 3/Exposureú║inflammatory cytokinesú1⁄4Outcomeú║Scoliosis - ╕▒▒╛/IFN Y/Supplementary Material 3 IFN Y 2.jpeg]

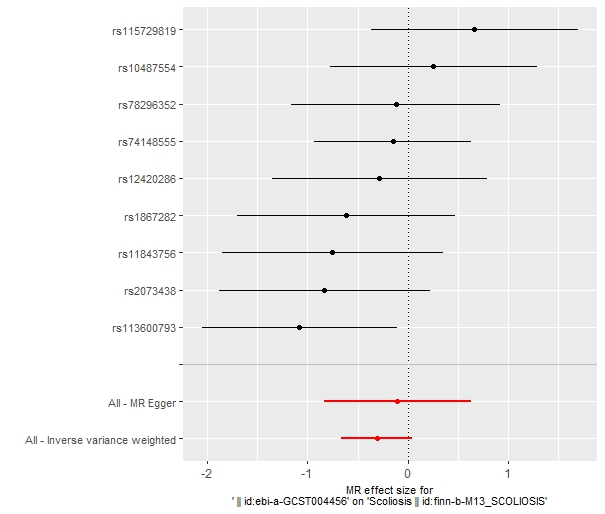

Supplement: Supplementary file 3 — Supplementary Material 3. [file JSP2-7-e70019-s004.zip › Supplementary Material 3/Exposureú║inflammatory cytokinesú1⁄4Outcomeú║Scoliosis - ╕▒▒╛/IFN Y/Supplementary Material 3 IFN Y 3.jpeg]

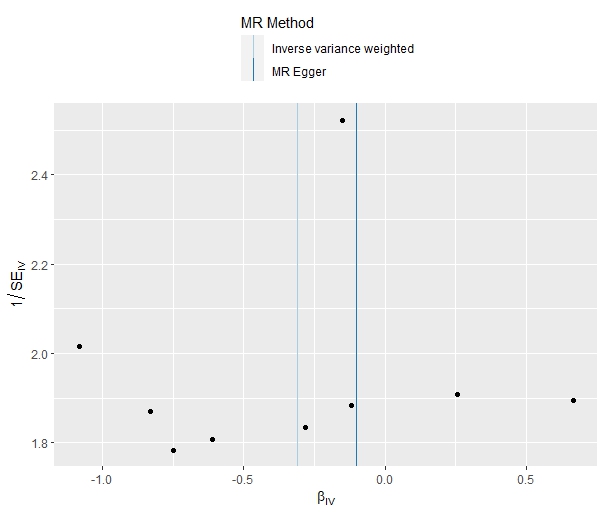

Supplement: Supplementary file 3 — Supplementary Material 3. [file JSP2-7-e70019-s004.zip › Supplementary Material 3/Exposureú║inflammatory cytokinesú1⁄4Outcomeú║Scoliosis - ╕▒▒╛/IFN Y/Supplementary Material 3 IFN Y 4.jpeg]

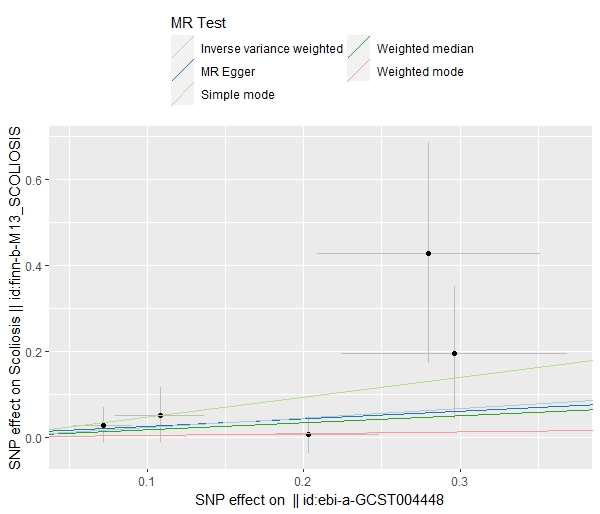

Supplement: Supplementary file 3 — Supplementary Material 3. [file JSP2-7-e70019-s004.zip › Supplementary Material 3/Exposureú║inflammatory cytokinesú1⁄4Outcomeú║Scoliosis - ╕▒▒╛/IL1 B/Supplementary Material 3 Supplementary Material 3 IFN Y 1.jpeg]

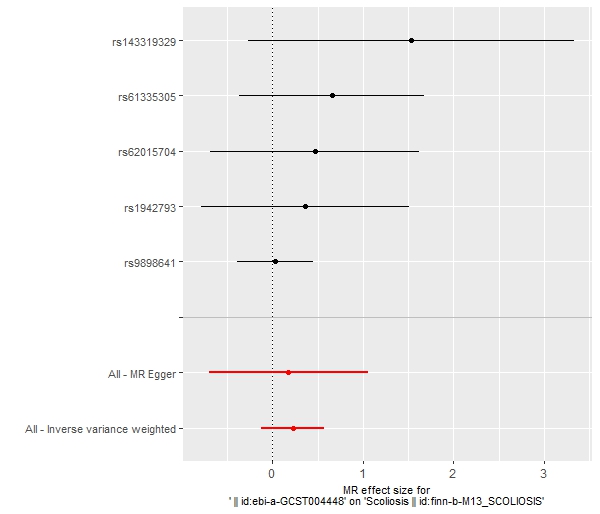

Supplement: Supplementary file 3 — Supplementary Material 3. [file JSP2-7-e70019-s004.zip › Supplementary Material 3/Exposureú║inflammatory cytokinesú1⁄4Outcomeú║Scoliosis - ╕▒▒╛/IL1 B/Supplementary Material 3 Supplementary Material 3 IFN Y 2.jpeg]

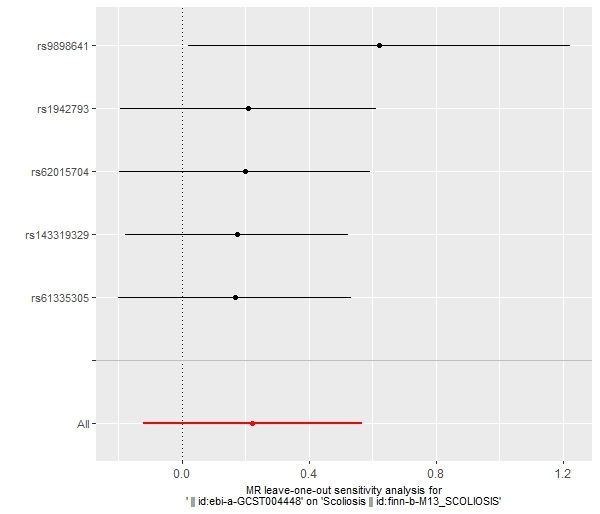

Supplement: Supplementary file 3 — Supplementary Material 3. [file JSP2-7-e70019-s004.zip › Supplementary Material 3/Exposureú║inflammatory cytokinesú1⁄4Outcomeú║Scoliosis - ╕▒▒╛/IL1 B/Supplementary Material 3 Supplementary Material 3 IFN Y 3.jpeg]

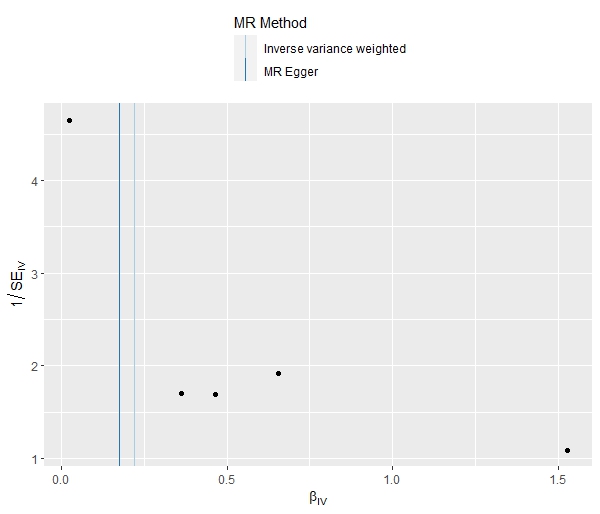

Supplement: Supplementary file 3 — Supplementary Material 3. [file JSP2-7-e70019-s004.zip › Supplementary Material 3/Exposureú║inflammatory cytokinesú1⁄4Outcomeú║Scoliosis - ╕▒▒╛/IL1 B/Supplementary Material 3 Supplementary Material 3 IFN Y 4.jpeg]

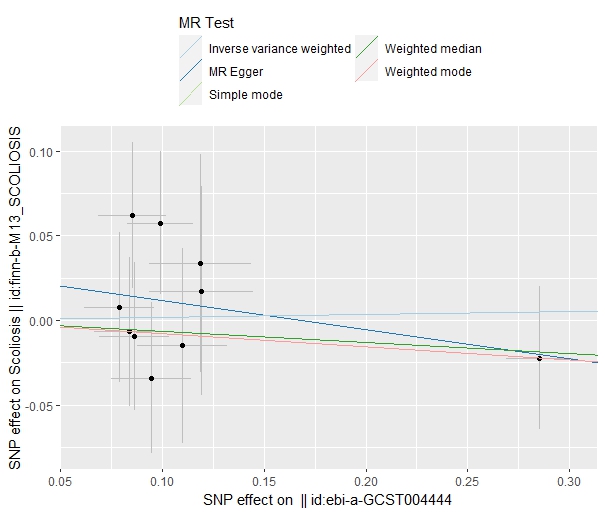

Supplement: Supplementary file 3 — Supplementary Material 3. [file JSP2-7-e70019-s004.zip › Supplementary Material 3/Exposureú║inflammatory cytokinesú1⁄4Outcomeú║Scoliosis - ╕▒▒╛/IL10/Supplementary Material 3 IL10 1.jpeg]

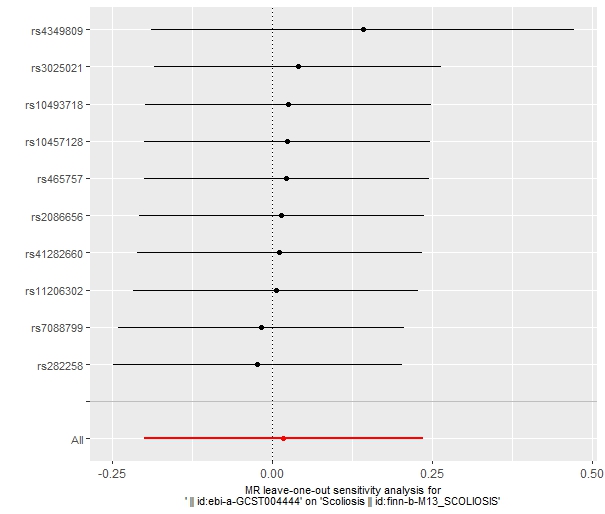

Supplement: Supplementary file 3 — Supplementary Material 3. [file JSP2-7-e70019-s004.zip › Supplementary Material 3/Exposureú║inflammatory cytokinesú1⁄4Outcomeú║Scoliosis - ╕▒▒╛/IL10/Supplementary Material 3 IL10 2.jpeg]

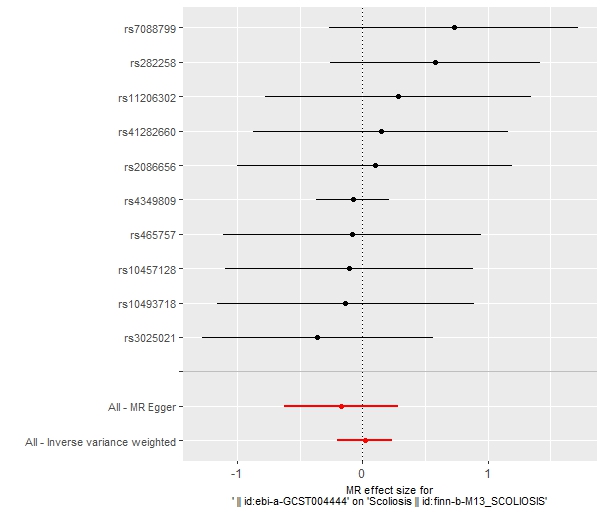

Supplement: Supplementary file 3 — Supplementary Material 3. [file JSP2-7-e70019-s004.zip › Supplementary Material 3/Exposureú║inflammatory cytokinesú1⁄4Outcomeú║Scoliosis - ╕▒▒╛/IL10/Supplementary Material 3 IL10 3.jpeg]

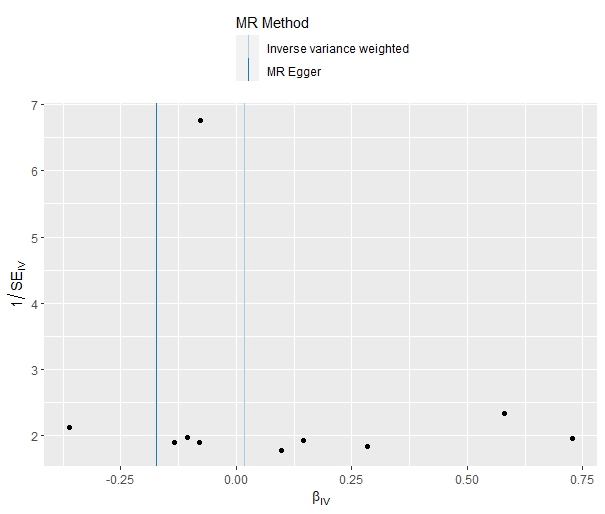

Supplement: Supplementary file 3 — Supplementary Material 3. [file JSP2-7-e70019-s004.zip › Supplementary Material 3/Exposureú║inflammatory cytokinesú1⁄4Outcomeú║Scoliosis - ╕▒▒╛/IL10/Supplementary Material 3 IL10 4.jpeg]

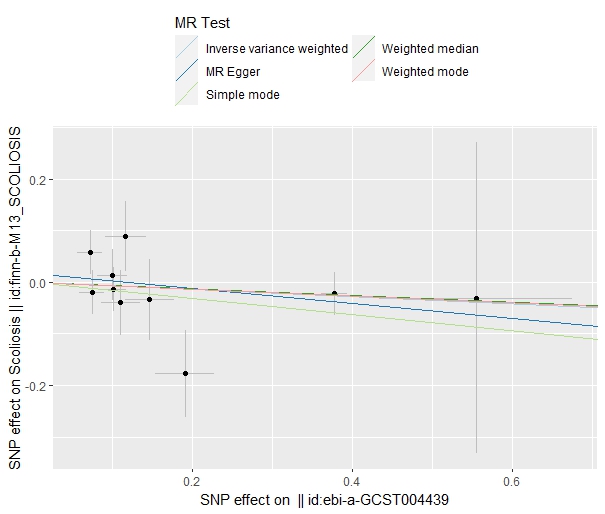

Supplement: Supplementary file 3 — Supplementary Material 3. [file JSP2-7-e70019-s004.zip › Supplementary Material 3/Exposureú║inflammatory cytokinesú1⁄4Outcomeú║Scoliosis - ╕▒▒╛/IL12p70/Supplementary Material 3 IL12p70 1.jpeg]

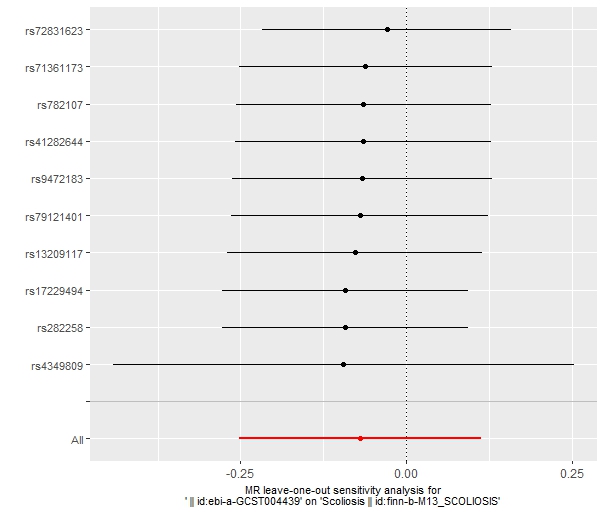

Supplement: Supplementary file 3 — Supplementary Material 3. [file JSP2-7-e70019-s004.zip › Supplementary Material 3/Exposureú║inflammatory cytokinesú1⁄4Outcomeú║Scoliosis - ╕▒▒╛/IL12p70/Supplementary Material 3 IL12p70 2.jpeg]

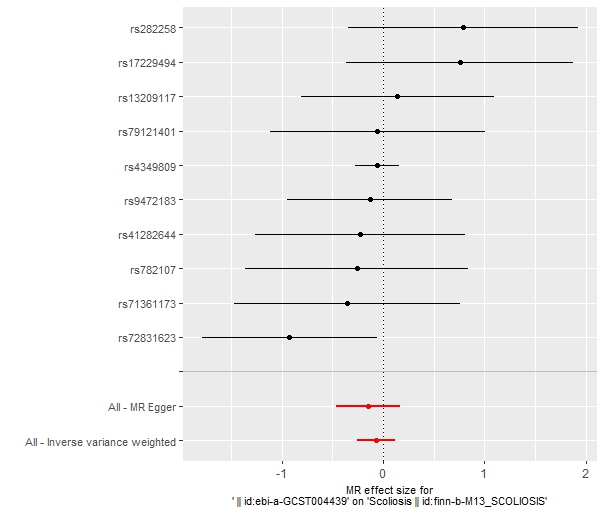

Supplement: Supplementary file 3 — Supplementary Material 3. [file JSP2-7-e70019-s004.zip › Supplementary Material 3/Exposureú║inflammatory cytokinesú1⁄4Outcomeú║Scoliosis - ╕▒▒╛/IL12p70/Supplementary Material 3 IL12p70 3.jpeg]

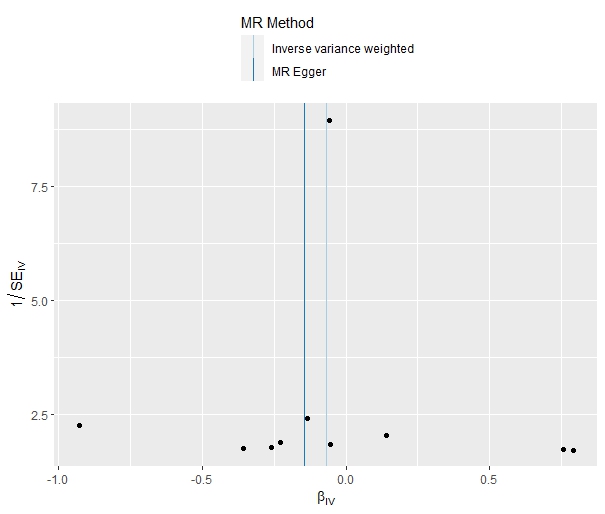

Supplement: Supplementary file 3 — Supplementary Material 3. [file JSP2-7-e70019-s004.zip › Supplementary Material 3/Exposureú║inflammatory cytokinesú1⁄4Outcomeú║Scoliosis - ╕▒▒╛/IL12p70/Supplementary Material 3 IL12p70 4.jpeg]

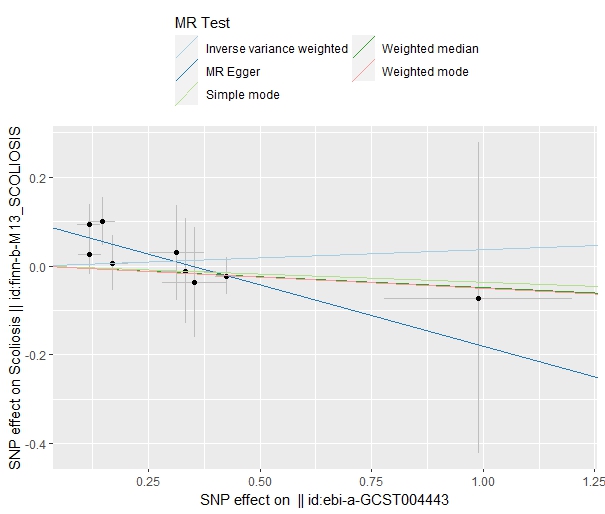

Supplement: Supplementary file 3 — Supplementary Material 3. [file JSP2-7-e70019-s004.zip › Supplementary Material 3/Exposureú║inflammatory cytokinesú1⁄4Outcomeú║Scoliosis - ╕▒▒╛/IL13/Supplementary Material 3 IL13 1.jpeg]

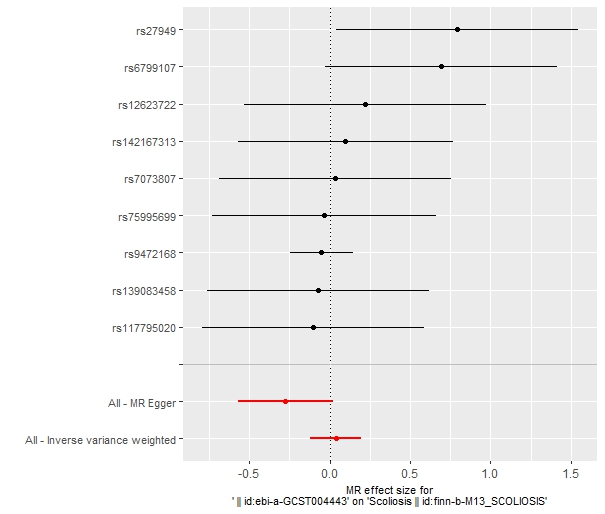

Supplement: Supplementary file 3 — Supplementary Material 3. [file JSP2-7-e70019-s004.zip › Supplementary Material 3/Exposureú║inflammatory cytokinesú1⁄4Outcomeú║Scoliosis - ╕▒▒╛/IL13/Supplementary Material 3 IL13 2.jpeg]

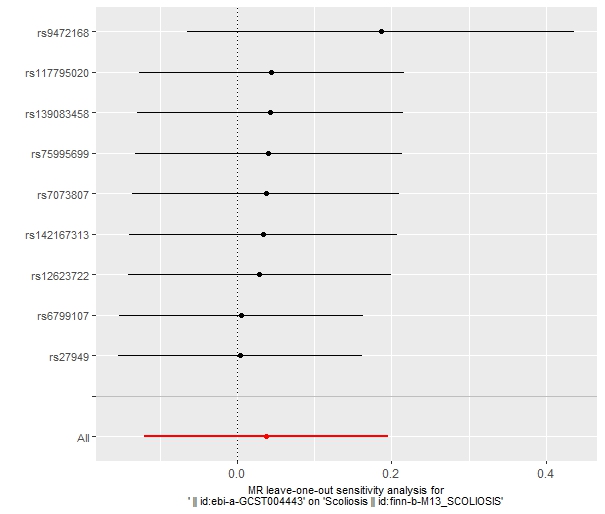

Supplement: Supplementary file 3 — Supplementary Material 3. [file JSP2-7-e70019-s004.zip › Supplementary Material 3/Exposureú║inflammatory cytokinesú1⁄4Outcomeú║Scoliosis - ╕▒▒╛/IL13/Supplementary Material 3 IL13 3.jpeg]

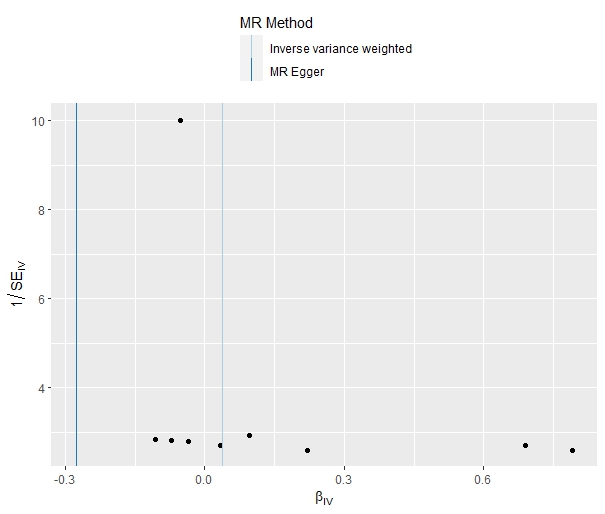

Supplement: Supplementary file 3 — Supplementary Material 3. [file JSP2-7-e70019-s004.zip › Supplementary Material 3/Exposureú║inflammatory cytokinesú1⁄4Outcomeú║Scoliosis - ╕▒▒╛/IL13/Supplementary Material 3 IL13 4.jpeg]

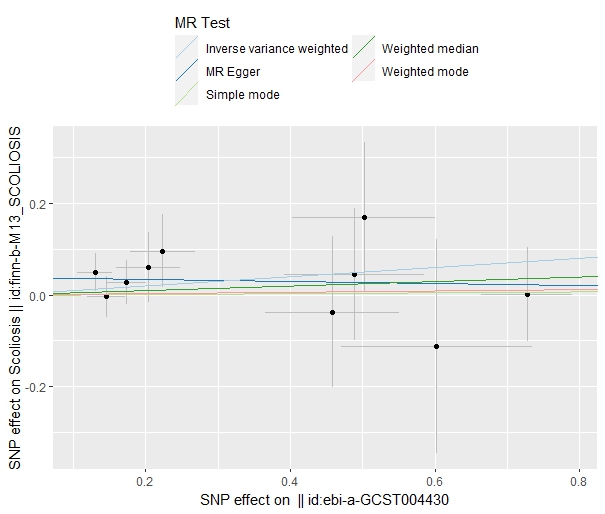

Supplement: Supplementary file 3 — Supplementary Material 3. [file JSP2-7-e70019-s004.zip › Supplementary Material 3/Exposureú║inflammatory cytokinesú1⁄4Outcomeú║Scoliosis - ╕▒▒╛/IL16/Supplementary Material 3 IL16 1.jpeg]

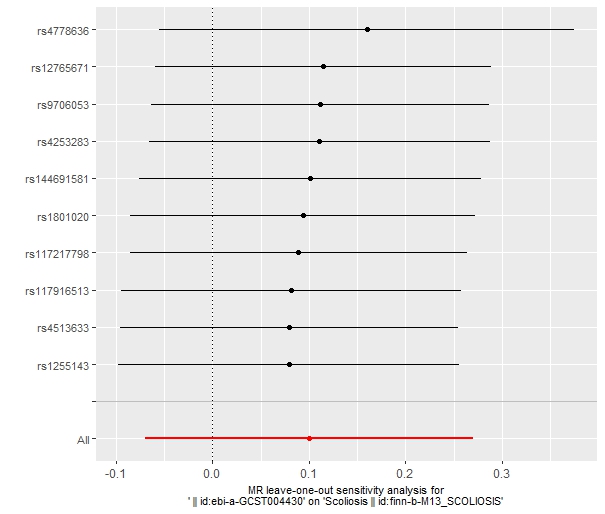

Supplement: Supplementary file 3 — Supplementary Material 3. [file JSP2-7-e70019-s004.zip › Supplementary Material 3/Exposureú║inflammatory cytokinesú1⁄4Outcomeú║Scoliosis - ╕▒▒╛/IL16/Supplementary Material 3 IL16 2.jpeg]

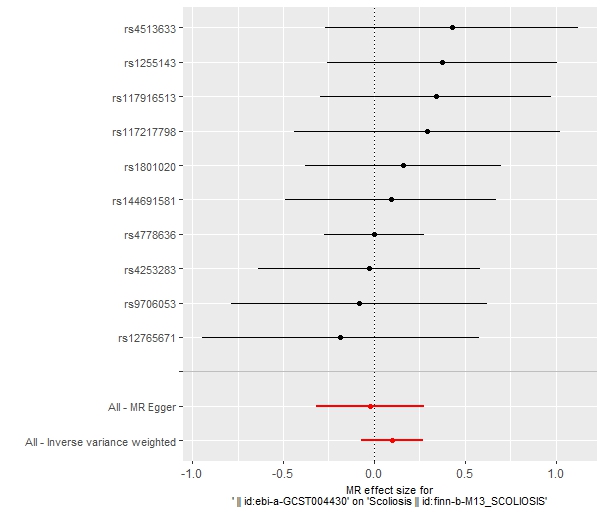

Supplement: Supplementary file 3 — Supplementary Material 3. [file JSP2-7-e70019-s004.zip › Supplementary Material 3/Exposureú║inflammatory cytokinesú1⁄4Outcomeú║Scoliosis - ╕▒▒╛/IL16/Supplementary Material 3 IL16 3.jpeg]

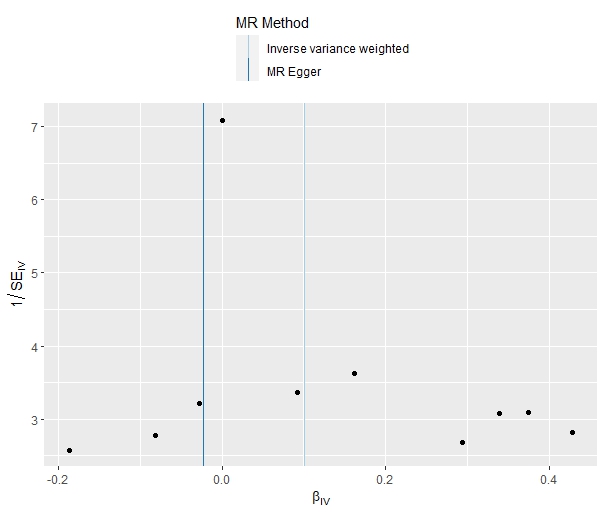

Supplement: Supplementary file 3 — Supplementary Material 3. [file JSP2-7-e70019-s004.zip › Supplementary Material 3/Exposureú║inflammatory cytokinesú1⁄4Outcomeú║Scoliosis - ╕▒▒╛/IL16/Supplementary Material 3 IL16 4.jpeg]

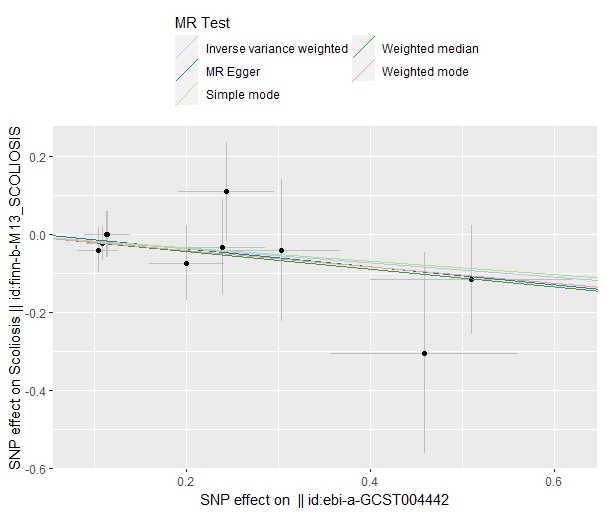

Supplement: Supplementary file 3 — Supplementary Material 3. [file JSP2-7-e70019-s004.zip › Supplementary Material 3/Exposureú║inflammatory cytokinesú1⁄4Outcomeú║Scoliosis - ╕▒▒╛/IL17/Supplementary Material 3 IL17 1.jpeg]

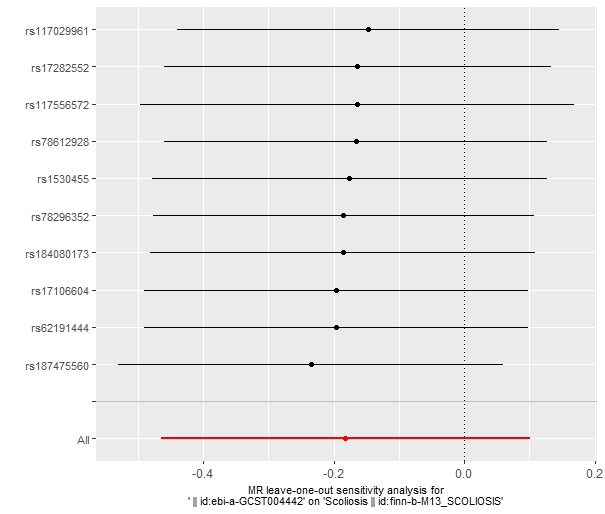

Supplement: Supplementary file 3 — Supplementary Material 3. [file JSP2-7-e70019-s004.zip › Supplementary Material 3/Exposureú║inflammatory cytokinesú1⁄4Outcomeú║Scoliosis - ╕▒▒╛/IL17/Supplementary Material 3 IL17 2.jpeg]

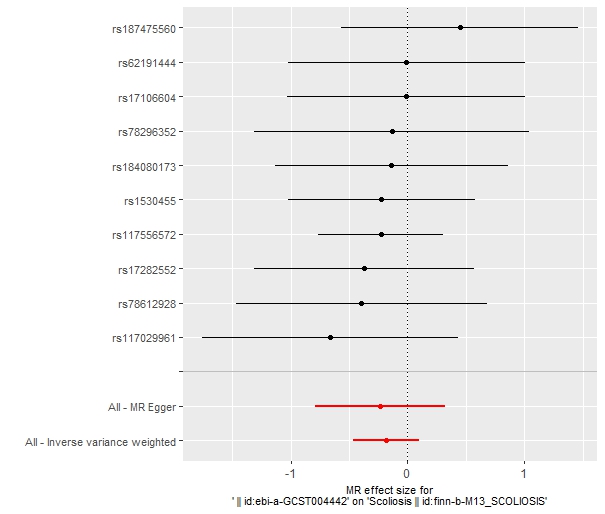

Supplement: Supplementary file 3 — Supplementary Material 3. [file JSP2-7-e70019-s004.zip › Supplementary Material 3/Exposureú║inflammatory cytokinesú1⁄4Outcomeú║Scoliosis - ╕▒▒╛/IL17/Supplementary Material 3 IL17 3.jpeg]

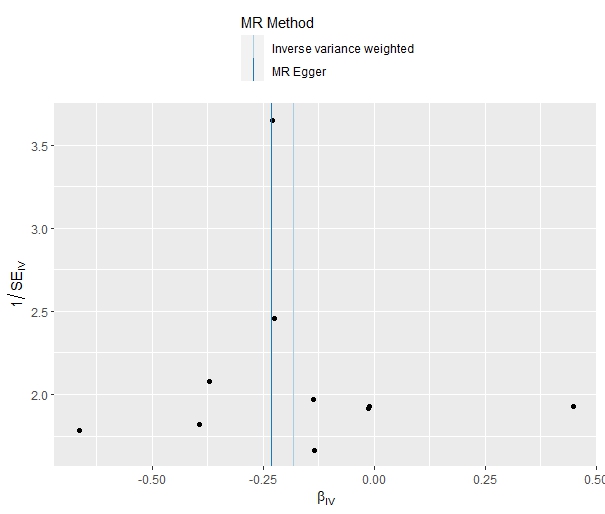

Supplement: Supplementary file 3 — Supplementary Material 3. [file JSP2-7-e70019-s004.zip › Supplementary Material 3/Exposureú║inflammatory cytokinesú1⁄4Outcomeú║Scoliosis - ╕▒▒╛/IL17/Supplementary Material 3 IL17 4.jpeg]

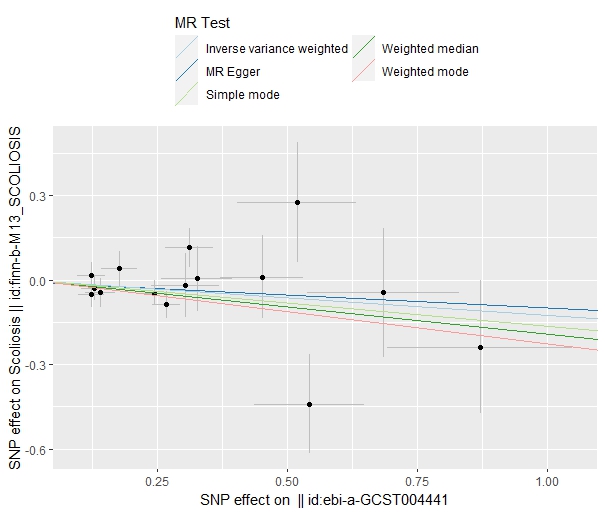

Supplement: Supplementary file 3 — Supplementary Material 3. [file JSP2-7-e70019-s004.zip › Supplementary Material 3/Exposureú║inflammatory cytokinesú1⁄4Outcomeú║Scoliosis - ╕▒▒╛/IL18/Supplementary Material 3 IL18 1.jpeg]

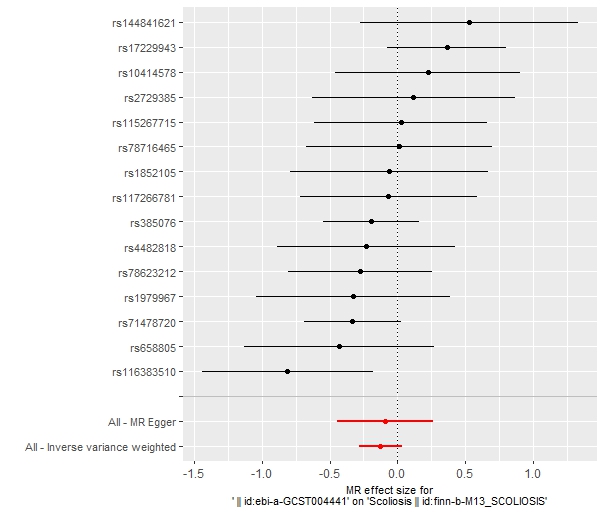

Supplement: Supplementary file 3 — Supplementary Material 3. [file JSP2-7-e70019-s004.zip › Supplementary Material 3/Exposureú║inflammatory cytokinesú1⁄4Outcomeú║Scoliosis - ╕▒▒╛/IL18/Supplementary Material 3 IL18 2.jpeg]

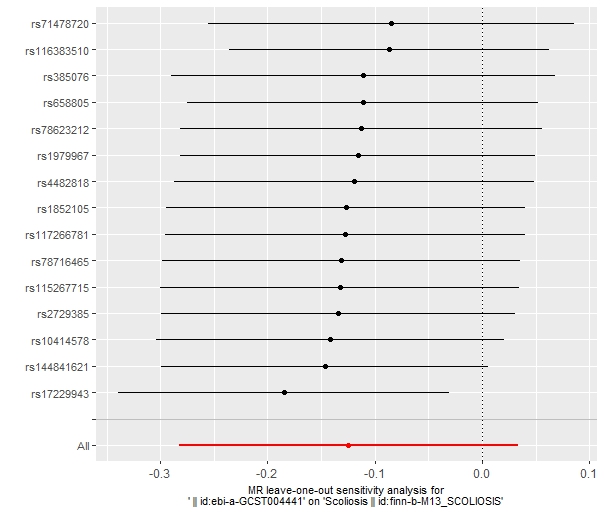

Supplement: Supplementary file 3 — Supplementary Material 3. [file JSP2-7-e70019-s004.zip › Supplementary Material 3/Exposureú║inflammatory cytokinesú1⁄4Outcomeú║Scoliosis - ╕▒▒╛/IL18/Supplementary Material 3 IL18 3.jpeg]

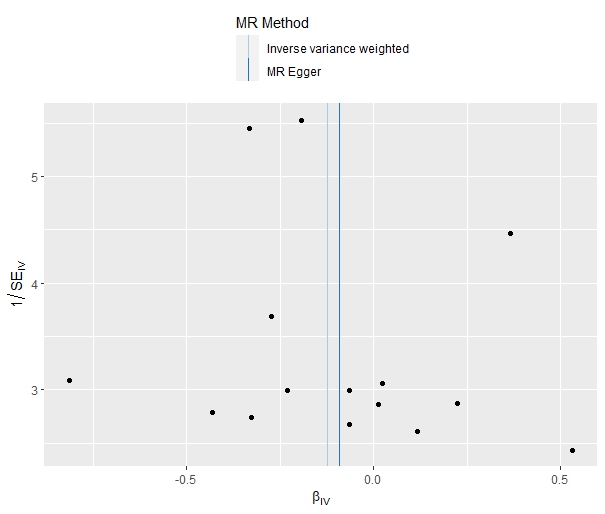

Supplement: Supplementary file 3 — Supplementary Material 3. [file JSP2-7-e70019-s004.zip › Supplementary Material 3/Exposureú║inflammatory cytokinesú1⁄4Outcomeú║Scoliosis - ╕▒▒╛/IL18/Supplementary Material 3 IL18 4.jpeg]

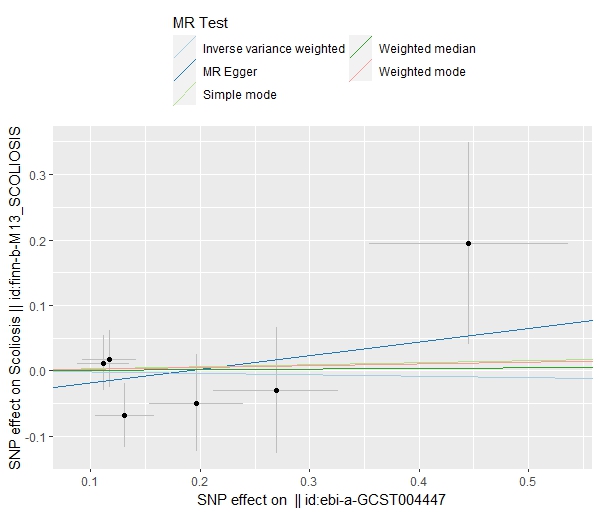

Supplement: Supplementary file 3 — Supplementary Material 3. [file JSP2-7-e70019-s004.zip › Supplementary Material 3/Exposureú║inflammatory cytokinesú1⁄4Outcomeú║Scoliosis - ╕▒▒╛/IL1RA/Supplementary Material 3 IL1RA 1.jpeg]

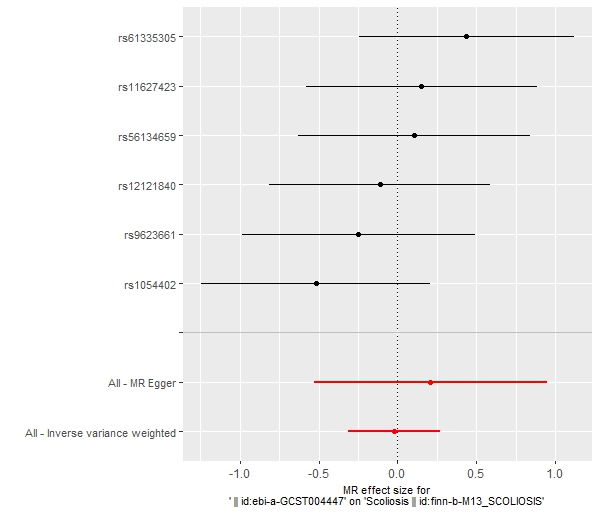

Supplement: Supplementary file 3 — Supplementary Material 3. [file JSP2-7-e70019-s004.zip › Supplementary Material 3/Exposureú║inflammatory cytokinesú1⁄4Outcomeú║Scoliosis - ╕▒▒╛/IL1RA/Supplementary Material 3 IL1RA 2.jpeg]

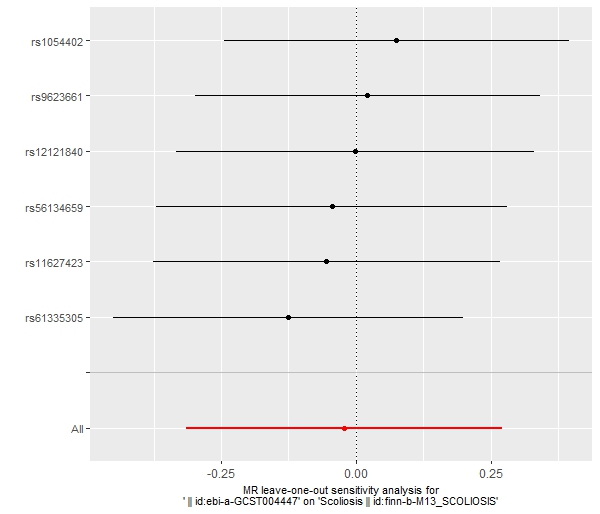

Supplement: Supplementary file 3 — Supplementary Material 3. [file JSP2-7-e70019-s004.zip › Supplementary Material 3/Exposureú║inflammatory cytokinesú1⁄4Outcomeú║Scoliosis - ╕▒▒╛/IL1RA/Supplementary Material 3 IL1RA 3.jpeg]

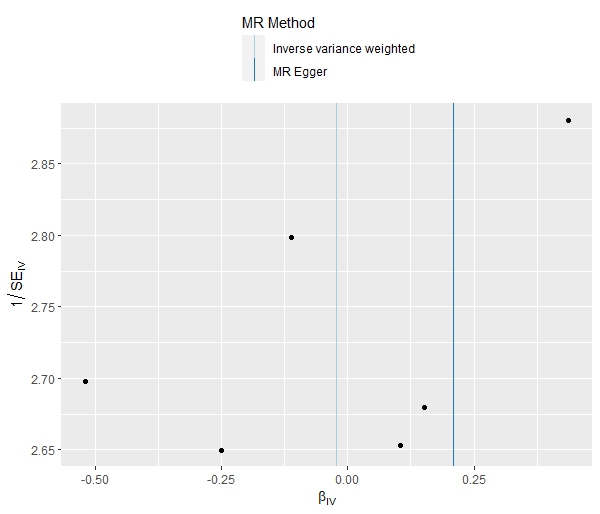

Supplement: Supplementary file 3 — Supplementary Material 3. [file JSP2-7-e70019-s004.zip › Supplementary Material 3/Exposureú║inflammatory cytokinesú1⁄4Outcomeú║Scoliosis - ╕▒▒╛/IL1RA/Supplementary Material 3 IL1RA 4.jpeg]

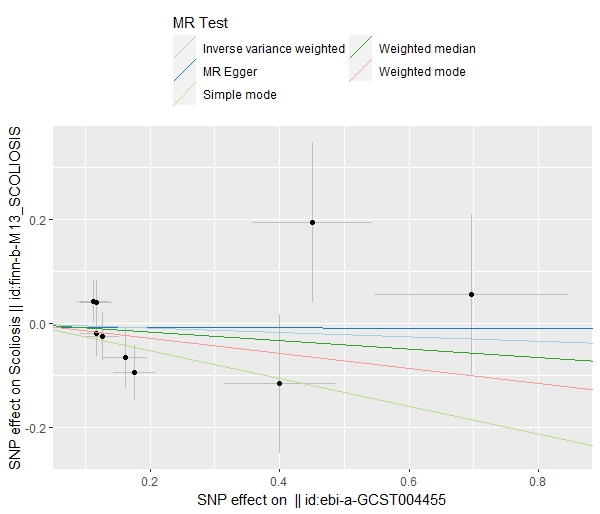

Supplement: Supplementary file 3 — Supplementary Material 3. [file JSP2-7-e70019-s004.zip › Supplementary Material 3/Exposureú║inflammatory cytokinesú1⁄4Outcomeú║Scoliosis - ╕▒▒╛/IL2/Supplementary Material 3 IL2 1.jpeg]

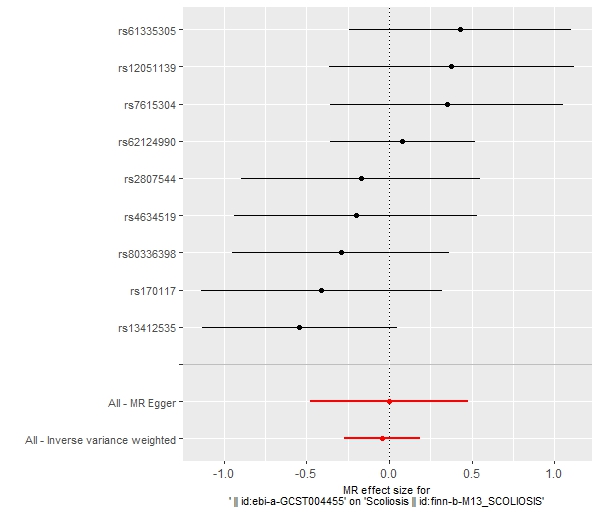

Supplement: Supplementary file 3 — Supplementary Material 3. [file JSP2-7-e70019-s004.zip › Supplementary Material 3/Exposureú║inflammatory cytokinesú1⁄4Outcomeú║Scoliosis - ╕▒▒╛/IL2/Supplementary Material 3 IL2 2.jpeg]

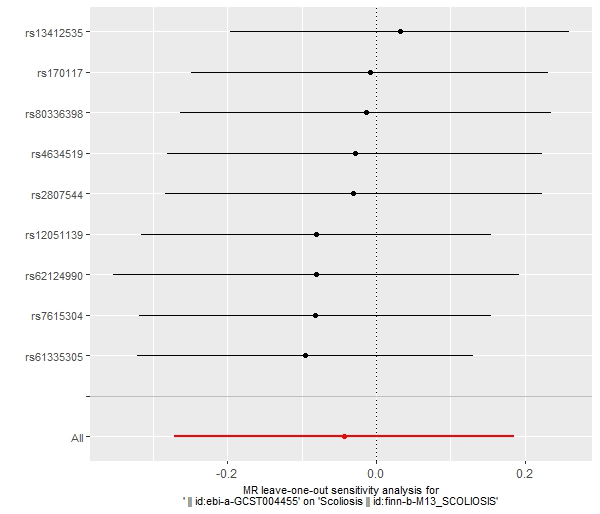

Supplement: Supplementary file 3 — Supplementary Material 3. [file JSP2-7-e70019-s004.zip › Supplementary Material 3/Exposureú║inflammatory cytokinesú1⁄4Outcomeú║Scoliosis - ╕▒▒╛/IL2/Supplementary Material 3 IL2 3.jpeg]

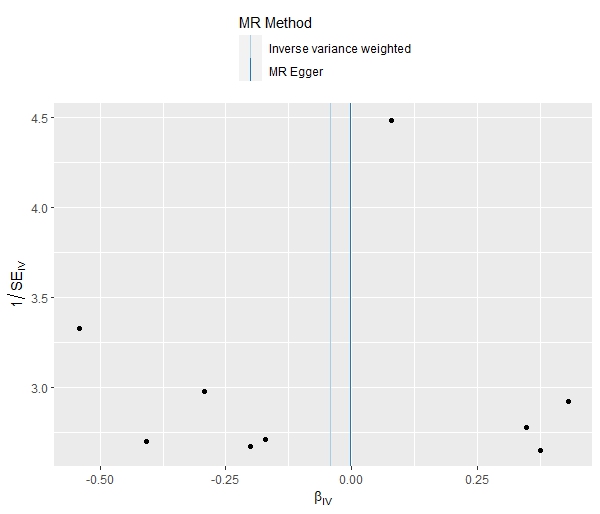

Supplement: Supplementary file 3 — Supplementary Material 3. [file JSP2-7-e70019-s004.zip › Supplementary Material 3/Exposureú║inflammatory cytokinesú1⁄4Outcomeú║Scoliosis - ╕▒▒╛/IL2/Supplementary Material 3 IL2 4.jpeg]

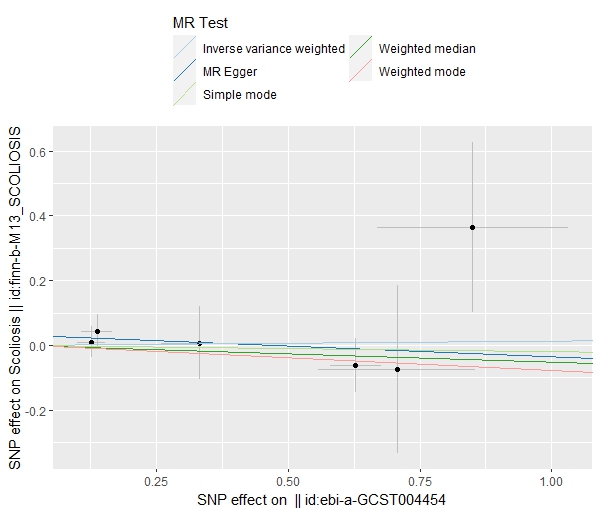

Supplement: Supplementary file 3 — Supplementary Material 3. [file JSP2-7-e70019-s004.zip › Supplementary Material 3/Exposureú║inflammatory cytokinesú1⁄4Outcomeú║Scoliosis - ╕▒▒╛/IL2RA/Supplementary Material 3 IL2RA 1.jpeg]

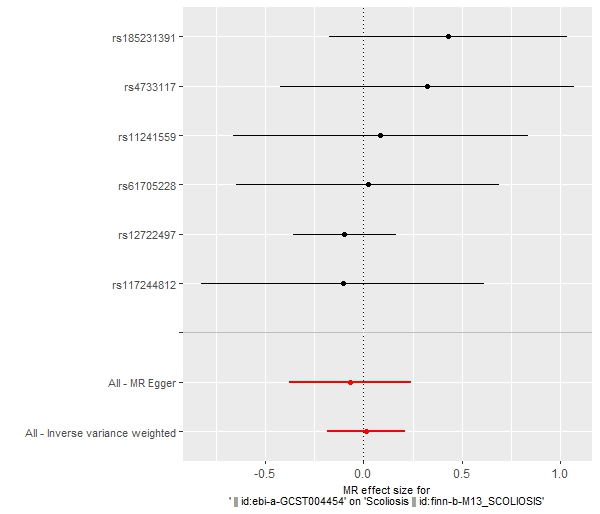

Supplement: Supplementary file 3 — Supplementary Material 3. [file JSP2-7-e70019-s004.zip › Supplementary Material 3/Exposureú║inflammatory cytokinesú1⁄4Outcomeú║Scoliosis - ╕▒▒╛/IL2RA/Supplementary Material 3 IL2RA 2.jpeg]

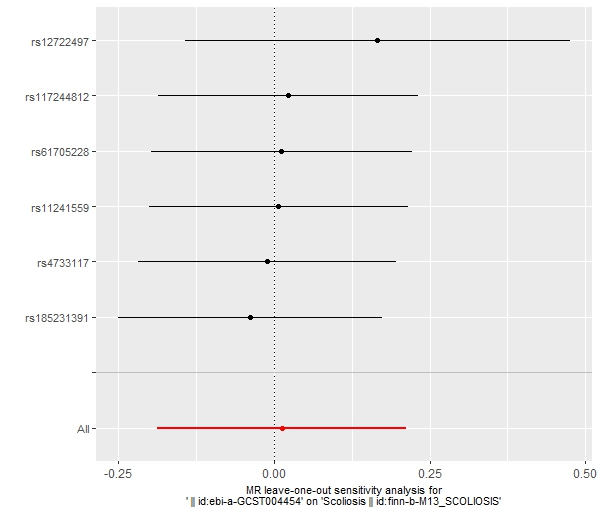

Supplement: Supplementary file 3 — Supplementary Material 3. [file JSP2-7-e70019-s004.zip › Supplementary Material 3/Exposureú║inflammatory cytokinesú1⁄4Outcomeú║Scoliosis - ╕▒▒╛/IL2RA/Supplementary Material 3 IL2RA 3.jpeg]

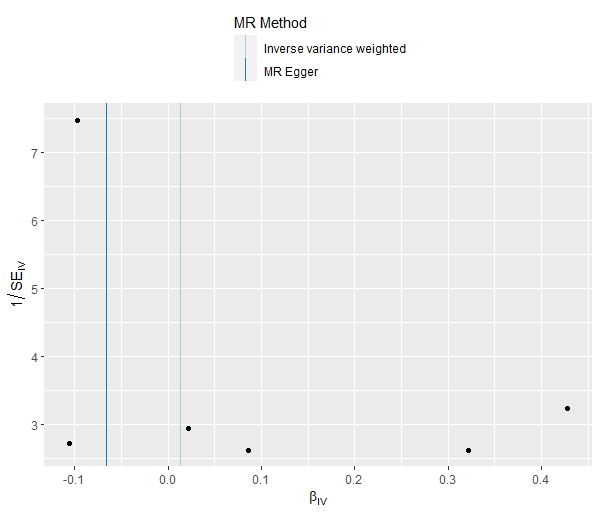

Supplement: Supplementary file 3 — Supplementary Material 3. [file JSP2-7-e70019-s004.zip › Supplementary Material 3/Exposureú║inflammatory cytokinesú1⁄4Outcomeú║Scoliosis - ╕▒▒╛/IL2RA/Supplementary Material 3 IL2RA 4.jpeg]

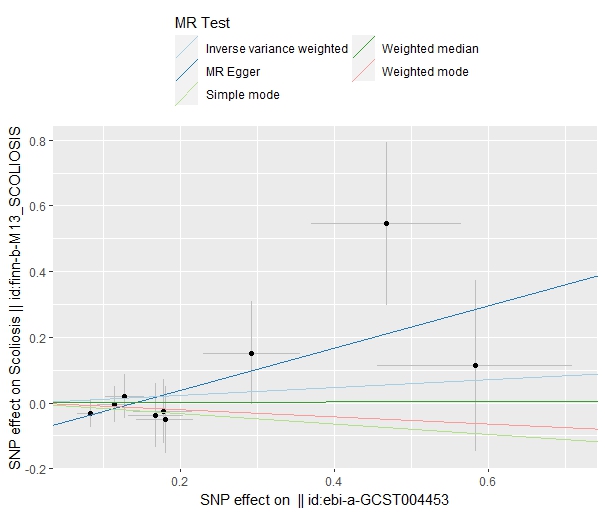

Supplement: Supplementary file 3 — Supplementary Material 3. [file JSP2-7-e70019-s004.zip › Supplementary Material 3/Exposureú║inflammatory cytokinesú1⁄4Outcomeú║Scoliosis - ╕▒▒╛/IL4/Supplementary Material 3 IL4 1.jpeg]

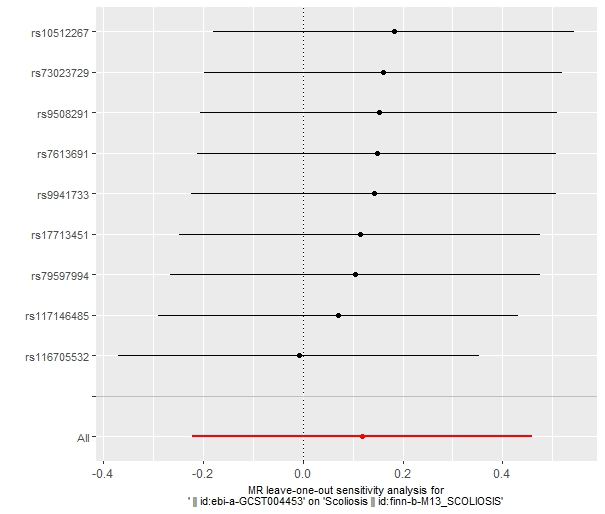

Supplement: Supplementary file 3 — Supplementary Material 3. [file JSP2-7-e70019-s004.zip › Supplementary Material 3/Exposureú║inflammatory cytokinesú1⁄4Outcomeú║Scoliosis - ╕▒▒╛/IL4/Supplementary Material 3 IL4 2.jpeg]

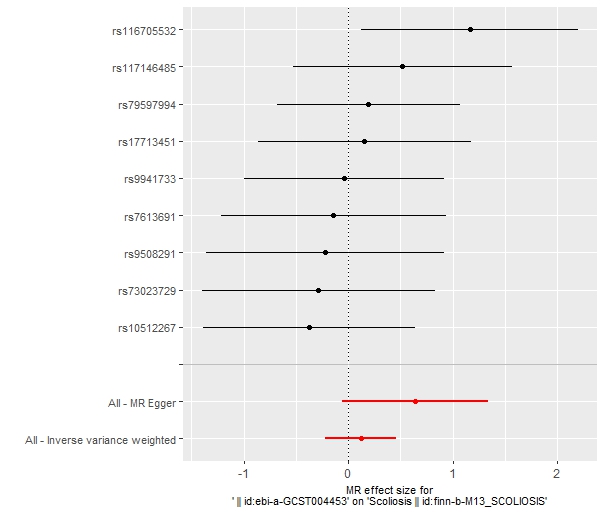

Supplement: Supplementary file 3 — Supplementary Material 3. [file JSP2-7-e70019-s004.zip › Supplementary Material 3/Exposureú║inflammatory cytokinesú1⁄4Outcomeú║Scoliosis - ╕▒▒╛/IL4/Supplementary Material 3 IL4 3.jpeg]

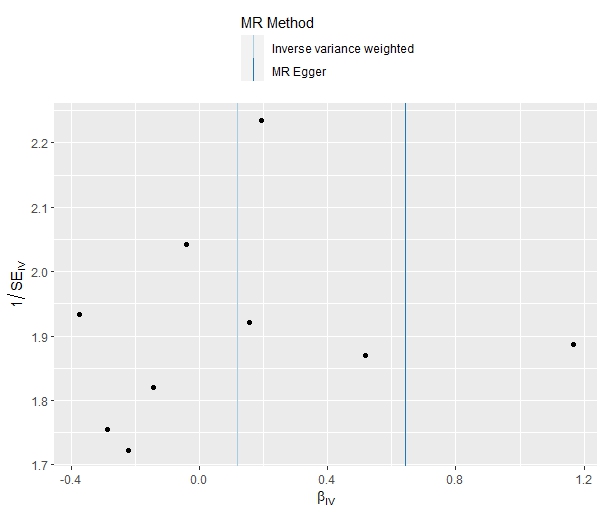

Supplement: Supplementary file 3 — Supplementary Material 3. [file JSP2-7-e70019-s004.zip › Supplementary Material 3/Exposureú║inflammatory cytokinesú1⁄4Outcomeú║Scoliosis - ╕▒▒╛/IL4/Supplementary Material 3 IL4 4.jpeg]

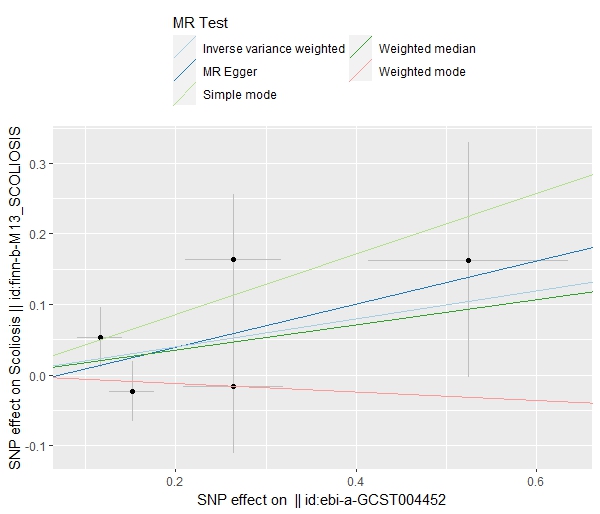

Supplement: Supplementary file 3 — Supplementary Material 3. [file JSP2-7-e70019-s004.zip › Supplementary Material 3/Exposureú║inflammatory cytokinesú1⁄4Outcomeú║Scoliosis - ╕▒▒╛/IL5/Supplementary Material 3 IL5 1.jpeg]

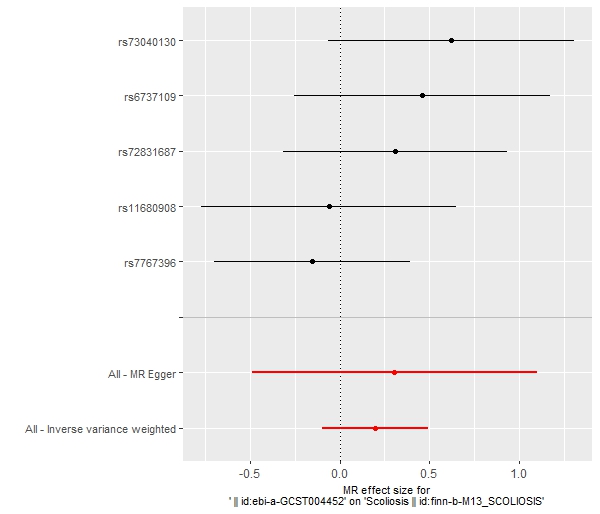

Supplement: Supplementary file 3 — Supplementary Material 3. [file JSP2-7-e70019-s004.zip › Supplementary Material 3/Exposureú║inflammatory cytokinesú1⁄4Outcomeú║Scoliosis - ╕▒▒╛/IL5/Supplementary Material 3 IL5 2.jpeg]

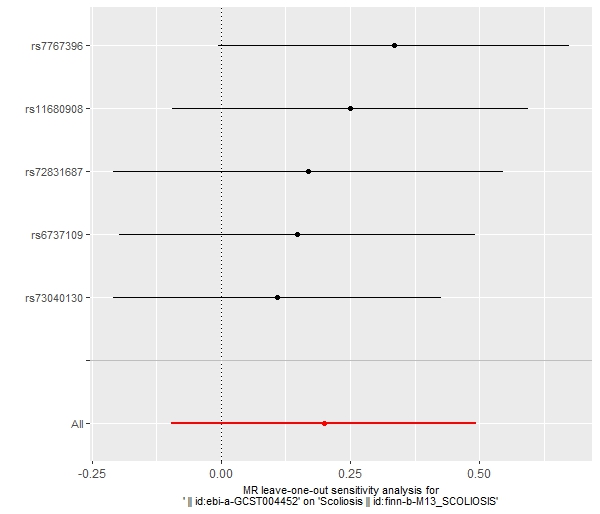

Supplement: Supplementary file 3 — Supplementary Material 3. [file JSP2-7-e70019-s004.zip › Supplementary Material 3/Exposureú║inflammatory cytokinesú1⁄4Outcomeú║Scoliosis - ╕▒▒╛/IL5/Supplementary Material 3 IL5 3.jpeg]

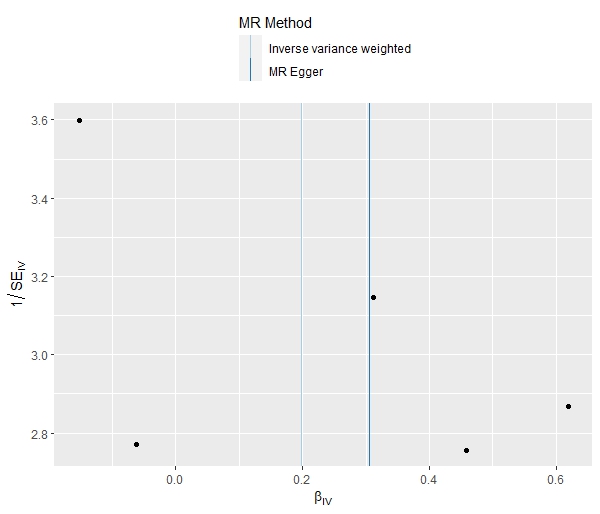

Supplement: Supplementary file 3 — Supplementary Material 3. [file JSP2-7-e70019-s004.zip › Supplementary Material 3/Exposureú║inflammatory cytokinesú1⁄4Outcomeú║Scoliosis - ╕▒▒╛/IL5/Supplementary Material 3 IL5 4.jpeg]

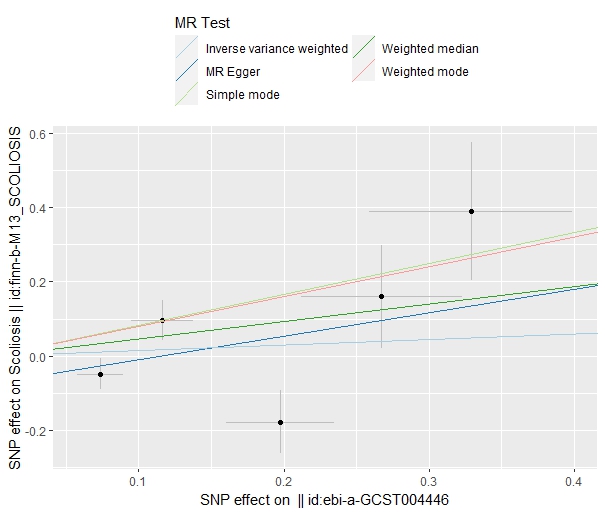

Supplement: Supplementary file 3 — Supplementary Material 3. [file JSP2-7-e70019-s004.zip › Supplementary Material 3/Exposureú║inflammatory cytokinesú1⁄4Outcomeú║Scoliosis - ╕▒▒╛/IL6/Supplementary Material 3 IL6 1.jpeg]

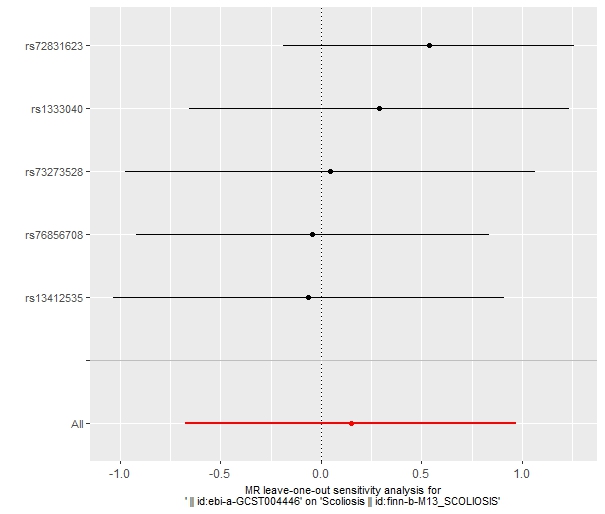

Supplement: Supplementary file 3 — Supplementary Material 3. [file JSP2-7-e70019-s004.zip › Supplementary Material 3/Exposureú║inflammatory cytokinesú1⁄4Outcomeú║Scoliosis - ╕▒▒╛/IL6/Supplementary Material 3 IL6 2.jpeg]

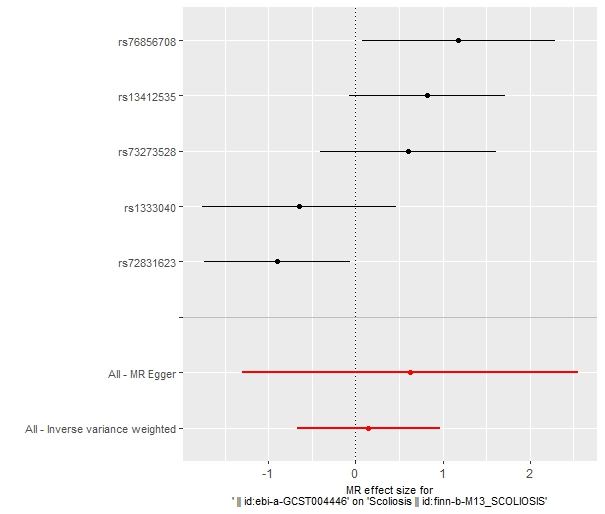

Supplement: Supplementary file 3 — Supplementary Material 3. [file JSP2-7-e70019-s004.zip › Supplementary Material 3/Exposureú║inflammatory cytokinesú1⁄4Outcomeú║Scoliosis - ╕▒▒╛/IL6/Supplementary Material 3 IL6 3.jpeg]

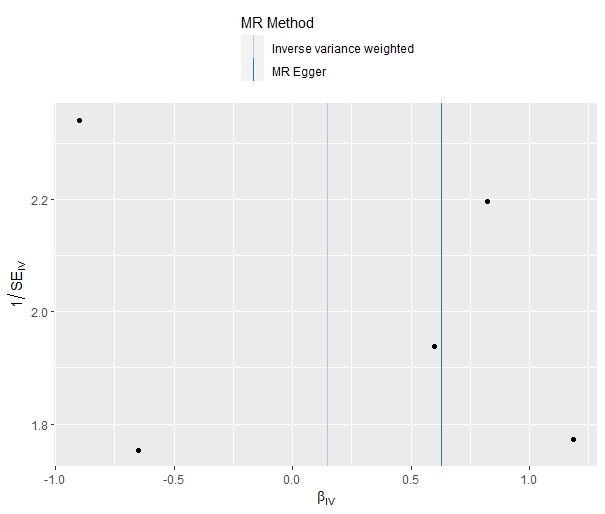

Supplement: Supplementary file 3 — Supplementary Material 3. [file JSP2-7-e70019-s004.zip › Supplementary Material 3/Exposureú║inflammatory cytokinesú1⁄4Outcomeú║Scoliosis - ╕▒▒╛/IL6/Supplementary Material 3 IL6 4.jpeg]

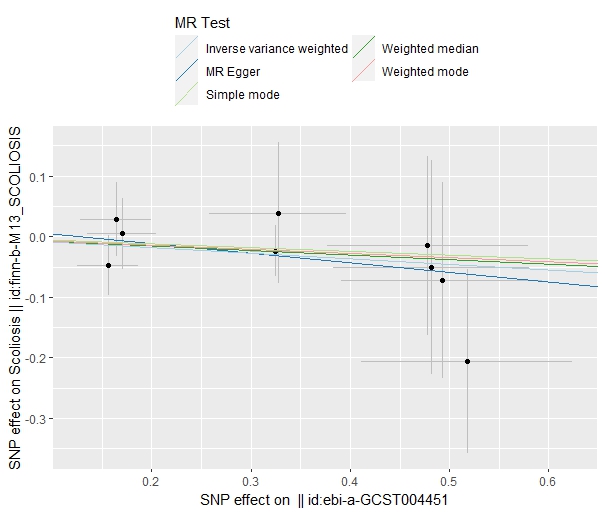

Supplement: Supplementary file 3 — Supplementary Material 3. [file JSP2-7-e70019-s004.zip › Supplementary Material 3/Exposureú║inflammatory cytokinesú1⁄4Outcomeú║Scoliosis - ╕▒▒╛/IL7/Supplementary Material 3 IL7 1.jpeg]

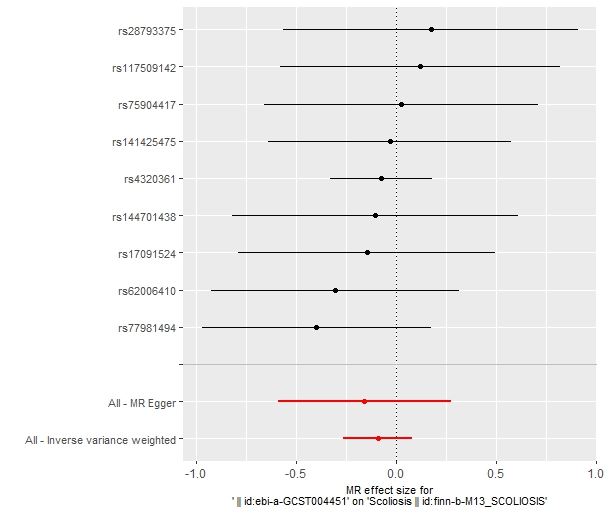

Supplement: Supplementary file 3 — Supplementary Material 3. [file JSP2-7-e70019-s004.zip › Supplementary Material 3/Exposureú║inflammatory cytokinesú1⁄4Outcomeú║Scoliosis - ╕▒▒╛/IL7/Supplementary Material 3 IL7 2.jpeg]

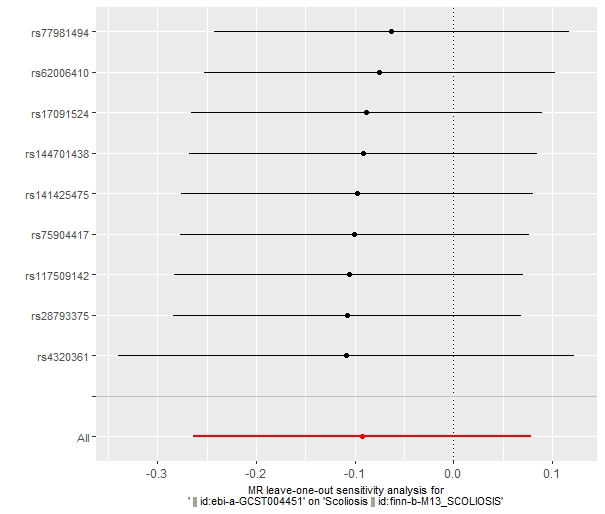

Supplement: Supplementary file 3 — Supplementary Material 3. [file JSP2-7-e70019-s004.zip › Supplementary Material 3/Exposureú║inflammatory cytokinesú1⁄4Outcomeú║Scoliosis - ╕▒▒╛/IL7/Supplementary Material 3 IL7 3.jpeg]

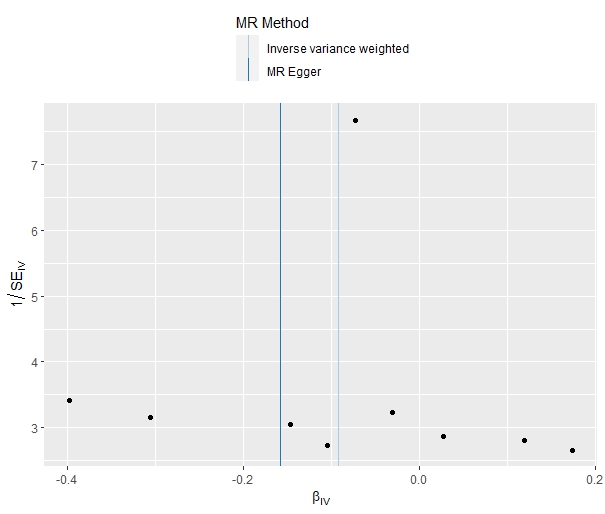

Supplement: Supplementary file 3 — Supplementary Material 3. [file JSP2-7-e70019-s004.zip › Supplementary Material 3/Exposureú║inflammatory cytokinesú1⁄4Outcomeú║Scoliosis - ╕▒▒╛/IL7/Supplementary Material 3 IL7 4.jpeg]

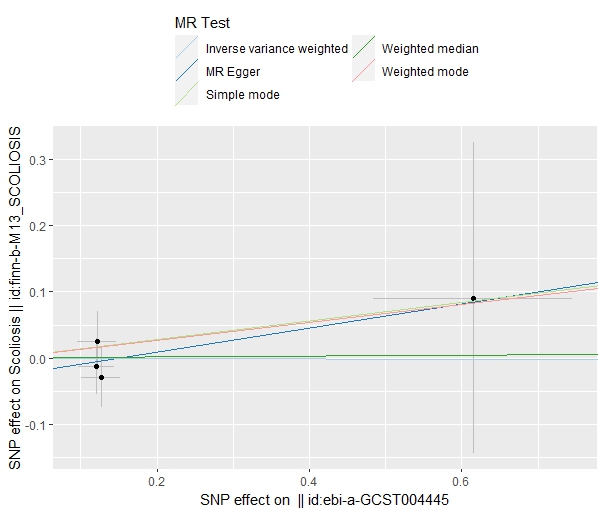

Supplement: Supplementary file 3 — Supplementary Material 3. [file JSP2-7-e70019-s004.zip › Supplementary Material 3/Exposureú║inflammatory cytokinesú1⁄4Outcomeú║Scoliosis - ╕▒▒╛/IL8/Supplementary Material 3 IL8 1.jpeg]

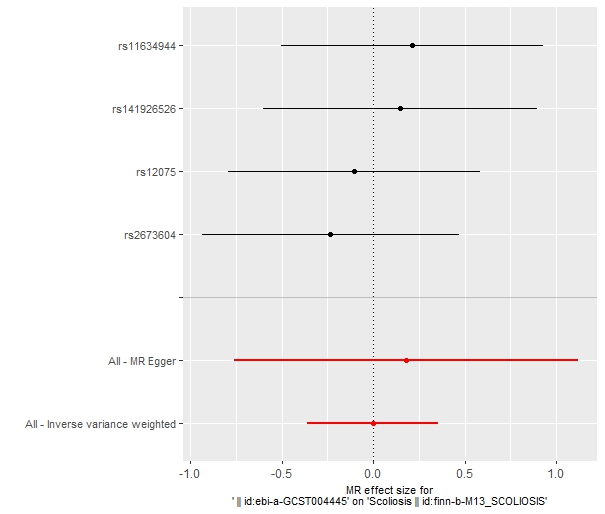

Supplement: Supplementary file 3 — Supplementary Material 3. [file JSP2-7-e70019-s004.zip › Supplementary Material 3/Exposureú║inflammatory cytokinesú1⁄4Outcomeú║Scoliosis - ╕▒▒╛/IL8/Supplementary Material 3 IL8 2.jpeg]

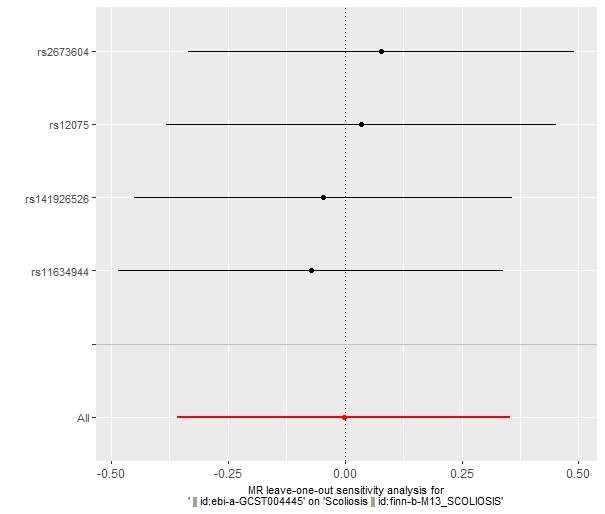

Supplement: Supplementary file 3 — Supplementary Material 3. [file JSP2-7-e70019-s004.zip › Supplementary Material 3/Exposureú║inflammatory cytokinesú1⁄4Outcomeú║Scoliosis - ╕▒▒╛/IL8/Supplementary Material 3 IL8 3.jpeg]

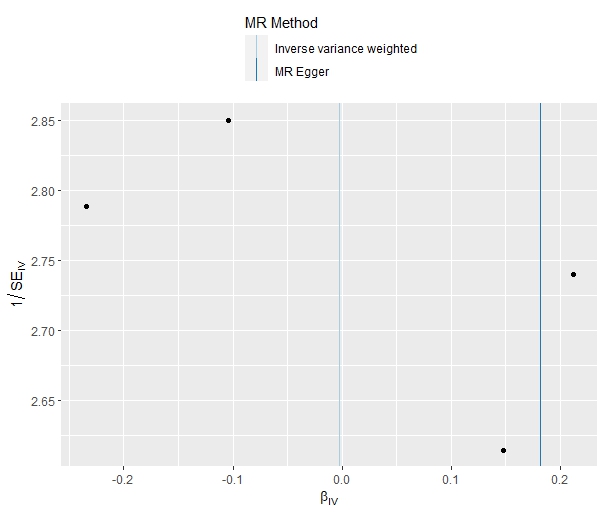

Supplement: Supplementary file 3 — Supplementary Material 3. [file JSP2-7-e70019-s004.zip › Supplementary Material 3/Exposureú║inflammatory cytokinesú1⁄4Outcomeú║Scoliosis - ╕▒▒╛/IL8/Supplementary Material 3 IL8 4.jpeg]

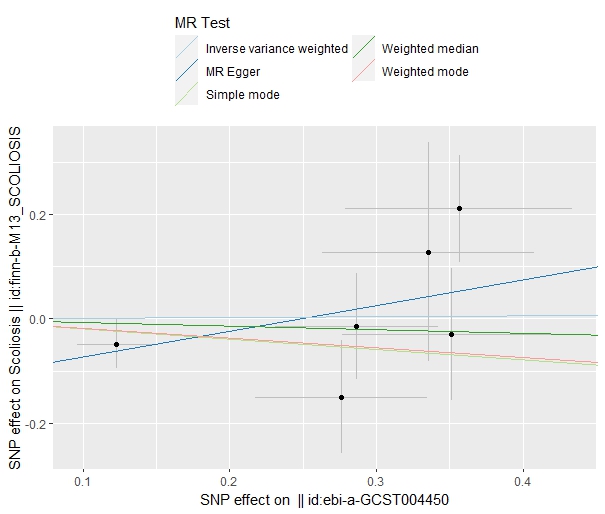

Supplement: Supplementary file 3 — Supplementary Material 3. [file JSP2-7-e70019-s004.zip › Supplementary Material 3/Exposureú║inflammatory cytokinesú1⁄4Outcomeú║Scoliosis - ╕▒▒╛/IL9/Supplementary Material 3 IL9 1.jpeg]

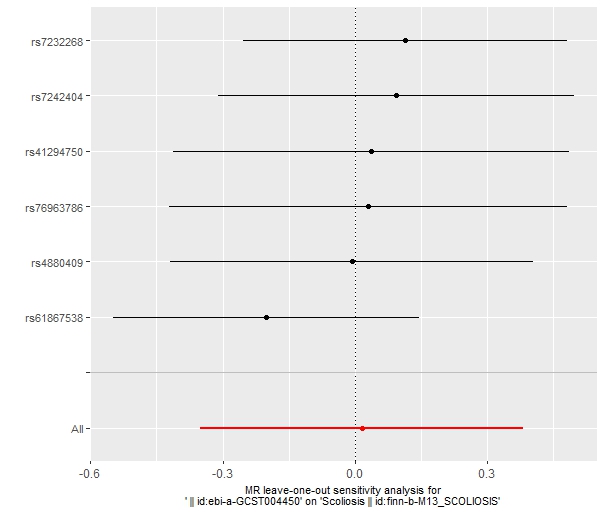

Supplement: Supplementary file 3 — Supplementary Material 3. [file JSP2-7-e70019-s004.zip › Supplementary Material 3/Exposureú║inflammatory cytokinesú1⁄4Outcomeú║Scoliosis - ╕▒▒╛/IL9/Supplementary Material 3 IL9 2.jpeg]

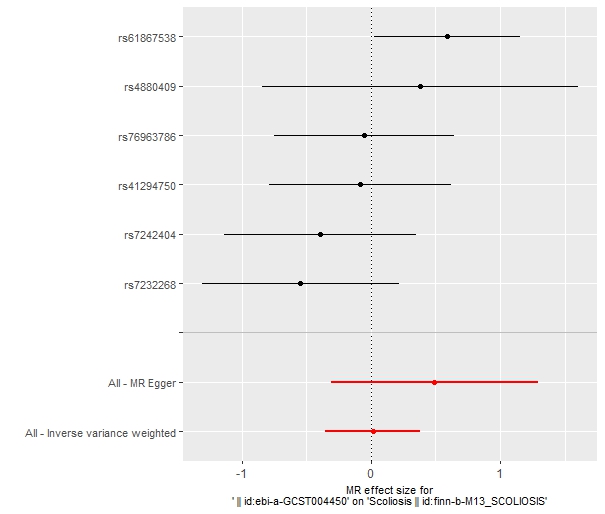

Supplement: Supplementary file 3 — Supplementary Material 3. [file JSP2-7-e70019-s004.zip › Supplementary Material 3/Exposureú║inflammatory cytokinesú1⁄4Outcomeú║Scoliosis - ╕▒▒╛/IL9/Supplementary Material 3 IL9 3.jpeg]

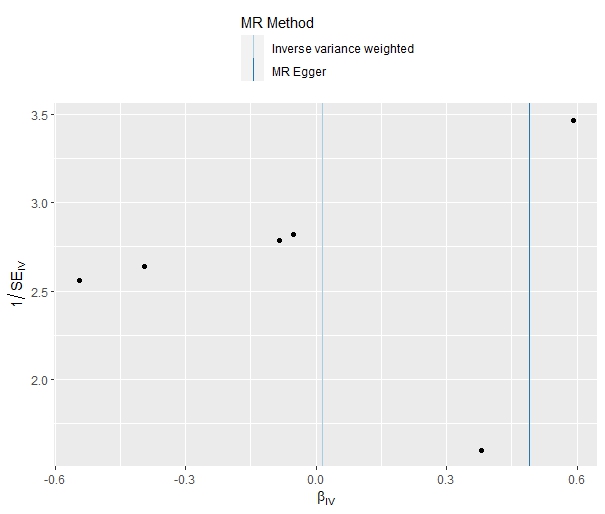

Supplement: Supplementary file 3 — Supplementary Material 3. [file JSP2-7-e70019-s004.zip › Supplementary Material 3/Exposureú║inflammatory cytokinesú1⁄4Outcomeú║Scoliosis - ╕▒▒╛/IL9/Supplementary Material 3 IL9 4.jpeg]

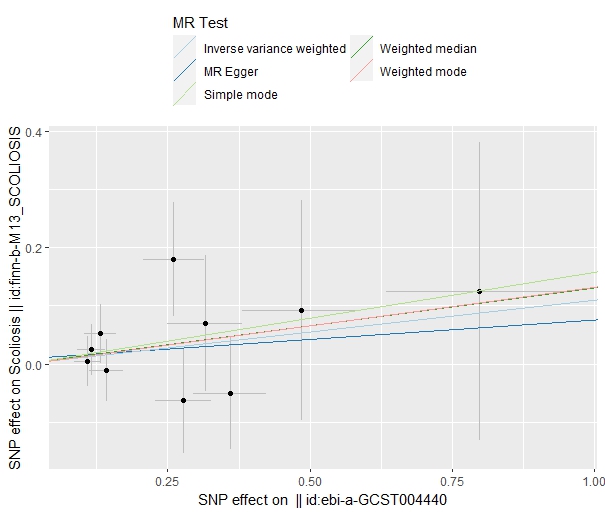

Supplement: Supplementary file 3 — Supplementary Material 3. [file JSP2-7-e70019-s004.zip › Supplementary Material 3/Exposureú║inflammatory cytokinesú1⁄4Outcomeú║Scoliosis - ╕▒▒╛/IP10/Supplementary Material 3 IP10 1.jpeg]

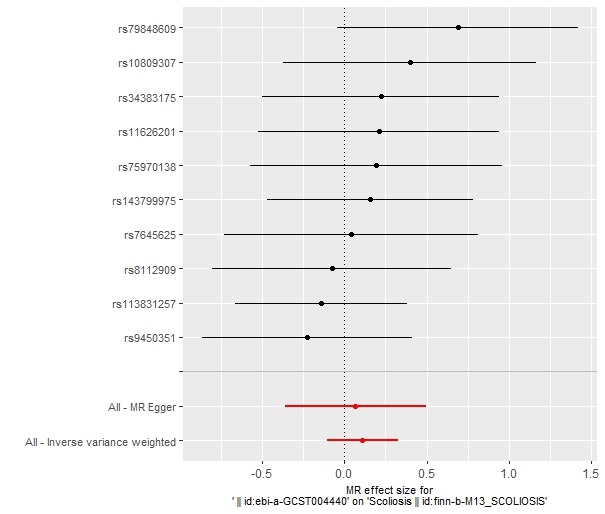

Supplement: Supplementary file 3 — Supplementary Material 3. [file JSP2-7-e70019-s004.zip › Supplementary Material 3/Exposureú║inflammatory cytokinesú1⁄4Outcomeú║Scoliosis - ╕▒▒╛/IP10/Supplementary Material 3 IP10 2.jpeg]

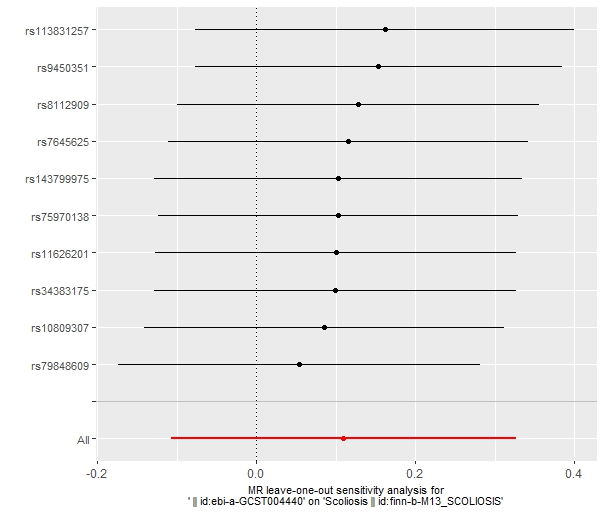

Supplement: Supplementary file 3 — Supplementary Material 3. [file JSP2-7-e70019-s004.zip › Supplementary Material 3/Exposureú║inflammatory cytokinesú1⁄4Outcomeú║Scoliosis - ╕▒▒╛/IP10/Supplementary Material 3 IP10 3.jpeg]

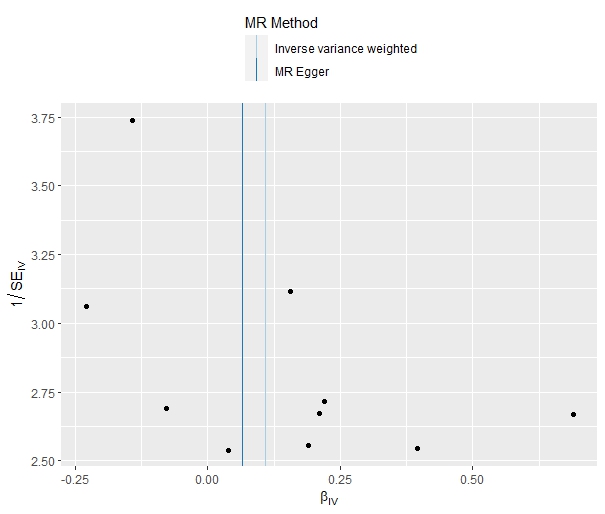

Supplement: Supplementary file 3 — Supplementary Material 3. [file JSP2-7-e70019-s004.zip › Supplementary Material 3/Exposureú║inflammatory cytokinesú1⁄4Outcomeú║Scoliosis - ╕▒▒╛/IP10/Supplementary Material 3 IP10 4.jpeg]

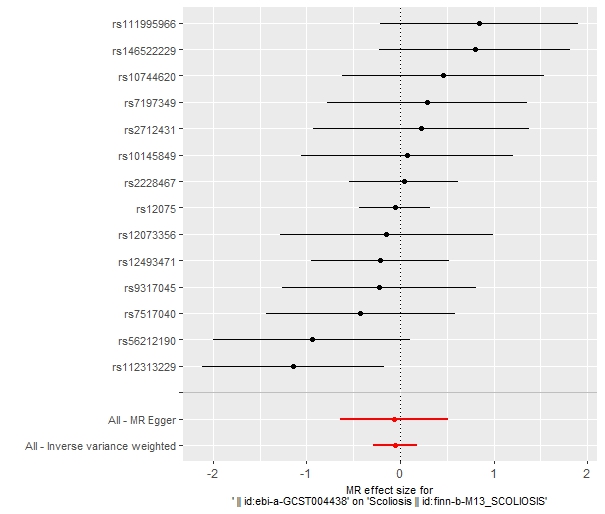

Supplement: Supplementary file 3 — Supplementary Material 3. [file JSP2-7-e70019-s004.zip › Supplementary Material 3/Exposureú║inflammatory cytokinesú1⁄4Outcomeú║Scoliosis - ╕▒▒╛/MCP1/Supplementary Material 3 MCP1 1.jpeg]

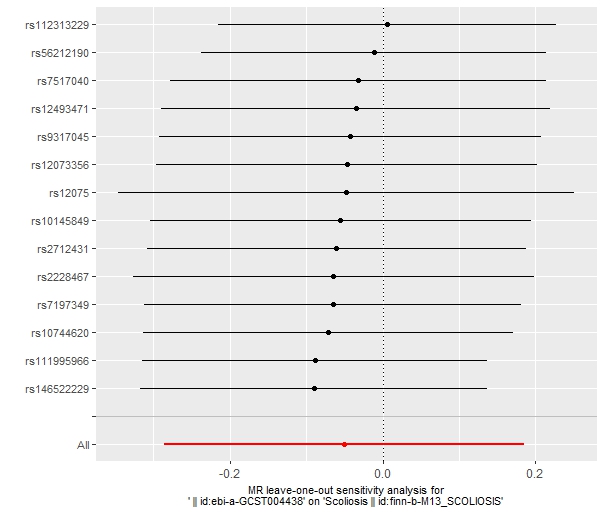

Supplement: Supplementary file 3 — Supplementary Material 3. [file JSP2-7-e70019-s004.zip › Supplementary Material 3/Exposureú║inflammatory cytokinesú1⁄4Outcomeú║Scoliosis - ╕▒▒╛/MCP1/Supplementary Material 3 MCP1 2.jpeg]

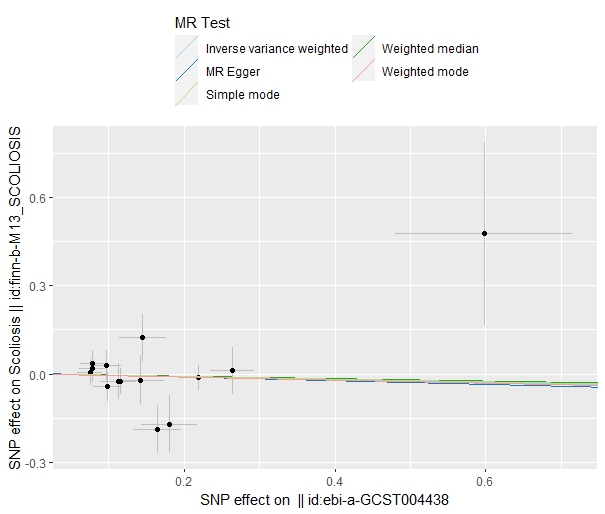

Supplement: Supplementary file 3 — Supplementary Material 3. [file JSP2-7-e70019-s004.zip › Supplementary Material 3/Exposureú║inflammatory cytokinesú1⁄4Outcomeú║Scoliosis - ╕▒▒╛/MCP1/Supplementary Material 3 MCP1 3.jpeg]

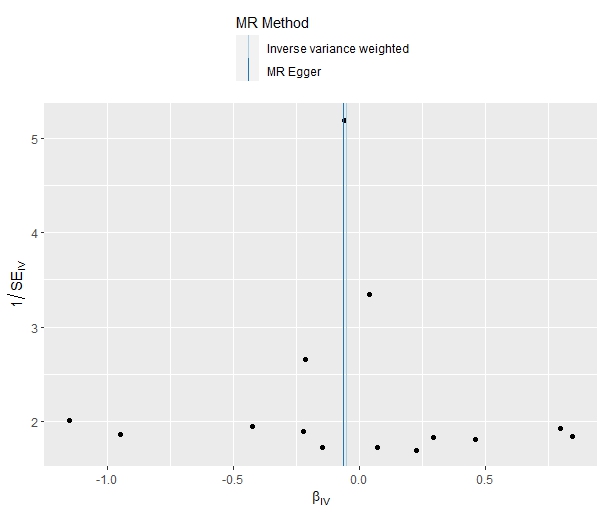

Supplement: Supplementary file 3 — Supplementary Material 3. [file JSP2-7-e70019-s004.zip › Supplementary Material 3/Exposureú║inflammatory cytokinesú1⁄4Outcomeú║Scoliosis - ╕▒▒╛/MCP1/Supplementary Material 3 MCP1 4.jpeg]
