# Supplementary figures and images for: Investigating the causal links between inflammatory cytokines and scoliosis through bidirectional Mendelian randomization analysis (part 3 of 3)
Source: JOR Spine. 2024 Dec 11;7(4):e70019. doi: 10.1002/jsp2.70019 (PMC11632254; doi:10.1002/jsp2.70019)

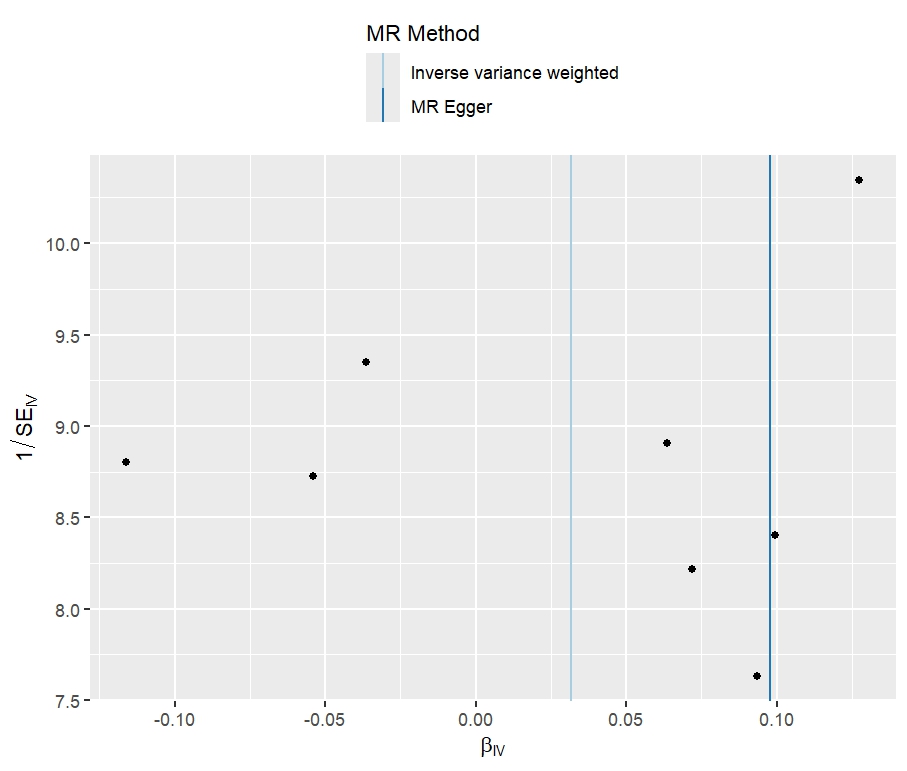

Supplement: Supplementary file 4 — Supplementary Material 4. [file JSP2-7-e70019-s002.zip › Supplementary Material 4/Exposureú║Scoliosisú1⁄4Outcomeú║inflammatory cytokines/IL2RA/Supplementary Material 4 IL2RA 4.jpeg]

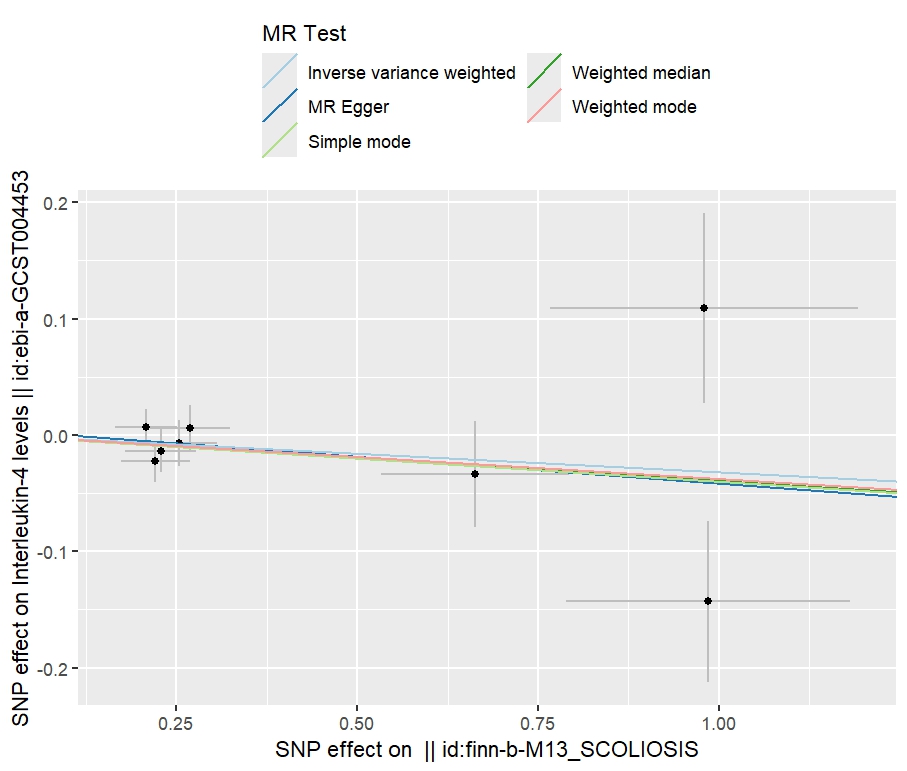

Supplement: Supplementary file 4 — Supplementary Material 4. [file JSP2-7-e70019-s002.zip › Supplementary Material 4/Exposureú║Scoliosisú1⁄4Outcomeú║inflammatory cytokines/IL4/Supplementary Material 4 IL4 1.jpeg]

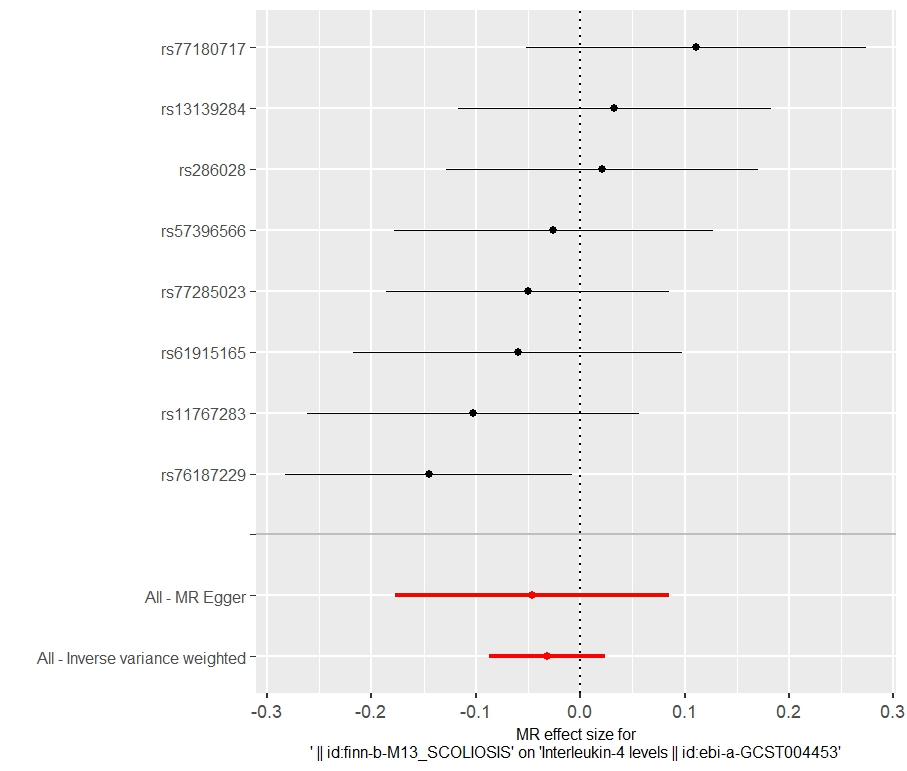

Supplement: Supplementary file 4 — Supplementary Material 4. [file JSP2-7-e70019-s002.zip › Supplementary Material 4/Exposureú║Scoliosisú1⁄4Outcomeú║inflammatory cytokines/IL4/Supplementary Material 4 IL4 2.jpeg]

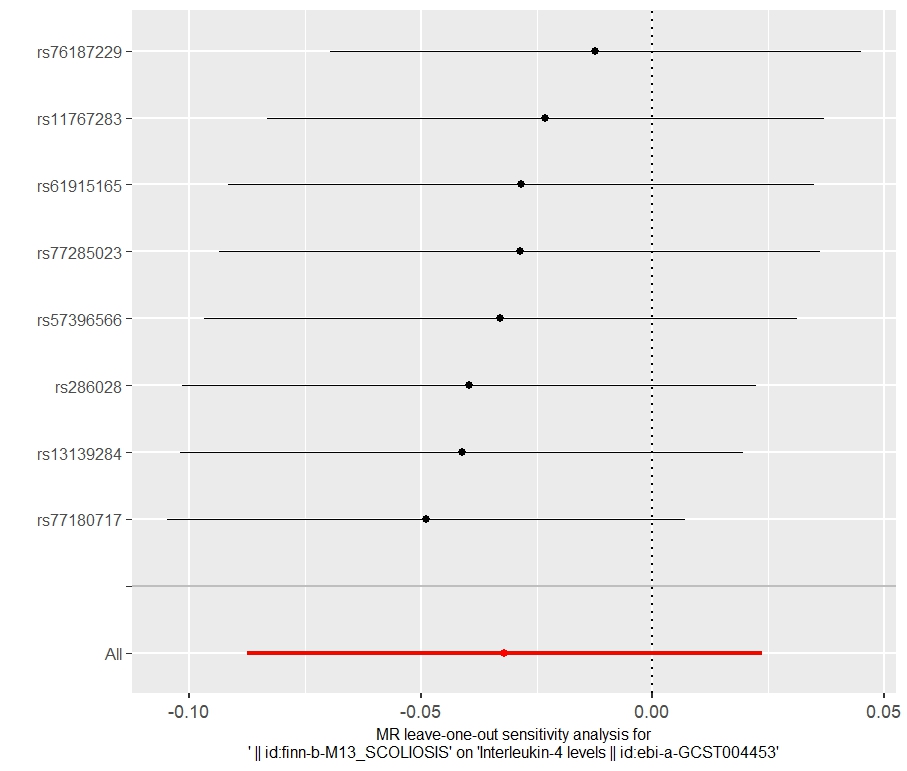

Supplement: Supplementary file 4 — Supplementary Material 4. [file JSP2-7-e70019-s002.zip › Supplementary Material 4/Exposureú║Scoliosisú1⁄4Outcomeú║inflammatory cytokines/IL4/Supplementary Material 4 IL4 3.jpeg]

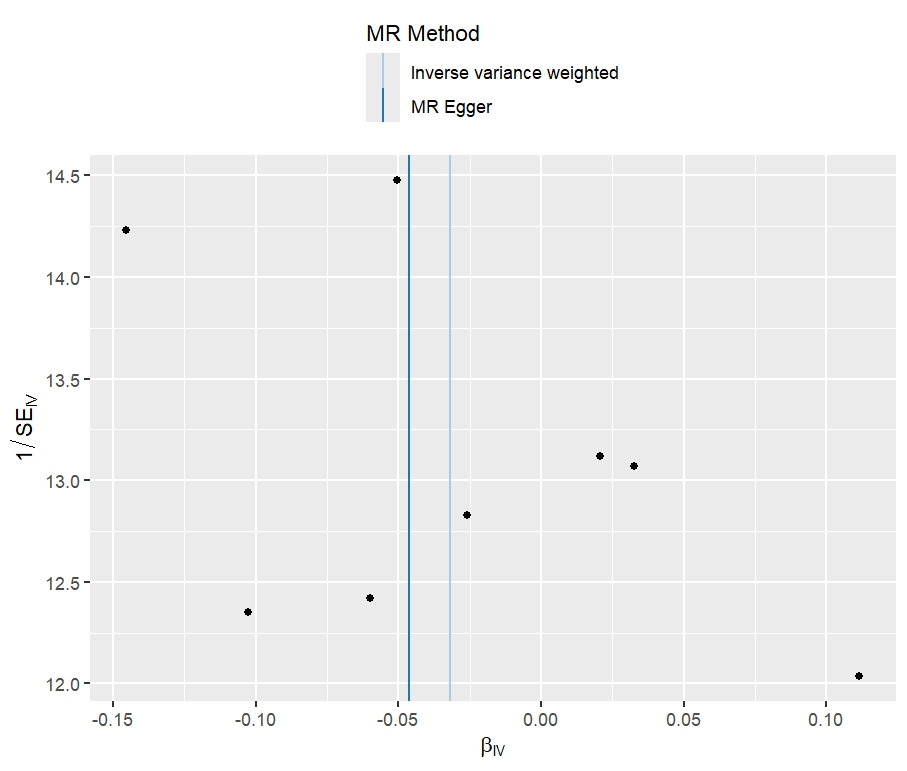

Supplement: Supplementary file 4 — Supplementary Material 4. [file JSP2-7-e70019-s002.zip › Supplementary Material 4/Exposureú║Scoliosisú1⁄4Outcomeú║inflammatory cytokines/IL4/Supplementary Material 4 IL4 4.jpeg]

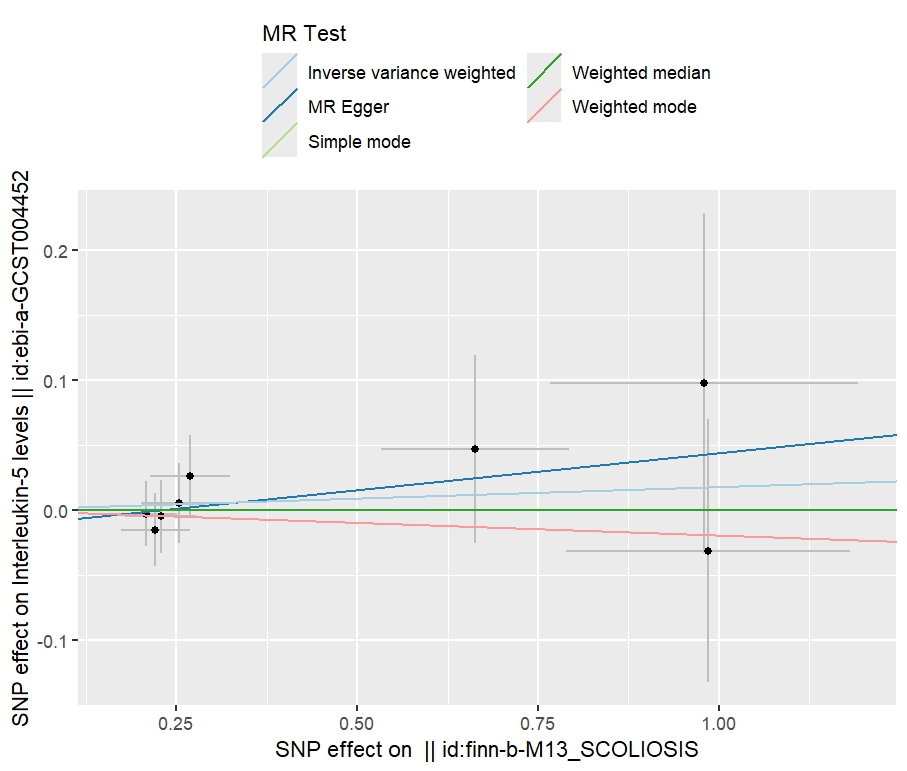

Supplement: Supplementary file 4 — Supplementary Material 4. [file JSP2-7-e70019-s002.zip › Supplementary Material 4/Exposureú║Scoliosisú1⁄4Outcomeú║inflammatory cytokines/IL5/Supplementary Material 4 IL5 1.jpeg]

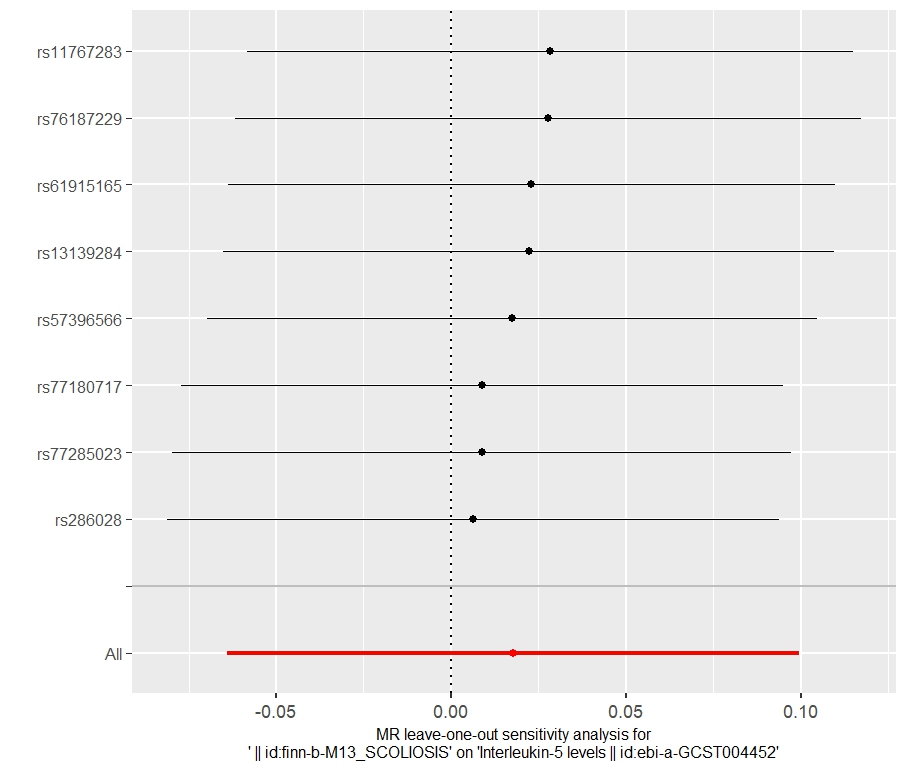

Supplement: Supplementary file 4 — Supplementary Material 4. [file JSP2-7-e70019-s002.zip › Supplementary Material 4/Exposureú║Scoliosisú1⁄4Outcomeú║inflammatory cytokines/IL5/Supplementary Material 4 IL5 2.jpeg]

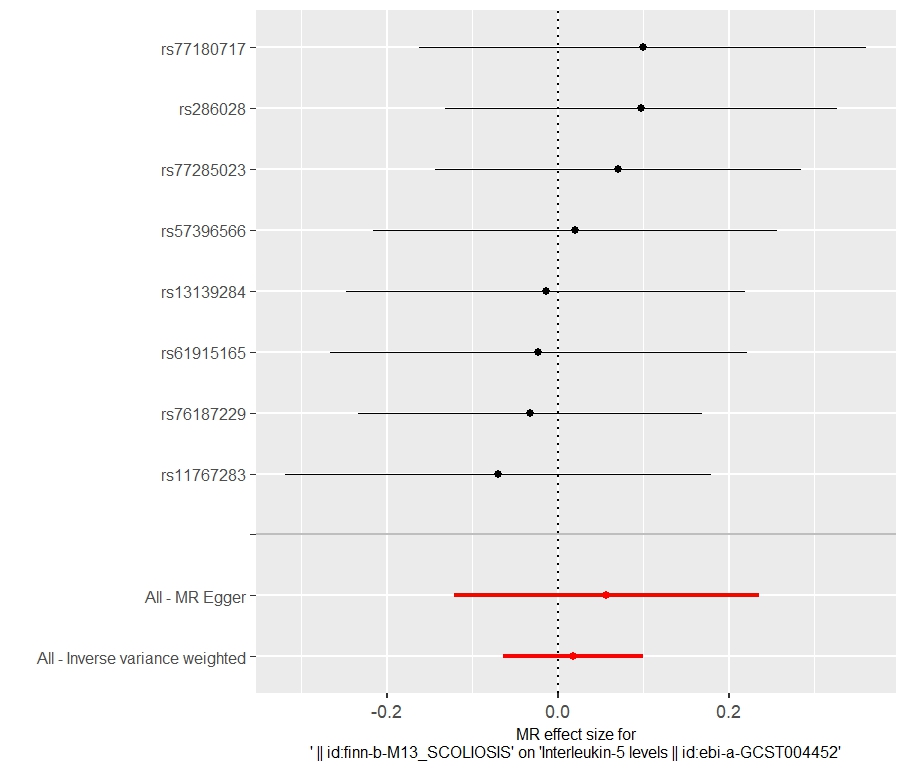

Supplement: Supplementary file 4 — Supplementary Material 4. [file JSP2-7-e70019-s002.zip › Supplementary Material 4/Exposureú║Scoliosisú1⁄4Outcomeú║inflammatory cytokines/IL5/Supplementary Material 4 IL5 3.jpeg]

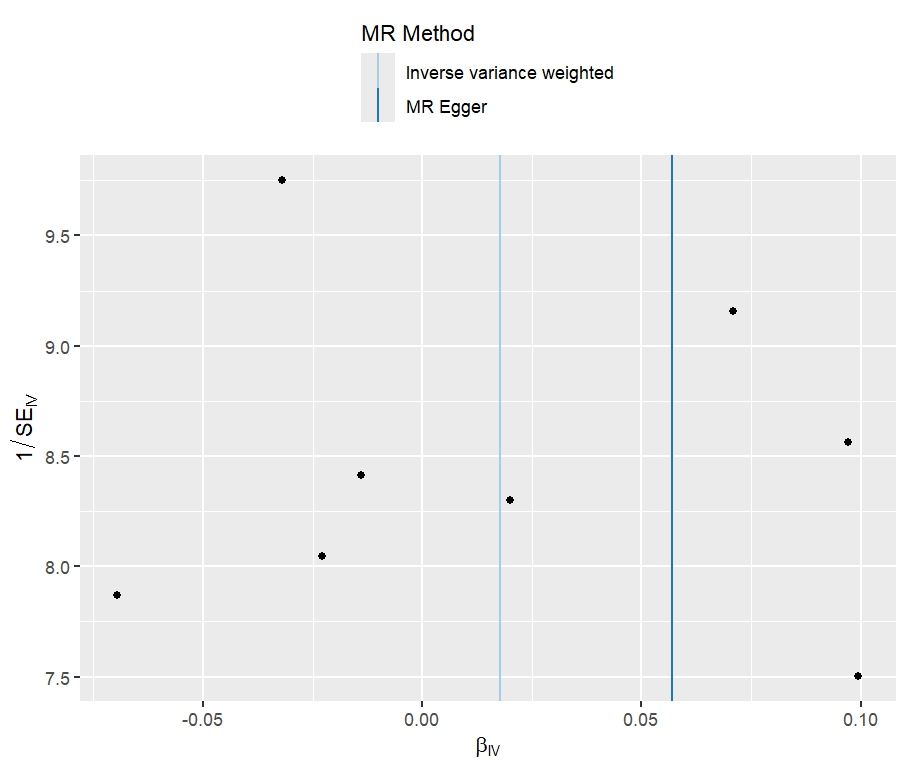

Supplement: Supplementary file 4 — Supplementary Material 4. [file JSP2-7-e70019-s002.zip › Supplementary Material 4/Exposureú║Scoliosisú1⁄4Outcomeú║inflammatory cytokines/IL5/Supplementary Material 4 IL5 4.jpeg]

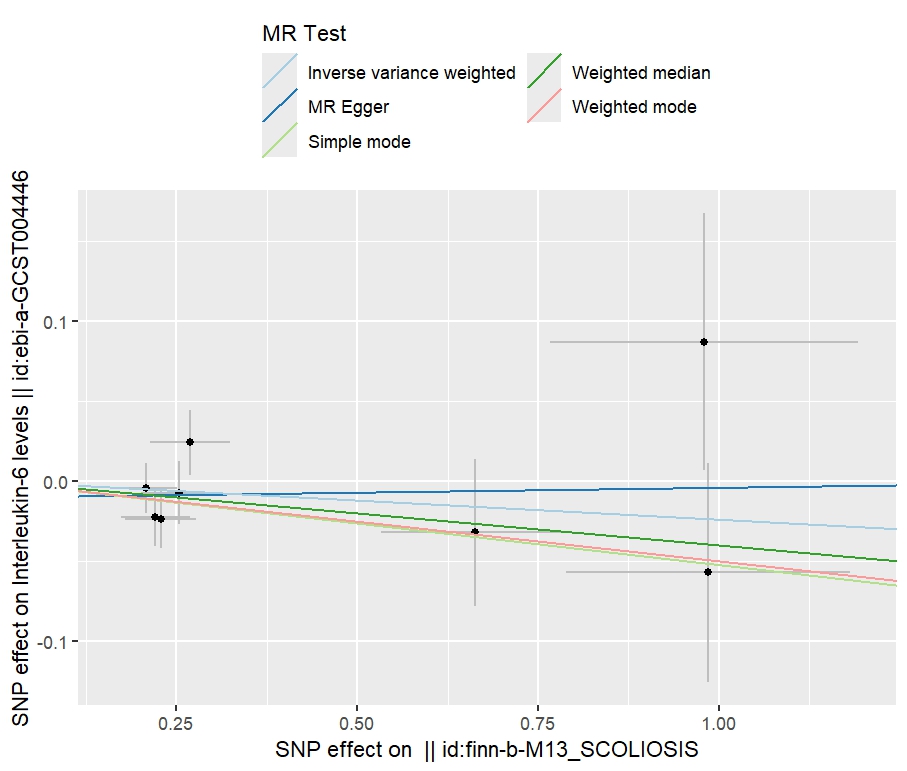

Supplement: Supplementary file 4 — Supplementary Material 4. [file JSP2-7-e70019-s002.zip › Supplementary Material 4/Exposureú║Scoliosisú1⁄4Outcomeú║inflammatory cytokines/IL6/Supplementary Material 4 IL6 1.jpeg]

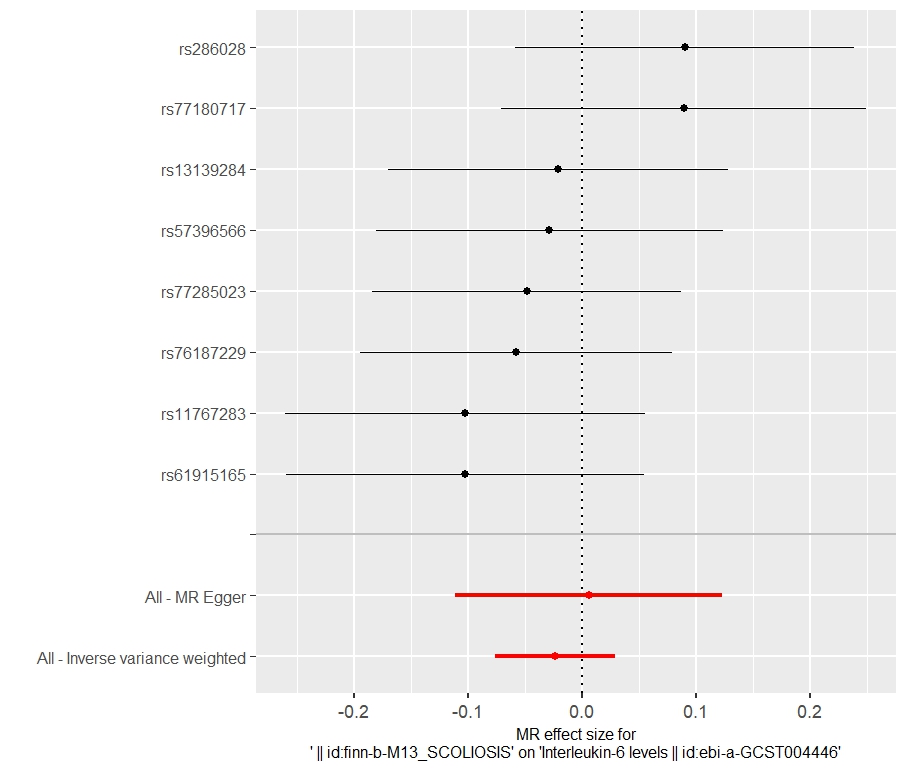

Supplement: Supplementary file 4 — Supplementary Material 4. [file JSP2-7-e70019-s002.zip › Supplementary Material 4/Exposureú║Scoliosisú1⁄4Outcomeú║inflammatory cytokines/IL6/Supplementary Material 4 IL6 2.jpeg]

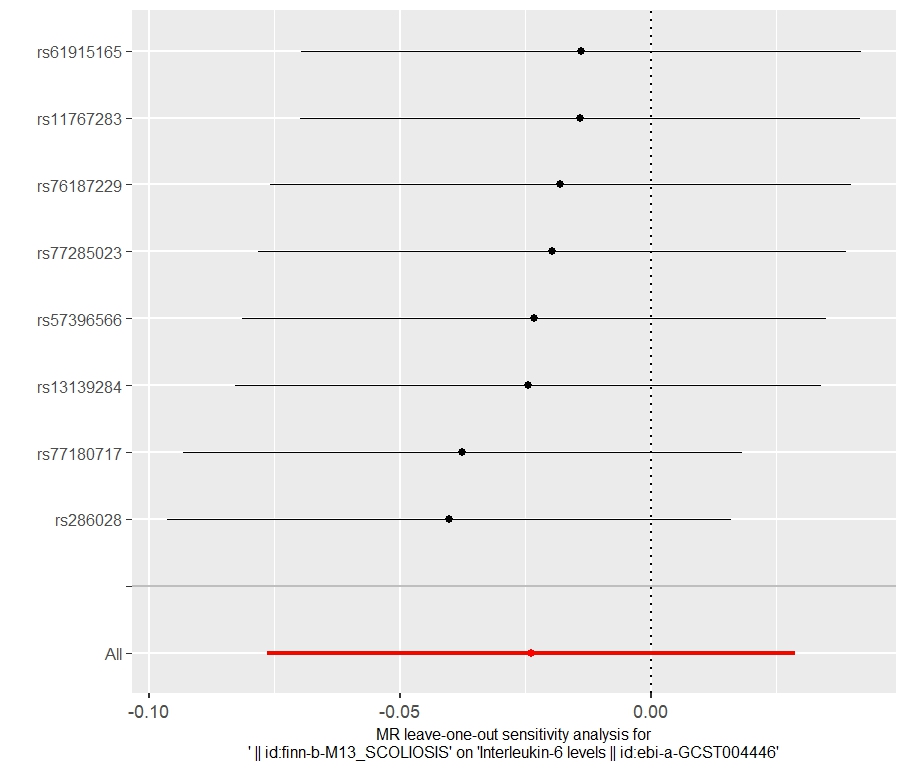

Supplement: Supplementary file 4 — Supplementary Material 4. [file JSP2-7-e70019-s002.zip › Supplementary Material 4/Exposureú║Scoliosisú1⁄4Outcomeú║inflammatory cytokines/IL6/Supplementary Material 4 IL6 3.jpeg]

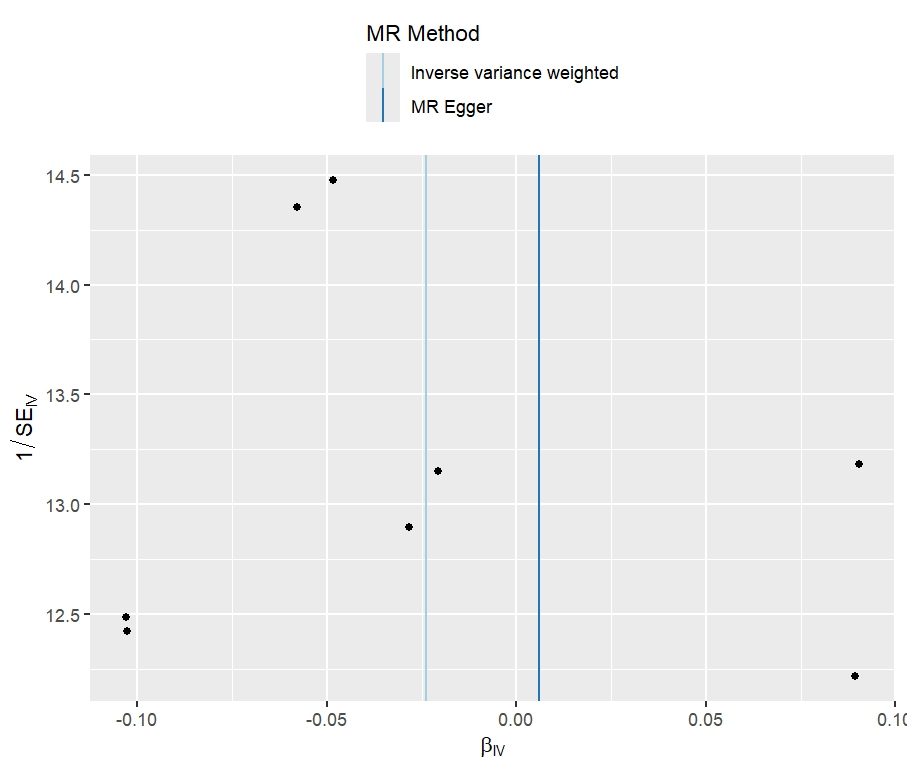

Supplement: Supplementary file 4 — Supplementary Material 4. [file JSP2-7-e70019-s002.zip › Supplementary Material 4/Exposureú║Scoliosisú1⁄4Outcomeú║inflammatory cytokines/IL6/Supplementary Material 4 IL6 4.jpeg]

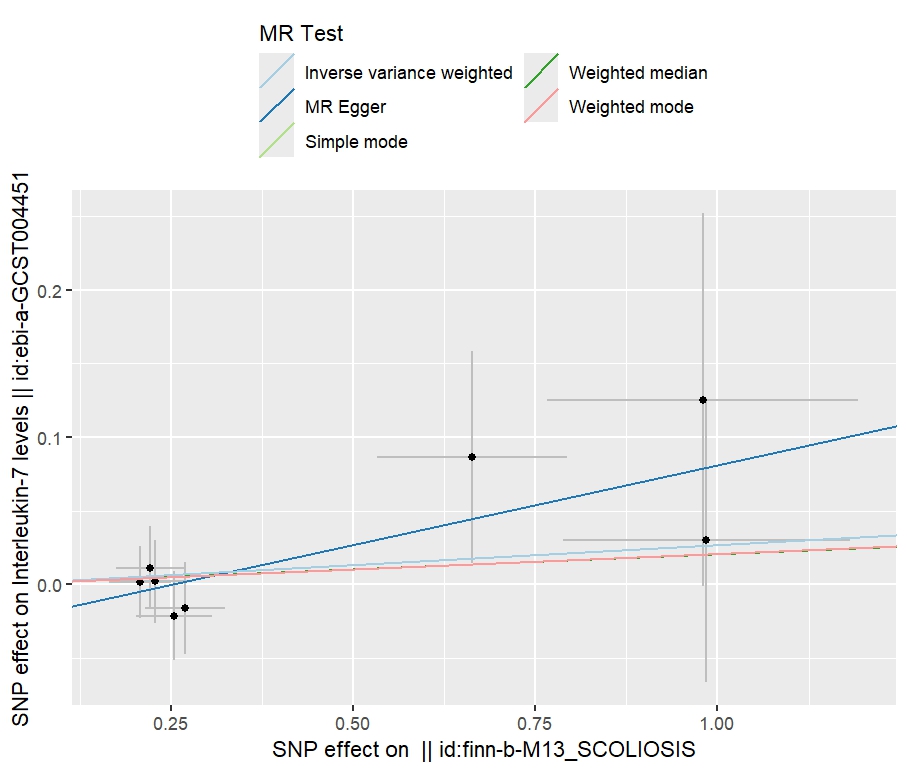

Supplement: Supplementary file 4 — Supplementary Material 4. [file JSP2-7-e70019-s002.zip › Supplementary Material 4/Exposureú║Scoliosisú1⁄4Outcomeú║inflammatory cytokines/IL7/Supplementary Material 4 IL7 1.jpeg]

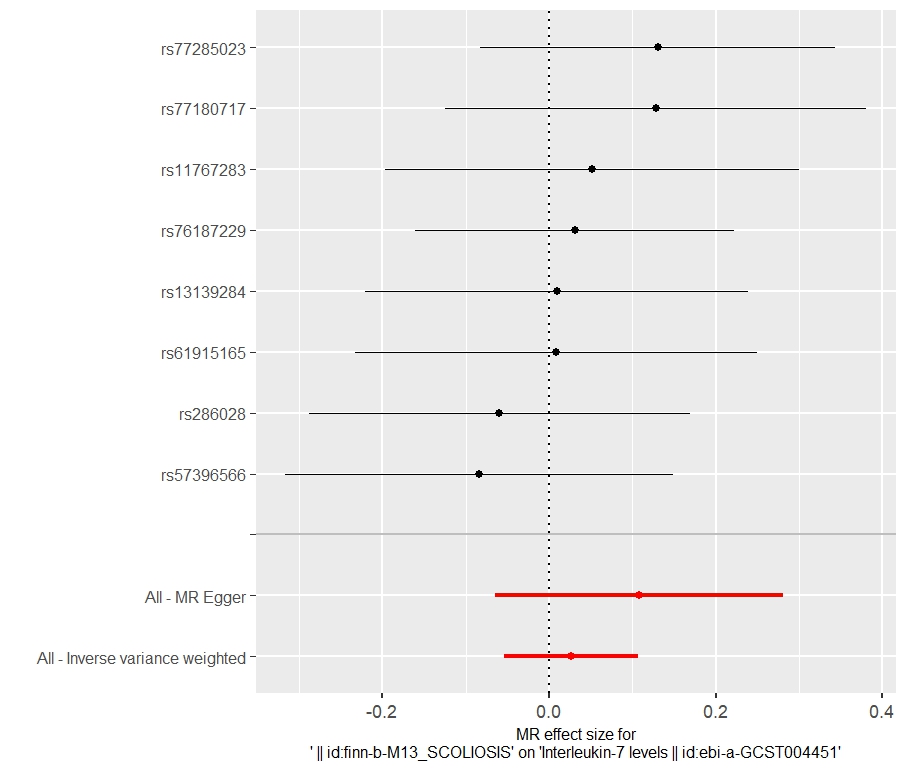

Supplement: Supplementary file 4 — Supplementary Material 4. [file JSP2-7-e70019-s002.zip › Supplementary Material 4/Exposureú║Scoliosisú1⁄4Outcomeú║inflammatory cytokines/IL7/Supplementary Material 4 IL7 2.jpeg]

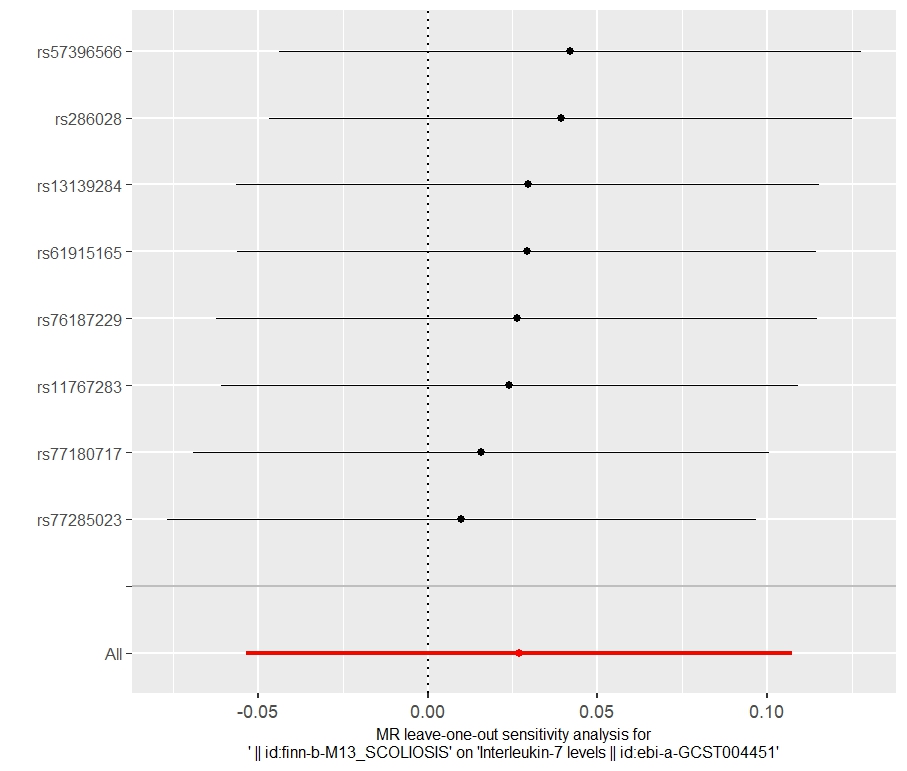

Supplement: Supplementary file 4 — Supplementary Material 4. [file JSP2-7-e70019-s002.zip › Supplementary Material 4/Exposureú║Scoliosisú1⁄4Outcomeú║inflammatory cytokines/IL7/Supplementary Material 4 IL7 3.jpeg]

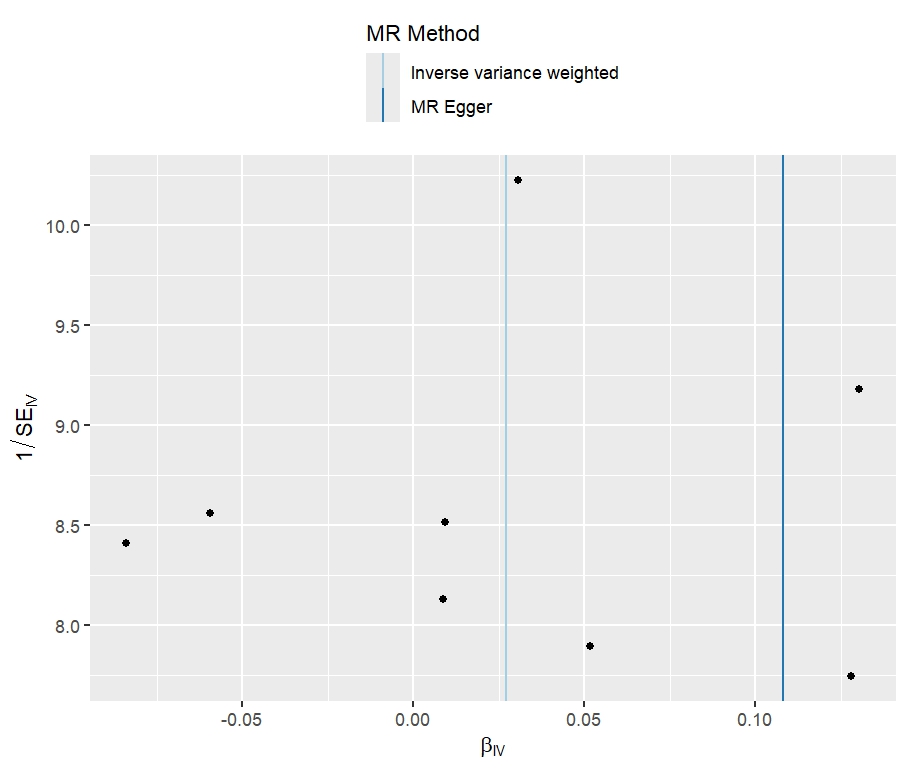

Supplement: Supplementary file 4 — Supplementary Material 4. [file JSP2-7-e70019-s002.zip › Supplementary Material 4/Exposureú║Scoliosisú1⁄4Outcomeú║inflammatory cytokines/IL7/Supplementary Material 4 IL7 4.jpeg]

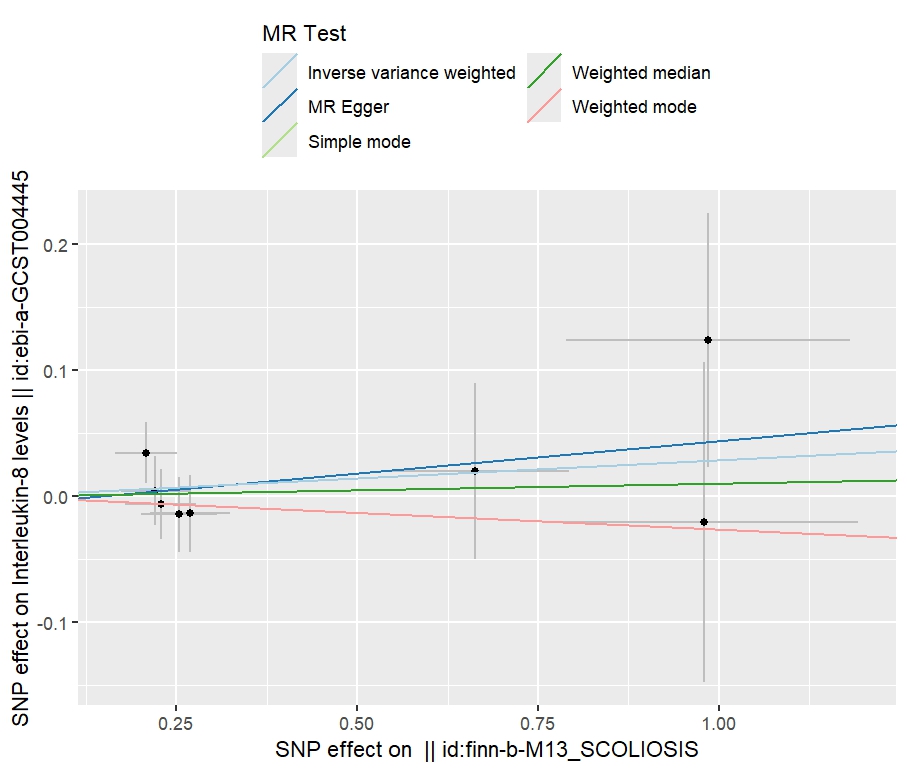

Supplement: Supplementary file 4 — Supplementary Material 4. [file JSP2-7-e70019-s002.zip › Supplementary Material 4/Exposureú║Scoliosisú1⁄4Outcomeú║inflammatory cytokines/IL8/Supplementary Material 4 IL8 1.jpeg]

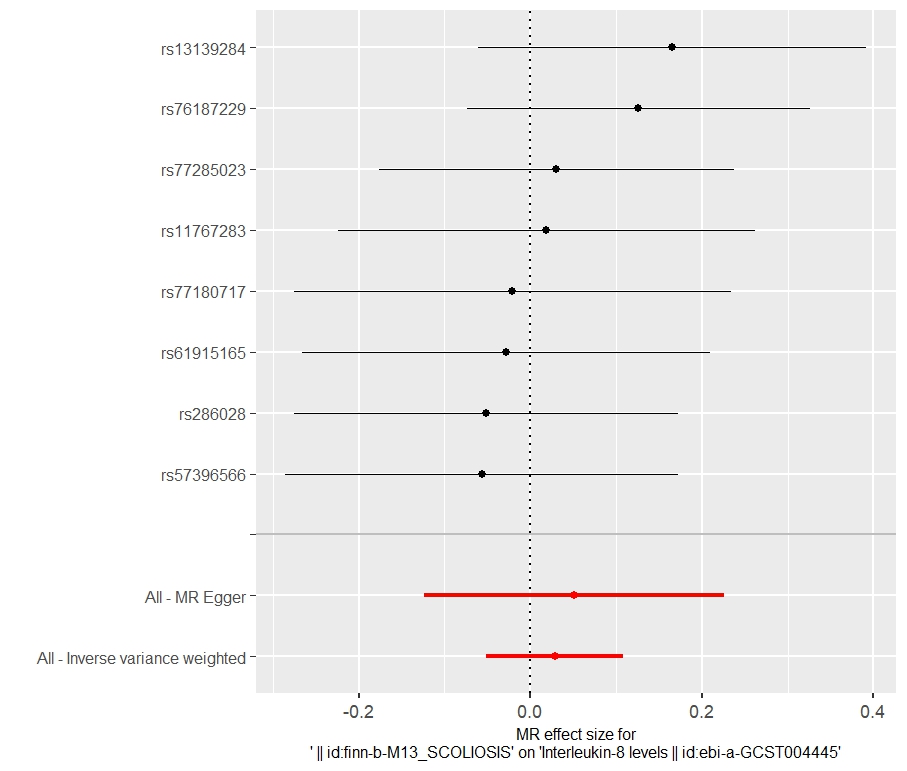

Supplement: Supplementary file 4 — Supplementary Material 4. [file JSP2-7-e70019-s002.zip › Supplementary Material 4/Exposureú║Scoliosisú1⁄4Outcomeú║inflammatory cytokines/IL8/Supplementary Material 4 IL8 2.jpeg]

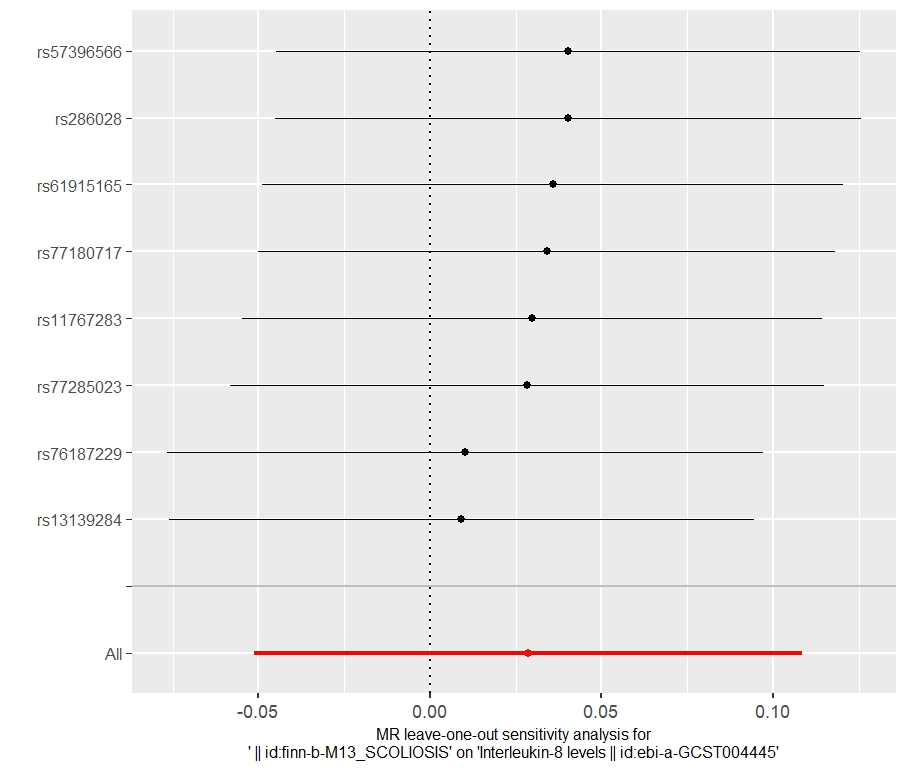

Supplement: Supplementary file 4 — Supplementary Material 4. [file JSP2-7-e70019-s002.zip › Supplementary Material 4/Exposureú║Scoliosisú1⁄4Outcomeú║inflammatory cytokines/IL8/Supplementary Material 4 IL8 3.jpeg]

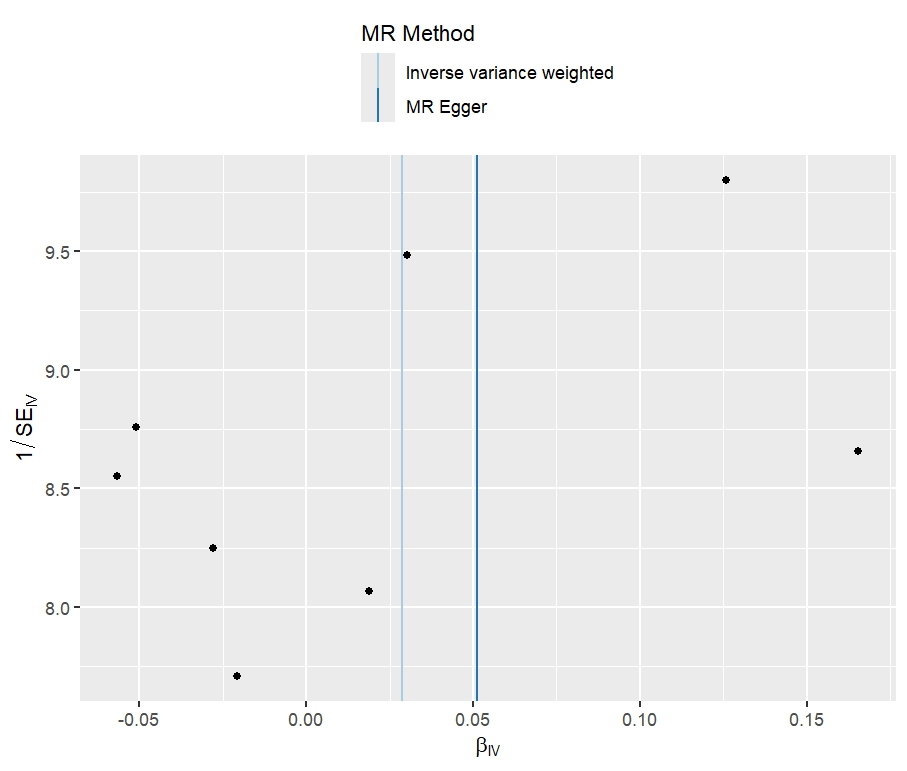

Supplement: Supplementary file 4 — Supplementary Material 4. [file JSP2-7-e70019-s002.zip › Supplementary Material 4/Exposureú║Scoliosisú1⁄4Outcomeú║inflammatory cytokines/IL8/Supplementary Material 4 IL8 4.jpeg]

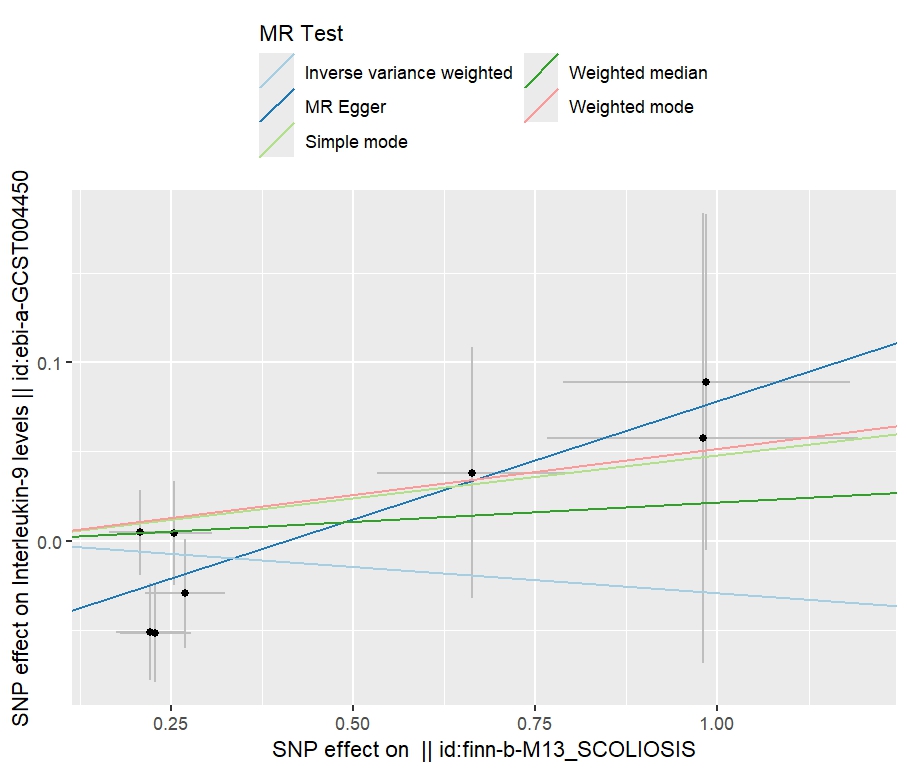

Supplement: Supplementary file 4 — Supplementary Material 4. [file JSP2-7-e70019-s002.zip › Supplementary Material 4/Exposureú║Scoliosisú1⁄4Outcomeú║inflammatory cytokines/IL9/Supplementary Material 4 IL9 1.jpeg]

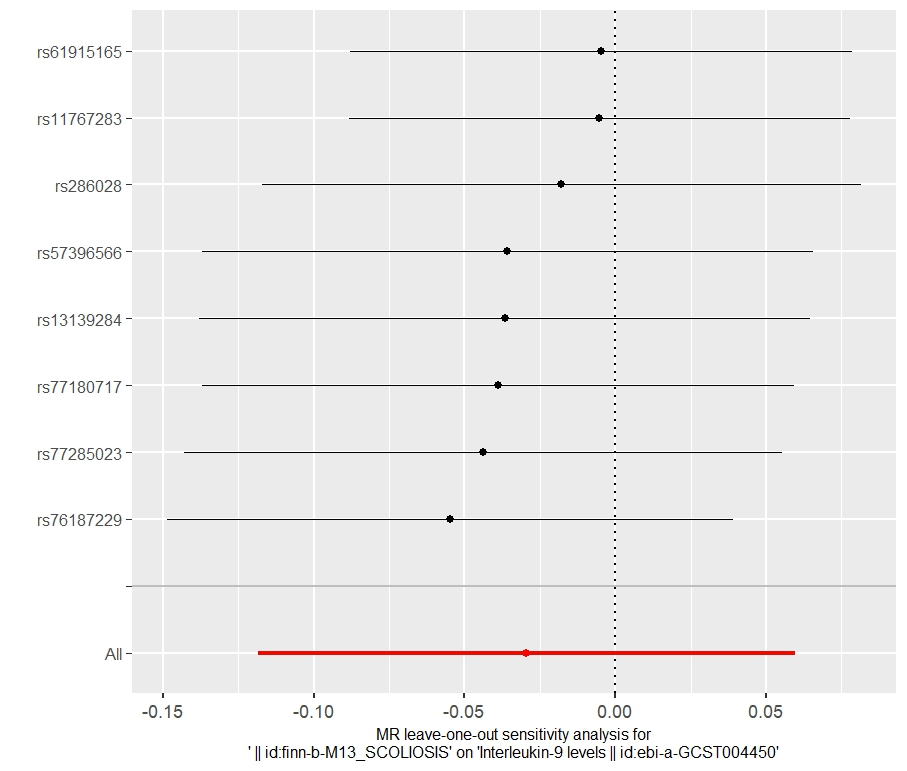

Supplement: Supplementary file 4 — Supplementary Material 4. [file JSP2-7-e70019-s002.zip › Supplementary Material 4/Exposureú║Scoliosisú1⁄4Outcomeú║inflammatory cytokines/IL9/Supplementary Material 4 IL9 2.jpeg]

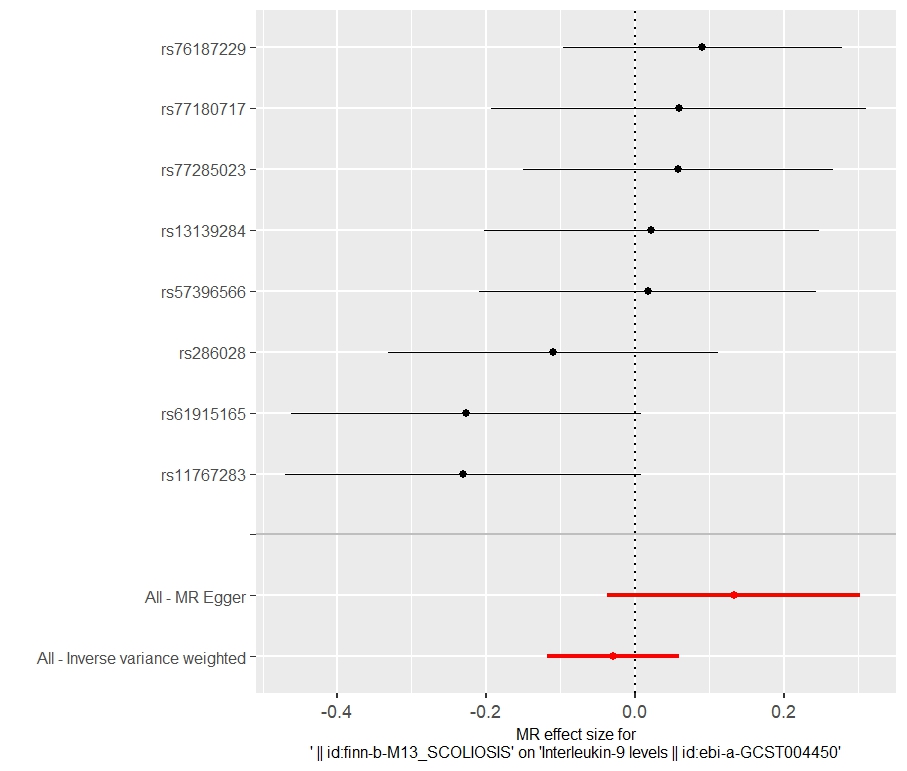

Supplement: Supplementary file 4 — Supplementary Material 4. [file JSP2-7-e70019-s002.zip › Supplementary Material 4/Exposureú║Scoliosisú1⁄4Outcomeú║inflammatory cytokines/IL9/Supplementary Material 4 IL9 3.jpeg]

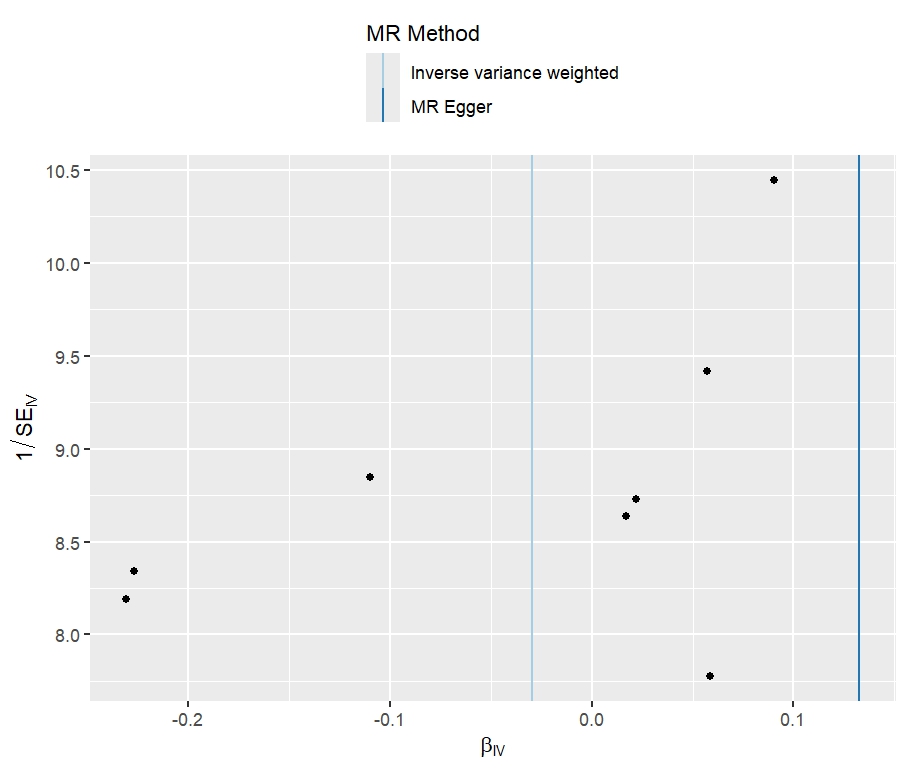

Supplement: Supplementary file 4 — Supplementary Material 4. [file JSP2-7-e70019-s002.zip › Supplementary Material 4/Exposureú║Scoliosisú1⁄4Outcomeú║inflammatory cytokines/IL9/Supplementary Material 4 IL9 4.jpeg]

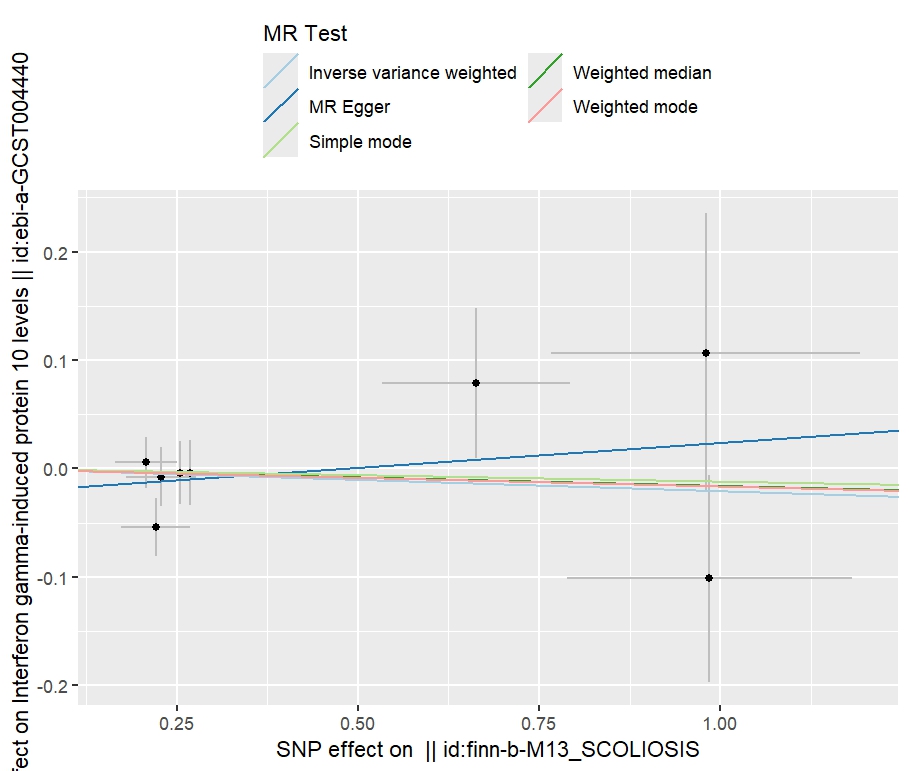

Supplement: Supplementary file 4 — Supplementary Material 4. [file JSP2-7-e70019-s002.zip › Supplementary Material 4/Exposureú║Scoliosisú1⁄4Outcomeú║inflammatory cytokines/IP10/Supplementary Material 4 IP10 1.jpeg]

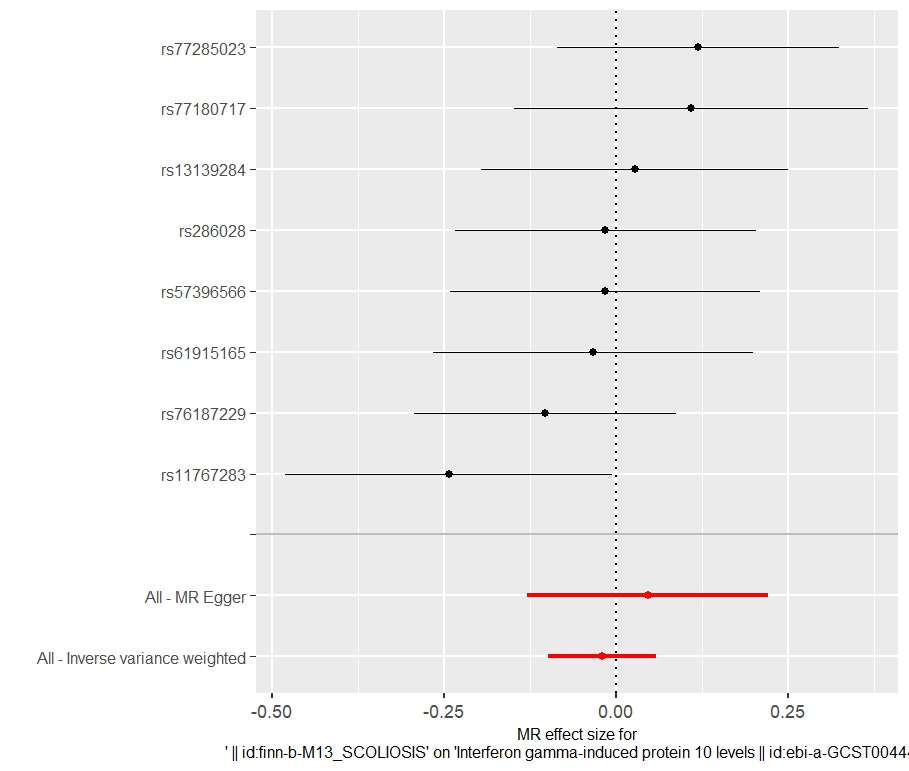

Supplement: Supplementary file 4 — Supplementary Material 4. [file JSP2-7-e70019-s002.zip › Supplementary Material 4/Exposureú║Scoliosisú1⁄4Outcomeú║inflammatory cytokines/IP10/Supplementary Material 4 IP10 2.jpeg]

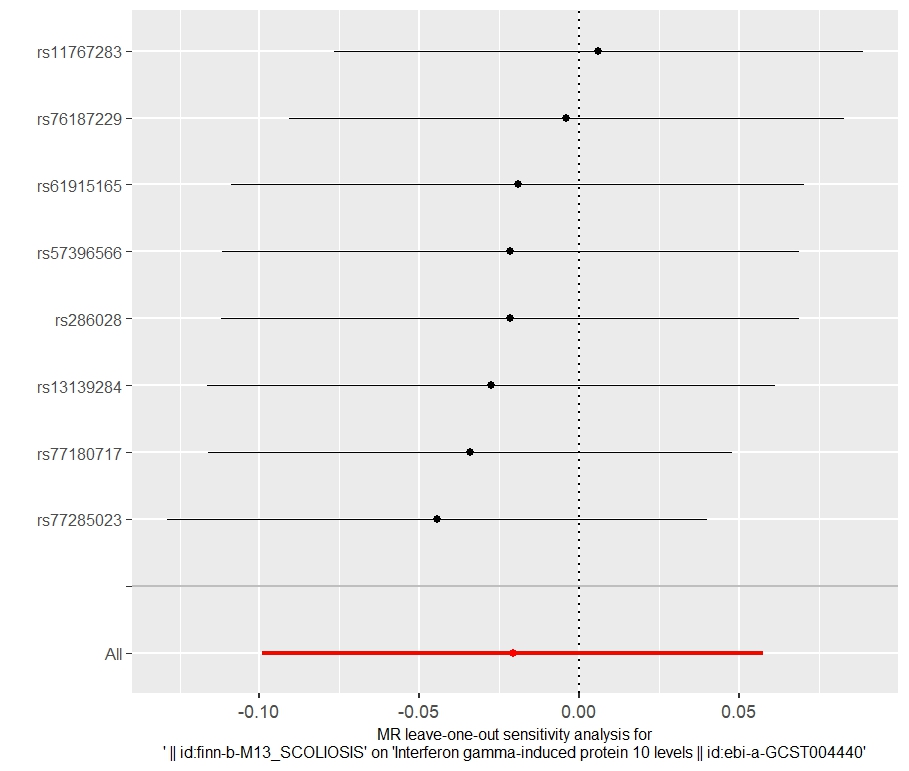

Supplement: Supplementary file 4 — Supplementary Material 4. [file JSP2-7-e70019-s002.zip › Supplementary Material 4/Exposureú║Scoliosisú1⁄4Outcomeú║inflammatory cytokines/IP10/Supplementary Material 4 IP10 3.jpeg]

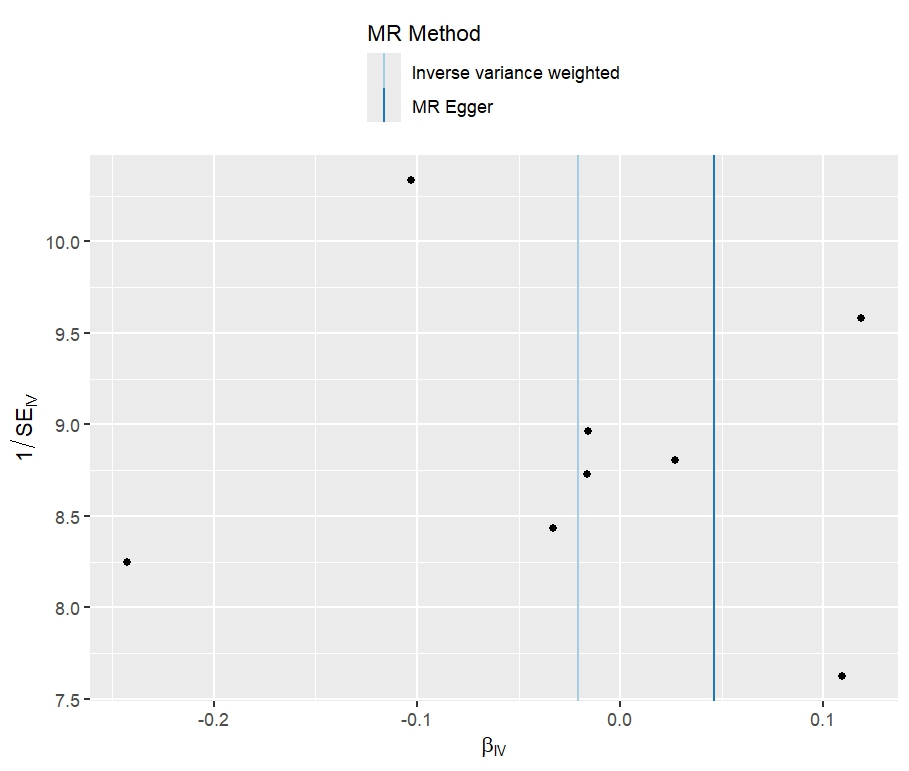

Supplement: Supplementary file 4 — Supplementary Material 4. [file JSP2-7-e70019-s002.zip › Supplementary Material 4/Exposureú║Scoliosisú1⁄4Outcomeú║inflammatory cytokines/IP10/Supplementary Material 4 IP10 4.jpeg]

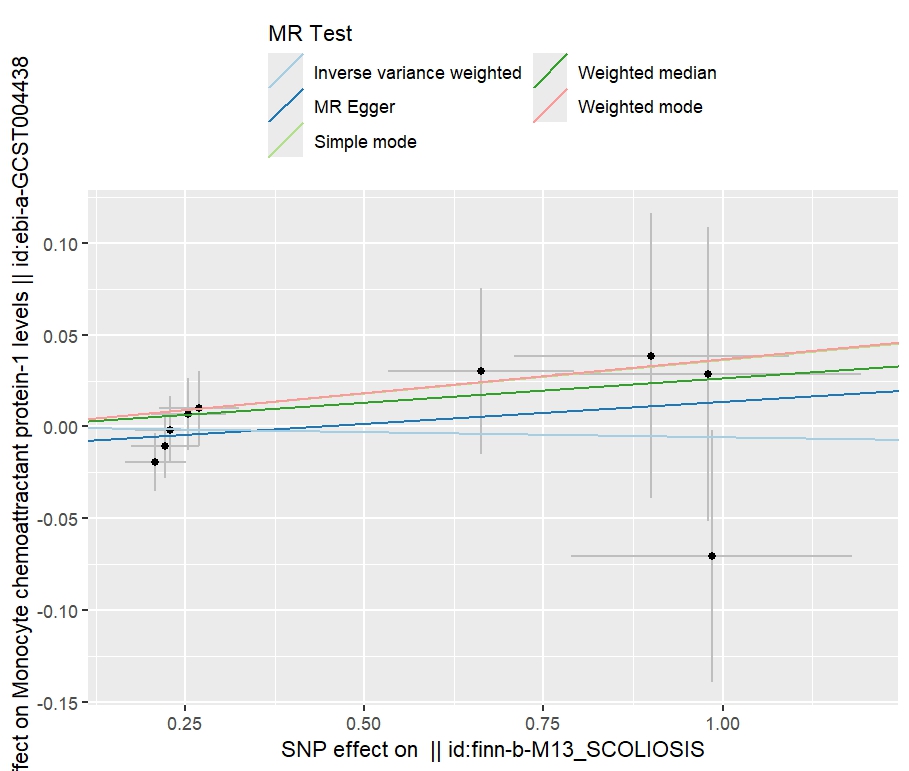

Supplement: Supplementary file 4 — Supplementary Material 4. [file JSP2-7-e70019-s002.zip › Supplementary Material 4/Exposureú║Scoliosisú1⁄4Outcomeú║inflammatory cytokines/MCP1/Supplementary Material 4 MCP1 1.jpeg]

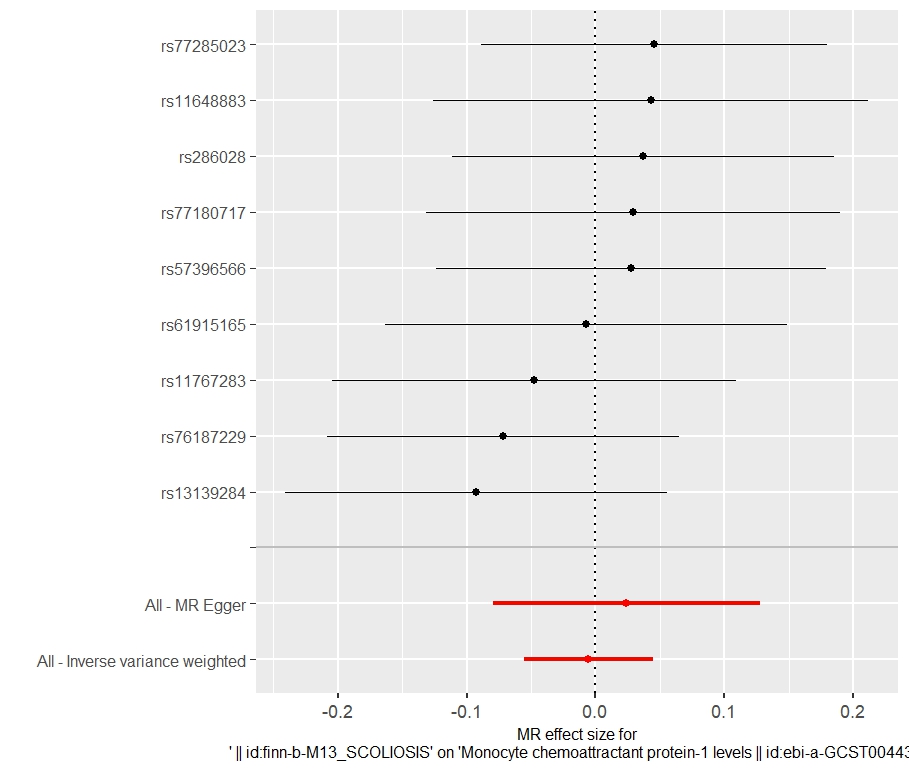

Supplement: Supplementary file 4 — Supplementary Material 4. [file JSP2-7-e70019-s002.zip › Supplementary Material 4/Exposureú║Scoliosisú1⁄4Outcomeú║inflammatory cytokines/MCP1/Supplementary Material 4 MCP1 2.jpeg]

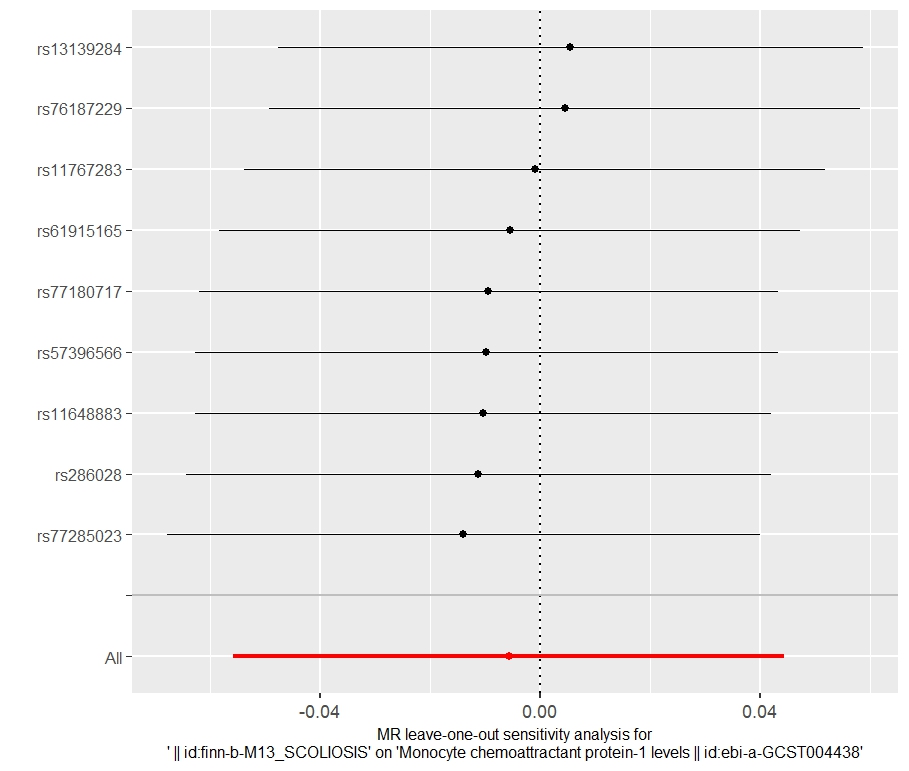

Supplement: Supplementary file 4 — Supplementary Material 4. [file JSP2-7-e70019-s002.zip › Supplementary Material 4/Exposureú║Scoliosisú1⁄4Outcomeú║inflammatory cytokines/MCP1/Supplementary Material 4 MCP1 3.jpeg]

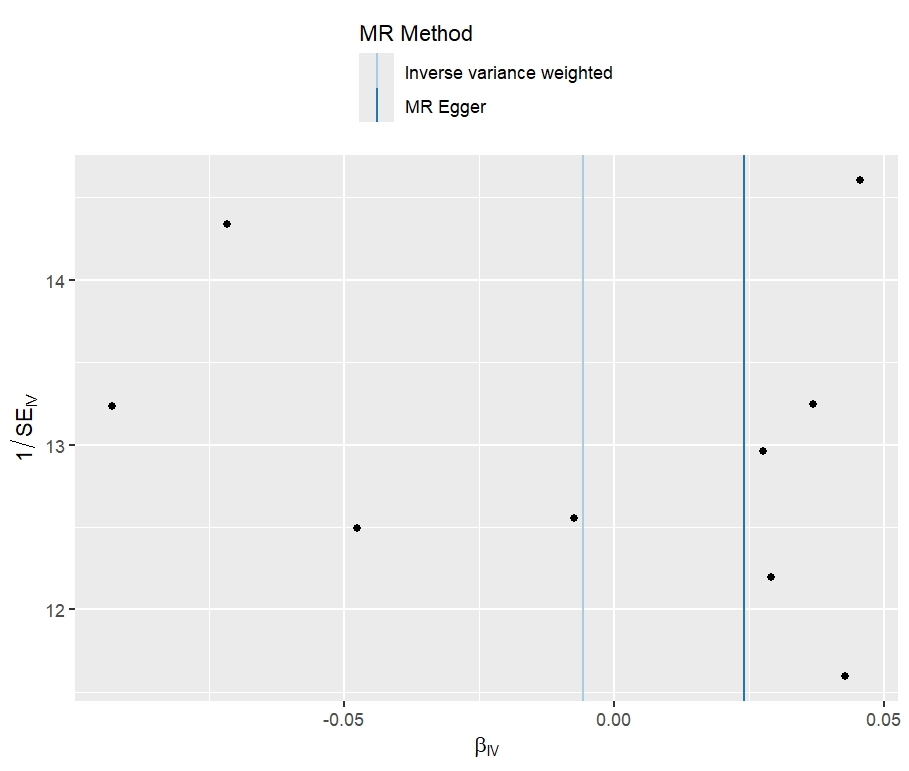

Supplement: Supplementary file 4 — Supplementary Material 4. [file JSP2-7-e70019-s002.zip › Supplementary Material 4/Exposureú║Scoliosisú1⁄4Outcomeú║inflammatory cytokines/MCP1/Supplementary Material 4 MCP1 4.jpeg]

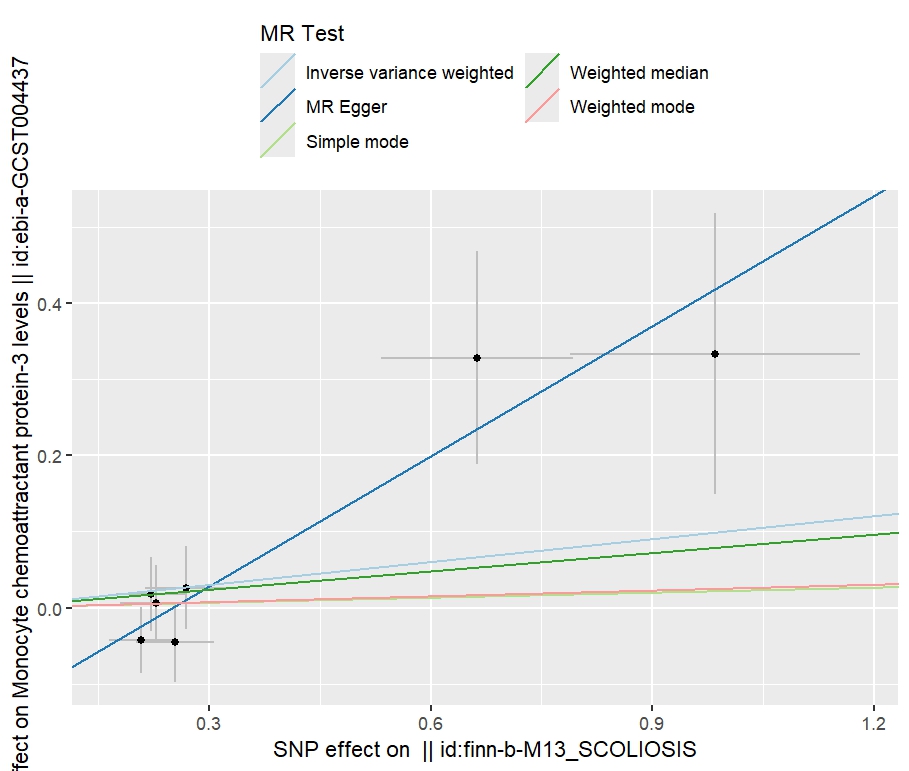

Supplement: Supplementary file 4 — Supplementary Material 4. [file JSP2-7-e70019-s002.zip › Supplementary Material 4/Exposureú║Scoliosisú1⁄4Outcomeú║inflammatory cytokines/MCP3/Supplementary Material 4 MCP3 1.jpeg]

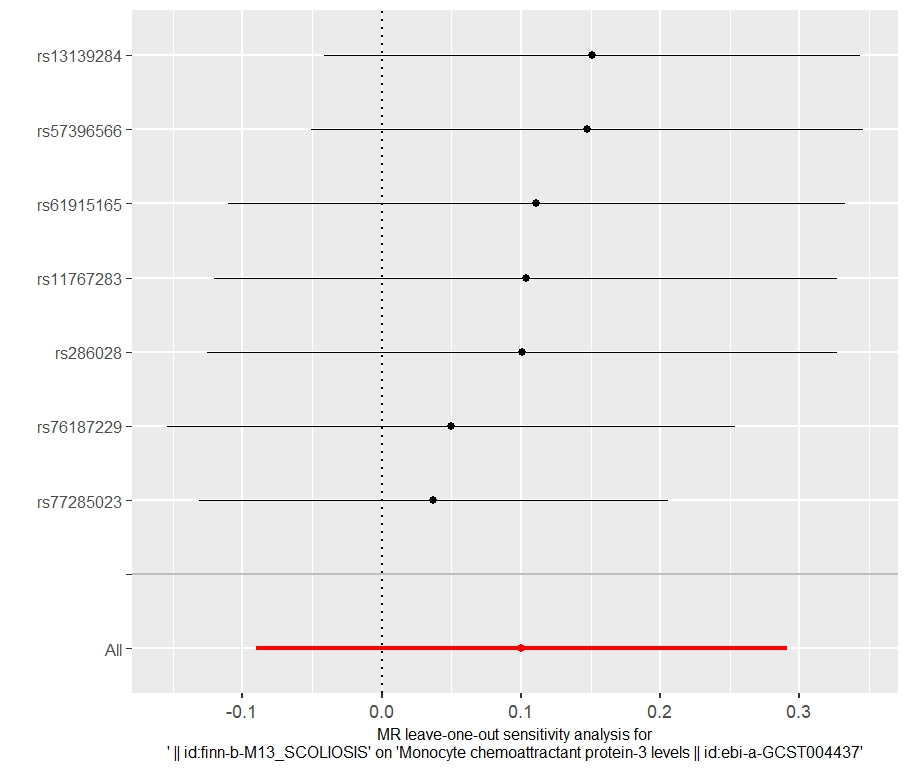

Supplement: Supplementary file 4 — Supplementary Material 4. [file JSP2-7-e70019-s002.zip › Supplementary Material 4/Exposureú║Scoliosisú1⁄4Outcomeú║inflammatory cytokines/MCP3/Supplementary Material 4 MCP3 2.jpeg]

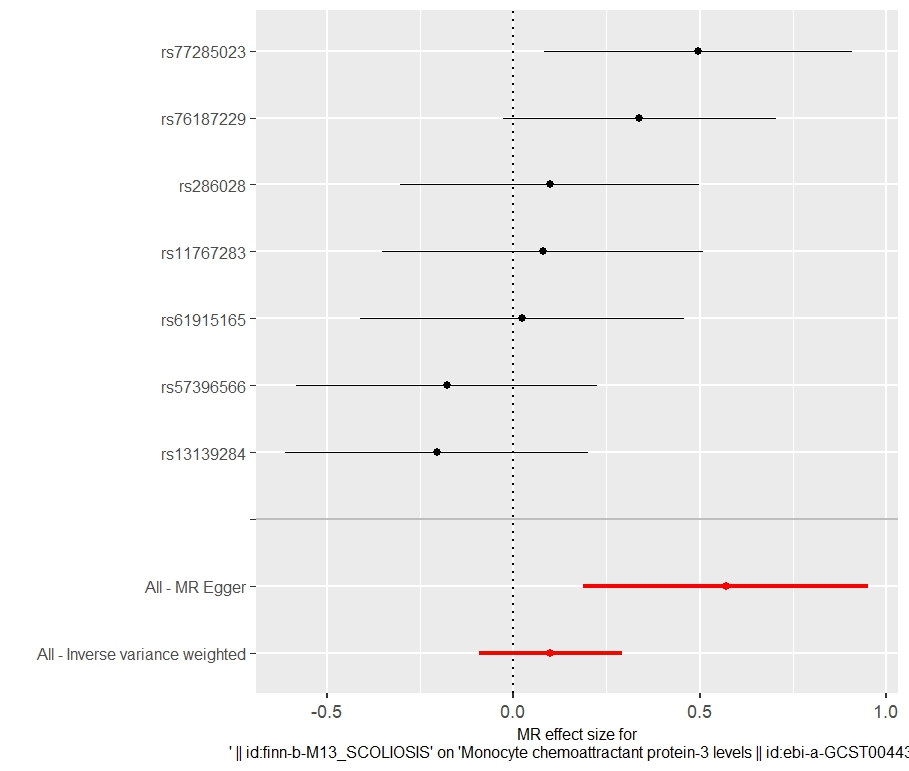

Supplement: Supplementary file 4 — Supplementary Material 4. [file JSP2-7-e70019-s002.zip › Supplementary Material 4/Exposureú║Scoliosisú1⁄4Outcomeú║inflammatory cytokines/MCP3/Supplementary Material 4 MCP3 3.jpeg]

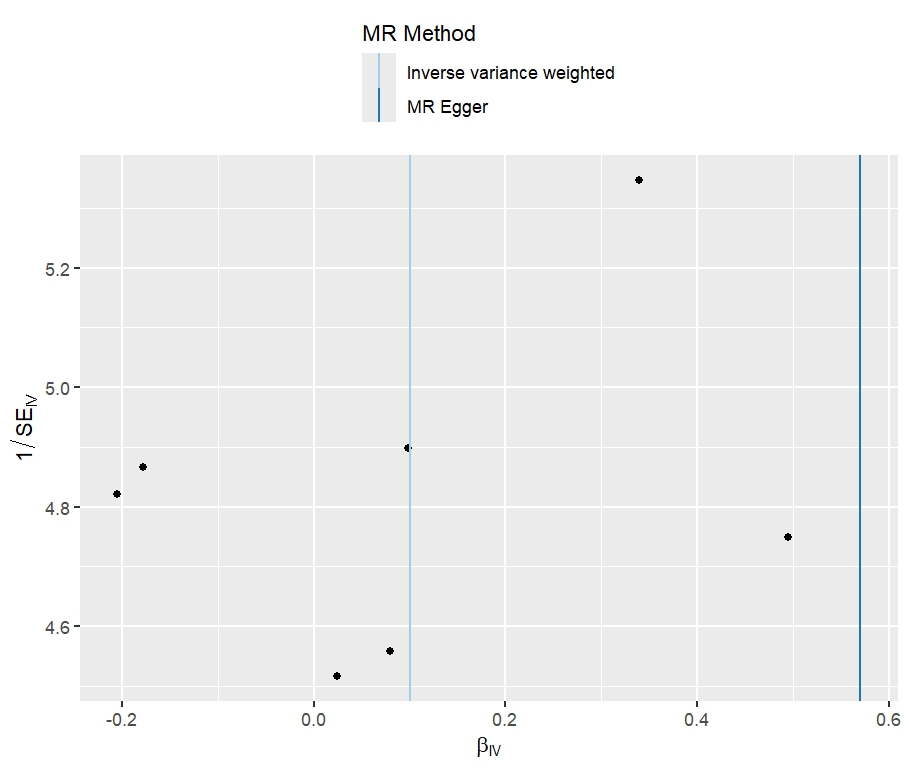

Supplement: Supplementary file 4 — Supplementary Material 4. [file JSP2-7-e70019-s002.zip › Supplementary Material 4/Exposureú║Scoliosisú1⁄4Outcomeú║inflammatory cytokines/MCP3/Supplementary Material 4 MCP3 4.jpeg]

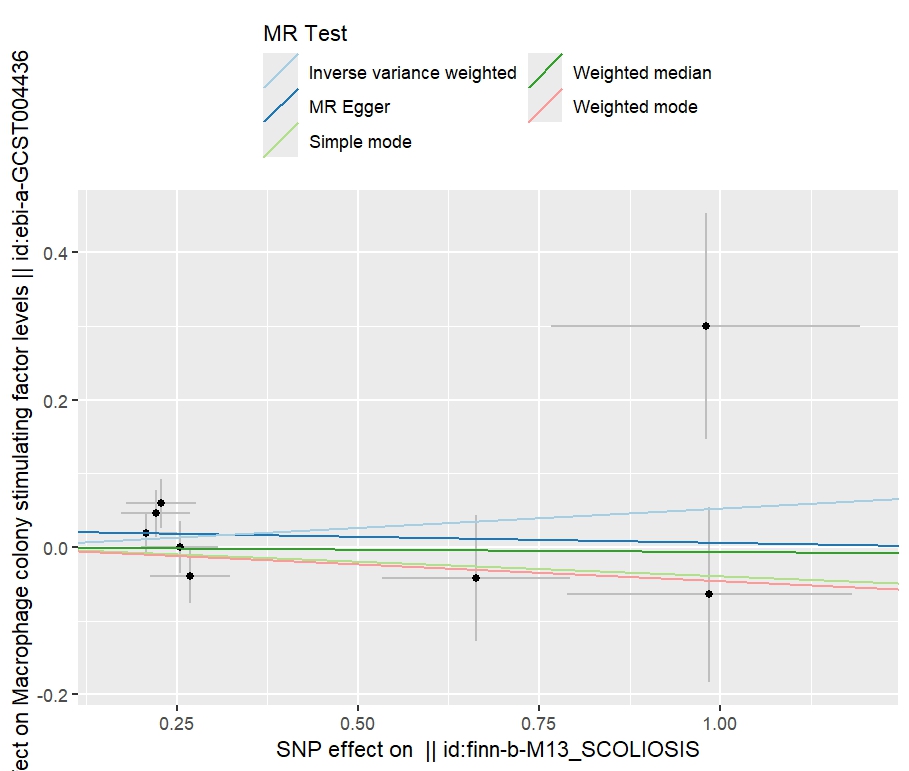

Supplement: Supplementary file 4 — Supplementary Material 4. [file JSP2-7-e70019-s002.zip › Supplementary Material 4/Exposureú║Scoliosisú1⁄4Outcomeú║inflammatory cytokines/MCSF/Supplementary Material 4 MCSF 1.jpeg]

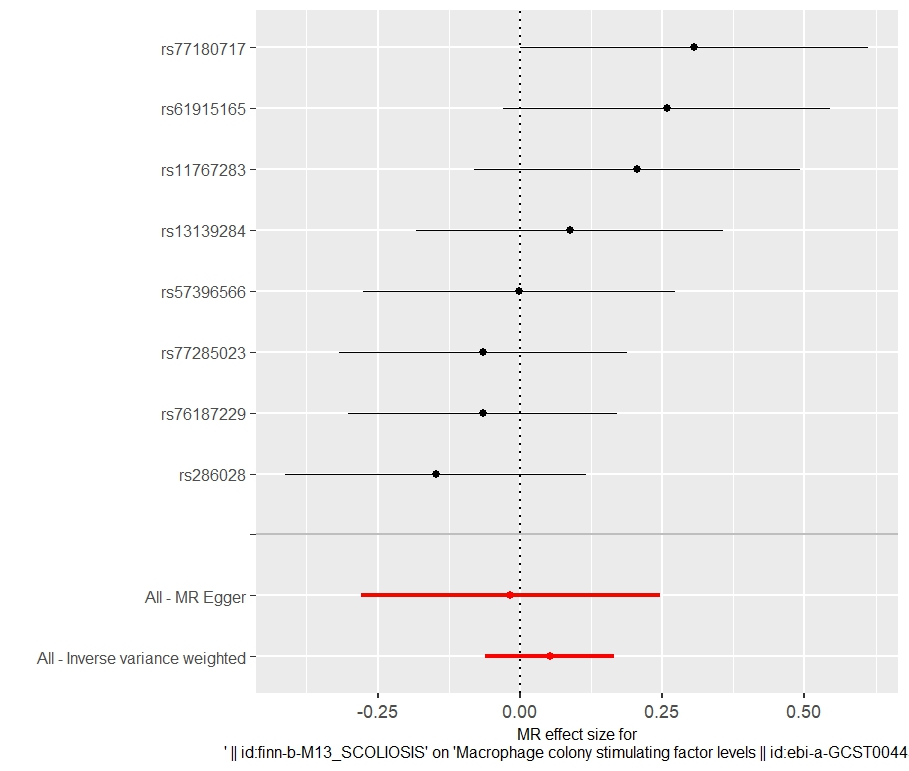

Supplement: Supplementary file 4 — Supplementary Material 4. [file JSP2-7-e70019-s002.zip › Supplementary Material 4/Exposureú║Scoliosisú1⁄4Outcomeú║inflammatory cytokines/MCSF/Supplementary Material 4 MCSF 2.jpeg]

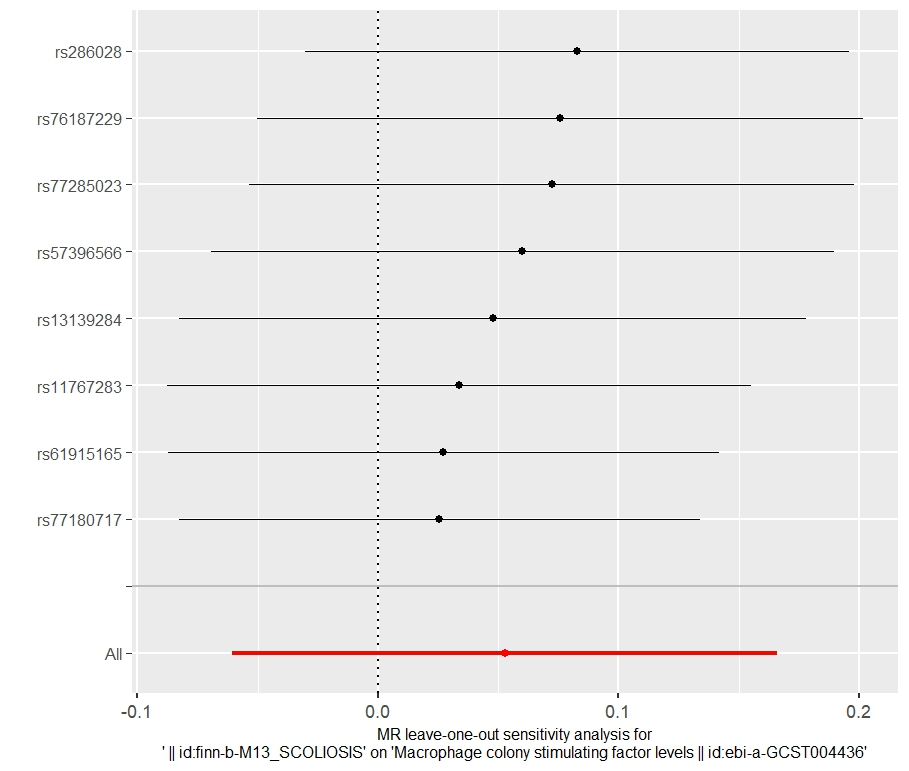

Supplement: Supplementary file 4 — Supplementary Material 4. [file JSP2-7-e70019-s002.zip › Supplementary Material 4/Exposureú║Scoliosisú1⁄4Outcomeú║inflammatory cytokines/MCSF/Supplementary Material 4 MCSF 3.jpeg]

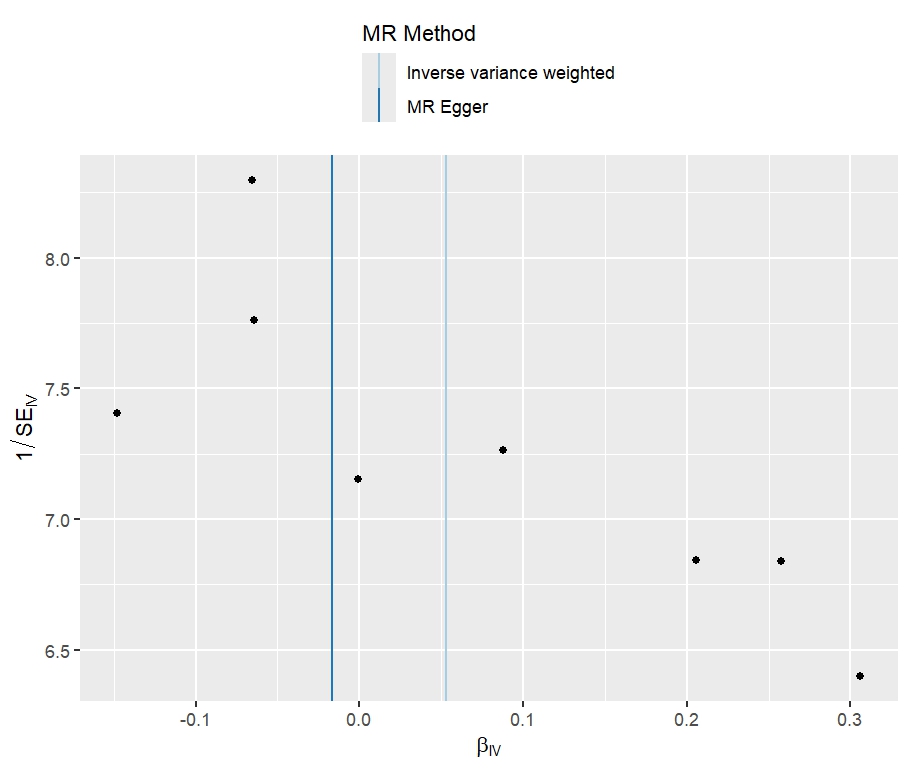

Supplement: Supplementary file 4 — Supplementary Material 4. [file JSP2-7-e70019-s002.zip › Supplementary Material 4/Exposureú║Scoliosisú1⁄4Outcomeú║inflammatory cytokines/MCSF/Supplementary Material 4 MCSF 4.jpeg]

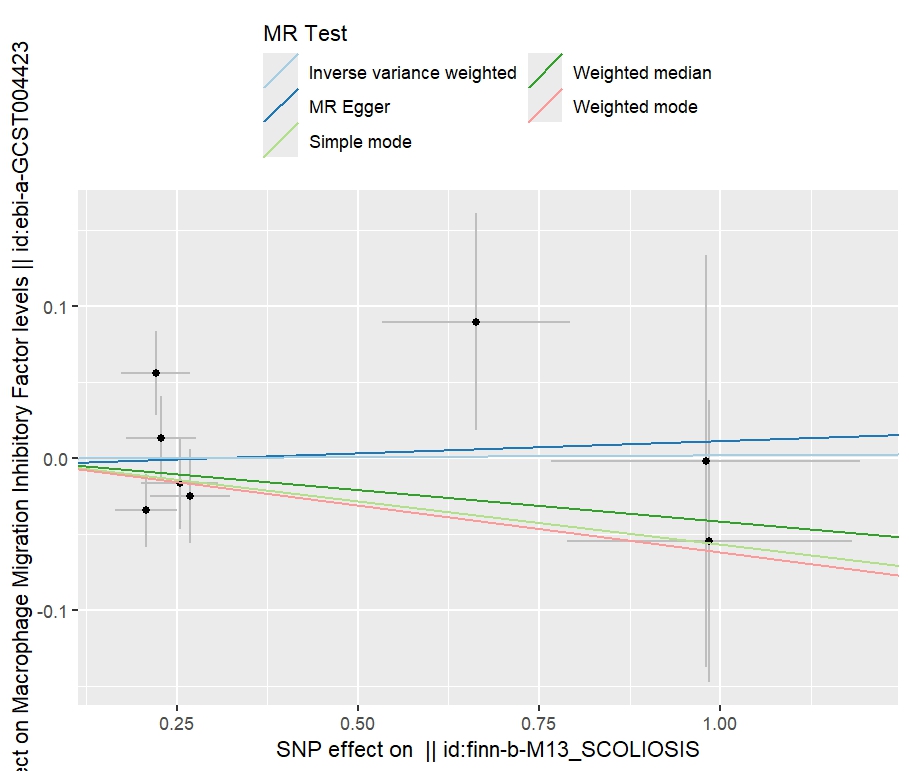

Supplement: Supplementary file 4 — Supplementary Material 4. [file JSP2-7-e70019-s002.zip › Supplementary Material 4/Exposureú║Scoliosisú1⁄4Outcomeú║inflammatory cytokines/MIF/Supplementary Material 4 MIF 1.jpeg]

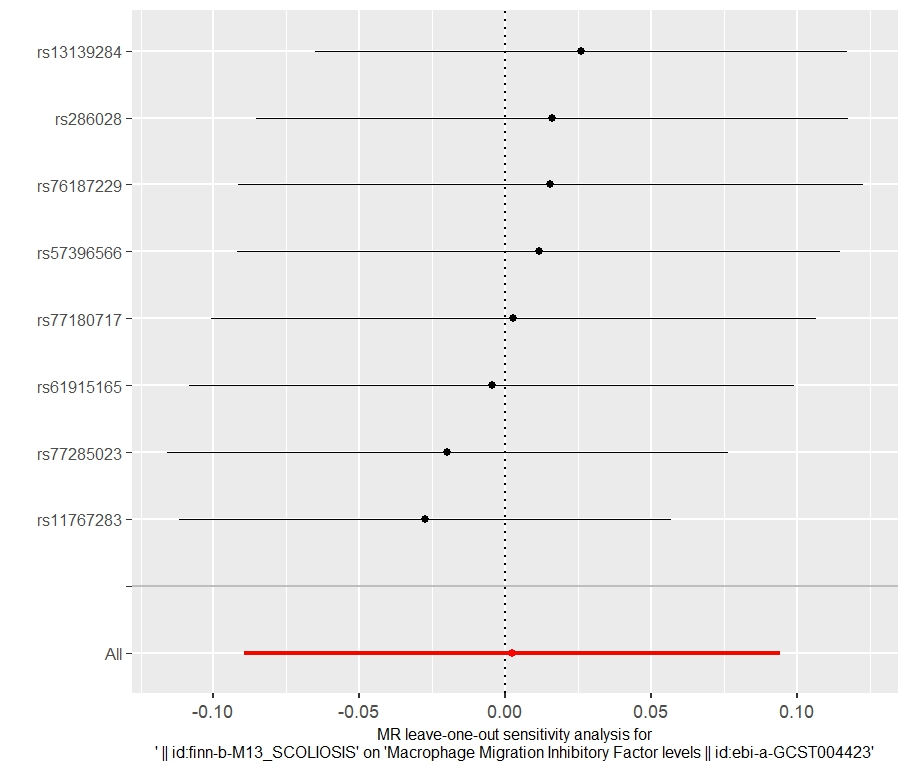

Supplement: Supplementary file 4 — Supplementary Material 4. [file JSP2-7-e70019-s002.zip › Supplementary Material 4/Exposureú║Scoliosisú1⁄4Outcomeú║inflammatory cytokines/MIF/Supplementary Material 4 MIF 2.jpeg]

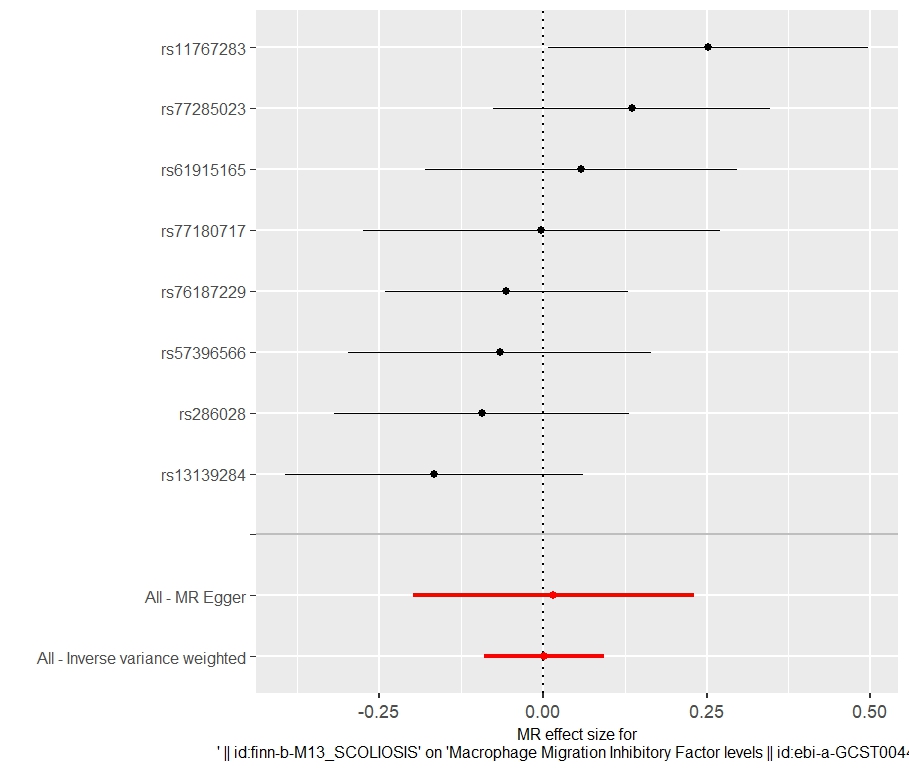

Supplement: Supplementary file 4 — Supplementary Material 4. [file JSP2-7-e70019-s002.zip › Supplementary Material 4/Exposureú║Scoliosisú1⁄4Outcomeú║inflammatory cytokines/MIF/Supplementary Material 4 MIF 3.jpeg]

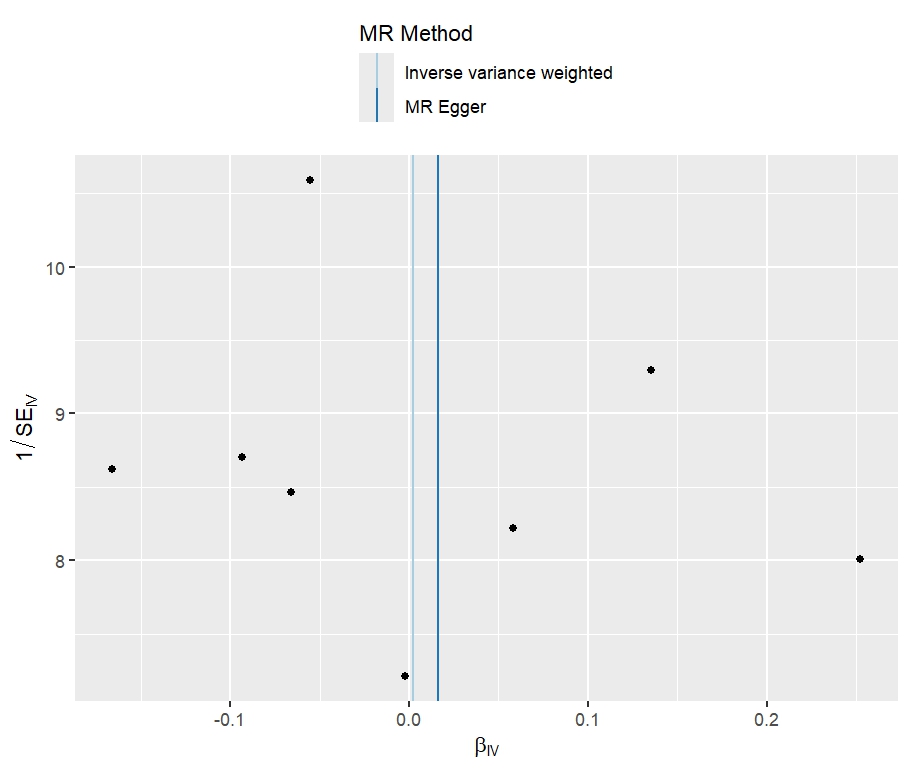

Supplement: Supplementary file 4 — Supplementary Material 4. [file JSP2-7-e70019-s002.zip › Supplementary Material 4/Exposureú║Scoliosisú1⁄4Outcomeú║inflammatory cytokines/MIF/Supplementary Material 4 MIF 4.jpeg]

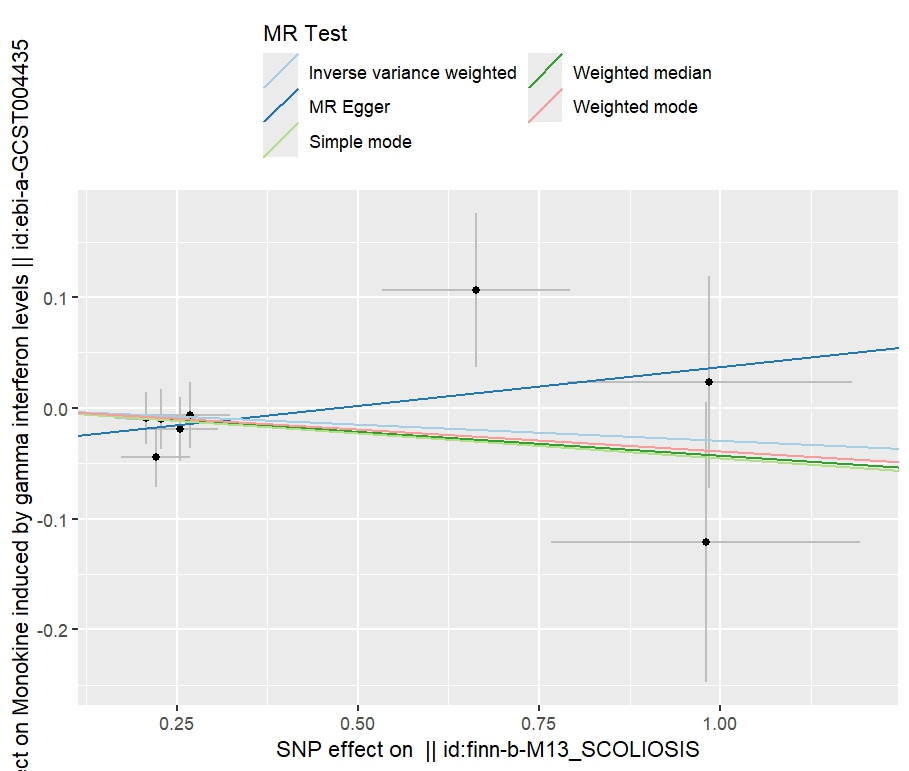

Supplement: Supplementary file 4 — Supplementary Material 4. [file JSP2-7-e70019-s002.zip › Supplementary Material 4/Exposureú║Scoliosisú1⁄4Outcomeú║inflammatory cytokines/MIG/Supplementary Material 4 MIG 1.jpeg]

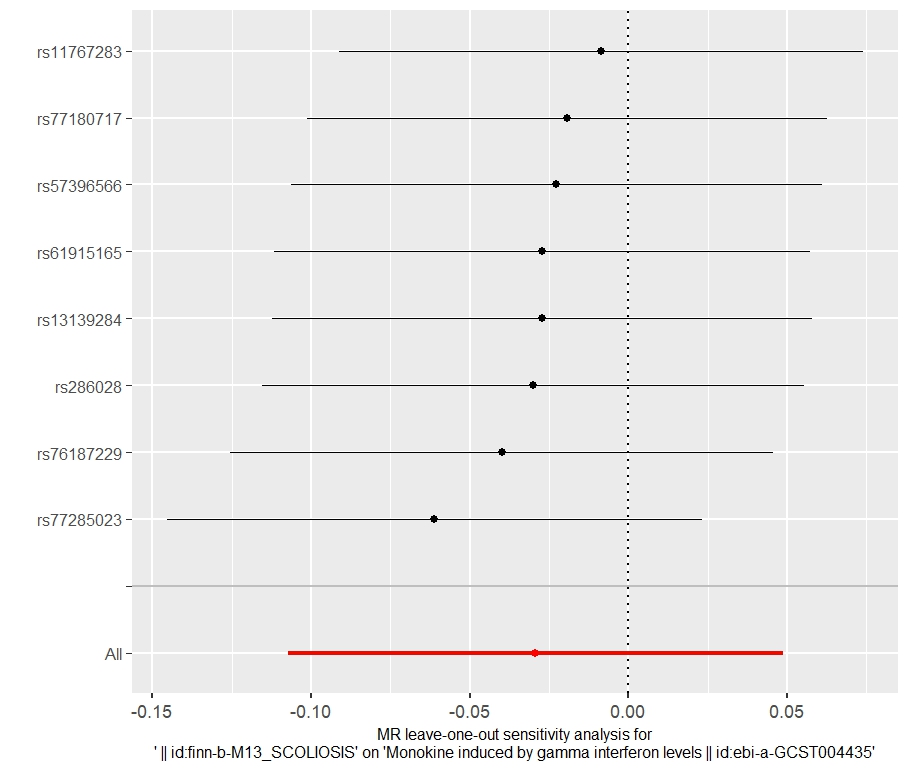

Supplement: Supplementary file 4 — Supplementary Material 4. [file JSP2-7-e70019-s002.zip › Supplementary Material 4/Exposureú║Scoliosisú1⁄4Outcomeú║inflammatory cytokines/MIG/Supplementary Material 4 MIG 2.jpeg]

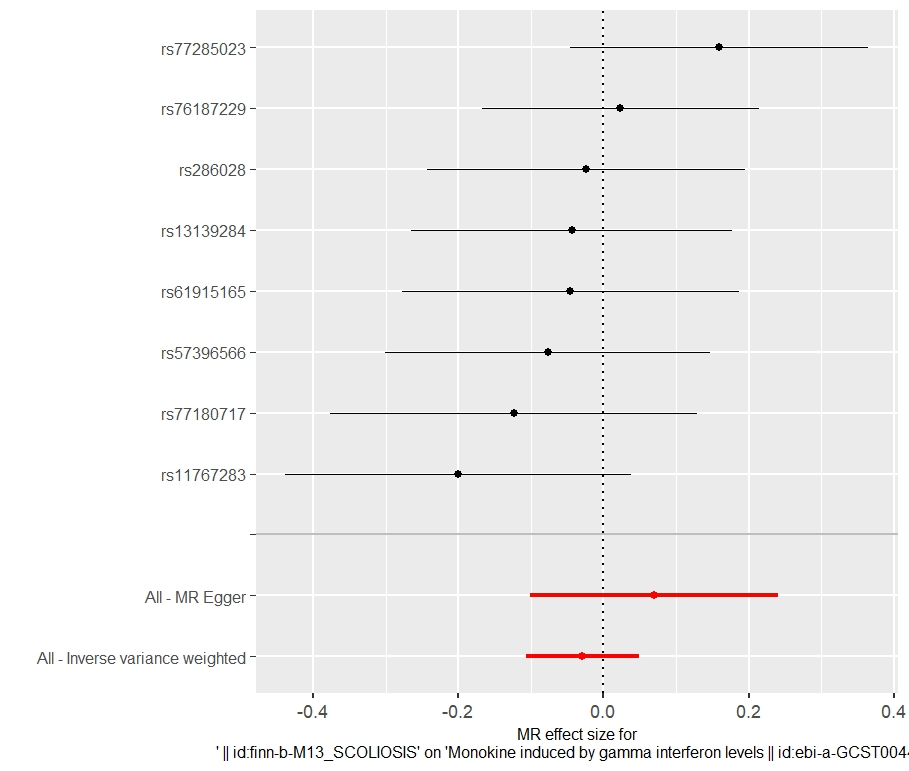

Supplement: Supplementary file 4 — Supplementary Material 4. [file JSP2-7-e70019-s002.zip › Supplementary Material 4/Exposureú║Scoliosisú1⁄4Outcomeú║inflammatory cytokines/MIG/Supplementary Material 4 MIG 3.jpeg]

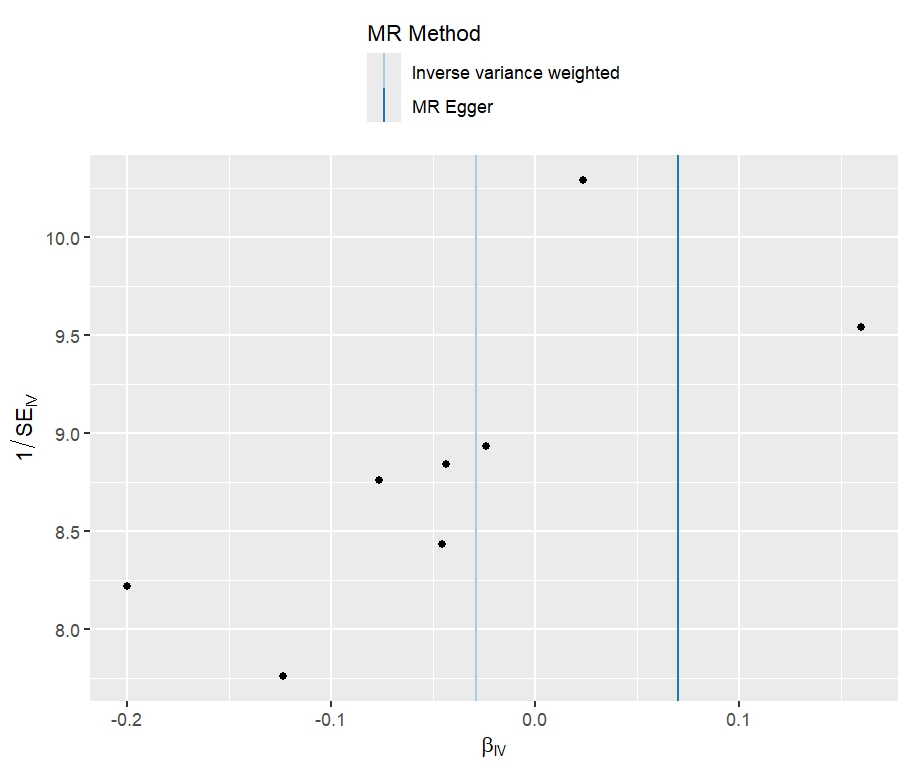

Supplement: Supplementary file 4 — Supplementary Material 4. [file JSP2-7-e70019-s002.zip › Supplementary Material 4/Exposureú║Scoliosisú1⁄4Outcomeú║inflammatory cytokines/MIG/Supplementary Material 4 MIG 4.jpeg]

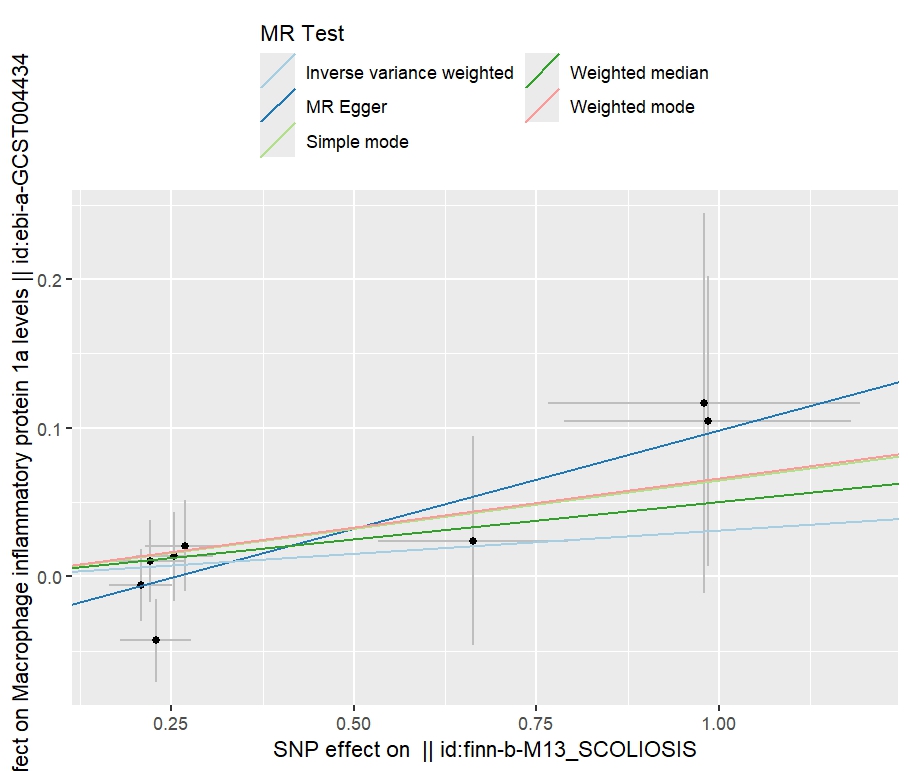

Supplement: Supplementary file 4 — Supplementary Material 4. [file JSP2-7-e70019-s002.zip › Supplementary Material 4/Exposureú║Scoliosisú1⁄4Outcomeú║inflammatory cytokines/MIP1 A/Supplementary Material 4 MIP1 A 1.jpeg]

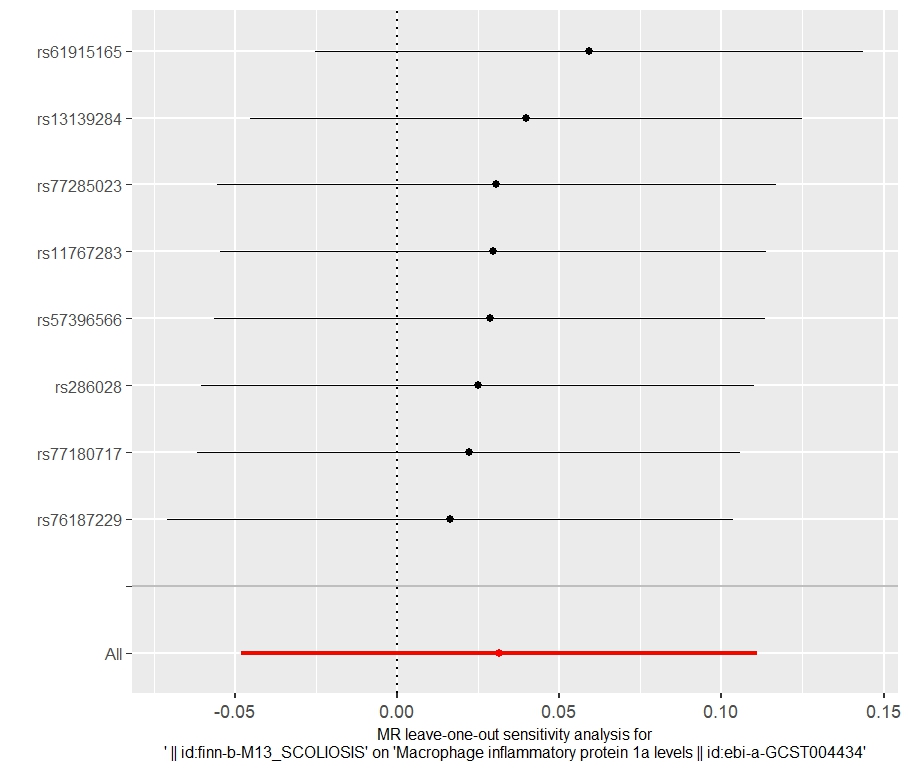

Supplement: Supplementary file 4 — Supplementary Material 4. [file JSP2-7-e70019-s002.zip › Supplementary Material 4/Exposureú║Scoliosisú1⁄4Outcomeú║inflammatory cytokines/MIP1 A/Supplementary Material 4 MIP1 A 2.jpeg]

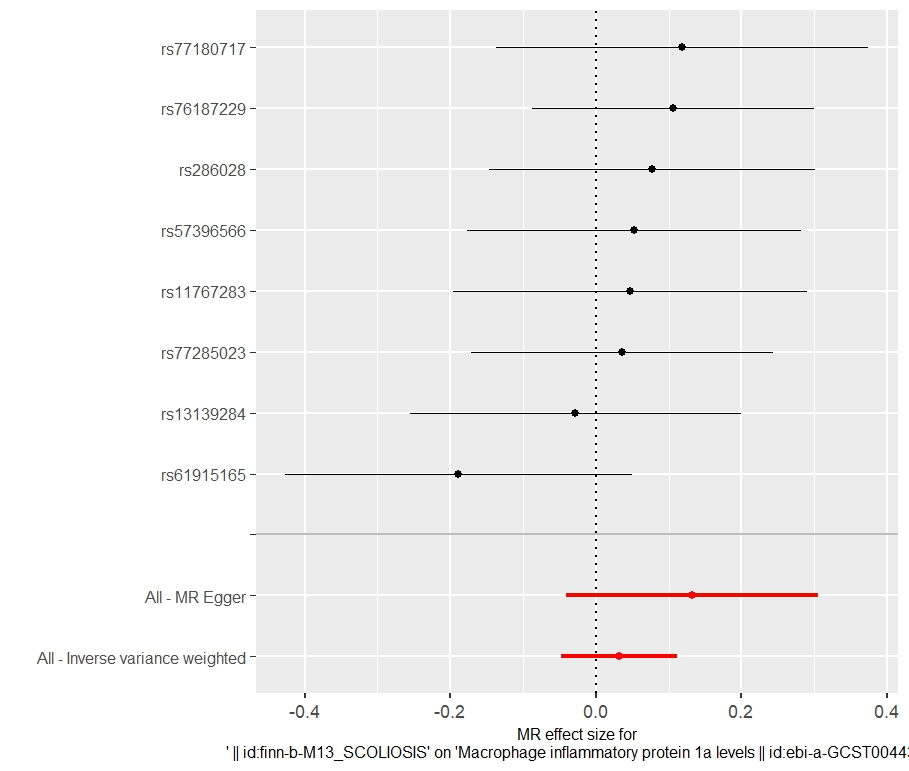

Supplement: Supplementary file 4 — Supplementary Material 4. [file JSP2-7-e70019-s002.zip › Supplementary Material 4/Exposureú║Scoliosisú1⁄4Outcomeú║inflammatory cytokines/MIP1 A/Supplementary Material 4 MIP1 A 3.jpeg]

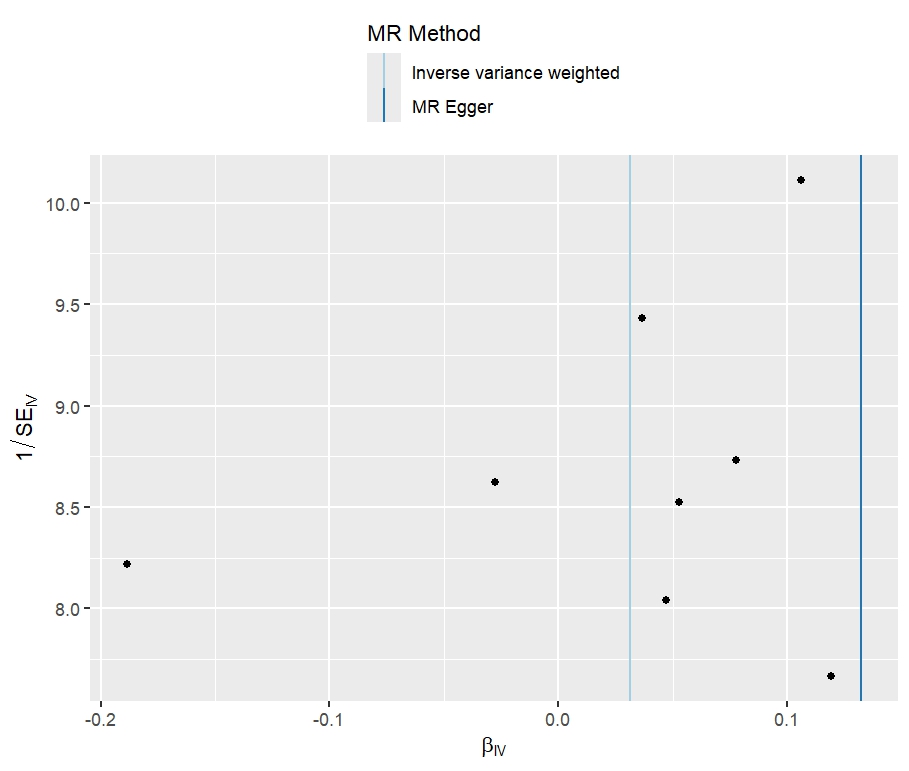

Supplement: Supplementary file 4 — Supplementary Material 4. [file JSP2-7-e70019-s002.zip › Supplementary Material 4/Exposureú║Scoliosisú1⁄4Outcomeú║inflammatory cytokines/MIP1 A/Supplementary Material 4 MIP1 A 4.jpeg]

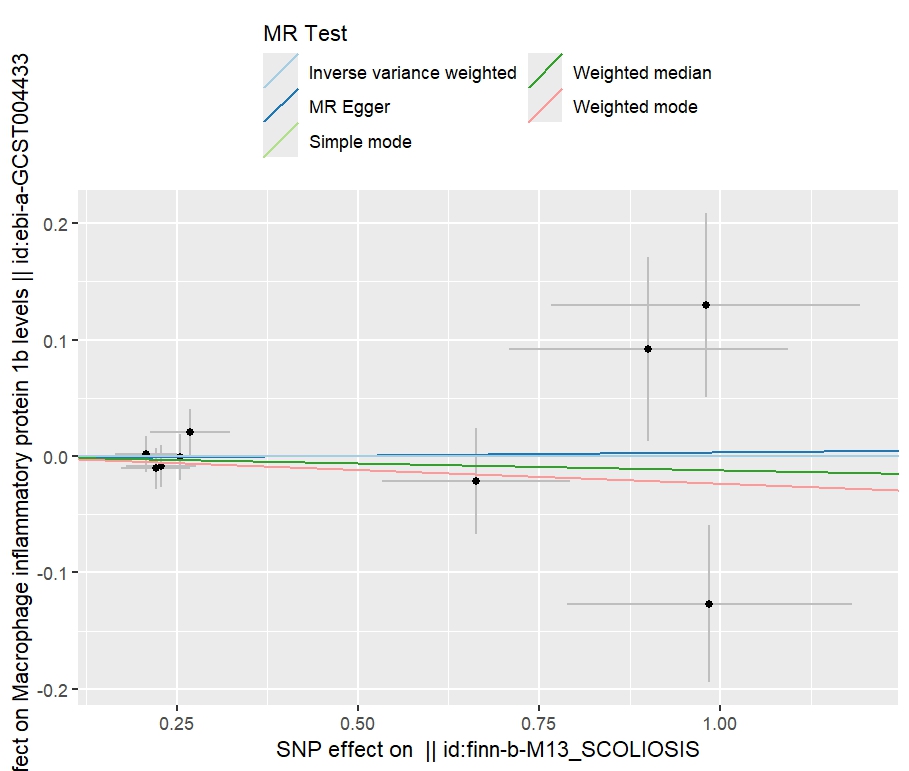

Supplement: Supplementary file 4 — Supplementary Material 4. [file JSP2-7-e70019-s002.zip › Supplementary Material 4/Exposureú║Scoliosisú1⁄4Outcomeú║inflammatory cytokines/MIP1 B/Supplementary Material 4 MIP1 B 1.jpeg]

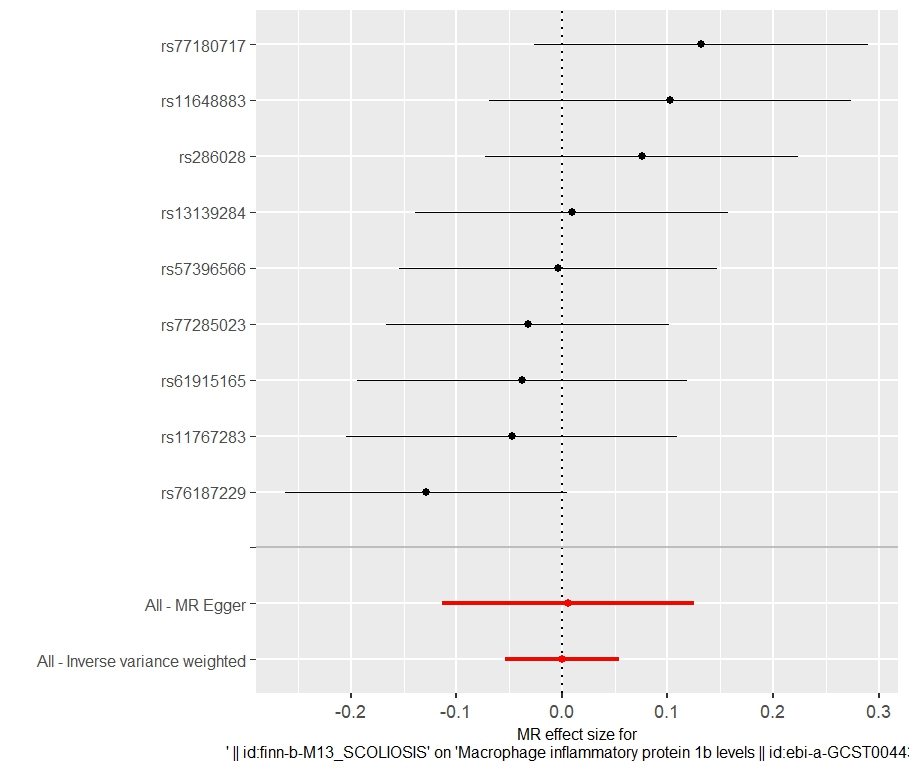

Supplement: Supplementary file 4 — Supplementary Material 4. [file JSP2-7-e70019-s002.zip › Supplementary Material 4/Exposureú║Scoliosisú1⁄4Outcomeú║inflammatory cytokines/MIP1 B/Supplementary Material 4 MIP1 B 2.jpeg]

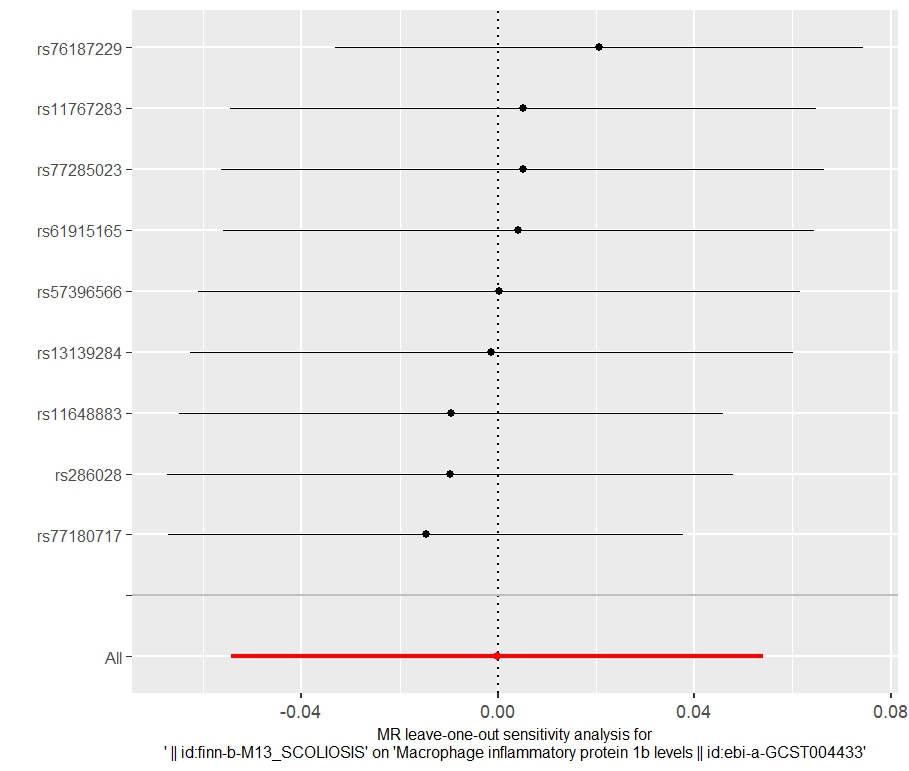

Supplement: Supplementary file 4 — Supplementary Material 4. [file JSP2-7-e70019-s002.zip › Supplementary Material 4/Exposureú║Scoliosisú1⁄4Outcomeú║inflammatory cytokines/MIP1 B/Supplementary Material 4 MIP1 B 3.jpeg]

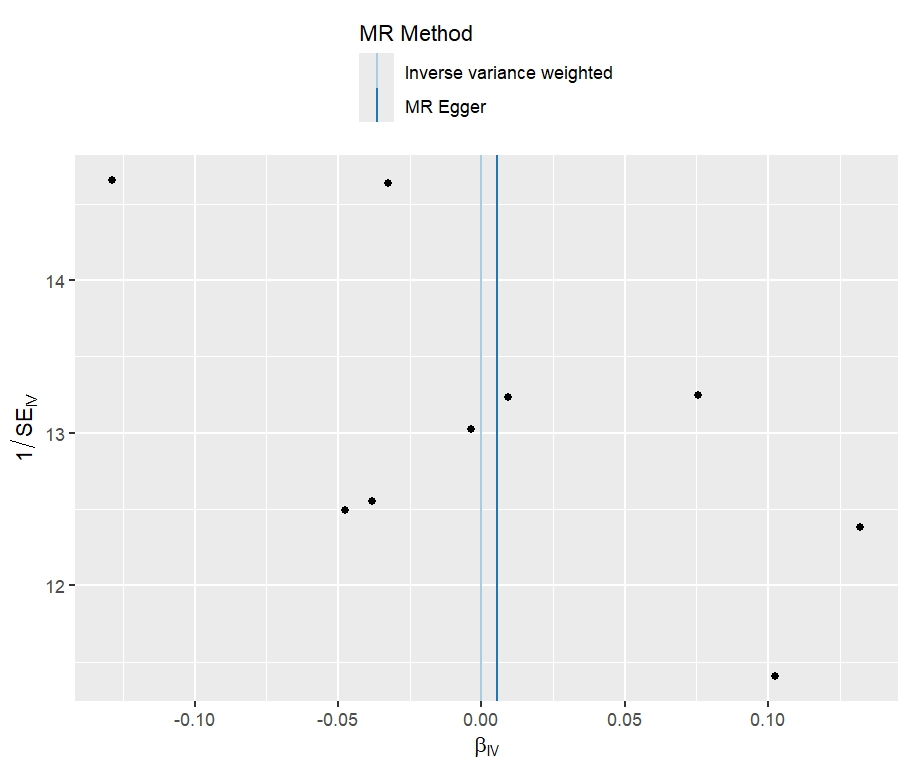

Supplement: Supplementary file 4 — Supplementary Material 4. [file JSP2-7-e70019-s002.zip › Supplementary Material 4/Exposureú║Scoliosisú1⁄4Outcomeú║inflammatory cytokines/MIP1 B/Supplementary Material 4 MIP1 B 4.jpeg]

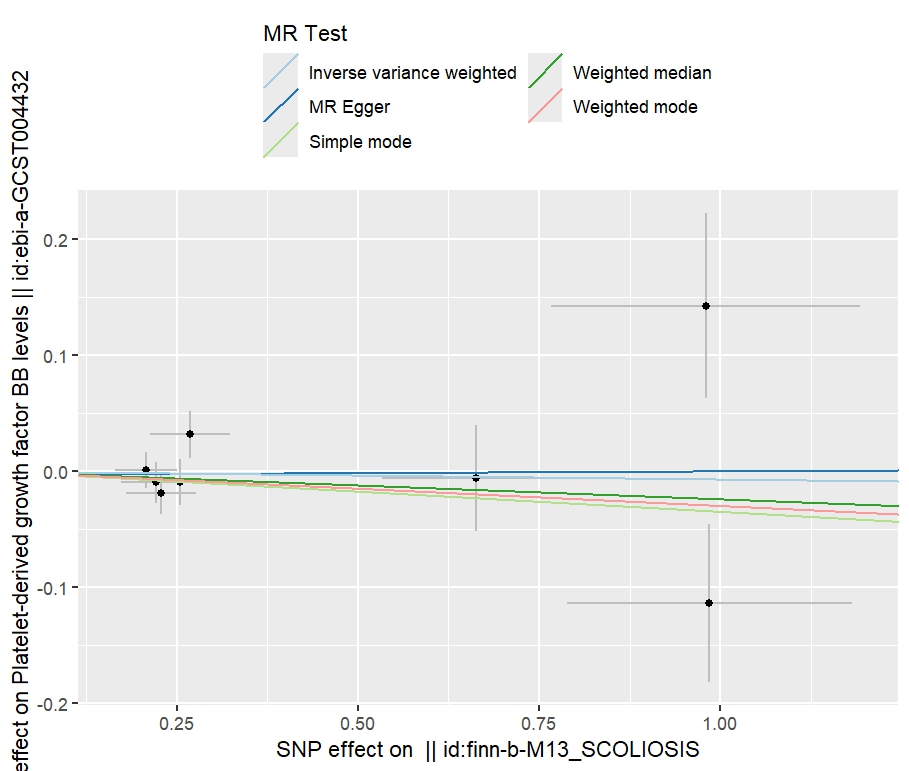

Supplement: Supplementary file 4 — Supplementary Material 4. [file JSP2-7-e70019-s002.zip › Supplementary Material 4/Exposureú║Scoliosisú1⁄4Outcomeú║inflammatory cytokines/PDGFbb/Supplementary Material 4 PDGFbb 1.jpeg]

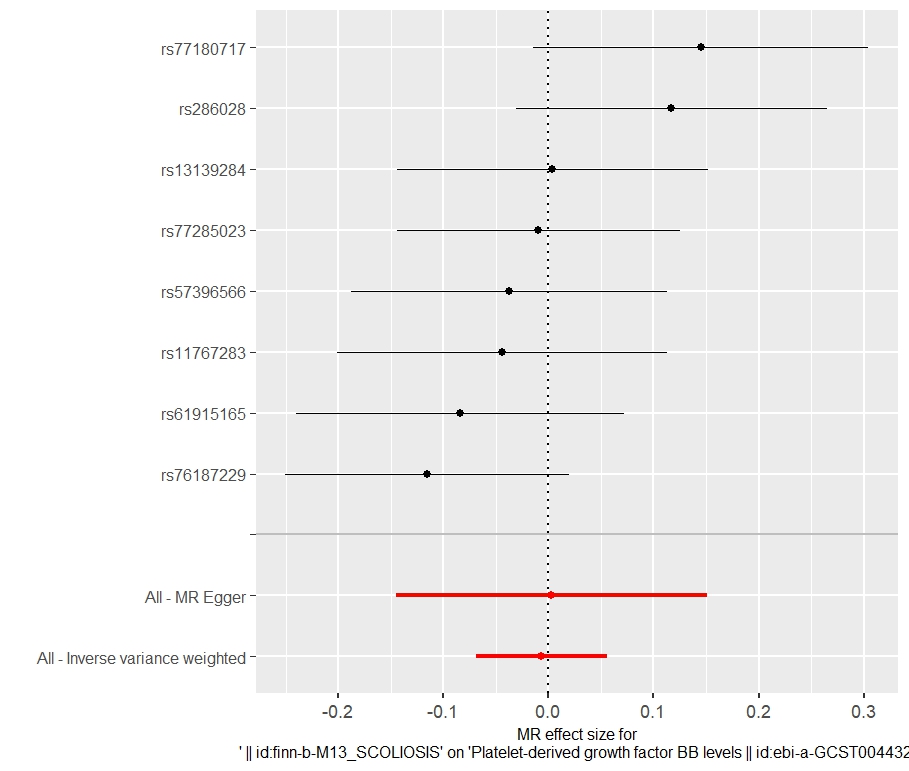

Supplement: Supplementary file 4 — Supplementary Material 4. [file JSP2-7-e70019-s002.zip › Supplementary Material 4/Exposureú║Scoliosisú1⁄4Outcomeú║inflammatory cytokines/PDGFbb/Supplementary Material 4 PDGFbb 2.jpeg]

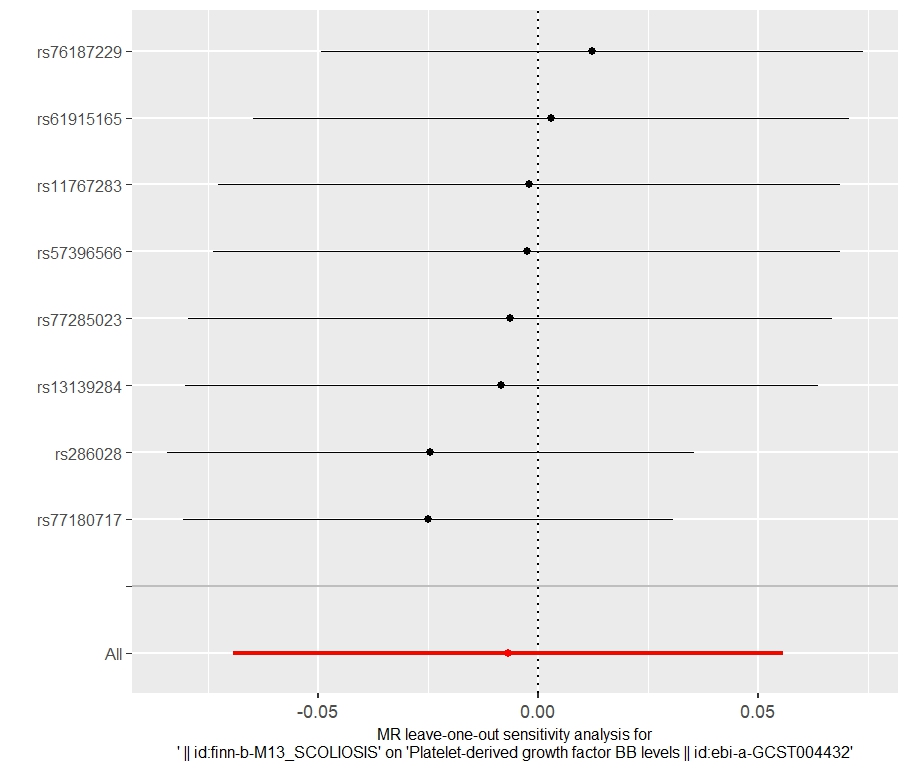

Supplement: Supplementary file 4 — Supplementary Material 4. [file JSP2-7-e70019-s002.zip › Supplementary Material 4/Exposureú║Scoliosisú1⁄4Outcomeú║inflammatory cytokines/PDGFbb/Supplementary Material 4 PDGFbb 3.jpeg]

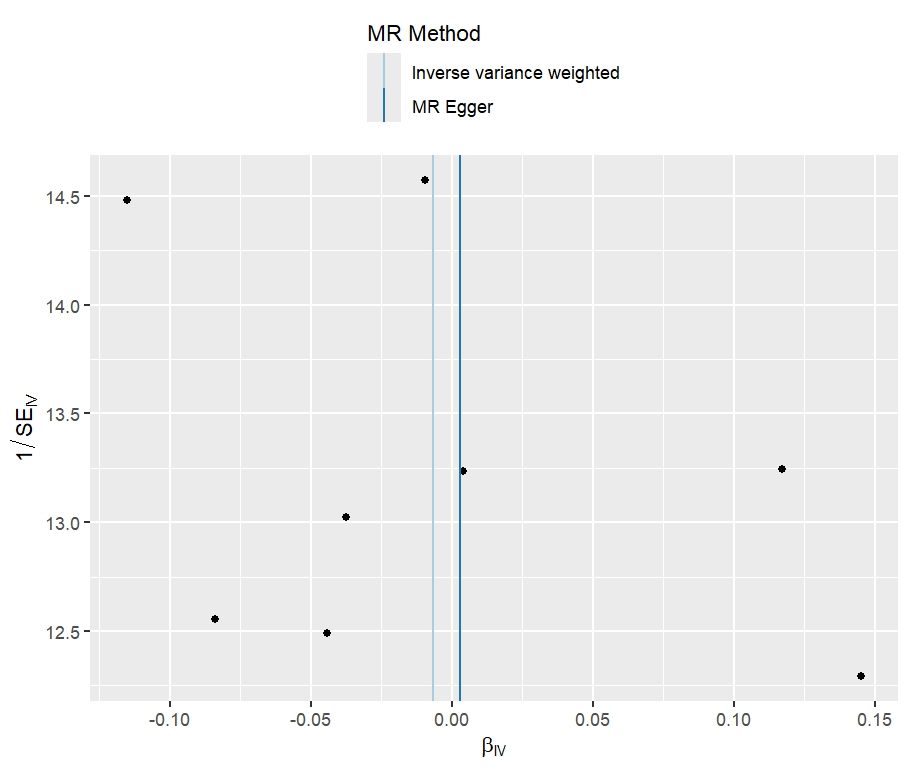

Supplement: Supplementary file 4 — Supplementary Material 4. [file JSP2-7-e70019-s002.zip › Supplementary Material 4/Exposureú║Scoliosisú1⁄4Outcomeú║inflammatory cytokines/PDGFbb/Supplementary Material 4 PDGFbb 4.jpeg]

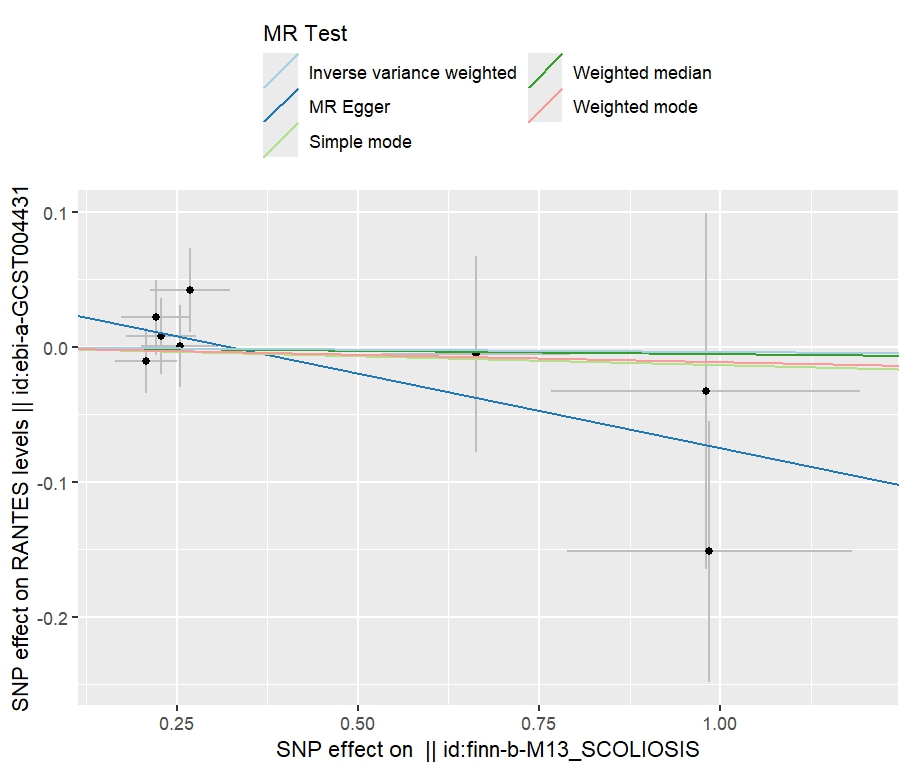

Supplement: Supplementary file 4 — Supplementary Material 4. [file JSP2-7-e70019-s002.zip › Supplementary Material 4/Exposureú║Scoliosisú1⁄4Outcomeú║inflammatory cytokines/RANTES/Supplementary Material 4 RANTES 1.jpeg]

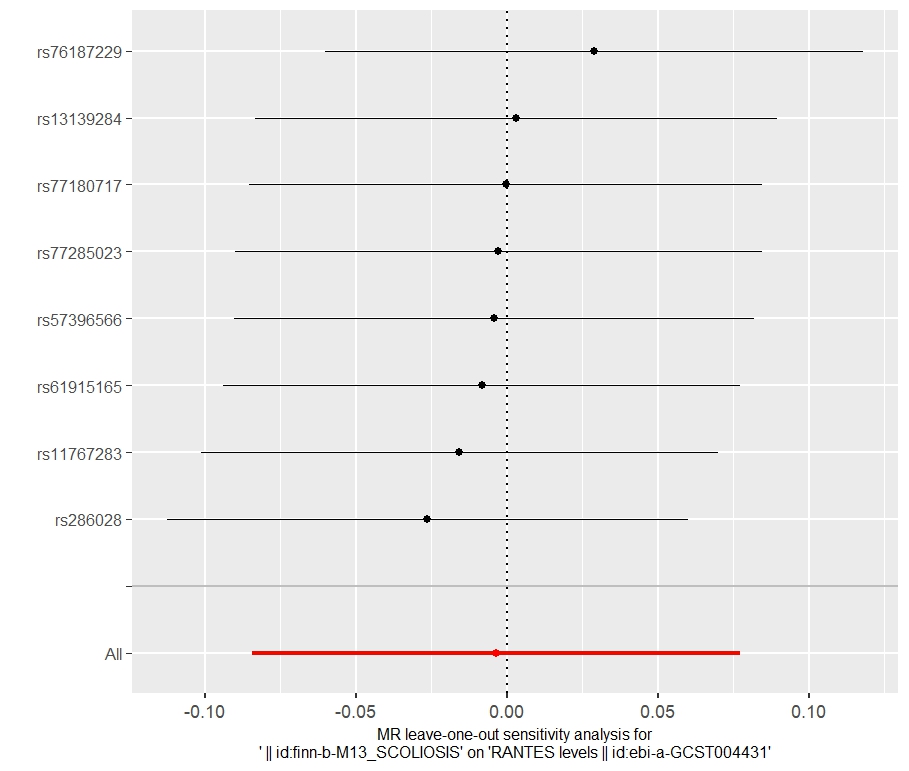

Supplement: Supplementary file 4 — Supplementary Material 4. [file JSP2-7-e70019-s002.zip › Supplementary Material 4/Exposureú║Scoliosisú1⁄4Outcomeú║inflammatory cytokines/RANTES/Supplementary Material 4 RANTES 2.jpeg]

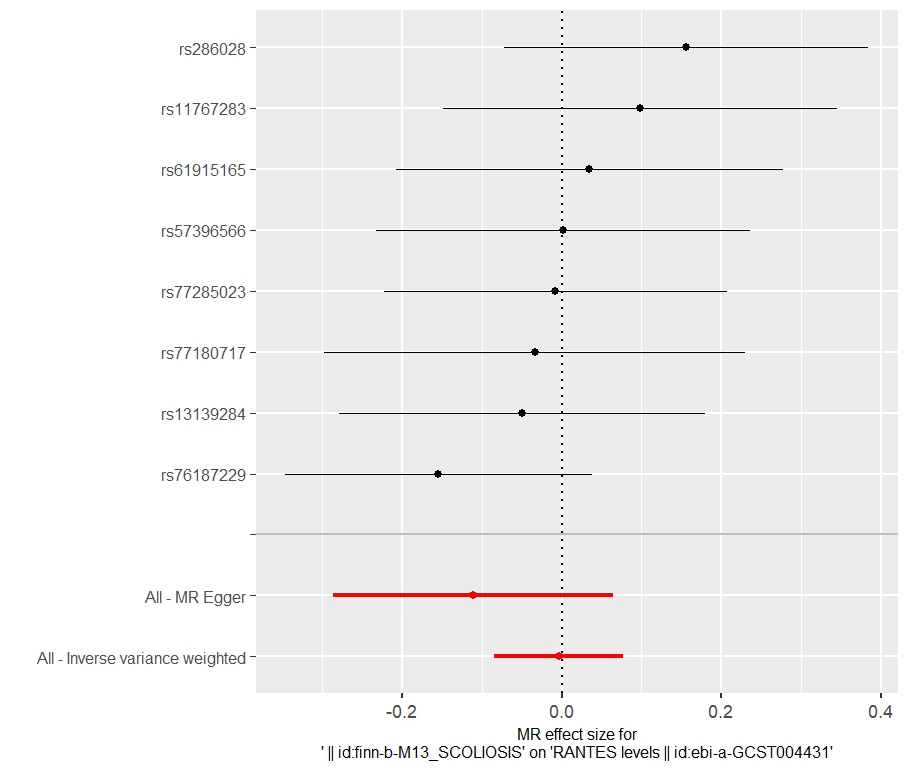

Supplement: Supplementary file 4 — Supplementary Material 4. [file JSP2-7-e70019-s002.zip › Supplementary Material 4/Exposureú║Scoliosisú1⁄4Outcomeú║inflammatory cytokines/RANTES/Supplementary Material 4 RANTES 3.jpeg]

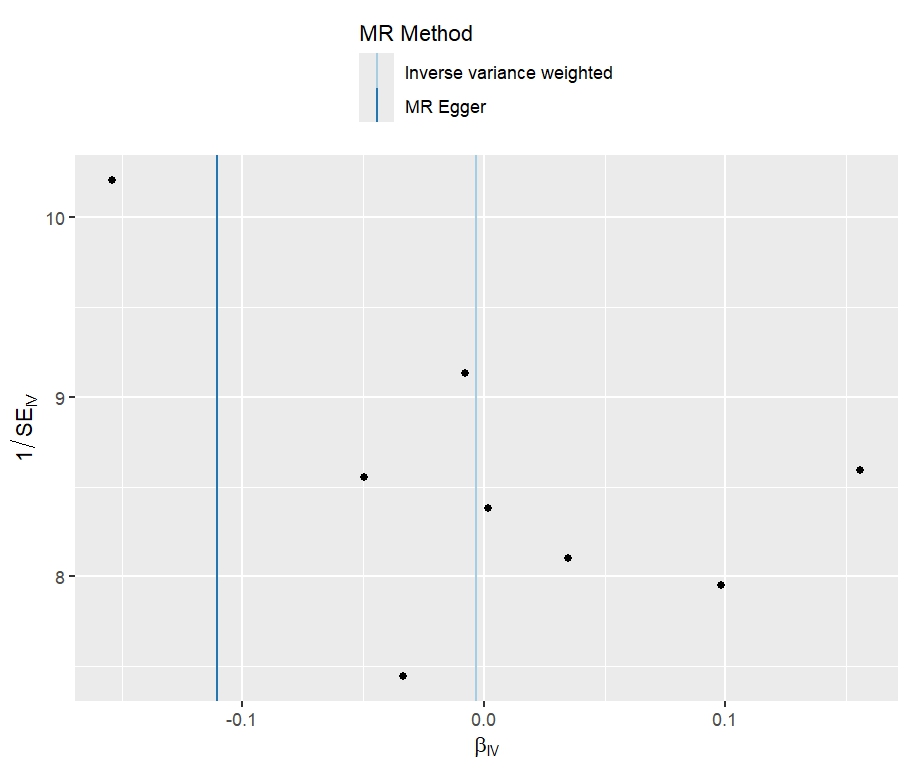

Supplement: Supplementary file 4 — Supplementary Material 4. [file JSP2-7-e70019-s002.zip › Supplementary Material 4/Exposureú║Scoliosisú1⁄4Outcomeú║inflammatory cytokines/RANTES/Supplementary Material 4 RANTES 4.jpeg]

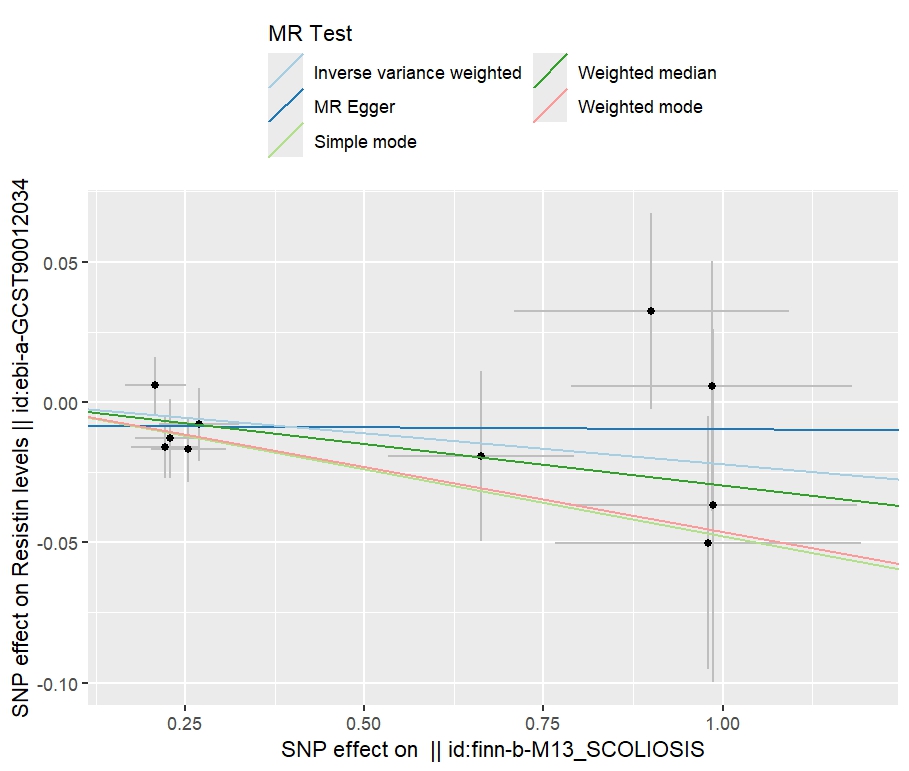

Supplement: Supplementary file 4 — Supplementary Material 4. [file JSP2-7-e70019-s002.zip › Supplementary Material 4/Exposureú║Scoliosisú1⁄4Outcomeú║inflammatory cytokines/Resistin/Supplementary Material 4 Resistin 1.jpeg]
